# Re-expressing coefficients from regression models for inclusion in a meta-analysis

**DOI:** 10.1186/s12874-023-02132-y

**Published:** 2024-01-08

**Authors:** Matthew W. Linakis, Cynthia Van Landingham, Alessandro Gasparini, Matthew P. Longnecker

**Affiliations:** 1 Ramboll U.S. Consulting, Raleigh, NC 27612 USA 3214 Charles B Root Wynd #130,; 2 Ramboll U.S. Consulting, Monroe, LA 71201 USA; 3 Red Door Analytics AB, Stockholm, Sweden

**Keywords:** Conversion, Meta-analysis as topic, Regression analysis, Transformation

## Abstract

Meta-analysis poses a challenge when original study results have been expressed in a non-uniform manner, such as when regression results from some original studies were based on a log-transformed key independent variable while in others no transformation was used. Methods of re-expressing regression coefficients to generate comparable results across studies regardless of data transformation have recently been developed. We examined the relative bias of three re-expression methods using simulations and 15 real data examples where the independent variable had a skewed distribution. Regression coefficients from models with log-transformed independent variables were re-expressed as though they were based on an untransformed variable. We compared the re-expressed coefficients to those from a model fit to the untransformed variable. In the simulated and real data, all three re-expression methods usually gave biased results, and the skewness of the independent variable predicted the amount of bias. How best to synthesize the results of the log-transformed and absolute exposure evidence streams remains an open question and may depend on the scientific discipline, scale of the outcome, and other considerations.

**Supplementary Information:**

The online version contains supplementary material available at 10.1186/s12874-023-02132-y.

## Introduction

The results of a group of studies deemed comparable can be synthesized quantitatively using meta-analysis. To base the meta-analysis on all available data, “Results extracted from study reports may need to be converted to a consistent, or usable, format for analysis” [1]. Methods of converting data presented by authors into a format suitable for meta-analysis have been well developed for effect sizes based on categorical representation of exposure**.** Our focus here, however, was on continuous measures of exposure, for which such methods are somewhat limited [2].

Our particular interest was in re-expression of results so that they could be included in a meta-analysis that could best inform a risk assessment. More specifically, the element of a risk assessment that we focused on in this work was meta-analysis to support a dose–response assessment [3]. Dose–response assessment in risk assessment is conducted so that the risk associated with any specific amount of an exposure can be examined [4]. When a dose–response evaluation is based on meta-analytic results, such results are more straightforward to relate to a specific exposure level if derived from models with exposure on an absolute, untransformed value. While our analyses also speak to matters related to the hazard assessment element of a risk assessment, these are addressed in our discussion. At any event, in conducting meta-analysis of exposure effects that might inform a risk assessment, often one encounters some original data reports where models of outcome were fitted in relation to log of exposure and others fitted in relation to absolute exposure, posing challenges to synthesis.

Various approaches to the problem of inconsistently-expressed effect estimates have been recommended or used in practice [5–8]. Obtaining the raw data or asking authors to re-analyze their data are the ideal solutions, though not always practical. When these options are not feasible, the results from studies using the less-frequent approach have been be excluded from the meta-analysis [6], or preferably the results of studies that used transformed and original units are analyzed separately [7, 8], and then synthesized without meta-analysis (SWiM) [5]. Some authors, however, have recently used re-expression methods to address the problem [9, 10]. The validity of these re-expression methods, however, has not been evaluated in detail. Here we consider methods of re-expressing regression coefficients from linear models fit to a log-transformed exposure variable as the coefficient that would have been obtained had the authors left the exposure in its original units. We refer to this process as re-expression of *β* to an untransformed basis.

**Fig. 1:**
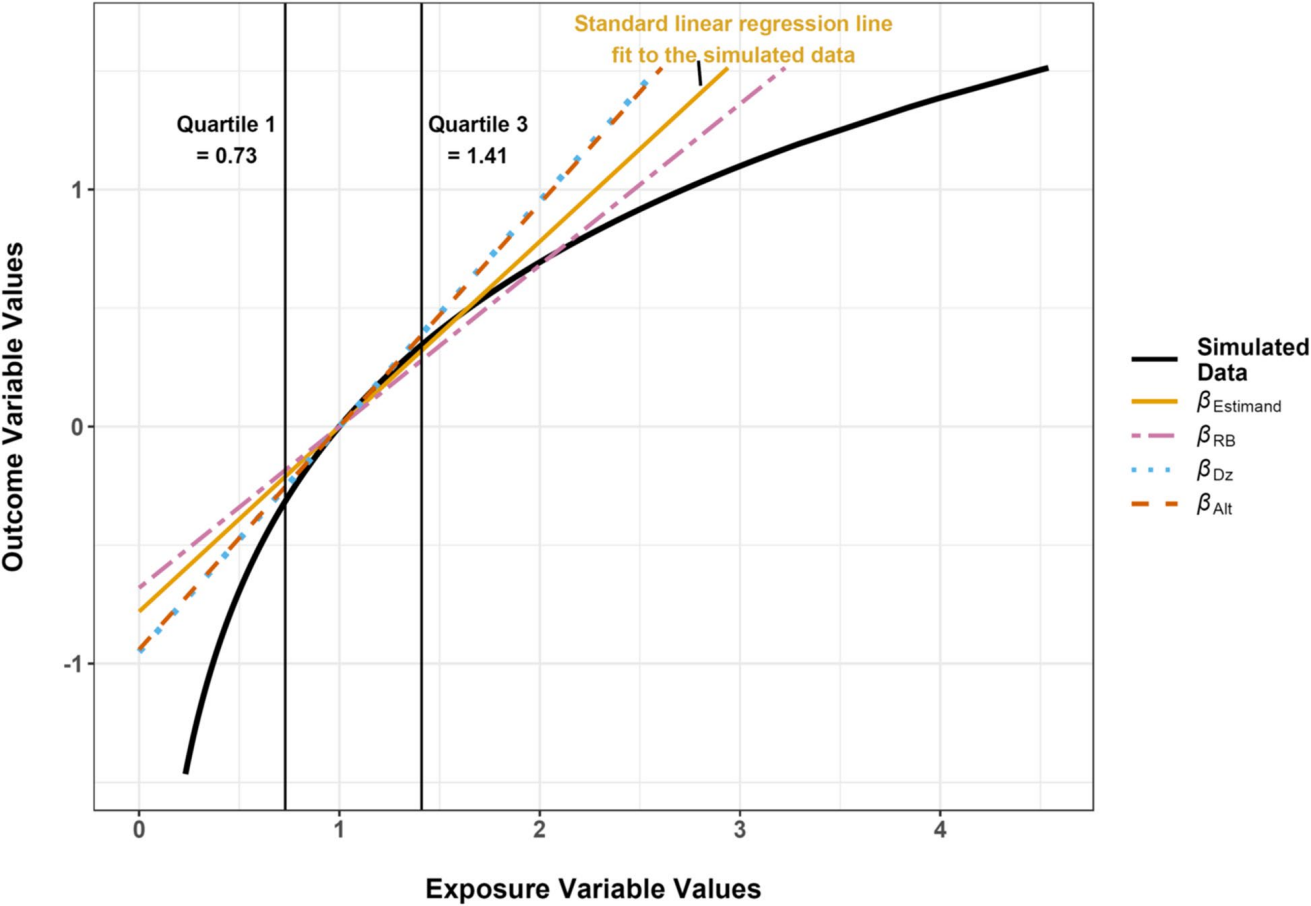
Plot of simulated values of *y* as a function of *x* from *y* = ln(*x*)(curve), along with slopes obtained by four methods (diagonal lines). The gold solid line represents a slope (*β*_Estimand_) fitted with the model y = α + βx. The three dashed lines are estimates of *β*_Estimand_ obtained by the re-expression methods described in the text. Vertical lines indicate the first and third quartiles of the x-values. The intercepts of the diagonal lines have been adjusted to emphasize the similarity of the slopes in the interquartile range

An algebraic method of re-expressing regression coefficients was recently described and evaluated using one simulated data set and one set of parameters [11]. Rodriguez-Barranco et al. found that in the setting of a log-transformed lognormally distributed independent variable, when the *β* coefficient from a model fit to the transformed data was re-expressed to what they would have gotten had the model been fit to the untransformed data, the re-expressed coefficient was half the size of the true (fitted) coefficient. They recommended caution in applying their method when the distribution of the independent variable was markedly asymmetric. More recently, other authors have developed computational methods of re-expressing coefficients from models fit to a log-transformed independent variable to approximate what would have been obtained if the model had been fit with the original unit continuous independent variable [9, 10]. The basic principle is to minimize the difference between the *y* predicted from *y* = *β*·log(*x*) and the *y* predicted from a *y* = *β*·*x* (over the same range of *x*) by varying *β* in the second equation. Figure 1 may aid visualization of the task, where *y* from *y* = *β*·log(*x*) is shown with a light blue dotted line, and the difference in *y* from a straight line is minimized over a range of *x*. When Steenland et al. originally described the procedure it was for a fixed range of *x*, applicable to a specific exposure, and the validity of their method was not evaluated. Dzierlenga et al. (2020) used the same basic principle as Steenland et al. with a modification of the method to be more flexible with respect to the range of the exposure variable and found that it performed well when evaluated using data from five studies of one exposure. In addition to the above re-expression methods, we developed a third (“Alternative”) estimator that is algebraic but different than that of Rodriguez-Barranco et al., and introduce it below, in the methods section.

The goal of the present project was to evaluate the validity of re-expression of regression coefficients to an untransformed basis for three methods using a wide variety of simulated and real data examples. To provide a context for interpretation of our results we have designated an amount of relative bias that we considered important. We note that an acceptable magnitude of bias is often not quantitated in reports like ours (e.g., [12, 13]). Reluctance to define general-use cutoffs for acceptable bias is also reflected throughout the epidemiologic literature; for example see ROBINS-E material on confounding [14]. Nonetheless, Freidrich et al. (2008), in their simulation study, defined bias as a ≥ 5% difference from an estimand [15]. A well-regarded textbook, Modern Epidemiology, 3rd Ed., p 261: gives a 5–10% difference in effect estimates as an amount that might be considered important, but they note (p. 262) that “the exact cutoff for importance is somewhat arbitrary but is limited in range by the subject matter” [16]. At any event, for the purposes of the present investigation, we considered a bias of ≥ 5% as reflecting an undesirable property of an estimator.

## Methods

In this section, we present the simulation study that was used to evaluate the three estimators, and then describe the real datasets that were used to further evaluate the estimators. Our description of the simulation study follows the “ADEMP” format recommended by Morris et al. (2019), where ADEMP stands for Aims, Data generating mechanism, Estimand (target of analysis), Methods, and Performance measures [17]. The methods subsection of the ADEMP gives a detailed specification of the estimators and is thus relatively long.

### Description of the simulation in ADEMP format

#### Aims

To examine bias in and coverage of the estimated regression coefficients (regression coefficient that would have been obtained had the original analysts not log-transformed exposure before fitting a regression model) calculated by three methods.

#### Data generating mechanism (DGM)

An independent random variable *x* with a log normal distribution used to define the dependent variable *y* = *β*_DGM_·log_*b*_(*x*) + *e*. The model parameters, possible values, and rationale for the chosen values are shown in Table 1 [18]. A *β*_DGM_ = 0 was not studied in the simulations because it caused instability in the relative bias performance measure. A range of *σ*, the standard deviation of the log-transformed exposure values, was chosen to cover the approximate range observed in the 15 real data studies. A factorial simulation design was used with the parameter values indicated in Table 1. Specifically, every possible combination of parameter values was used, for a total of 960 simulation scenarios (each with n_sim_ = 2000).

**Table 1:** Values of parameters used in the simulation and rationale for their choice. Number of simulations = 2000

Parameter	Possible values	Rationale for the choice
*n*_obs_	162, 8474	Quantiles (10th, 90th) of the distribution of sample sizes for the 15 real data examples
*e*	Selected from a normal distribution with mean = 0 and SD = SD_*e*_^a^	Standard deviation selected to result in an R^2^ ~ 0.2, to make realistic models (Aslibekyan et al., 2014) [17]
*β*_DGM_	-15, 0.5, 1, 10, 30	Broad range of effect sizes encompassing those in the real data examples
Logbase	2, e, 10	Log bases used in the 15 real data examples
*σ*	0.25, 0.45, 0.65, 0.85	Selected values cover the approximate range of *σ* values in the 15 real data examples
Median *(µ* = log(median))	0.25, 0.5, 1, 2, 4, 8, 16, 32	Selected values cover the range of median exposure in the 15 real data examples

^a^SD_e_ was calculated using the following method: SD_e_ =|*β*_DGM_ * log(median + 2) * Mult_SD_| where *β*_DGM_ and median are from the table above and Mult_SD_ is a multiplier to prevent extremely large variability. For each median, the Mult_SD_ was as follows: median 0.25, Mult_SD_ 1; 0.5, 0.886; 1, 0.771; 2, 0.657; 4, 0.543; 8, 0.429; 16, 0.315; 32, 0.120). Mult_SD_ was selected based on values that will introduce enough error to result in r^2^ values around 0.2 (similar to many epidemiological studies) for estimation of β_Estimand_

#### Estimand

The *β* coefficient from fitting *y* = *β*_Estimand_·*x* + *e* with an ordinary least squares (OLS) model.

#### Methods

The three estimators evaluated were: 1) as described by Rodriguez-Barranco et al. (2017), 2) as described by Dzierlenga et al. (2020), and 3) an approach we introduce below and call the Alternative estimator. We refer to these as *β*_RB_, *β*_Dz_, and *β*_Alt_, respectively.

An algebraic method for re-expression of *β* to an untransformed exposure was first presented by Rodriguez-Barranco et al. (2017). Equation 1 below shows their formula (see Model B in Table 1 of their publication):

1 $${\beta }_{RB}={log}_{b}\left(1+ \frac{c}{E\left[X\right]}\right)* {\beta }_{\mathrm{from model} y = \beta \cdot {\text{log}}\left(x\right)}$$

In Eq. 1, *β*_RB_ is the re-expressed *β* coefficient using the Rodriguez-Barranco method, *b* is the log base used to transform *x*, *c* is the absolute change in exposure *x* (*c* = 1 unit of exposure in the present study), E[*X*] is the mean of the exposure, and *β*_*…*_ is the regression coefficient from the model using the log-transformed exposure. The same formula was applied to the confidence limits of *β* from the log(*x*) model.

A computational method for re-expression of *β* to an untransformed exposure was developed by Steenland et al. (2018), who described their method as

… iteratively minimizing the squared deviation of a new linear curve from the original logarithmic one, over a scale of 0 to 10 ng/ml PFOA [perfluorooctanoic acid], typical of studies in the general population. We also minimized squared deviation of a linear upper and lower confidence limit from the original logarithmic confidence interval curves. For any given study, the iteration was conducted by minimizing the sum of squares of the difference between the candidate linear curve and the logarithmic curve reported in the literature, across 10 points, at 1, 2…. through 10 ng/ml. Iteration began with an educated guess for a candidate linear curve that would approximate the logarithmic curves and proceeded by varying the candidate linear curve until the sum of squares of the differences were minimized.

Dzierlenga et al. (2020) used this same principle to calculate *β*_Dz_, though it modified it so that the method was more flexible with respect to the range of the exposure variable. The modification used an algorithmic optimization over 6 points from the 25th to the 75th percentiles (25th, 35th…75th) of the estimated exposure distribution.

The Alternative method of algebraic re-expression that we developed for this report was based on the principle of calculating, on the untransformed scale of exposure, the increment that represented a doubling, a 2.718-fold increase, or a tenfold increase (i.e., one log unit, with a base of 2, e, or 10). This was done by subtracting or adding 0.5 units on the log scale to the log(median exposure), back-transforming the results, and taking the difference (see Eq. 2).

2 $$I={b}^{{log}_{b}\left(median\right)+0.5}-{b}^{{log}_{b}\left(median\right)-0.5}$$

In Eq. 2, *I* is the increment used to re-express *β* from log to linear and *b* is the logarithm base. Then

3 $${\beta }_{{\text{Alt}} }={\beta }_{\mathrm{from model withlog}\left({\text{x}}\right)/I}$$

The same formula was applied to the confidence limits of *β* from the log(*x*) model. R scripts/functions and data files for applying each of these three re-expression methods are available in the supplemental materials (Supplemental_Code.zip).

#### Performance measures

We focused on relative bias, coverage probability, and the Monte Carlo standard error of the relative bias for each estimator. An example of the formula for the mean relative bias for a given scenario is:

4 $$Relative\, Bias=\frac{1}{{n}_{sim}}\sum_{i=1}^{{n}_{sim}}\frac{{\beta }_{{RB}_{i}}- {\beta }_{{estimand}_{i}}}{{|\beta }_{{estimand}_{i}}|}$$

where, e.g., ${\beta }_{{RB}_{i}}$ refers to the *β* coefficient obtained from the Rodriguez-Barranco et al. estimator for the *i*^th^ repetition, ${\beta }_{{estimand}_{i}}$ refers to the *β* coefficient obtained from the ordinary least squares estimator on the untransformed, simulated data, and *n*_sim_ is the number of simulations conducted. Absolute value of the β_estimand_ is used as the denominator in order to generate the correct sign for the absolute bias when both β values are negative. β_estimand_ is used rather than β_DGM_ to calculate the relative bias in Eq. 4 so that the results reflect the performance of the estimator(s) in specific datasets. An example formula for the Monte Carlo standard error of the relative bias for a given scenario is:

5 $$Monte\, Carlo\, SE\, of\, Relative\, Bias=\sqrt{\frac{1}{{n}_{sim}\left({n}_{sim}-1\right)}\sum_{i=1}^{{n}_{sim}}{\left(\frac{{\beta }_{{RB}_{i}}-{\beta }_{{estimand}_{i}}}{\left|{\beta }_{{estimand}_{i}}\right|}-mean\, of\, relative\, bias\right)}^{2}}$$

Evaluation of the determinants of relative bias using the simulated data.

After running the simulations using the parameter values shown in Table 1, for each estimator we fit ordinary least squares models of the relative bias as a function of median, *σ*, *b* (log base), *n*_*obs*_ (number of observations), and *β*_*DGM*_, and interaction terms between *σ* and these variables. A both-directions stepwise approach was taken where the multiple of the number of degrees of freedom used for the penalty (k) was set to a value ~ 3.84 (*p* < 0.05 in Chi-square test) and the optimal model was selected by minimization of the AIC value [19]. Each observation in the dataset analyzed was the average result from 2000 simulations. Use of the average rather than the data for all 1,920,000 (960·2000) observations resulted in essentially the same models and produced more interpretable plots.

### Evaluation of the validity of the three estimators using real data

To further evaluate the validity of re-expression methods and guide our simulations, we sought examples for various types of outcomes (dichotomous, log-continuous, untransformed continuous) and a variety of environmental agents with exposure measured using a biomarker. Environmental exposures measured with a biomarker frequently are used in risk assessment and often have skewed distributions with a long tail to the right. We first identified a series of published analyses based on data that were publicly available. Second, we identified a similar series of published analyses that did not have raw data available but that presented regression results obtained with and without log transformation of the exposure.

For the example data that involved our re-analysis of published results, we chose results that could efficiently be replicated to a reasonable degree of accuracy using the originally described methods. When the authors presented results for more than one outcome or more than one exposure in a report, in general we arbitrarily chose one result that was statistically significant for inclusion in our evaluation; the exception was data from Xu et al. (2020), for which we included two results. Xu et al. (2020) showed results for two different outcomes, one continuous, and one dichotomous, that were examined in relation to the same exposure; the regression coefficients were statistically significant for both. A more detailed description of the methods of identifying the real data examples is in Suppl. Methods Sect. 1.

For each real data example, we calculated the relative bias for each of the three estimators (compared to the coefficient from models using the untransformed exposure), and then for the 15 examples calculated the median, quartiles, and range of relative bias values for each estimator.

In the two example datasets where the relative bias in the three estimators was largest, we explored whether the exclusion of influential observations affected the accuracy of the re-expression using *β*_Dz_. In two additional examples datasets where the relative bias was typical of other studies, we also examined the effect of excluding influential points on the validity of the re-expression with* β*_Dz_. Influential observations were identified with a difference in *β* analysis (change in *β* with each observation excluded one at a time) performed on the regression using untransformed exposure. A t-test-like statistic was used to identify the 5% of points that were unusually influential (|DFBETAS|> 2/√n) [20]. In addition, to evaluate whether our results were sensitive to the specific results selected as real data examples from the 15 reports, in each report we enumerated all results eligible for inclusion in our analysis, and selected one at random (regardless of statistical significance); when only two such results were available, however, we selected the one not previously selected. We refer these additional results below as the second set of real data examples. Please see Suppl. Methods Sect. 2 for more details.

### Adjustment for bias in the estimators

The regression equations we developed to evaluate the determinants of relative bias in the simulated data (Sect. 2.2) were used to predict the relative bias in each estimator based on σ and other parameters, as needed. The predicted relative bias was used to estimate what the value of the estimator would have been were it not biased, e.g., *β*_Alt,adjusted_ = *β*_Alt_/(1 + predicted relative bias of *β*_Alt_). We applied this to the real datasets, to see if the adjustment resulted in an estimator with less relative bias.

## Results

### Simulations

A simplified example simulation with data generated by *y* = *β*_DGM_·log_e_(*x*) and parameters *β*_DGM_ = 1, median = 1, *σ* = 0.5, *SD*_e_ = 0 is depicted in Fig. 1. In this scenario *β*_RB_ slightly undershot the slope estimated from the fitted regression line, whereas the *β*_Dz_ and *β*_Alt_ estimators overshot the fitted slope, by a slightly greater magnitude. The range of parameter values in the simulation and original set of real data examples overlapped substantially (Table 1, Suppl. Table S1).

**Fig. 2:**
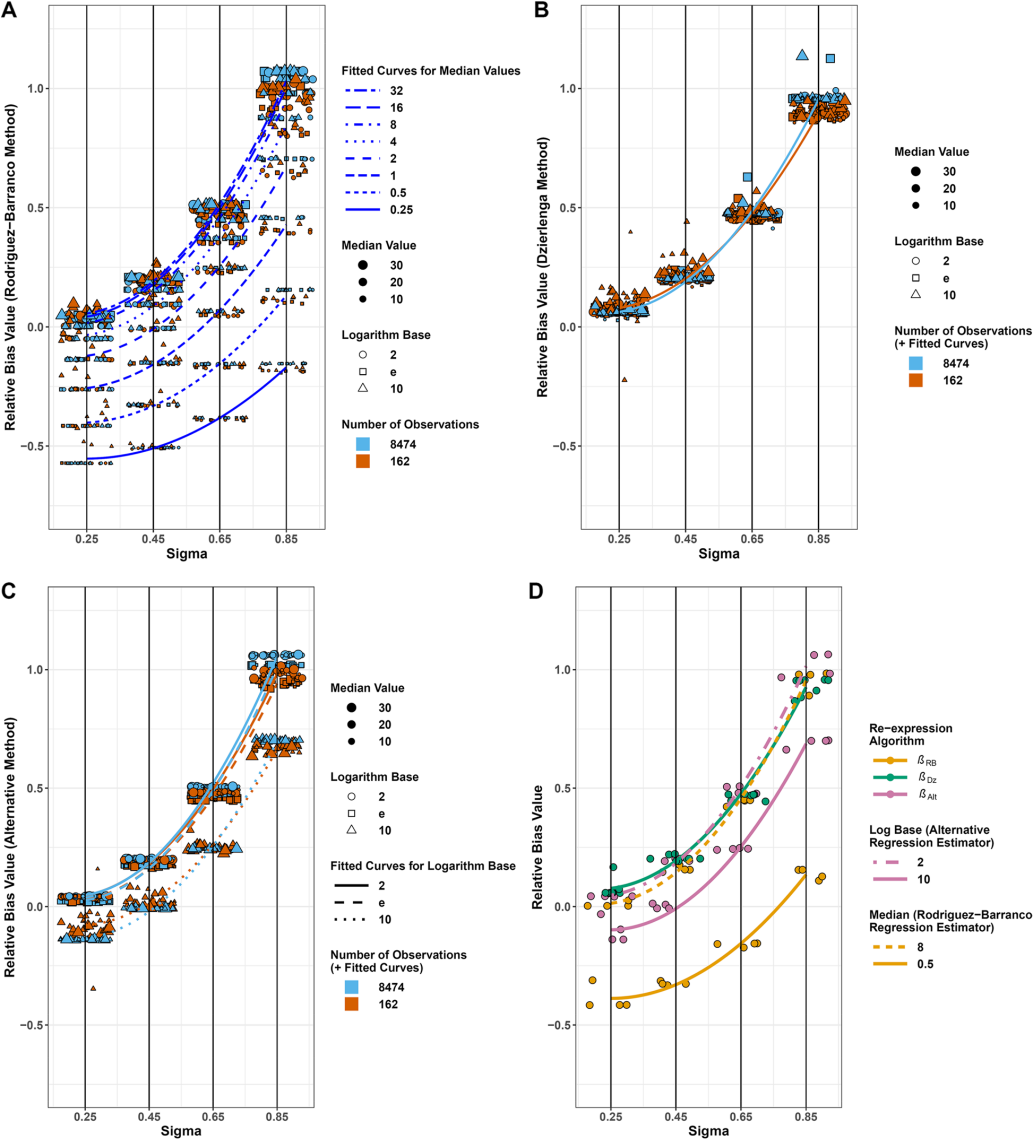
Plots of relative bias as a function of skewness (*σ*) in the exposure *x*, by type of estimator. Individual points represent the average result (*n*_sim_ = 2000) for each simulation scenario. A total of 890 of the possible 1,920,000 observations (960 scenarios × 2000 simulations) were not used in the calculation of the average results because *β*_estimand_ was < 0.0001 (essentially zero). Lines represent quadratic fits to the data for a specified prediction equation and set of values of independent variables (see text). Note that data have been artificially spread along the x-axis for visualization purposes, all actual x-values are the closest black vertical line (0.25, 0.45, 0.65, or 0.85). Figures **A**-**C** show points for 768 simulation scenarios (β_DGM_ > 0, see Figure S1 for plots including β_DGM_ < 0); Figure D shows points for a subset of scenarios (*n* = 32) chosen because they demonstrate differences among the estimator properties. **A** Rodriguez-Barranco estimator, **B** Dzierlenga estimator, and **C** Alternative estimator. **D** Shows all 3 estimators in the same plot with a subset of the data of the simulation data where *β*_DGM_ = 0.5, log base = 2 or 10, and median value = 0.5 or 8

The relative bias of *β*_RB_ was a function of *σ* and the median exposure level (Fig. 2A). When *x* was significantly skewed (e.g., *σ* = 0.65) and the median was 1, the relative bias was close to zero, but with other combinations of *σ* and median the range of bias was substantial. The coefficients for the model of relative bias in *β*_RB_ are shown in Table S2.

The relative bias of *β*_Dz_ was primarily a function of *σ* (Fig. 2B, Table S2). Within the parameter space investigated, the absolute difference in relative bias due to the interaction of *σ* and *n*_obs_ was, with *n*_obs_ = 8474 (cf.* n*_obs_ = 162), < 0.05 (not shown).

 The relative bias of *β*_Alt_ was primarily a function of *σ* and log base (Fig. 2C, Table S2). When log base was 10, at a given value of *σ* the relative bias was lower than when log base was 2 or e. When log base was 2 or e, *β*_Alt_ performed similarly to *β*_Dz_.

As noted earlier, the models of relative bias for each estimator were fit to datasets with an n of 960, where each of the 960 observations was the average of 2000 simulations for each scenario (parameter set). When the same models were fit to all of the original data points (960·2000) the model fit statistics were essentially the same (Table S3).

Figure 2D shows the relative bias after restricting the parameter set for the simulation to best display the key properties of each estimator: all depend on *σ*,* β*_RB_ additionally depends on the median, and *β*_Alt_ additionally depends on the log base. The overall interpretation based on the figure was that in the simulations in general, with *σ* > 0.45 the estimators were substantially biased except for specific circumstances where *β*_RB_ did well. Another way to summarize the overall findings was by the performance measures presented in Table 2, based on the results for all simulation scenarios with |β_Estimand_|> 0. The median Monte Carlo standard error (MCSE) across all three estimators was 0.002. Thirty percent of the 2880 simulations (960 scenarios ·3 estimators) had an MCSE > 0.005. More than 95% of all 2880 simulations had an MCSE that was ≤ 0.02 (relatively small compared with the average relative bias). Among the < 5% with an MCSE > 0.02, the n_obs_ was 162 and the log base was 10 in all instances. The maximum MCSEs were: β_RB_, 0.196; β_Dz_, 0.320; and β_Alt_, 0.262. The coverage probabilities were substantially below 95%, reflecting how infrequently the estimators performed well. Compared with *β*_RB_, the other two estimators, on average, had larger positive bias, but with higher coverage probabilities.

**Table 2:** Performance measures based on simulations with all possible values for each parameter for a total of 960 simulation scenarios with 2000 simulations per scenario (*n* = 1,919,110)^a^

Estimator	Average absolute relative bias	IQR of absolute relative bias	Average coverage probability (%)^b^
*β*_*RB*_	0.362	0.120–0.499	36.5
*β*_*Dz*_	0.430	0.112–0.597	39.4
*β*_*Alt*_	0.389	0.090–0.558	45.5

^a^A total of 890 of the possible 1,920,000 observations were not used in the calculations because *β*_estimand_ was < 0.0001 (essentially zero)

^b^Calculated as the sum of the replicates where the re-expression method confidence interval included the observed *β* (*β*_estimand_) divided by the total number of 1,919,110 replicates

Because the regression analysis indicated that the main determinants of bias were σ, median, and log base for one or more of the estimators, for each estimator we examined coverage in relation to two values of these three parameters (Table 3). In general, as the average relative bias increased, the coverage decreased. The coverage tended to be better with exposure re-expressed by the original authors using a log base 10 than log base 2.

**Table 3:** Performance measures based on simulation scenarios with stated values of σ, median, and log base for a total of 8 simulation scenarios^a^ (n_sim_ = 2000 per scenario^b^)

σ	Median	Log base	Estimator	Average relative bias	Average coverage probability (%)^c^
0.45	2	2	*β*_*RB*_	-0.008	99.1
			*β*_*Dz*_	0.199	78.7
			*β*_*Alt*_	0.2	78.6
0.45	2	10	*β*_*RB*_	0.021	99.9
			*β*_*Dz*_	0.231	99.9
			*β*_*Alt*_	0.019	99.9
0.45	16	2	*β*_*RB*_	0.182	81.9
			*β*_*Dz*_	0.195	79.5
			*β*_*Alt*_	0.195	79
0.45	16	10	*β*_*RB*_	0.21	100
			*β*_*Dz*_	0.224	99.9
			*β*_*Alt*_	0.012	100
0.85	2	2	*β*_*RB*_	0.655	2.4
			*β*_*Dz*_	0.901	0.2
			*β*_*Alt*_	1.003	0
0.85	2	10	*β*_*RB*_	0.678	56.7
			*β*_*Dz*_	0.927	37.4
			*β*_*Alt*_	0.676	57.1
0.85	16	2	*β*_*RB*_	0.979	0
			*β*_*Dz*_	0.898	0.1
			*β*_*Alt*_	1	0
0.85	16	10	*β*_*RB*_	0.987	31.4
			*β*_*Dz*_	0.91	36.9
			*β*_*Alt*_	0.658	56.9

^a^For all scenarios used in this table the *n*_obs_ was 162 and the β_DGM_ was 1

^b^The median Monte Carlo standard error of the relative bias was ≤ 0.003 for all estimation methods

^c^Calculated as the sum of the scenarios where the re-expression method confidence interval included the observed *β* (*β*_estimand_) divided by the total number of scenarios (960)

### The real data and application of the estimators to it

We identified nine published analyses of data for which the raw data were publicly available and that met our criteria for selection (Table S4, Table S5 for second set of real data) [21–28]. The results of our re-analyses were generally the same order of magnitude as those originally published (Tables S4 and S5). The specific finding that we used in the analysis and its location in the original publication are listed in Supplementary Material Table S6 (Table S7 for second set of real data), as are the median, quartiles, and mean of the exposure distributions, which were estimated in some cases as indicated by table footnotes. We identified six published analyses of data where the original authors presented regression results using exposure with and without a log-transformation (Table S8, Table S9 for second set of real data) [10, 29–33]. Five of these were included in the assessment of validity by Dzierlenga et al. (2020) [9]. Among the fifteen example studies, a variety of outcomes and exposure variables were examined, though in two thirds of the studies the exposure was a perfluoroalkyl substance (either PFOA, perfluorohexanesulphonic acid, or perfluorooctane sulfonic acid).

When *β* was re-expressed as if it had been fit to untransformed exposure data, the range in relative bias across all three estimators was -0.5 to 16.8 (Table 4) and the interquartile ranges in relative bias were relatively wide. In the comparison of results for specific studies across re-expression methods, the relative bias was, for most of the studies, similar across methods (Table 4). These were studies where the median exposure was > 4 units (Table S6) – as would be expected based on Fig. 2D. For the Lee et al. (2020) and the two Xu et al. (2020) results [22, 24], however, *β*_RB_ had a much smaller relative bias than the other two methods. In these three instances, the median of the exposure variable was less than one, which was not the case for the other studies (see Supplementary Materials Table S6) and the *σ* was > 0.8 – which is the setting where the relative bias in *β*_RB_ was expected to be relatively small compared with the other estimators.

**Table 4:** Comparison of fitted and re-expressed *β* coefficients and relative bias in *β* for three methods of re-expression

First author, year	*β*_Estimand_ from analysis of raw data	*β*_RB_^a^	Relative Bias^b^	*β*_Dz_	Relative Bias^b^	*β*_Alt_^c^	Relative Bias^b^
Abraham, 2020 [28]	-0.0636 log_e_/ ng·ml^−1^	-0.0324	-0.49	-0.0368	-0.42	-0.0384	-0.40
Apelberg, 2007 [29]	-12.9 g/(ng/ml)	-11.7	-0.10	-13.2	0.03	-13.2	0.03
Bulka, 2021 [21]	0.015 log_e_(OR)/ ng·ml^−1^	0.043	1.87	0.0512	2.41	0.0531	2.54
Cheang, 2021 [27]	0.192 mg·dl/pmol·g^−1^	0.335	0.74	0.352	0.83	0.353	0.84
Chen, 2012 [32]	-11.3 g/(ng/ml)	-16.3	0.44	-17.9	0.58	-17.8	0.58
Darrow, 2013 [33]	-2.3 g/(ng/ml)	-1.95	-0.15	-2.02	-0.12	-2.00	-0.13
Hamm, 2010 [31]	1.5 g/(ng/ml)	3.66	1.44	3.92	1.62	3.85	1.57
Lee, 2020 [22]	1.444 log_e_(OR)/μg·dl^−1^	1.31	-0.09	3.30	1.28	3.46	1.4
Odebeatu, 2019 [23]	6.86 × 10^–4^ log_e_(OR)/ng·ml^−1^	0.0114	15.69	0.0122	16.82	0.0116	15.86
Pilkerton, 2018 [26]	-0.0049 log_e_/ng·ml^−1^	-0.0207	3.22	-0.0302	5.17	-0.0306	5.24
Steenland, 2009 [10]	0.00105 log_e_/ ng·ml^−1^	0.00116	0.11	0.00127	0.21	0.00126	0.20
Stein, 2016 [25]	-0.0039 log_e_/ng·ml^−1^	-0.00676	0.73	-0.0069	0.77	-0.00694	0.78
Washino, 2009 [30]	-10.94 g/(ng/ml)	-11.4	0.04	-12.1	0.10	-10.0	-0.08
Xu, 2020a [24]	0.513 log_e_(OR)/ ng·ml^−1^	0.508	-0.01	0.962	0.87	1.01	0.96
Xu, 2020b [24]	29.3 mg·dl/ng·ml^−1^	25.4	-0.13	48.1	0.64	50.4	0.72
Median			0.11		0.77		0.78
First quartile			-0.095		0.155		0.115
Third quartile			1.09		1.45		1.485
Minimum			-0.49		-0.42		-0.40
Maximum			15.69		16.82		15.86

^a^ Using the notation of Rodriguez-Barranco et al., k = base of log transformation used; c = 1

^b^ Proportional difference between β in column to the left compared with the one from the analysis of raw data, calculated using the same method as in previous table ((beta in column to left – beta from analysis of raw data)/beta from analysis of raw data). Note that the β in column to the left was calculated using β with different units in denominator than for the raw data analysis shown in the table

^c^ Let the log unit increment I in untransformed units = *b*^(l*og*^_*b*_^(median) +0.5)^-*b*^(log^_*b*_^(median) – 0.5)^, where *b* = 2, e, or 10, depending on the base. For observed β_o_ with units of ∆y/∆log_*b*_(x), to get re-expressed β_r_ with units ∆y/∆x, calculate β_r_ = β_o_/I. If the units of β_o_ are ∆y/∆x, to get β_r_ with units ∆y/∆log_*b*_(x), calculate β_r_ = β_o_· I

Our results for Odebeatu et al. (2019) and Pilkerton et al. (2018) were the ones with the greatest discrepancy between the re-expressed *β* coefficients and the *β* fitted to the untransformed exposure [23, 26] (Table 4). This discrepancy suggested that there may have been observations that were influential, and that the influence was affected by whether the exposure had been log-transformed. Thus, we conducted an analysis of whether exclusion of influential points affected the accuracy of the re-expression. For comparison, similar analyses were conducted using the data from Cheang et al. (2021) and Xu et al. (2020) (dichotomous outcome), which showed smaller relative differences between the re-expressed and fitted *β*s. The analyses with and without the inclusion of especially influential points in the real data sets showed that the accuracy of the re-expression estimators was affected by their exclusion (Table S10). The relative bias was affected by the influential points more so for Odebeatu et al. (2019) and Pilkerton et al., (2019) than for Cheang et al. (2021) and Xu et al. (2020), but even with the exclusion of influential observations the re-expression methods still had a high relative bias.

As was true for the original set of real data examples, the range of parameter values in the simulation and second set of real data examples overlapped substantially (Table 1, Suppl. Table S1). When the relative bias of the re-expressed ϐ coefficients was examined using the second set of real data examples (Suppl. Materials, Table S1), the range of relative bias (-18.1 to 10.7) was greater than in the original set of real examples (-0.5 to 16.8), and the interquartile ranges were narrower for β_RB_ than for β_Dz_ and β_Alt_. In general, however, these distributions were all relatively wide, as in the original set of real data examples. For some studies the relative bias was similar across estimators (e.g., Abraham et al., 2020; Bulka et al., 2021; Darrow et al., 2013; Pilkerton et al., 2018; and Stein et al., 2016). As with the original set of data examples, agreement in degree of bias across re-expression methods tended to be higher when the median exposure was > 4. As before, a tendency for *β*_RB_ to have the lowest bias occurred when the median exposure was < 1 (Lee et al., 2020), especially when σ was > 0.8 (Odebeatu et al., 2019; Xu et al. 2020b). Similarly, *β*_Dz_ and *β*_Alt_ tended to have a smaller relative bias than *β*_RB_ when the median exposure was > 1 and σ was < 0.8 (e.g., Apelberg et al., 2008; Xu et al. 2020a). But median exposure and σ did not perfectly predict the lowest-bias estimator, and few results had a relative bias that was in the range of 0 ± 0.05.

When we used the regression equations (Table S2) to predict the relative bias in the estimators when applied to each of the real data examples, and then adjusted the re-expressed *β* to remove the bias, the adjusted *β*s, on average, showed less relative bias, but as before, the interquartile range of the adjusted relative bias was wide (Table 5).

**Table 5:** Comparison of fitted and re-expressed β coefficients and proportional difference in β for three methods of re-expression, adjusted by their OLS describing the relationship between sigma and relative bias

First author, year	*β*_Estimand_ from analysis of raw data	*β*_RB_^a^	Relative Bias^b^	*β*_Dz_	Relative Bias^b^	*β*_Alt_^c^	Relative Bias^b^
Abraham, 2020 [28]	-0.0636 log_e_/ ng·ml^−1^	-0.0202	-0.68	-0.0214	-0.66	-0.0214	-0.66
Apelberg, 2007 [29]	-12.9 g/(ng/ml)	-10.6	-0.18	-9.41	-0.27	-9.37	-0.27
Bulka, 2021 [21]	0.015 log_e_(OR)/ ng·ml^−1^	0.0339	1.26	0.0303	1.02	0.0301	1.01
Cheang, 2021 [27]	0.192 mg·dl/pmol·g^−1^	0.224	0.17	0.288	0.50	0.290	0.51
Chen, 2012 [32]	-11.3 g/(ng/ml)	-14.8	0.31	-12.8	0.14	-12.8	0.13
Darrow, 2013 [33]	-2.3 g/(ng/ml)	-1.69	-0.27	-1.56	-0.32	-1.56	-0.32
Hamm, 2010 [31]	1.5 g/(ng/ml)	3.79	1.52	3.24	1.16	3.24	1.16
Lee, 2020 [22]	1.444 log_e_(OR)/μg·dl^−1^	0.957	-0.34	1.79	0.24	1.79	0.24
Odebeatu, 2019 [23]	6.86 × 10^–4^ log_e_(OR)/ng·ml^−1^	0.00374	4.45	0.00361	4.26	0.00364	4.30
Pilkerton, 2018 [26]	-0.0049 log_e_/ng·ml^−1^	-0.021	3.26	-0.0232	3.74	-0.0231	3.71
Steenland, 2009 [10]	0.00105 log_e_/ ng·ml^−1^	0.00088	-0.16	0.00090	-0.14	0.00090	-0.14
Stein, 2016 [25]	-0.0039 log_e_/ng·ml^−1^	-0.00527	0.35	-0.00542	0.39	-0.00542	0.39
Washino, 2009 [30]	-10.94 g/(ng/ml)	-11.6	0.06	-9.34	-0.15	-9.40	-0.14
Xu, 2020a [24]	0.513 log_e_(OR)/ ng·ml^−1^	0.380	-0.26	0.535	0.04	0.534	0.04
Xu, 2020b [24]	29.3 mg·dl/ng·ml^−1^	19.0	-0.35	26.7	-0.09	26.7	-0.09
Median			0.06		0.14		0.13
First quartile			-0.265		-0.145		-0.140
Third quartile			0.805		0.760		0.760
Minimum			-0.68		-0.66		-0.66
Maximum			4.45		4.26		4.30

^a^ Using the notation of Rodriguez-Barranco et al., k = base of log transformation used; c = 1

^b^ Proportional difference between β in column to the left compared with the one from the analysis of raw data, calculated using the same method as in previous table ((beta in column to left – beta from analysis of raw data)/beta from analysis of raw data). Note that the β in column to the left was calculated using β with different units in denominator than for the raw data analysis shown in the table

^c^ Let the log unit increment I in untransformed units = *b*^(l*og*^_*b*_^(median) +0.5)-^*b*^(log^_*b*_^(median) – 0.5)^, where *b* = 2, e, or 10, depending on the base. For observed β_o_ with units of ∆y/∆log_*b*_(x), to get re-expressed β_r_ with units ∆y/∆x, calculate β_r_ = β_o_/I. If the units of β_o_ are ∆y/∆x, to get β_r_ with units ∆y/∆log_*b*_(x), calculate β_r_ = β_o_· I

## Discussion

In the simulations, the bias in each of the three estimators was evaluated in relation to the median of the exposure variable, the skewness in the exposure variable, the log base used to transform the exposure variable, the *β* in the model generating the data, and the n_obs_ simulated. For all three re-expression methods, the relative bias was more positive as the skewness of the exposure distribution increased. The relative bias in *β*_RB_ was also determined by the median of the exposure distribution, and the relative bias in *β*_Alt_ was also affected by the base of the log used to transform the exposure variable. Although a few specific circumstances were found where the relative bias in a given re-expression method was lower, in general, when the skewness of *x* was large enough that a log transformation might be applied, the methods gave results that were sufficiently biased that their use would not be advisable. The results from applying the re-expression methods to real datasets generally agreed with those from the simulation, but the relative bias was greater than predicted based on the simulations. The relative bias in the real data was not much affected by the exclusion of influential observations. The especially high relative bias of the re-expression methods in the case of the Odebeatu et al. (2019) data may have been due to the small size of the slope being re-expressed.

Rodriguez-Barranco et al. (2017) recognized the importance of skewness in causing bias in their estimator, though the degree of skewness in their simulations was not specified and only one median value was used. For the re-expression method proposed by Steenland et al. (2018), apparently it was assumed that if an exposure distribution had an upper bound near 10 units, their empirical re-expression method would be sufficiently accurate [10]. Our results suggested that the range of exposure was predictive of the validity of the re-expression only for the RB estimator. In a previous evaluation of bias in the Dzierlenga estimator [9], little bias was found. The five empirical data examples in that previous evaluation were all included in the present analysis. The relatively small number of empirical data studies in the previous evaluation may have led to an overly-optimistic appraisal of the method.

In this report we focused on re-expressing regression coefficients from linear models fit to a log-transformed exposure variable. We could have also addressed the opposite: re-expression of regression coefficients from linear models fit to the untransformed exposure variable. To simplify the manuscript, we did not address this opposite type of re-expression. In risk assessment, results based on untransformed exposure are usually of greatest use, hence our focus on expressing all results in absolute units.

The real data examples used to inform the parameter space in the simulations represented a limited range of subject matter. In other fields the parameter space may differ from what we investigated. For example, if the n_obs_ in a study exceeded 8474, then the interaction between n_obs_ and σ might have a larger effect on the relative bias of the Dzierlenga estimator than noted here. Furthermore, the informal nature of our process for identifying real data examples to inform the parameter space (Suppl. Methods Sects. 1 & 2) precludes generalizing our simulation results to all environmental epidemiology studies with exposure measured with a biomarker. Nonetheless, the range of parameter values was broad enough it seems likely that the results may apply to many environmental epidemiology and perhaps other studies where relatively little variance in the outcome is explained. Similarly, the results of using the re-expression methods on the real data examples cannot be generalized to all environmental epidemiology studies with exposure measured with a biomarker. Examination of results for the real data examples, however, provided insights into the behavior of the re-expression methods not provided by the simulations alone and suggested that in practice, none of the re-expression methods were likely to work well. We also recognize that the focus on outcome-exposure relations considered here was a simple linear relationship, and that the dose–response relation in a given study might be better represented with a quadratic or other function.

How best to synthesize the results of the log-transformed and absolute exposure evidence streams remains an open question and may depend on the scientific discipline, scale of the outcome, and other considerations. In fields such as economics and psychology, meta-analysis of correlation coefficients is a well-recognized approach that could be applied to the evidence synthesis problem discussed here [12]. Regression coefficients would need to first be re-expressed as correlation coefficients [13]. Meta-analysis of correlation coefficients when both the outcome and exposure are continuous variables is a widely used approach in some fields [34]. However, Pearson correlation coefficients depend on the variance of the outcome and exposure [35], which can vary across studies. In epidemiology, meta-analysis of correlations has been criticized because they can distort the results [36]. In the field of randomized clinical trials, meta-analysis of correlation coefficients has received scant discussion, while Synthesis Without Meta-analyses (SWiM) is well-accepted [2]. Our particular interest was in re-expression of results so that they could be included in a meta-analysis that could inform a risk assessment. In that context, the two relevant elements of a risk assessment are hazard identification and dose–response assessment. As noted in the introduction, when a dose–response evaluation is based on meta-analytic results, such results are more straightforward to relate to a specific exposure level if derived from models with exposure in absolute, untransformed units. For hazard identification the results of epidemiologic studies with exposure that has been log-transformed and those with exposure in absolute units are both informative and use of SWiM might be the best solution to the synthesis problem. For a dose–response assessment in environmental epidemiology the re-expression methods studied in the present work appear to cause more bias than would be acceptable. A more general discussion of issues in evidence synthesis methods has been addressed elsewhere [37] and is outside the scope of the present work.

The results of this assessment of validity have implications for systematic reviewers and meta-analysts considering or using these re-expression methods. The bias due to re-expression with the three methods evaluated was affected by the skewness of the exposure variable, and, for some estimators, the median exposure or the type of transformation used. Even with adjustment for the bias these re-expression methods, the estimates, on average, were too biased, and too variable in their degree of bias, to justify their use to support meta-analyses used in risk assessment. Future studies comparing different methods of synthesis across evidence streams might clarify the settings in which distortion of results might be most likely to occur, quantify the magnitude of distortion, and explicate their strengths and weaknesses.

## Supplementary Information

**Additional file 1.**

**Additional file 2.**

## Data Availability

The data used in this manuscript are freely available from their respective publications or upon request from the original authors. R scripts/functions and data files for applying each of these three re-expression methods are available in the supplemental materials (Supplemental_Code.zip).

## Abbreviations

*β*_Estimand_: *β* Coefficient from fitting *y* = *β*_Estimand_·*x* + *e* with an ordinary least squares (OLS) model
*β*_RB_: Estimator evaluated using the Rodriguez-Barranco method
*β*_Dz_: Estimator evaluated using the Dzierlenga method
*β*_Alt_: Estimator evaluated using the Alternative method
ADEMP: Aims, Data generating mechanism, Estimand, Methods, and Performance measures (Simulation study methodology)
DGM: Data generating mechanism
PFOA: Perfluorooctanoic acid
