# Supplementary material for: Re-expressing coefficients from regression models for inclusion in a meta-analysis
Source: BMC Med Res Methodol. 2024 Jan 8;24:6. doi: 10.1186/s12874-023-02132-y (PMC10773134; doi:10.1186/s12874-023-02132-y)
Supplement: Supplementary file 1 — Additional file 1. [file 12874_2023_2132_MOESM1_ESM.docx]

Supplementary Materials for: Re-expressing coefficients from regression models for inclusion in a meta-analysis

Matthew W. Linakis,^a,*^ Cynthia Van Landingham,^b^ Alessandro Gasparini^c^, Matthew P. Longnecker^a^

^a^ Ramboll U.S. Consulting, Raleigh NC 27612 U.S.A.

^b^ Ramboll U.S. Consulting, Monroe LA 71201 U.S.A.

^c^ Red Door Analytics AB, Stockholm, Sweden

*Corresponding Author:

Matthew W. Linakis

Ramboll, 3214 Charles B Root Wynd #130, Raleigh, NC 27612 U.S.A.

mlinakis@ramboll.com

Phone: (919) 987-3063

Contents

[Supplemental Methods Section 1: Primary data examples 3](#_Toc150417713)

[Selection of real data examples 3](#_Toc150417714)

[How we identified published analyses based on data that were publicly available 3](#_Toc150417715)

[How we identified reports in which coefficients from regression analyses with and without log transformation of the exposure had been presented 4](#_Toc150417716)

[Comment 4](#_Toc150417717)

[Supplemental Methods Section 2: Secondary data examples 5](#_Toc150417718)

[Selection of the second set of real data examples 5](#_Toc150417719)

[Comment 5](#_Toc150417720)

[Figure S1 7](#_Toc150417721)

[Table S1 8](#_Toc150417722)

[Table S2 9](#_Toc150417723)

[Table S3 11](#_Toc150417724)

[Table S4 12](#_Toc150417725)

[Table S5 14](#_Toc150417726)

[Table S6 16](#_Toc150417727)

[Table S7 17](#_Toc150417728)

[Table S8 18](#_Toc150417729)

[Table S9 19](#_Toc150417730)

[Table S10 20](#_Toc150417731)

[Table S11 21](#_Toc150417732)

# Supplemental Methods Section 1: Primary data examples

## Selection of real data examples

We selected real data examples by two approaches. The first approach was to identify environmental epidemiology reports based on data that were publicly available. The second approach was to select reports in which coefficients from regression analyses with and without log transformation of the exposure had been presented. Each method is described in a section below.

## How we identified published analyses based on data that were publicly available

Data from the U.S. National Health and Nutrition Examination Survey (NHANES) are publicly available ([NHANES Questionnaires, Datasets, and Related Documentation (cdc.gov)](https://wwwn.cdc.gov/nchs/nhanes/)). To create a list of potentially useful reports, we conducted a PubMed search using the search algorithm “national health and nutrition examination survey” and environmental not “korea national” not “korean national”. The date of publication had to be 1-1-1960 to 5-31-2021. This generated a list of 1,596 reports.

The list of 1,596 reports was evaluated by MPL, and the following initial screening criteria were applied to identify results that were potentially of interest: date appeared in print had to be by 5-31-21 (rather than electronically available), the outcome had to be a recognized disease or clinically-important biomarker, the exposure had to be a biomarker of an environmental contaminant, the analysis had to present results for associations with a single contaminant in relation to the outcome (rather than a mixture), the concentration of the contaminant had to have been examined as a continuous variable, the results had to be presented as a ratio-type measure of association (e.g., relative risk, odds ratio) or a beta coefficient, and at least one result had to be statistically significant at the p < 0.05 level (two sided).

Among the reports that were potentially eligible, the selection for potential inclusion was arbitrary and represented a subset of reports that could have been further examined. For example, Tsoi et al. (2021) met the eligibility criteria but was not further evaluated because another study with lead as an exposure was selected. We aimed to include studies of a variety of outcomes and environmental contaminants.

In the final screening of results for inclusion, we required that we were able to replicate the selected result that was statistically significant, meaning the same association was statistically significant when we analyzed the data. This was a labor-intensive exercise and precluded a formal definition of the sampling frame. Thus, we began with an arbitrary subset of articles meeting the initial screening and selection criteria described above, and then identified among those 9 results that could be replicated and we deemed sufficient for obtaining a general sense of the range of parameters of lognormal distributions encountered in practice as well as a variety of examples to which our set of estimators could be applied. While it is interesting that some published results could not be replicated (as we define it), that issue was not our focus and we do not address it further.

Unlike the other studies that were included, Xu, 2020 provided results for one set of outcomes that were represented as a dichotomous variable and for a second set of different outcomes that were represented as a continuous variable. In other words, their publication was like two reports rolled into one, and the analyses seemed different enough to serve as independent examples. Note that in the new (second) set of results selected from this study for use as a real data example (see Supplemental Methods 2), the exposures were different in the two results selected from this study.

## How we identified reports in which coefficients from regression analyses with and without log transformation of the exposure had been presented

In general, few authors report the paired set of coefficients that we sought. However, because of our familiarity with reports in the epidemiology of per- and polyfluoroalkyl substances (PFAS), we were aware of six studies with such results.

## Comment

Due to the informal nature of the process for selecting real data examples of the type needed, it is likely that the parameter space examined in the simulations (Table 1) did not include all configurations of parameters that might be encountered in practice. Furthermore, the results of using the re-expression methods on the real data examples cannot be generalized to all environmental epidemiology studies with exposure measured with a biomarker. Nonetheless, examination of results for the real datasets provided insights into the behavior of the re-expression methods not provided by the simulations alone.

# Supplemental Methods Section 2: Secondary data examples

## Selection of the second set of real data examples

To evaluate whether the choice of simulation parameters based on the first set of real data examples would have been the same if a different sent of real data examples had been used, and to see if our conclusions based on use of the re-expression estimators on the first set of real data examples were robust, we selected a second set of real data examples. The second set of real data examples was based on the same reports used for the first set of real data examples. However, we used a different method of choosing results for inclusion in the second set of real data examples. We enumerated all possible adjusted main results (no subgroup results) that could have been selected, and then selected a result at random. This was done for all reports used for second set of real data examples except those in which only two results were eligible for inclusion (Chen et al., 2012; Washino et al., 2009). In the cases where only two results were eligible for inclusion, for the second set of real data examples we included the result that had not been included in the first set of real data examples. For those reports where results were selected at random for the second set of real data examples, by chance, none of the results selected for the first set of real data examples were included. Thus, the results in the first and second sets of real data examples were different.

## Comment

As described in Supplement Section __, our method of selecting reports for use as the source of the real data examples was laborious and as a practical matter precluded a well-delineated sampling frame. Thus, the second set of real data examples has, in this respect, the same limitations as described for the first. The second set of real data examples, however, was selected without considering the statistical significance of the results, and thus gave a better sense of how the estimators worked in the setting of smaller effect sizes.


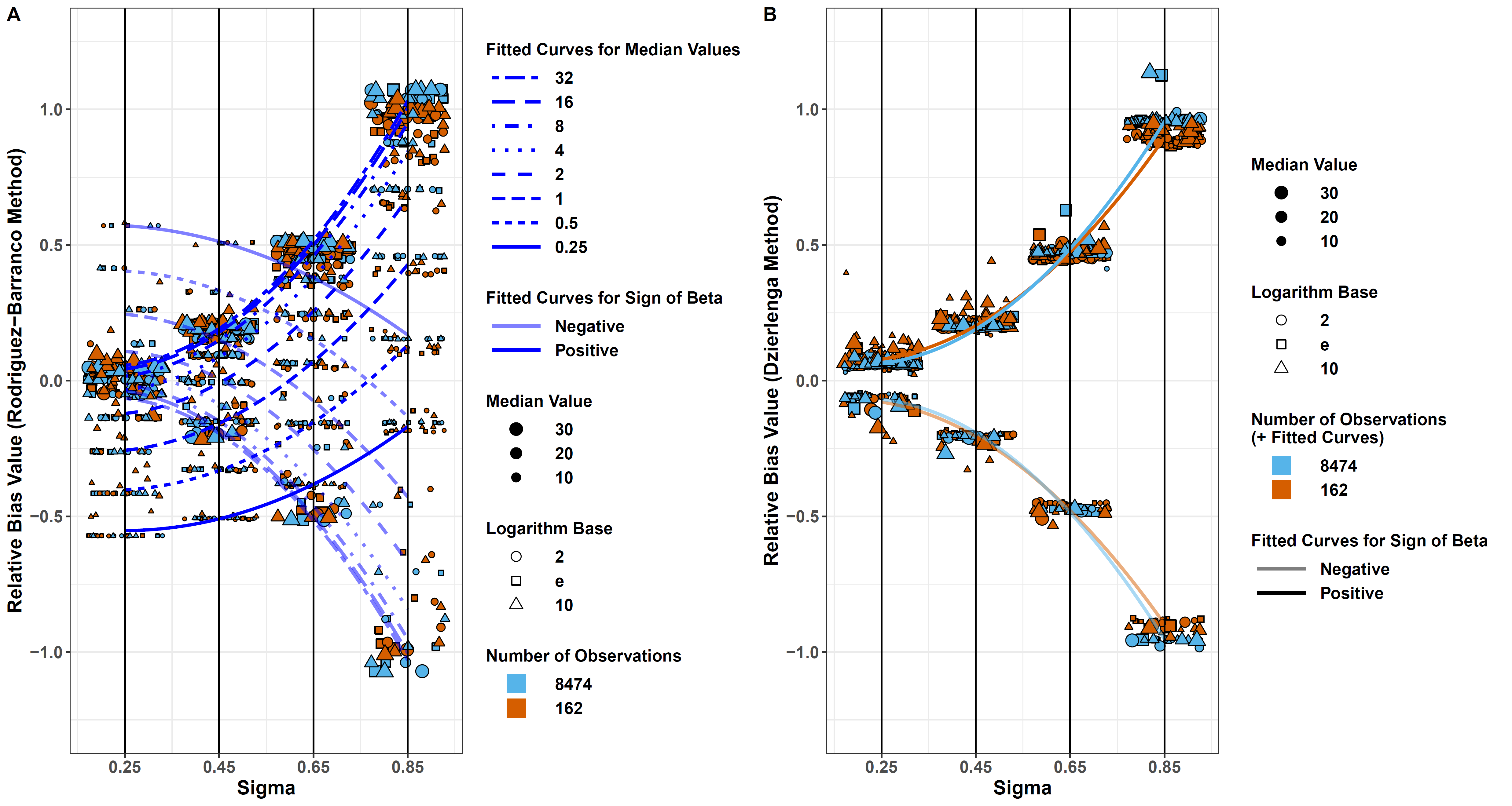


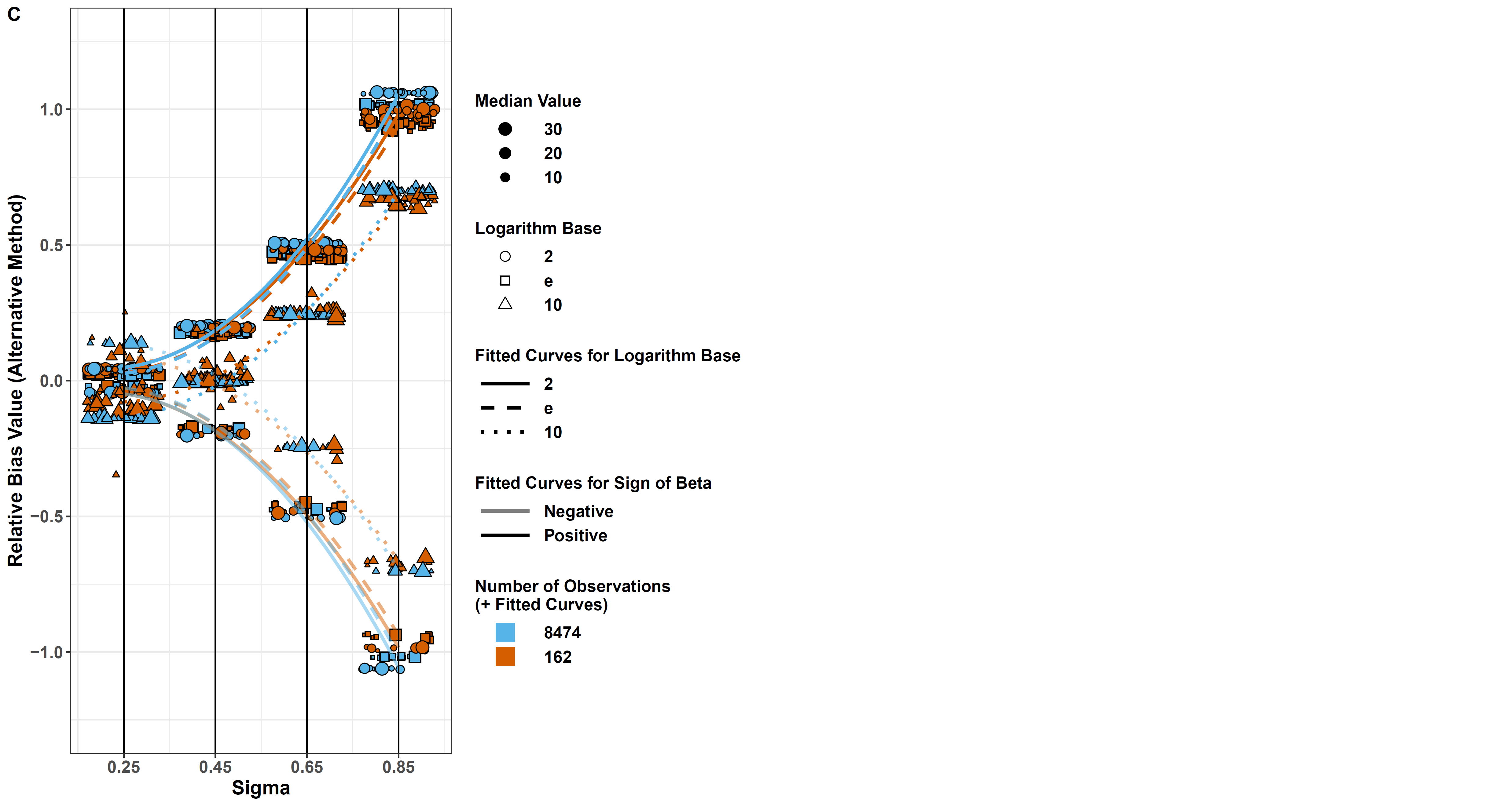


Figure S1: Plots of relative bias as a function of skewness (σ) in the exposure x, by type of estimator, including the scenario where β_DGM_ < 0. Individual points represent the average result (n_sim_ = 2000) for each simulation scenario. A total of 890 of the possible 1,920,000 observations (960 scenarios x 2000 simulations) were not used in the calculation of the average results because *β*_estimand_ was < 0.0001 (essentially zero). Lines represent quadratic fits to the data for a specified prediction equation and set of values of independent variables (see text). Note that data have been artificially spread along the x-axis for visualization purposes, all actual x-values are the closest black vertical line (0.25, 0.45, 0.65, or 0.85). Figures A-C show points for 960 simulations. A) Rodriguez-Barranco estimator, B) Dzierlenga estimator, and C) Alternative estimator.

Table S1: Parameter values for the original and second real data results used in our analyses

| Study | Parameter | | | | | | | | | |
| --- | --- | --- | --- | --- | --- | --- | --- | --- | --- | --- |
|  | Original  n_obs­_ | Second  n_obs­_ | Original  *β*_DGM_^a^ | Second  *β*_DGM_^a^ | Original  *σ* | Second  *σ* | Original  median | Second  median | Original  logbase | Second  logbase |
| Bulka 2021 | 8778 | 8778 | 0.015 | 0.0245 | 0.75 | 0.77 | 2.77 | 5.19 | 2 | 2 |
| Lee 2020 | 124 | 124 | 1.444 | 0.967 | 0.83 | 0.54 | 0.24 | 0.53 | 2 | 2 |
| Odebeatu 2019 | 7765 | 7765 | 0.000686 | 0.0502 | 1.26 | 1.07 | 12.3 | 0.142 | 10 | 10 |
| Xu 2020a | 1947 | 1947 | 0.513 | 0.028 | 0.81 | 0.39 | 0.521 | 2.09 | 2 | 2 |
| Xu 2020b | 1947 | 1947 | 29.3 | 0.0348 | 0.81 | 0.84 | 0.521 | 0.524 | 2 | 2 |
| Stein 2016 | 1191 | 1191 | -0.0039 | -0.00413 | 0.52 | 0.52 | 22.2 | 22.2 | 2 | 2 |
| Pilkerton 2018 | 621 | 621 | -0.0049 | 0.000161 | 0.55 | 0.57 | 4.3 | 21.8 | 2 | 2 |
| Cheang 2021 | 2899 | 2899 | 0.192 | 0.0333 | 0.47 | 0.51 | 38.7 | 83.5 | 2 | 2 |
| Abraham 2020 | 101 | 101 | -0.0636 | -0.00797 | 0.78 | 0.78 | 14.3 | 14.3 | 2 | 2 |
| Apelberg 2007 | 293 | 293 | -12.9 | -0.043 | 0.62 | 0.41 | 5 | 1.6 | e | e |
| Washino 2009 | 428 | 428 | -10.94 | -2.07 | 0.53 | 0.6 | 5.2 | 1.3 | 10 | 10 |
| Hamm 2010 | 252 | 252 | 1.5 | 3.9 | 0.47 | 1.11 | 7.8 | 1.1 | e | e |
| Chen 2012 | 429 | 429 | -11.3 | -11.15 | 0.61 | 0.98 | 5.94 | 1.89 | e | e |
| Darrow 2013 | 1630 | 1630 | -2.3 | 0.0322 | 0.54 | 0.54 | 13.9 | 13.9 | e | e |
| Steenland 2009 | 46294 | 46294 | 0.00105 | 6.00E-05 | 0.57 | 1.51 | 20.2 | 26.6 | e | e |
| Min | 101 | 101 | -12.9 | -11.15 | 0.47 | 0.39 | 0.24 | 0.142 | 53.3%^a^ | 53.3%^a^ |
| Q1 | 360.5 | 360.5 | 0.000686 | 0.0245 | 0.535 | 0.53 | 3.535 | 1.2 | 33.3%^b^ | 33.3%^b^ |
| Median | 1191 | 1191 | 29.3 | 3.9 | 0.61 | 0.6 | 5.94 | 2.09 | 13.3%^c^ | 13.3%^c^ |
| Q3 | 2423 | 2423 | 0.015 | 0.0245 | 0.795 | 0.91 | 14.1 | 18.05 | - | - |
| Max | 46294 | 46294 | 1.444 | 0.967 | 1.26 | 1.51 | 38.7 | 83.5 | - | - |

^a^% of studies with log base of 2

^b^% of studies with log base of e

^c^% of studies with log base of 10

Table S2: Coefficients from ordinary least squares models of relative bias, by re-expression method. Each method was described by a quadratic fit (ax^2^+bx+c) with additional predictor variables as described in the table.

| Method | Parameter | Coefficient | Standard Error | *p*-value | RMSE | Adjusted R^2^ |
| --- | --- | --- | --- | --- | --- | --- |
| Rodriguez-Barranco | Intercept (c) | -0.13 | 0.0596 | 0.026 | 0.230 | 0.706 |
| Method (*β*_RB_) | Linear sigma term (b) | -0.95 | 0.2342 | <0.001 |  |  |
|  | Quadratic sigma term (a) | 1.87 | 0.2085 | <0.001 |  |  |
|  | Median | 0.01 | 0.0021 | 0.001 |  |  |
|  | Interaction term between median and sigma (µ:σ) | 0.02 | 0.0036 | <0.001 |  |  |
| Dzierlenga Method | Intercept (c) | 0.18 | 0.0076 | <0.001 | 0.028 | 0.993 |
| (*β*_Dz_) | Linear σ term (b) | -0.84 | 0.0283 | <0.001 |  |  |
|  | Quadratic σ term (a) | 2.00 | 0.0251 | <0.001 |  |  |
|  | Number of observations in simulated study (nobs) | -7.02E-06 | 6.40E-07 | <0.001 |  |  |
|  | logbase (logbase = 10) | 0.0167 | 0.0025 | <0.001 |  |  |
|  | logbase (logbase = 2) | -0.0026 | 0.0025 | 0.29 |  |  |
|  | β_DGM_ | 0.0002 | 8.39E-05 | 0.004 |  |  |
|  | Median | 0.0005 | 9.68E-05 | <0.001 |  |  |
|  | Interaction term between nobs and sigma (nobs:σ) | 1.51E-05 | 1.08E-06 | <0.001 |  |  |
| Alternative Method | Intercept (c) | 0.13 | 0.0071 | <0.001 | 0.024 | 0.996 |
| (*β*_Alt_) | Linear σ term (b) | -0.90 | 0.0247 | <0.001 |  |  |
|  | Quadratic σ term (a) | 2.20 | 0.0214 | <0.001 |  |  |
|  | logbase (logbase = 10) | -0.05 | 0.0056 | <0.001 |  |  |
|  | logbase (logbase = 2) | 9.41E-03 | 0.0056 | 0.091 |  |  |
|  | number of observations in simulated study (nobs) | -7.21E-06 | 5.46E-07 | <0.001 |  |  |
|  | Interaction term between σ and logbase = 10 (logbase10: σ) | -0.27 | 0.0094 | <0.001 |  |  |
|  | Interaction term between σ and logbase = 2 (logbase2: σ) | 0.04 | 0.0094 | <0.001 |  |  |
|  | Interaction term between σ and nobs (σ:nobs) | 1.56E-05 | 9.20E-07 | <0.001 |  |  |

Table S3: Comparison of model fits (R^2^) when using each simulation scenario with β_DGM_ > 0 (n = 768) or using each observation with β_DGM_ > 0 (n = 768 * 2000)

| r^2^ for: | β_RB_ | β_Dz_ | β_Alt_ |
| --- | --- | --- | --- |
| n = 768 | 0.706 | 0.992 | 0.996 |
| n = 768 * 2000 | 0.709 | 0.992 | 0.996 |

Table S4. Published analyses (original set) of an outcome in relation to a biomarker-based measure of environmental exposure, with raw data available

| 1^st^ author, year | Outcome | Type of outcome^a^ | Exposure^b^ | Original unit of exposure | Result presented by original authors^c^ | Our result^c^  (re-analysis of raw data) |
| --- | --- | --- | --- | --- | --- | --- |
| Bulka, 2021 | Herpes Simplex Virus 2 | D | PFOA | Log_2_ | 1.11  (1.05, 1.17) | 1.11  (1.05, 1.17) |
| Lee, 2020 | Infertility | D | Cadmium | Log_2_ | 1.8  (1.1, 3.1) | 1.8  (1.1, 3.1) |
| Odebeatu, 2019 | Asthma | D | Mono-benzyl phthalate (urine) | Log_10_ | 1.50  (1.09, 2.08) | 1.50  (1.08, 2.08) |
| Xu, 2020 | CVD^d^ | D | Isopentanaldehyde | ng/ml | P<0.001 | 1.67  (1.17, 2.25) |
| Xu, 2020 | Triglycerides (mg/dl) | C | Isopentanaldehyde | ng/ml | 25.0  (4.8, 45.1) | 29.3  (14.1, 44.6) |
| Stein, 2016 | Mumps IgG^e^ (%∆) | C | PFOS | Log_2_ | -7.4  (-12.8, -1.7) | -10.3  (-19.3, -0.024) |
| Pilkerton, 2018 | Rubella IgG^e^ (%∆) | C | PFOA | Log_2_^f^ | -8.9  (-16.9, -0.2)^f^ | N.A. |
| Cheang, 2021 | Triglycerides (mg/dl) | C | Glycidamide^g^ | Log_2_ | 11.4  (5.1, 17.7) | 9.65  (1.62, 17.7) |
| Abraham, 2020 | Ln(Hib^h^ IgG^e^) | C | PFOA | Log_2_ | -0.3887^i^  (-0.6957, -0.0817) | N.A. |

^a^ C = continuous, D = dichotomous

^b^ Measured in serum unless noted otherwise

^c^ Results shown are from regression analyses. For dichotomous outcomes, these are odds ratios (and 95% confidence intervals). For continuous outcome, the results are either regression coefficients, or regression results re-expressed as percent difference (%∆) in outcome per unit exposure

^d^ CVD, cardiovascular disease

^e^ IgG, immunoglobulin G

^f^ The original units were quartiles; we re-analyzed the data to get the original result shown, in percent change in Rubella antibody per log_2_ increase in PFOA. See Crawford et al. (reference), Supplementary Material, Section X for an account of the re-analysis.

^g^ As reflected by concentration of hemoglobin adduct of glycidamide (HbGA)

^h^ Hib, Hemophilus Influenza

^i^ Our analysis of the Abraham data. See Crawford et al. (submitted, 2021), Supplementary Material, Section S7 for an account of the re-analysis.

Table S5. Second set of published analyses of an outcome in relation to a biomarker-based measure of environmental exposure, with raw data available

| 1^st^ author, year | Outcome | Type of outcome^a^ | Exposure^b^ | Original unit of exposure | Result presented by original authors^c^ | Our result^c^  (re-analysis of raw data) |
| --- | --- | --- | --- | --- | --- | --- |
| Bulka, 2021 | Toxocara | D | PFOS | Log_2_ | 1.57^d^  (1.26, 1.96) | 1.68  (1.26, 2.26) |
| Lee, 2020 | Infertility | D | Lead | Log_2_ | 2.60  (1.05, 6.41) | 2.63  (1.08, 6.44) |
| Odebeatu, 2019 | Asthma | D | ∑DEHP (urine) | Log_10_ | 1.17^e^  (0.78, 1.74) | 1.11  (0.836, 1.46) |
| Xu, 2020 | CVD^f^ | D | Propanaldehyde | ng/ml | P=0.63^g^ | 1.03  (0.856, 1.236) |
| Xu, 2020 | Monocytes  (10^9^/L) | C | Isopentanaldehyde | ng/ml | 0.03  (-0.01, 0.06) | 0.035  (0.002, 0.068) |
| Stein, 2016 | Mumps IgG^h^ (%∆) | C | PFHxS | Log_2_ | -2.6  (-6.7, 1.7) | -2.6  (-6.5, 1.5) |
| Pilkerton, 2018 | Rubella IgG^h^ (%∆) | C | PFOS | Log_2_^i^ | -4.16  (-13.26, 5.90)^i^ | N.A. |
| Cheang, 2021 | LDL^j^  (mg/dl) | C | HbAA + HbGA^k^ | Log_2_ | 1.40  (-1.10, 3.90) | 3.31  (-0.096, 6.72) |
| Abraham, 2020 | Ln(Diphtheria) IgG^h^) | C | PFOA | ng/ml | -0.00797^l^  (-0.01336, -0.00258) | N.A. |

^a^ C = continuous, D = dichotomous

(cont.)

(Footnotes to Table S2b, cont.)

^b^ Measured in serum unless noted otherwise

^c^ Results shown are from regression analyses. For dichotomous outcomes, these are odds ratios (and 95% confidence intervals). For continuous outcome, the results are either regression coefficients, or regression results re-expressed as percent difference (%∆) in outcome per unit exposure

^d^ Subjects aged 20-49 y

^e^ Adults

^f^ CVD, cardiovascular disease

^g^ P value for result presented by original authors was based on a quartile analysis

^h^ IgG, immunoglobulin G

^i^ The original units were quartiles; we re-analyzed the data to get the original result shown, in percent change in Rubella antibody per log_2_ increase in PFOS. See Crawford et al. (2023), Supplementary Material, Section 6 for an account of the re-analysis.

^j^ LDL, low density lipoprotein

^k^ HbAA + HbGA, hemoglobin adducts of acrylamide plus hemoglobin adducts of glycidamide

^l^ Our analysis of the Abraham data. See Crawford et al. (2023), Supplementary Material, Section S4 for an account of the re-analysis.

Table S6. Additional details about the 15 example studies

| Study, Year | Specific Finding  (Location, Outcome) | Exposure Distribution | | | | |
| --- | --- | --- | --- | --- | --- | --- |
|  |  | Median | 1^st^  Quartile | 3^rd^  Quartile | σ (lognormal distribution) | Mean |
| Abraham 2020 | Our analysis, Hib IgG | 14.3^a^ | 6.70 | 19.3 | 0.78 | 16.8 |
| Apelberg, 2007 | Table 3 (Fully Adjusted), Birth Weight (g) | 5.00^b^ | 3.40 | 7.90 | 0.62 | 5.43^f^ |
| Bulka, 2021 | Table 3 (20-49 y), HSV 2 | 2.77^a^ | 1.67 | 4.6 | 0.75 | 3.0^f^ |
| Cheang, 2021 | Table 3, Triglycerides (mg/dL) | 38.7^a^ | 29.4 | 55.2 | 0.47 | 41.1^f^ |
| Chen, 2012 | Table 3 (Adjusted), Birth Weight (g) | 5.94^e^ | 3.94 | 8.94 | 0.61 | 6.27^f^ |
| Darrow, 2013 | Table 6 (Adjusted All Births, Per in unit increase), Birth Weight (g) | 13.9^b^ | 9.5 | 19.7 | 0.54 | 14.4^f^ |
| Hamm, 2010 | Table 5 (Hamm, PFOS), Birth Weight (g) | 7.80^d^ | 5.70 | 10.7 | 0.47 | 8.07^f^ |
| Lee, 2020 | Table 2 (Model 2), Infertility | 0.240^a^ | 0.14 | 0.43 | 0.83 | 0.270^f^ |
| Odebeatu, 2019 | Figure 1a (MBzP), Asthma | 12.3^b^ | 5.00 | 27.3 | 1.26 | 14.9^f^ |
| Pilkerton, 2018 | Table 4, Rubella (%Δ) | 4.30^a^ | 3.00 | 6.3 | 0.55 | 6.00 |
| Steenland 2009 | Table 4, Total Cholesterol | 20.2^c^ | 13.6 | 29.3 | 0.57 | 22.4 |
| Stein, 2016c | Table 2, Mumps (%Δ) | 22.2^a^ | 15.35 | 30.8 | 0.52 | 22.8^f^ |
| Xu, 2020 | Table 2 (Model 1), CVD | 0.521^a^ | 0.346 | 1.03 | 0.81 | 0.632^f^ |
| Xu, 2020 | Table 4 (Model 2), Triglycerides (mg/dL) | 0.521^a^ | 0.346 | 1.03 | 0.81 | 0.632^f^ |
| Washino, 2009 | Table 5 (Fully Adjusted), Birth Weight (g) | 5.20^b^ | 3.40 | 7.00 | 0.53 | 5.20^f^ |

^a^Median and IQR calculated from raw data

^b^Median and IQR pulled from publication

^c^Median from publication and IQR adjusted from Steenland 2010 by subtracting 0.6 (difference between medians in studies)

^d^Median from publication and IQR adjusted from Lind 2017, by subtracting 0.3 (difference between medians in studies)

^e^Median from publication and IQR adjusted from Chen 2017 by adding 0.24 (difference between medians in studies)

^f^Mean estimated from median and IQR using the formula from Wan 2014: (Q1+Median+Q3)/3

Table S7. Additional details about the second set of 15 example studies

| Study, Year | Specific Finding  (Location, Outcome, Exposure) | Exposure Distribution | | | | |
| --- | --- | --- | --- | --- | --- | --- |
|  |  | Median | 1^st^  Quartile | 3^rd^  Quartile | σ (lognormal distribution) | Mean |
| Abraham 2020 | Our analysis, Diphtheria IgG, PFOA | 14.3^a^ | 6.70 | 19.3 | 0.78 | 16.8 |
| Apelberg, 2007 | Table 3 (Fully Adjusted), Ponderal Index (g cm^3^ x 100), PFOA | 1.6^b^ | 1.2 | 2.1 | 0.41 | 1.63^c^ |
| Bulka, 2021 | Table 3 (20-49 y), Toxocara, PFOS | 5.19^a^ | 3.00 | 8.50 | 0.77 | 6.92 |
| Cheang, 2021 | Table 3, LDL (mg/dL), HbAA+HbGA | 83.5^a^ | 64.1 | 127.7 | 0.51 | 119.9 |
| Chen, 2012 | Table 3 (Adjusted), Birth Weight (g), PFOA | 1.89^d^ | 0.99 | 3.71 | 0.98 | 2.20^c^ |
| Darrow, 2013 | Table 4 (Adjusted First Prospective Births, Per ln unit increase), Pregnancy Induced Hypertension, PFOS | 13.9^b^ | 9.5 | 19.7 | 0.54 | 15.6^c^ |
| Hamm, 2010 | P 592, left column, Birth Weight (g), PFHxS | 1.10^e^ | 0.51 | 2.28 | 1.11 | 1.3^c^ |
| Lee, 2020 | Table 2 (Model 2), Infertility, Lead | 0.53^a^ | 0.350 | 0.725 | 0.54 | 0.687 |
| Odebeatu, 2019 | Figure 3b, Asthma, ∑DEHP (µmol/L) | 0.142^a^ | 0.069 | 0.292 | 1.07 | 0.390 |
| Pilkerton, 2018 | Table 4, Rubella (%Δ), PFOS | 21.8^a^ | 14.2 | 30.7 | 0.57 | 25.0 |
| Steenland 2009 | Table 4, HDL, PFOA | 26.6^f^ | 9.2 | 70.9 | 1.51 | 80.3 |
| Stein, 2016c | Table 2 (All), Mumps (%Δ), PFHxS | 22.2^a^ | 15.35 | 30.8 | 0.52 | 22.8^c^ |
| Xu, 2020 | Table 2 (Model 2), CVD, Propanaldehyde | 2.09^a^ | 1.57 | 2.67 | 0.6 | 2.21 |
| Xu, 2020 | Table 4 (Model 2), Monocytes, Isopentanaldehyde | 0.524 ^a^ | 0.343 | 1.06 | 0.39 | 0.775 |
| Washino, 2009 | Table 5 (Fully Adjusted), Birth Weight (g), PFOA (From Verner et al. 2015) | 1.3^b^ | 0.8 | 1.8 | 0.84 | 1.4 |

^a^Median and IQR calculated from raw data

^b^Median and IQR from publication

^c^Mean estimated from median and IQR using the formula from Wan 2014: (Q1+Median+Q3)/3.

^d^Median and IQR from Chen 2017 used to estimate a lognormal distribution with quartiles shown.

^e^Assumed median = geometric mean from publication; used geometric standard deviation to estimate quartiles.

^f^Median and mean from publication used to estimate a lognormal distribution with quartiles shown.

Table S8. Published analyses of an outcome in relation to a biomarker-based measure of environmental exposure, where the original authors presented results using exposure with and without a log-transformation

| 1^st^ author, year | Outcome | Type of outcome | Exposure | Logarithmic (β in units/log(ng/ml))  Result reported by original authors^a^ | Untransformed (β in units/(ng/ml))  Result reported by original authors^b^ |
| --- | --- | --- | --- | --- | --- |
| Apelberg, 2007 | Birth weight (g) | C | PFOS | -69  (-149, 10) | -12.9  (-27.8, 2) |
| Chen, 2012 | Birth weight (g) | C | PFOS | -110.2  (-176.0, -44.5) | -11.30  (-17.40, -5.20) |
| Darrow, 2013 | Birth weight (g) | C | PFOS | -29  (-66, 7) | -2.3  (-4.8, 0.3) |
|  |  |  |  |  |  |
| Hamm, 2010 | Birth weight (g) | C | PFOS | 31.3  (-43.3, 105.9) | 1.5  (-7.6, 10.6) |
| Steenland, 2009 | Ln(Serum cholesterol (mg/dl)) | C | PFOS | 0.0266  (0.0239, 0.0293) | 0.00105  (0.0009, 0.0012) |
| Washino, 2009 | Birth weight (g) | C | PFOS | -148.8  (-297.0, -0.5) | -10.94  (-22.9, 1.10) |

^a^ Values shown are for log_e_ transformations, except for Washino et al, for which a log_10_ transformation was used. Values in parentheses are 95% confidence intervals.

^b^ Values for Washino et al. (2009) and Chen et al. (2012) in this column were presented in Verner et al. (2015).

Table S9. Second set of published analyses of an outcome in relation to a biomarker-based measure of environmental exposure, where the original authors presented results using exposure with and without a log-transformation

| 1^st^ author, year | Outcome | Type of outcome | Exposure | Logarithmic (β in units/log(ng/ml))  Result reported by original authors^a^ | Untransformed (β in units/(ng/ml))  Result reported by original authors^b^ |
| --- | --- | --- | --- | --- | --- |
| Apelberg, 2007 | Ponderal index g/cm^3^ x 100) | C | PFOA | -0.070  (-0.138, -0.001) | -0.043  (-0.088, -0.001) |
| Chen, 2012 | Birth weight (g) | C | PFOA | -19.2  (-63.5, 25.1) | -11.15  (-26.01, 3.71) |
| Darrow, 2013 | Pregnancy induced hypertension | D | PFOS | 0.703  (0.104, 1.297) | 0.0322  (0.005, 0.059) |
| Hamm, 2010 | Birth weight (g) | C | PFHxS | 21.9  (-23.4, 67.2) | 3.9  (-8.2, 16.0) |
| Steenland, 2009 | Ln(HDL cholesterol (mg/dl)) | C | PFOA | 0.0276  (-0.00447, 0.00635) | 0.00006  (-0.00004, 0.000158) |
| Washino, 2009 | Birth weight (g) | C | PFOA | -75.1  (-191.8, 41.6) | -2.07  (-41.80, 37.65) |

^a^ Values shown are for log_e_ transformations, except for Washino et al, for which a log_10_ transformation was used. Values in parentheses are 95% confidence intervals.

^b^ Values for Washino et al. (2009) and Chen et al. (2012) in this column were presented in Verner et al. (2015).

Table S10. Comparison of re-expression outcome according to whether influential observations were included in the data

| 1^st^ author, year | Proportional difference  with influential observations | Proportional difference  with no influential observations |
| --- | --- | --- |
| Odebeatu 2019 | 16.82 | 19.58 |
| Pilkerton 2018 | 5.17 | 2.18 |
| Cheang 2021 | 0.84 | 1.11 |
| Xu 2020a | 0.88 | 1.38 |

Table S11: Comparison of fitted and re-expressed *β* coefficients and relative bias in *β* for three methods of re-expression,

second set of real data examples

| First author,  year | *β*_Estimand_ from analysis of  raw data | *β*_RB_^a^ | Relative Bias^b^ | *β*_Dz_ | Relative Bias^b^ | *β*_Alt_^c^ | Relative Bias^b^ |
| --- | --- | --- | --- | --- | --- | --- | --- |
| Abraham, 2020 | -0.00797 log_e_/ng·ml^-1^ | -0.00376 | -0.53 | -0.00427 | -0.46 | -0.00446 | -0.44 |
| Apelberg, 2007 | -0.0433 g/ng·ml^-1^ | -0.0335 | -0.22 | -0.043 | 0 | -0.042 | -0.02 |
| Bulka, 2021 | 0.0245 log_e_(OR)/ng·ml^-1^ | 0.101 | 3.12 | 0.136 | 4.54 | 0.141 | 4.77 |
| Cheang, 2021 | 0.0333 mg·dl/pmol·g^-1^ | 0.0397 | 0.19 | 0.0556 | 0.67 | 0.0561 | 0.68 |
| Chen, 2012 | -11.15 g/ng·ml^-1^ | -7.19 | -0.35 | -9.23 | -0.17 | -9.75 | -0.13 |
| Darrow, 2013 | 0.0322 log_e_(OR)/ng·ml^-1^ | 0.0437 | 0.36 | 0.0491 | 0.52 | 0.0485 | 0.51 |
| Hamm, 2010 | 3.9 g/ng·ml^-1^ | 12.5 | 2.2 | 17.6 | 3.51 | 19.1 | 3.9 |
| Lee, 2020 | 0.967 log_e_(OR)/μg·dl^-1^ | 1.25 | 0.3 | 2.56 | 1.64 | 2.58 | 1.67 |
| Odebeatu, 2019 | 0.0502 log_e_(OR)/µmol·L^-1^ | 0.0576 | 0.15 | 0.285 | 4.67 | 0.258 | 4.14 |
| Pilkerton, 2018 | 0.000161 log_e_/ng·ml^-1^ | -0.0024 | -15.9 | -0.00272 | -17.9 | -0.00276 | -18.1 |
| Steenland, 2009 | 0.00006 log_e_/ ng·ml^-1^ | 3.42E-05 | -0.43 | 8.30E-05 | 0.38 | 9.96E-05 | 0.66 |
| Stein, 2016 | -0.00413 log_e_/ng·ml^-1^ | -0.00163 | -0.6 | -0.00167 | -0.6 | -0.00168 | -0.59 |
| Washino, 2009 | -2.07 g/ng·ml^-1^ | -17.6 | 7.49 | -24.2 | 10.7 | -20.3 | 8.81 |
| Xu, 2020a | 0.0280 log_e_(OR)/ng·ml^-1^ | 0.0159 | -0.43 | 0.0201 | -0.28 | 0.02 | -0.29 |
| Xu, 2020b | 0.0348 mg·dl/ng·ml^-1^ | 0.034 | -0.02 | 0.0729 | 1.09 | 0.0766 | 1.2 |
| Median |  |  | -0.02 |  | 0.52 |  | 0.66 |
| First quartile |  |  | -0.43 |  | -0.225 |  | -0.21 |
| Third quartile |  |  | 0.33 |  | 2.575 |  | 2.785 |
| Minimum |  |  | -15.9 |  | -17.9 |  | -18.1 |
| Maximum |  |  | 7.49 |  | 10.7 |  | 8.81 |

^a^ Using the notation of Rodriguez-Barranco et al., k = base of log transformation used; c = 1.

^b^ Proportional difference between β in column to the left compared with the one from the analysis of raw data, calculated using the same method as in previous table ((beta in column to left – beta from analysis of raw data)/beta from analysis of raw data). Note that the β in column to the left was calculated using β with different units in denominator than for the raw data analysis shown in the table.

^c^ Let the log unit increment I in untransformed units = *b*^(l^*^og^_b_*^(median) + 0.5)^ - *b*^(log^*_b_*^(median) – 0.5)^, where *b* = 2, e, or 10, depending on the base. For observed β_o_ with units of ∆y/∆log*_b_*(x), to get re-expressed β_r_ with units ∆y/∆x, calculate β_r_ = β_o_/I. If the units of β_o_ are ∆y/∆x, to get β_r_ with units ∆y/∆log*_b_*(x), calculate β_r_ = β_o_· I.
