# Supplementary material for: Re-expressing coefficients from regression models for inclusion in a meta-analysis
Source: BMC Med Res Methodol. 2024 Jan 8;24:6. doi: 10.1186/s12874-023-02132-y (PMC10773134; doi:10.1186/s12874-023-02132-y)
Supplement: Supplementary file 2 — Additional file 2. [file 12874_2023_2132_MOESM2_ESM.zip › Supp_Files_R1/Figure_and_Table_Generation_v3.nb.html]

R Script for Data Analysis and Table Generation


Code 

- Show All Code
- Hide All Code
- Download Rmd

# R Script for Data Analysis and Table Generation

# ADEMP Overview

## Aims

The aim of this simulation is to demonstrate the performance of the
three re-expression methods (Rodriguez-Barranco, rb; Alternate, alt;
Dzierlenga, dz) when utilized for a range of different, realistic
populations and scenarios.

## Data-generating mechanisms

Data will be generated by producing parametric draws from a known,
truncated lognormal distribution using the EnvStats::rlnormTrunc()
function. The number of individuals simulated (nobs) was selected by
finding the 10th and 90th percentiles of the number of individuals
included in the 14 studies examined in this investigation. A list of
values representing a reasonable range of x-value means was
log-transformed and used for meanlog (mu) for the distribution, while
sigma values were selected to be 0.5 or 1 and used for sdlog
(sigma).

## Estimands

Our estimand of interest in this study is the re-expressed regression
model fit parameter, betarex.

## Methods

1. This investigation will use 1000 simulated distributions (nsim) for
   each set of parameters to generate x-values
2. Y-values will then be calculated using y = beta \* x + error where
   beta is set to 0.5, 1, or 10 and error is randomly assigned from a
   normal distribution with a mean of 0 and standard deviation of beta \* mu
   \* 0.2.
3. Regression models (log-linear or linear depending on the direction
   of re-expression) will then be generated from the x and y values
4. Re-expressed betas are then generated using each of the
   re-expression functions defined below (rbexp, altexp, dzexp)
5. Finally, diagnostic values, including relative bias and Monte Carlo
   standard error of the relative bias, are calculated in order to
   determine how well the re-expressed betas reflect true beta values.

## Performance Measures

The accuracy of the re-expressed beta values will be calculated as
the relative bias using the following formula:

Relative Bias = 1/nsim \* sum((betarex - beta)/beta)

while the skewedness of the re-expressed value was calculated using
the Monte Carlo standard error of the relative bias:

MCSE = sqrt((1/(nsim - (nsim - 1))) \* sum(((betarex -
beta)/beta)^2))

# Setup

Load in the needed packages


```
list.of.packages <- c(
  "foreach",
  "doParallel",
  "ggplot2",
  "dplyr",
  "EnvStats",
  "survey",
  "data.table",
  "doRNG",
  "doSNOW",
  "tcltk",
  "knitr",
  "MASS",
  "tidyverse",
  "ggnewscale",
  "ggpubr",
  "ggrepel"
  )

new.packages <- list.of.packages[!(list.of.packages %in% installed.packages()[,"Package"])]

if(length(new.packages) > 0){
  install.packages(new.packages, dep=TRUE)
}

#loading packages
for(package.i in list.of.packages){
  suppressPackageStartupMessages(
    library(
      package.i, 
      character.only = TRUE
      )
    )
}
```


Load in the dataframes


```
data <- read.csv(file = "data/Re_expression_Full_Table_11072022.csv")
datab <- read.csv(file = "data/Re_expression_Full_Table_10162023.csv")

#The next dataset, the nscenario output, can be regenerated by running fullsim.R
#Note that the output .csv will have the date that the simulation was completed
#requiring that the below line be updated with the desired file date
lrexbetatab <- fread("lrexbetatab_2023-02-22.csv")

#Finally, for working with each individual nsim for all nscenario
#the below will generate a single dataset. It will take a few seconds to load,
#and the values will change if the full simulation has been run again.
files <- list.files(path = "simdata",pattern = ".csv")
temp <- lapply(paste0("simdata/",files),fread,sep=",")
allsimdata <- rbindlist(temp)
rm(files,temp)
```


Load in the re-expression functions


```
source("./functions/rbexp.R")
source("./functions/dzexp.R")
source("./functions/altexp.R")
```

# Figure 1

First we set up the administrative things for our simple
pseudosimulation to generate Figure 1, including setting a seed to get
reproducible results and defining a few variables such as our list of
x\_medians of interest. This simulates a specific set of scenarios
(median, sigma, etc.) as defined below and those values can be changed
to explore other scenarios.


```
set.seed(1234)

medlist <- 1
nrep <- 2E3
mu <- log(medlist)
sigma <- 0.5 
logbase <- exp(1) 
beta <- 1 
err <- 0
rel <- "log" 

# Generate the x-values by sampling from a lognormal distribution "nrep" times, 
# and then we generate y-values based on those x-values.
xlog = rlnormTrunc(nrep, meanlog = log(medlist), sdlog = sigma)
ylog = beta * log(xlog, base = logbase)

#Storing values from the linear regression of y vs. x or log(x).
lmlin <- lm(ylog~xlog)
lmlog <- lm(ylog~log(xlog, base = logbase))

rsquare <- summary(lmlin)$r.squared
beta <- lmlin$coef[[2]]
lbeta <- lmlog$coef[[2]]
lb_low_ci <- confint(lmlog)[[2,1]]
lb_hi_ci <- confint(lmlog)[[2,2]]
  
fig1df <- data.frame("ex_no" = 1, 
                         "x_median" = round(median(xlog), 2), 
                         "x_25" = round(summary(xlog),2)[[2]], 
                         "x_75" = round(summary(xlog),2)[[5]],
                         "rb" = rbexp(model = "b", 
                              y.exp = "abs", 
                              x.exp = "abs", 
                              beta = lbeta, 
                              se = (lb_hi_ci-lb_low_ci)/3.92,
                              mean.x = median(xlog),
                              mean.y = 0,
                              a = logbase, 
                              b = logbase, 
                              k = logbase, 
                              c = 1)[[1]], 
                     "dz" = dzexp(input.base = "log",
                              exp.dist = c(med = median(xlog), 
                                           iqr1 = summary(xlog)[[2]], 
                                           iqr3 =summary(xlog)[[5]]),
                              beta.dist = c(lbeta,
                                            lb_low_ci, 
                                            lb_hi_ci),
                              log.base = logbase)[[1]], 
                         "alt" = altexp(input.base = "log", 
                                median = median(xlog),
                                beta.dist = c(lbeta,
                                              lb_low_ci, 
                                              lb_hi_ci),
                                log.base = logbase)[[1]], 
                         "observed_beta" = round(beta,2),
                         "rsquare" = rsquare)
```


## Pseudosimulation Figure

We can then take the values simulated to generate a figure that can
help demonstrate the range over which a given re-expression method may
be useful.


```
p1 <- ggplot()+
  geom_line(aes(x = xlog, y = ylog, color = 'og'), size = 1.4, linetype = 1)+
  geom_segment(aes(x = 0, y = 0*fig1df$observed_beta-fig1df$observed_beta, xend = (max(ylog)+fig1df$observed_beta)/fig1df$observed_beta, yend = max(ylog), color = 'obs'),size = 1)+
  geom_segment(aes(x = 0, y = 0*fig1df$rb-fig1df$rb, xend = (max(ylog)+fig1df$rb)/fig1df$rb, yend = max(ylog), color = 'rb'),size = 1, linetype = 6)+
  geom_segment(aes(x = 0, y = (0)*fig1df$dz-fig1df$dz, xend = (max(ylog)+fig1df$dz)/fig1df$dz, yend = max(ylog), color = 'dz'),size = 1.5,linetype = 3)+
  geom_segment(aes(x = 0, y = (0)*fig1df$alt-fig1df$alt, xend = (max(ylog)+fig1df$alt)/fig1df$alt, yend = max(ylog), color = 'alt'),size = 1, linetype = 2)+
  scale_colour_manual(name = '',
                      values =c('og'='black','obs' = '#E69F00', 'rb' = '#CC79A7','dz'='#56B4E9','alt'='#D55E00'),
                      # labels = c("Simulated Data","Standard", "Rodriguez Barranco","Alternate","Dzierlenga"),
                      labels = c("Simulated\nData", 
                                 expression(italic("\u03b2")[Estimand]),
                                expression(italic("\u03b2")[RB]),
                                expression(italic("\u03b2")[Dz]),
                                expression(italic("\u03b2")[Alt])),
                      guide = guide_legend(override.aes = list(linetype = c(1,1,6,3,2),lwd = c(1,1,1,1,1))))+
  theme_bw()+
  scale_y_continuous(name = "Outcome Variable Values\n")+
  scale_x_continuous(name = "\nExposure Variable Values")+
  geom_vline(aes(xintercept = fig1df$x_25))+
  geom_vline(aes(xintercept = fig1df$x_75))+
  annotate(geom = "text", x = fig1df$x_25-(fig1df$x_25*0.4), y = max(ylog)*0.8, label = paste0("Quartile 1\n= ", round(fig1df$x_25,2)), fontface = 2)+
  annotate(geom = "text", x = fig1df$x_75+(fig1df$x_75*0.2), y = max(ylog)*0.8, label = paste0("Quartile 3\n= ", round(fig1df$x_75,2)), fontface = 2)+
  annotate(geom = "text", x = ((max(ylog)+fig1df$observed_beta)/fig1df$observed_beta)*1.01, y = max(ylog)*1.08, color = 'goldenrod', fontface = 2, label = "Standard linear regression line\nfit to the simulated data")+
  geom_segment(aes(x = ((max(ylog)+fig1df$observed_beta)/fig1df$observed_beta)*0.95, y = max(ylog)*1.02, xend = (max(ylog)*0.93 +fig1df$observed_beta)/fig1df$observed_beta, yend = max(ylog)*0.95),size = 0.75,color = 'black')+
  theme(axis.title.x = element_text(size = 14, face = 'bold'),
        axis.text.x = element_text(size = 12, face = 'bold'),
        axis.title.y = element_text(size = 14, face = 'bold'),
        axis.text.y = element_text(size = 12, face = 'bold'),
        legend.title = element_text(size = 14, face = 'bold'),
        legend.text = element_text(size = 12, face = 'bold'),
        legend.key.width = unit(2,"line"),
        legend.text.align = 0)
```


```
Warning: Using `size` aesthetic for lines was deprecated in ggplot2 3.4.0.
Please use `linewidth` instead.
```


```
p1
```

# Table 1

First we need to do some light formatting of the datasets used for
this part of the analysis. For example, we can generate a small table
detailing some of the information from the actual studies and the
simulation dataset that details the scenarios used in the simulation


```
studylist <- c("Bulka 2021", "Lee 2020","Odebeatu 2019",
               "Xu 2020","Stein 2016","Pilkerton 2018",
               "Cheang 2021","Abraham 2020","Apelberg 2007",
               "Washino 2009","Hamm 2010", "Chen 2012",
               "Darrow 2013","Steenland 2009") #Study IDs
studyn <- c(8778, 124,7765, 1947, 1191, 621, 2899, 
            101, 293, 428, 252, 429, 1630, 46294) #n in each study
aggn <- round(c(quantile(studyn, 0.1),quantile(studyn, 0.25),
                quantile(studyn, 0.5),quantile(studyn, 0.75),
                quantile(studyn, 0.9)), digits = 0) #n of various quantiles for studies overall

rel <- c("log") #For this investigation, only re-expressed from log to lin
logbase <- c(exp(1),2,10) #various log bases investigated
sigma <- c(0.25,0.45,0.65,0.85) #various sigma values investigated
beta <- c(-15,0.5,1,10,30) #BetaDGM, Note that including a beta = 0 breaks things because we're normally filtering out very small betas because even a small change in a very small beta can result in a huge relative bias value that's not really relevant on most scales (e.g. difference between 1.0E-8 and 1.0E-9)
medlist <- c(0.25,0.5,1,2,4,8,16,32) #Medians
nobs <- c(aggn[[1]],aggn[[5]]) #Considered the 10th and 90th %ile of actual study n's
nsim <- 2000 #2000 simulations chosen for this investigation

simdf <- as.data.table(expand.grid(nobs = nobs,medlist = medlist,beta = beta,sigma = sigma,logbase = logbase,rel = rel))
simdf$ex_no <- c(1:nrow(simdf))
simdf <- rev(simdf)
simdf$nsim <- nsim

lrexbetatab$logbase[lrexbetatab$logbase > 2 & lrexbetatab$logbase < 3] <- "e"
allsimdata$logbase[allsimdata$logbase == exp(1)] <- "e"

allsimdata <- allsimdata[,c("ex_no","xlog","error","errsd","ylog","x_median","x_25","x_75","rbl","rb","rbu","altl","alt","altu","dzl","dz","dzu","b_low_ci","observed_beta","b_hi_ci","rsquare","RelBias_rbl","RelBias_rb","RelBias_rbu","RelBias_altl","RelBias_alt","RelBias_altu","RelBias_dzl","RelBias_dz","RelBias_dzu","MC_SE_RelBias_rb","MC_SE_RelBias_alt","MC_SE_RelBias_dz")]
allsimdata <- merge(simdf,allsimdata,by = "ex_no")

#Create a dataframe for when beta > 0
lrexbetareg <- lrexbetatab[beta>0]
```


Then we can generate Table 1


```
tab1 <- data.frame("Parameter" = c("n~obs~",
                                   "e",
                                   "\u03b2~DGM~",
                                   "Logbase",
                                   "S",
                                   "median"),
                   "Possible Values" = c(paste(nobs,collapse = ", "),
                                         "Selected from a normal distribution with mean = 0 and SD = SDe*",
                                          paste(beta, collapse = ", "),
                                          paste(logbase, collapse = ", "),
                                          paste(sigma, collapse = ", "),
                                          paste(medlist, collapse = ", ")),
                   "Rationale for Choice" = c("Quantiles (10th,90th) of the distribution of sample sizes for the 15 real data examples",
                                              "Standard deviation selected to result in an R^2 ~ 0.2, to make realistic models",
                                              "Broad range of effect sizes encompassing those int he real data examples.",
                                              "Log bases used in the 15 real data examples",
                                              "Selected values cover the approximate range of sigma values in the 15 real data examples.",
                                              "Selected values cover the range of median exposure in the 15 real data examples."))
tab1 <- knitr::kable(tab1)

tab1
```


| Parameter | Possible.Values | Rationale.for.Choice |
| --- | --- | --- |
| nobs | 162, 8474 | Quantiles (10th,90th) of the distribution of sample sizes for the 15 real data examples |
| e | Selected from a normal distribution with mean = 0 and SD = SDe\* | Standard deviation selected to result in an R^2 ~ 0.2, to make realistic models |
| βDGM | -15, 0.5, 1, 10, 30 | Broad range of effect sizes encompassing those int he real data examples. |
| Logbase | 2.71828182845905, 2, 10 | Log bases used in the 15 real data examples |
| S | 0.25, 0.45, 0.65, 0.85 | Selected values cover the approximate range of sigma values in the 15 real data examples. |
| median | 0.25, 0.5, 1, 2, 4, 8, 16, 32 | Selected values cover the range of median exposure in the 15 real data examples. |

# Figure 2

## Ordinary Least Squares Regression

Here is where we run the ordinary least squares regression and pick
the most appropriate model based on minimizing AIC with a k-parameter
~3.84.


```
#Fit for Relative Bias vs. sigma for Alternative Method
  #Baseline fit with all variables included
fit2p5 <- lm(RelBias_alt~poly(sigma,2,raw=T)+
               C(as.factor(logbase),base = 3)+logbase:sigma+
               beta+beta:sigma+
               medlist+medlist:sigma+
               nobs+nobs:sigma, data = lrexbetareg) 
  #Minimize AIC based on a k = p < 0.05 in Chi-sq test
step.2p5 <- stepAIC(fit2p5,direction = "both",trace = F, 
                    k = qchisq(0.05,1,lower.tail = FALSE)) 
# summary(step.2p5)
  #Based on variables retained above, generate fits for the lower and upper confidence intervals
fit2p5l <- lm(RelBias_altl~poly(sigma,2,raw=T)+C(as.factor(logbase),base = 3)+logbase:sigma+nobs+nobs:sigma, data = lrexbetareg)
# summary(fit2p5l)
 fit2p5u <- lm(RelBias_altu~poly(sigma,2,raw=T)+C(as.factor(logbase),base = 3)+logbase:sigma+nobs+nobs:sigma, data = lrexbetareg)
# summary(fit2p5u)

#Fit for Relative Bias vs. sigma for Rodriguez-Barranco Method
fit2p6 <- lm(RelBias_rb~poly(sigma,2,raw=T)+
               logbase+logbase:sigma+
               beta+beta:sigma+
               medlist+medlist:sigma+
               nobs+nobs:sigma, data = lrexbetareg)
step.2p6 <- stepAIC(fit2p6,direction = "both",trace = F, 
                    k = qchisq(0.05,1,lower.tail = FALSE))
# summary(step.2p6)
fit2p6l <- lm(RelBias_rbl~poly(sigma,2,raw=T)+medlist+sigma:medlist, data = lrexbetareg)
# summary(fit2p6l)
fit2p6u <- lm(RelBias_rbu~poly(sigma,2,raw=T)+medlist+sigma:medlist, data = lrexbetareg)
# summary(fit2p6u)

#Fit for Relative Bias vs. sigma for Dzierlenga Method
fit2p7 <- lm(RelBias_dz~poly(sigma,2,raw=T)+
               C(as.factor(logbase),base = 3)+logbase:sigma+
               beta+beta:sigma+
               medlist+medlist:sigma+
               nobs+nobs:sigma, data = lrexbetareg)
step.2p7 <- stepAIC(fit2p7,direction = "both",trace = F, 
                    k = qchisq(0.05,1,lower.tail = FALSE))
# summary(step.2p7)
fit2p7l <- lm(RelBias_dzl~poly(sigma,2,raw=T)+nobs+nobs:sigma, data = lrexbetareg)
# summary(fit2p7l)
fit2p7u <- lm(RelBias_dzu~poly(sigma,2,raw=T)+nobs+nobs:sigma, data = lrexbetareg)
# summary(fit2p7u)

#We then repeat the same thing for the full nsim*nscenario dataset (where beta > 0)
fit2p5f <- lm(RelBias_alt~poly(sigma,2,raw=T)+
                logbase+logbase:sigma+
                beta+beta:sigma+
                medlist+medlist:sigma+
                nobs+nobs:sigma, data = allsimdata[beta > 0])
step.2p5f <- stepAIC(fit2p5f,direction = "both",trace = F, 
                     k = qchisq(0.05,1,lower.tail = FALSE))
# summary(step.2p5f)

fit2p6f <- lm(RelBias_rb~poly(sigma,2,raw=T)+
                logbase+logbase:sigma+
                beta+beta:sigma+
                medlist+medlist:sigma+
                nobs+nobs:sigma, data = allsimdata[beta > 0])
step.2p6f <- stepAIC(fit2p6f,direction = "both",trace = F, 
                     k = qchisq(0.05,1,lower.tail = FALSE))
# summary(step.2p6f)

fit2p7f <- lm(RelBias_dz~poly(sigma,2,raw=T)+
                logbase+logbase:sigma+
                beta+beta:sigma+
                medlist+medlist:sigma+
                nobs+nobs:sigma, data = allsimdata[beta > 0])
step.2p7f <- stepAIC(fit2p7f,direction = "both",trace = F, 
                     k = qchisq(0.05,1,lower.tail = FALSE))
#summary(step.2p7f)
```

# Refits


```
#Fit for Relative Bias vs. sigma for Alternative Method
  #Baseline fit with all variables included
fit2p5r <- lm(RelBias_alt~poly(sigma,2,raw=T)+
               C(as.factor(logbase),base = 3)+logbase:sigma+
               beta+beta:sigma+
               medlist+medlist:sigma+
               nobs+nobs:sigma+
               rsquare+rsquare:sigma, data = lrexbetareg) 
  #Minimize AIC based on a k = p < 0.05 in Chi-sq test
step.2p5r <- stepAIC(fit2p5,direction = "both",trace = F, 
                    k = qchisq(0.05,1,lower.tail = FALSE)) 
# summary(step.2p5r)
  #Based on variables retained above, generate fits for the lower and upper confidence intervals
fit2p5lr <- lm(RelBias_altl~poly(sigma,2,raw=T)+C(as.factor(logbase),base = 3)+logbase:sigma+nobs+nobs:sigma, data = lrexbetareg)
# summary(fit2p5lr)
 fit2p5ur <- lm(RelBias_altu~poly(sigma,2,raw=T)+C(as.factor(logbase),base = 3)+logbase:sigma+nobs+nobs:sigma, data = lrexbetareg)
# summary(fit2p5ur)

#Fit for Relative Bias vs. sigma for Rodriguez-Barranco Method
fit2p6r <- lm(RelBias_rb~poly(sigma,2,raw=T)+
               logbase+logbase:sigma+
               beta+beta:sigma+
               medlist+medlist:sigma+
               nobs+nobs:sigma+
               rsquare+rsquare:sigma, data = lrexbetareg)
step.2p6r <- stepAIC(fit2p6,direction = "both",trace = T, 
                    k = qchisq(0.05,1,lower.tail = FALSE))
```


```
Start:  AIC=-2208.36
RelBias_rb ~ poly(sigma, 2, raw = T) + logbase + logbase:sigma + 
    beta + beta:sigma + medlist + medlist:sigma + nobs + nobs:sigma

                          Df Sum of Sq    RSS     AIC
- logbase:sigma            2    0.0038 40.584 -2216.0
- sigma:beta               1    0.0000 40.581 -2212.2
- sigma:nobs               1    0.1207 40.701 -2209.9
<none>                                 40.581 -2208.4
- sigma:medlist            1    1.9670 42.548 -2175.8
- poly(sigma, 2, raw = T)  1    4.3070 44.888 -2134.7

Step:  AIC=-2215.98
RelBias_rb ~ poly(sigma, 2, raw = T) + logbase + beta + medlist + 
    nobs + sigma:beta + sigma:medlist + sigma:nobs

                          Df Sum of Sq    RSS     AIC
- logbase                  2    0.0534 40.638 -2222.7
- beta:sigma               1    0.0000 40.584 -2219.8
- nobs:sigma               1    0.1207 40.705 -2217.5
<none>                                 40.584 -2216.0
+ logbase:sigma            2    0.0038 40.581 -2208.4
- medlist:sigma            1    1.9670 42.551 -2183.5
- poly(sigma, 2, raw = T)  2   16.5984 57.183 -1960.3

Step:  AIC=-2222.65
RelBias_rb ~ poly(sigma, 2, raw = T) + beta + medlist + nobs + 
    beta:sigma + medlist:sigma + nobs:sigma

                          Df Sum of Sq    RSS     AIC
- beta:sigma               1    0.0000 40.638 -2226.5
- nobs:sigma               1    0.1207 40.759 -2224.2
<none>                                 40.638 -2222.7
+ logbase                  2    0.0534 40.584 -2216.0
- medlist:sigma            1    1.9670 42.605 -2190.2
- poly(sigma, 2, raw = T)  2   16.5984 57.236 -1967.3

Step:  AIC=-2226.49
RelBias_rb ~ poly(sigma, 2, raw = T) + beta + medlist + nobs + 
    medlist:sigma + nobs:sigma

                          Df Sum of Sq    RSS     AIC
- beta                     1    0.0001 40.638 -2230.3
- nobs:sigma               1    0.1207 40.759 -2228.1
<none>                                 40.638 -2226.5
+ sigma:beta               1    0.0000 40.638 -2222.7
+ logbase                  2    0.0534 40.584 -2219.8
- medlist:sigma            1    1.9670 42.605 -2194.0
- poly(sigma, 2, raw = T)  2   20.0921 60.730 -1925.6

Step:  AIC=-2230.33
RelBias_rb ~ poly(sigma, 2, raw = T) + medlist + nobs + medlist:sigma + 
    nobs:sigma

                          Df Sum of Sq    RSS     AIC
- nobs:sigma               1    0.1207 40.759 -2231.9
<none>                                 40.638 -2230.3
+ beta                     1    0.0001 40.638 -2226.5
+ logbase                  2    0.0534 40.585 -2223.7
- medlist:sigma            1    1.9670 42.605 -2197.9
- poly(sigma, 2, raw = T)  2   20.0921 60.730 -1929.5

Step:  AIC=-2231.89
RelBias_rb ~ poly(sigma, 2, raw = T) + medlist + nobs + medlist:sigma

                          Df Sum of Sq    RSS     AIC
- nobs                     1     0.015 40.773 -2235.4
<none>                                 40.759 -2231.9
+ sigma:nobs               1     0.121 40.638 -2230.3
+ beta                     1     0.000 40.759 -2228.1
+ logbase                  2     0.053 40.705 -2225.2
- medlist:sigma            1     1.967 42.726 -2199.5
- poly(sigma, 2, raw = T)  2    33.818 74.577 -1775.6

Step:  AIC=-2235.45
RelBias_rb ~ poly(sigma, 2, raw = T) + medlist + medlist:sigma

                          Df Sum of Sq    RSS     AIC
<none>                                 40.773 -2235.4
+ nobs                     1     0.015 40.759 -2231.9
+ beta                     1     0.000 40.773 -2231.6
+ logbase                  2     0.053 40.720 -2228.8
- medlist:sigma            1     1.967 42.741 -2203.1
- poly(sigma, 2, raw = T)  2    33.818 74.591 -1779.3
```


```
# summary(step.2p6r)
fit2p6lr <- lm(RelBias_rbl~poly(sigma,2,raw=T)+medlist+sigma:medlist, data = lrexbetareg)
# summary(fit2p6lr)
fit2p6ur <- lm(RelBias_rbu~poly(sigma,2,raw=T)+medlist+sigma:medlist, data = lrexbetareg)
# summary(fit2p6ur)

#Fit for Relative Bias vs. sigma for Dzierlenga Method
fit2p7r <- lm(RelBias_dz~poly(sigma,2,raw=T)+
               C(as.factor(logbase),base = 3)+logbase:sigma+
               beta+beta:sigma+
               medlist+medlist:sigma+
               nobs+nobs:sigma+
               rsquare+rsquare:sigma, data = lrexbetareg)
step.2p7r <- stepAIC(fit2p7,direction = "both",trace = F, 
                    k = qchisq(0.05,1,lower.tail = FALSE))
# summary(step.2p7r)
fit2p7lr <- lm(RelBias_dzl~poly(sigma,2,raw=T)+nobs+nobs:sigma, data = lrexbetareg)
# summary(fit2p7lr)
fit2p7ur <- lm(RelBias_dzu~poly(sigma,2,raw=T)+nobs+nobs:sigma, data = lrexbetareg)
# summary(fit2p7ur)

#We then repeat the same thing for the full nsim*nscenario dataset (where beta > 0)
fit2p5fr <- lm(RelBias_alt~poly(sigma,2,raw=T)+
                logbase+logbase:sigma+
                beta+beta:sigma+
                medlist+medlist:sigma+
                nobs+nobs:sigma+
               rsquare+rsquare:sigma, data = allsimdata[beta > 0])
step.2p5fr <- stepAIC(fit2p5f,direction = "both",trace = F, 
                     k = qchisq(0.05,1,lower.tail = FALSE))
# summary(step.2p5fr)

fit2p6fr <- lm(RelBias_rb~poly(sigma,2,raw=T)+
                logbase+logbase:sigma+
                beta+beta:sigma+
                medlist+medlist:sigma+
                nobs+nobs:sigma+
               rsquare+rsquare:sigma, data = allsimdata[beta > 0])
step.2p6fr <- stepAIC(fit2p6f,direction = "both",trace = F, 
                     k = qchisq(0.05,1,lower.tail = FALSE))
# summary(step.2p6fr)

fit2p7fr <- lm(RelBias_dz~poly(sigma,2,raw=T)+
                logbase+logbase:sigma+
                beta+beta:sigma+
                medlist+medlist:sigma+
                nobs+nobs:sigma+
               rsquare+rsquare:sigma, data = allsimdata[beta > 0])
step.2p7fr <- stepAIC(fit2p7f,direction = "both",trace = F, 
                     k = qchisq(0.05,1,lower.tail = FALSE))
#summary(step.2p7fr)

# summary(allsimdata$rsquare)
```


Those models can then be used to guide how the fits are displayed in
Figure 2


```
fig2c <- ggplot(data = lrexbetareg, aes(x = sigma, y = RelBias_alt))+
  geom_jitter(aes(shape = as.factor(logbase), size = medlist
                  , fill = as.character(nobs)
                  )
              , color = "black"
              )+
  geom_smooth(data = lrexbetareg, method = 'lm',
              formula = y~poly(x,2,raw=T),
              aes(shape = as.factor(logbase),
                  color = as.character(nobs),
                  linetype = as.factor(logbase)),
              alpha = 0.0,
              size = 1.1) +
  scale_x_continuous(name = "Sigma",breaks = c(0.25,0.45,0.65,0.85))+
  scale_y_continuous(name = "Relative Bias Value (Alternative Method)", lim = c(-0.75,1.25))+
  scale_color_manual(name = "Number of Observations (+ Fitted Curves)",
                     values = c("#D55E00","#56B4E9"),
                     guide = "none"
                     )+
  scale_fill_manual(name = "Number of Observations\n(+ Fitted Curves)",
                    values = alpha(c("#D55E00","#56B4E9"),c(1,1)))+
  scale_shape_manual(name = "Logarithm Base",
                       breaks = c(2,"e",10),
                       labels = c("2","e","10"),
                       # values = c(15,18,20)
                       values = c(21,22,24)
                     )+
  scale_linetype_manual(name = "Fitted Curves for Logarithm Base",
                       breaks = c(10,"e",2),
                       labels = c("10","e","2"),
                       values = c(3,2,1))+
  scale_size(range = c(1,4),name = "Median Value",
             guide = guide_legend(reverse = TRUE)) +
  geom_vline(xintercept = sigma)+
  theme_bw()+
  guides(size = guide_legend(order = 1, reverse = TRUE),
         shape = guide_legend(order = 2,
                              override.aes = list(size = 3)),
         fill = guide_legend(order = 4,
                             reverse = TRUE,
                             override.aes = list(color = c("#56B4E9","#D55E00"),
                                                 alpha = 1,
                                                 size = 6,
                                                 shape = 15)),
         linetype = guide_legend(order = 3, reverse = TRUE, 
                                 override.aes = list(color = "black")
                                 ))+
  theme(axis.title = element_text(size = 14, face = "bold"),
        axis.text = element_text(size = 12, face = "bold"),
        legend.title = element_text(size = 12, face = "bold"),
        legend.text = element_text(size = 12, face = "bold"),
        legend.key.width = unit(3,"line"))


fig2a <- ggplot(data = lrexbetareg, aes(x = sigma, y = RelBias_rb))+
  geom_jitter(aes(shape = as.factor(logbase), size = medlist, fill = as.character(nobs)),
              color = "black"
              # , alpha = 0.5
              )+
  geom_smooth(data = lrexbetareg, method = 'lm',
              formula = y~poly(x,2,raw=T),
              aes(linetype = as.factor(medlist)),
              alpha = 0.0,
              color = "blue") +
  scale_x_continuous(name = "Sigma",breaks = c(0.25,0.45,0.65,0.85))+
  scale_y_continuous(name = "Relative Bias Value (Rodriguez-Barranco Method)", lim = c(-0.75,1.25))+
  scale_shape_manual(name = "Logarithm Base",
                       breaks = c(2,"e",10),
                       labels = c("2","e","10"),
                       values = c(21,22,24)
                     )+
  scale_fill_manual(name = "Number of Observations",
                    values = c("#D55E00","#56B4E9"))+
  scale_linetype_discrete(name = "Fitted Curves for Median Values")+
  scale_size(range = c(1,4),
             name = "Median Value",
             guide = guide_legend(reverse = TRUE)) +
  geom_vline(xintercept = sigma)+
  theme_bw()+
  guides(size = guide_legend(order = 2, reverse = TRUE),
         shape = guide_legend(order = 3,
                              override.aes = list(size = 3)),
         fill = guide_legend(order = 4, reverse = TRUE,
                              override.aes = list(shape = 15, 
                                                  size = 6, 
                                                  color = c("#56B4E9","#D55E00"),
                                                  alpha = 1)),
         linetype = guide_legend(order = 1, reverse = TRUE))+
  theme(axis.title = element_text(size = 14, face = "bold"),
        axis.text = element_text(size = 12, face = "bold"),
        legend.title = element_text(size = 12, face = "bold"),
        legend.text = element_text(size = 12, face = "bold"),
        legend.key.width = unit(3,"line"))

fig2b <- ggplot(data = lrexbetareg, aes(x = sigma, y = RelBias_dz))+ 
  geom_jitter(aes(shape = as.factor(logbase), size = medlist, fill = as.character(nobs)),
              color = "black"
              )+
  geom_smooth(data = lrexbetareg, method = 'lm', 
              formula = y~poly(x,2,raw=T),
              aes(color = as.factor(nobs)),alpha = 0.0) + 
  scale_x_continuous(name = "Sigma",breaks = c(0.25,0.45,0.65,0.85))+
  scale_y_continuous(name = "Relative Bias Value (Dzierlenga Method)", lim = c(-0.75,1.25))+
  scale_color_manual(name = "Number of Observations",
                     values = c("#D55E00","#56B4E9"),
                     guide = "none"#guide_legend(reverse = TRUE)
                     )+
  scale_fill_manual(name = "Number of Observations\n(+ Fitted Curves)",
                    values = alpha(c("#D55E00","#56B4E9"),c(1,1)))+
  scale_shape_manual(name = "Logarithm Base",
                       breaks = c(2,"e",10),
                       labels = c("2","e","10"),
                       values = c(21,22,24))+
  scale_size(range = c(1,4),name = "Median Value",
             guide = guide_legend(reverse = TRUE)) +
  geom_vline(xintercept = sigma)+
  theme_bw()+
  guides(size = guide_legend(order = 1, reverse = TRUE),
         shape = guide_legend(order = 2,
                              override.aes = list(size = 3)),
         fill = guide_legend(order = 3,
                             reverse = TRUE,
                             override.aes = list(color = c("#56B4E9","#D55E00"),
                                                 alpha = 1,
                                                 size = 6,
                                                 shape = 15)))+
  theme(axis.title = element_text(size = 14, face = "bold"), 
        axis.text = element_text(size = 12, face = "bold"),
        legend.title = element_text(size = 12, face = "bold"),
        legend.text = element_text(size = 12, face = "bold"),
        legend.key.width = unit(3,"line"))

#Creating a dataframe with the specific scenarios of interest and converting it
#from wide to long for Figure 2D
lrexbetatabsub <- lrexbetatab[beta == 0.5 & medlist %in% c(0.5,8)&logbase %in% c(2,10),]
lrexbetalong <- melt(lrexbetatabsub,measure = c("RelBias_rb","RelBias_alt","RelBias_dz"))

fig2d <- ggplot(data = lrexbetalong, 
               aes(x = sigma, y = value, fill = variable))+
  geom_jitter(shape = 21,
              color = "black",
              size = 3
              # , alpha = 0.5
              )+
  geom_smooth(data = lrexbetalong[rel == "log" & (variable %in% c("RelBias_dz"))],
              method = 'lm', 
              formula = y~poly(x,2,raw=T), 
              alpha = 0.0,
              color = "#009E73",
              size = 1.5) +
  geom_smooth(data = lrexbetalong[rel == "log" & (variable %in% c("RelBias_alt"))],
              method = 'lm', 
              formula = y~poly(x,2,raw=T),
              aes(linetype = logbase), 
              alpha = 0.0,
             color = "#CC79A7",
             size = 1.5)+ 
  scale_x_continuous(name = "Sigma",breaks = c(0.25,0.45,0.65,0.85))+
  scale_y_continuous(name = "Relative Bias Value", lim = c(-0.75,1.25))+
  scale_color_manual(name = "Re-expression\nAlgorithm",
                       breaks = c("RelBias_rb","RelBias_dz","RelBias_alt"),
                       labels = c(expression("\u03b2"[RB]),
                                  expression("\u03b2"[Dz]),
                                  expression("\u03b2"[Alt])),
                       values = c("#E69F00","#009E73","#CC79A7"),
                       guide = "none")+
    scale_fill_manual(name = "Re-expression\nAlgorithm",
                      breaks = c("RelBias_rb","RelBias_dz","RelBias_alt"),
                      labels = c(expression(italic("\u03b2")[RB]),
                                 expression(italic("\u03b2")[Dz]),
                                 expression(italic("\u03b2")[Alt])),
                      values = c("#E69F00","#009E73","#CC79A7")
                      )+
  scale_linetype_manual(name = "Log Base (Alternative\nRegression Estimator)",
                        guide = guide_legend(reverse = TRUE),
                        values = c(1,4))+
  new_scale("linetype")+
    geom_smooth(data = lrexbetalong[rel == "log" & (variable == "RelBias_rb")],
              color = "#E69F00", method = "lm",formula = y~poly(x,2,raw=T),
              aes(linetype = as.factor(medlist)), alpha = 0.0, size = 1.5)+
    scale_linetype_discrete(name = "Median (Rodriguez-Barranco\nRegression Estimator)",
             guide = guide_legend(reverse = TRUE))+
  geom_vline(xintercept = sigma)+
  theme_bw()+
  guides(fill = guide_legend(order = 1,
                             override.aes = list(color = c("#E69F00","#009E73","#CC79A7"))
                             ))+
  theme(axis.title = element_text(size = 14, face = "bold"), 
        axis.text = element_text(size = 12, face = "bold"),
        legend.title = element_text(size = 12, face = "bold"),
        legend.text = element_text(size = 12, face = "bold"),
        legend.key.width = unit(3,"line"))
ggarrange(fig2a,fig2b,fig2c,fig2d,nrow = 2)
```


```
$`1`

$`2`

attr(,"class")
[1] "list"      "ggarrange"
```


```
# Creates figure 2-- Uncomment to generate
# pdf("figure2.pdf",width = 15, height = 8)
# ggpubr::ggarrange(fig2a,fig2b, labels = c("A","B"), align = "v")
# ggpubr::ggarrange(fig2c,fig2d, labels = c("C","D"), align = "v")
# dev.off()

lrexbetareg$rsqcat <- 0
lrexbetareg$rsqcat <- ifelse(lrexbetareg$rsquare < 0.07,"<0.07",
                             ifelse(lrexbetareg$rsquare > 0.34, ">0.34","0.07-0.34"))

altfig2a <- ggplot(data = lrexbetareg, aes(x = sigma, y = RelBias_rb))+
  geom_jitter(aes(shape = as.factor(logbase), size = medlist, fill = as.character(nobs)),
              color = "black"
              # , alpha = 0.5
              )+
 geom_line(data = lrexbetareg, 
            stat = 'smooth',
            method = 'lm',
              formula = y~poly(x,2,raw=T),
              aes(linetype = as.factor(medlist),
                  alpha = rsqcat),
              color = "blue",
           size = 1.1) +
  scale_x_continuous(name = "Sigma",breaks = c(0.25,0.45,0.65,0.85))+
  scale_y_continuous(name = "Relative Bias Value (Rodriguez-Barranco Method)", lim = c(-1.25,1.25))+
  scale_shape_manual(name = "Logarithm Base",
                       breaks = c(2,"e",10),
                       labels = c("2","e","10"),
                       # values = c(15,18,20)
                       values = c(21,22,24)
                     )+
  scale_fill_manual(name = "Number of Observations",
                    values = c("#D55E00","#56B4E9"))+
  scale_linetype_discrete(name = "Fitted Curves for Median Values")+
    scale_alpha_manual(name = "Rsquared Value",
                     values = c(0.33,0.67,1))+
  scale_size(range = c(1,4),
             # breaks = c(0.25,0.5,1,2,4,8,16,32),
             name = "Median Value",
             guide = guide_legend(reverse = TRUE)) +
  geom_vline(xintercept = sigma)+
  theme_bw()+
  guides(size = guide_legend(order = 3, reverse = TRUE),
         shape = guide_legend(order = 4,
                              override.aes = list(size = 3)),
         fill = guide_legend(order = 5, reverse = TRUE,
                              override.aes = list(shape = 15, 
                                                  size = 6, 
                                                  color = c("#56B4E9","#D55E00"),
                                                  alpha = 1)),
         linetype = guide_legend(order = 1, reverse = TRUE),
         alpha = guide_legend(order = 2))+
  theme(axis.title = element_text(size = 14, face = "bold"),
        axis.text = element_text(size = 12, face = "bold"),
        legend.title = element_text(size = 12, face = "bold"),
        legend.text = element_text(size = 12, face = "bold"),
        legend.key.width = unit(3,"line"))

altfig2b <- ggplot(data = lrexbetareg, aes(x = sigma, y = RelBias_dz))+ 
  geom_jitter(aes(shape = as.factor(logbase), size = medlist, fill = as.character(nobs)),
              color = "black"
              # , alpha = 0.5
              )+
  geom_line(data = lrexbetareg[rel == "log"], 
            stat = 'smooth',
            method = 'lm',
              formula = y~poly(x,2,raw=T),
              aes(alpha = rsqcat,
                  color = as.character(nobs)),
              size = 1.1) +
  scale_x_continuous(name = "Sigma",breaks = c(0.25,0.45,0.65,0.85))+
  scale_y_continuous(name = "Relative Bias Value (Dzierlenga Method)", lim = c(-1.25,1.25))+
  scale_color_manual(name = "Number of Observations",
                     values = c("#D55E00","#56B4E9"),
                     guide = "none"#guide_legend(reverse = TRUE)
                     )+
  scale_alpha_manual(name = "Rsquared Value",
                     values = c(0.33,0.67,1))+
  scale_fill_manual(name = "Number of Observations\n(+ Fitted Curves)",
                    values = alpha(c("#D55E00","#56B4E9"),c(1,1)))+
  scale_shape_manual(name = "Logarithm Base",
                       breaks = c(2,"e",10),
                       labels = c("2","e","10"),
                       # values = c(15,18,20)
                       values = c(21,22,24))+
  scale_size(range = c(1,4),name = "Median Value",
             guide = guide_legend(reverse = TRUE)) +
  geom_vline(xintercept = sigma)+
  theme_bw()+
  guides(size = guide_legend(order = 1, reverse = TRUE),
         shape = guide_legend(order = 2,
                              override.aes = list(size = 3)),
         fill = guide_legend(order = 3,
                             reverse = TRUE,
                             override.aes = list(color = c("#56B4E9","#D55E00"),
                                                 alpha = 1,
                                                 size = 6,
                                                 shape = 15)))+
  theme(axis.title = element_text(size = 14, face = "bold"), 
        axis.text = element_text(size = 12, face = "bold"),
        legend.title = element_text(size = 12, face = "bold"),
        legend.text = element_text(size = 12, face = "bold"),
        legend.key.width = unit(3,"line"))

altfig2c <- ggplot(data = lrexbetareg, aes(x = sigma, y = RelBias_alt))+
  geom_jitter(aes(shape = as.factor(logbase), size = medlist
                  , fill = as.character(nobs)
                  )
              , color = "black"
              # , alpha = 0.6
              )+
  geom_line(data = lrexbetareg, 
            stat = 'smooth',
            method = 'lm',
              formula = y~poly(x,2,raw=T),
              aes(linetype = logbase,
                  shape = logbase,
                  alpha = rsqcat,
                  color = as.character(nobs)),
              size = 1.1) +
  scale_x_continuous(name = "Sigma",breaks = c(0.25,0.45,0.65,0.85))+
  scale_y_continuous(name = "Relative Bias Value (Alternative Method)", lim = c(-1.25,1.25))+
  scale_color_manual(name = "Number of Observations",
                     values = c("#D55E00","#56B4E9"),
                     guide = "none"
                     )+
  scale_fill_manual(name = "Number of Observations\n(+ Fitted Curves)",
                    values = alpha(c("#D55E00","#56B4E9"),c(1,1)))+
  scale_shape_manual(name = "Logarithm Base",
                       breaks = c(2,"e",10),
                       labels = c("2","e","10"),
                       # values = c(15,18,20)
                       values = c(21,22,24)
                     )+
  scale_alpha_manual(name = "Rsquared Value",
                     values = c(0.33,0.67,1))+
  scale_linetype_manual(name = "Fitted Curves for Logarithm Base",
                       breaks = c(10,"e",2),
                       labels = c("10","e","2"),
                       values = c(3,2,1))+
  scale_size(range = c(1,4),name = "Median Value",
             guide = guide_legend(reverse = TRUE)) +
  geom_vline(xintercept = sigma)+
  theme_bw()+
  guides(size = guide_legend(order = 1, reverse = TRUE),
         shape = guide_legend(order = 2,
                              override.aes = list(size = 3)),
         # color = guide_legend(order = 4, reverse = TRUE,
         #                      override.aes = list(color = c("#D55E00","#56B4E9"),
         #                                          size = 3)),
         fill = guide_legend(order = 5,
                             reverse = TRUE,
                             override.aes = list(color = c("#56B4E9","#D55E00"),
                                                 alpha = 1,
                                                 size = 6,
                                                 shape = 15)),
         linetype = guide_legend(order = 3, reverse = TRUE, 
                                 override.aes = list(color = "black")
                                 ),
         alpha = guide_legend(order = 4))+
  theme(axis.title = element_text(size = 14, face = "bold"),
        axis.text = element_text(size = 12, face = "bold"),
        legend.title = element_text(size = 12, face = "bold"),
        legend.text = element_text(size = 12, face = "bold"),
        legend.key.width = unit(3,"line"))
```

# Table 2

For the full nsim\*nscenario dataset, calculate the relative bias and
coverage probability for each individual simulation.


```
allsimdata$RelBias_rb1 <- (allsimdata$rb-allsimdata$observed_beta)/abs(allsimdata$observed_beta)
allsimdata$RelBias_dz1 <- (allsimdata$dz-allsimdata$observed_beta)/abs(allsimdata$observed_beta)
allsimdata$RelBias_alt1 <- (allsimdata$alt-allsimdata$observed_beta)/abs(allsimdata$observed_beta)

allsimdata$coverage_prob_rb <- allsimdata$coverage_prob_dz <- allsimdata$coverage_prob_alt <- 0
allsimdata$coverage_prob_rb <- c(0,1)[(allsimdata$observed_beta <= allsimdata$rbu & allsimdata$observed_beta >= allsimdata$rbl)+1]
allsimdata$coverage_prob_dz <- c(0,1)[(allsimdata$observed_beta <= allsimdata$dzu & allsimdata$observed_beta >= allsimdata$dzl)+1]
allsimdata$coverage_prob_alt <- c(0,1)[(allsimdata$observed_beta <= allsimdata$altu & allsimdata$observed_beta >= allsimdata$altl)+1]
```


Then Table 2 can be generated


```
tab2 <- data.frame("Estimator" = c("\u03b2~RB~",
                                   "\u03b2~Dz~",
                                   "\u03b2~Alt~"),
                   "Average absolute relative bias" = c(mean(abs(allsimdata$RelBias_rb1),na.rm = T),
                                               mean(abs(allsimdata$RelBias_dz1),na.rm = T),
                                               mean(abs(allsimdata$RelBias_alt1),na.rm = T)),
                   "First Quartile absolute relative bias" = c(summary(abs(allsimdata$RelBias_rb1))[[2]],
                                               summary(abs(allsimdata$RelBias_dz1))[[2]],
                                               summary(abs(allsimdata$RelBias_alt1))[[2]]),
                   "Third Quartile absolute relative bias" = c(summary(abs(allsimdata$RelBias_rb1))[[5]],
                                               summary(abs(allsimdata$RelBias_dz1))[[5]],
                                               summary(abs(allsimdata$RelBias_alt1))[[5]]),
                   "Average coverage probability" = c(mean(allsimdata$coverage_prob_rb,na.rm = T),
                                                      mean(allsimdata$coverage_prob_dz,na.rm = T),
                                                      mean(allsimdata$coverage_prob_alt,na.rm = T)))
names(tab2) <- c("Estimator","Average absolute relative bias","First quartile absolute relative bias","Third Quartile absolute relative bias","Average coverage probability")

tab2 <- knitr::kable(tab2)

tab2
```


| Estimator | Average absolute relative bias | First quartile absolute relative bias | Third Quartile absolute relative bias | Average coverage probability |
| --- | --- | --- | --- | --- |
| βRB | 0.3619204 | 0.1201265 | 0.4989599 | 0.3651146 |
| βDz | 0.4302710 | 0.1117345 | 0.5973034 | 0.3942213 |
| βAlt | 0.3894790 | 0.0901077 | 0.5580900 | 0.4551245 |

# Table 3


```
tab2n <- allsimdata[allsimdata$nobs == 162 & allsimdata$beta == 1 & (allsimdata$sigma == 0.45 | allsimdata$sigma == 0.85) & (allsimdata$medlist == 2 | allsimdata$medlist == 16) & (allsimdata$logbase == 2 | allsimdata$logbase == 10)]
tab2n <- dplyr::select(tab2n,sigma,medlist,logbase,RelBias_rb,RelBias_dz,RelBias_alt, coverage_prob_rb, coverage_prob_dz, coverage_prob_alt)
tab2n <- aggregate(.~sigma+medlist+logbase, data = tab2n, mean)
tab2n <- tab2n[order(tab2n$sigma, tab2n$medlist, tab2n$logbase),]
tab2n1 <- pivot_longer(tab2n, 
                      cols = c("coverage_prob_rb", "coverage_prob_dz", "coverage_prob_alt"), 
                      names_to = "var2",
                      values_to = "coverage_prob")
tab2n <- pivot_longer(tab2n, 
                      cols = c("RelBias_rb","RelBias_dz","RelBias_alt"), 
                      names_to = "rexmethod",
                      values_to = "RelBias")
tab2n$coverage_prob <- tab2n1$coverage_prob
tab2n$rexmethod[str_detect(tab2n$rexmethod,"rb")] <- "rb"
tab2n$rexmethod[str_detect(tab2n$rexmethod,"dz")] <- "dz"
tab2n$rexmethod[str_detect(tab2n$rexmethod,"alt")] <- "alt"
tab2n[,4:6] <- NULL
tab2n <- tab2n %>% mutate_at(c("RelBias","coverage_prob"),round, digits = 3)
tab2n$coverage_prob <- tab2n$coverage_prob * 100
tab2n
```

# Table 4

We’re first plugging in estimates for medians and IQRs for studies
that don’t report them. While the Dzierlenga method doesn’t explicitly
need these values, for the sake of comparison across methods, it will
also use these estimated values for Table 3. Additionally in this step,
means are estimated from medians when the former aren’t available but
the latter are.


```
#Add medians and IQRs
data[data$study_id == "Stein 2016",4:6] <- list(22.2, 15.35, 30.8)
data[data$study_id == "Pilkerton 2018",4:6] <- list(4.3, 3, 6.3)
data[data$study_id == "Hamm 2010",4:6] <- list(7.8, 5.7, 10.7)
data[data$study_id == "Chen 2012",4:6] <- list(5.94, 3.94, 8.94)
data[data$study_id == "Steenland 2009",4:6] <- list(20.2, 13.6, 29.3)

#Calculate mean and SD from median
data$exp_mean[is.na(data$exp_mean) & !is.na(data$exp_med)] <- 
  (data$exp_1q[is.na(data$exp_mean) & !is.na(data$exp_med)] + 
     data$exp_med[is.na(data$exp_mean) & !is.na(data$exp_med)] + 
     data$exp_3q[is.na(data$exp_mean) & !is.na(data$exp_med)]) / 3
```


First we run the Rodriguez-Barranco method using model A
(linear-linear) for linear-base data (relative x) and model B
(log-linear) for log-base data (absolute x). Absolute y is always used.
The absolute change (c) is 1 whereas the relative change (k) is
equivalent to the log base being used.


```
rbout <- data.frame("study" = data$study_id, "input_base" = data$input_base, "actual_beta" = data$manual_beta, "actual_blci" = data$manual_blci, "actual_buci" = data$manual_buci, "beta" = c(1:nrow(data)), "beta_lci" = c(1:nrow(data)), "beta_uci" = c(1:nrow(data)), "logbase" = data$logbase, "nobs" = data$nobs, "sigma" = data$sigma, "medlist" = data$medlist)
#Here we're defining the log-base exposures as absolute expressions and the linear as relative expressions
x.exp <- ifelse(data$input_base == "log", "abs", "rel")
for(i in 1:nrow(data)){
  rbout[i,6:8] <- rbexp(model = data$rb_model[i],
                        y.exp = "abs",
                        x.exp = x.exp[i],
                        beta = data$beta_mean[i],
                        se = (data$beta_uci[i]-data$beta_lci[i])/3.92,
                        mean.x = data$exp_mean[i], #data$exp_med[i], changed 12/13/22
                        mean.y = 0,
                        a = exp(1),
                        b = data$logbase[i],
                        k = data$logbase[i],
                        c = 1)
}
rbout$method <- "Rodriguez-Barranco Method"
```


Next we run the “Alternative” method. This one is relatively
straightforward, and the values can just be input directly from the data
frame.


```
altout <- data.frame("study" = data$study_id, "input_base" = data$input_base, "actual_beta" = data$manual_beta, "actual_blci" = data$manual_blci, "actual_buci" = data$manual_buci, "beta" = c(1:nrow(data)), "beta_lci" = c(1:nrow(data)), "beta_uci" = c(1:nrow(data)), "logbase" = data$logbase, "nobs" = data$nobs, "sigma" = data$sigma, "medlist" = data$medlist)
for(i in 1:nrow(data)){
  altout[i,6:8] <- altexp(input.base = data$input_base[i],
                          median = data$exp_med[i],
                          log.base = data$logbase[i],
                          beta.dist = c(data$beta_mean[i], data$beta_lci[i], data$beta_uci[i]))
}
altout$method <- "Alternative Method"
```


Finally, we’ll replicate the code used above for Table 3 with the
Dzierlenga method, but this time using the estimated medians for
consistency across methods.


```
dzout <- data.frame("study" = data$study_id, "input_base" = data$input_base, "actual_beta" = data$manual_beta, "actual_blci" = data$manual_blci, "actual_buci" = data$manual_buci, "beta" = c(1:nrow(data)), "beta_lci" = c(1:nrow(data)), "beta_uci" = c(1:nrow(data)), "logbase" = data$logbase, "nobs" = data$nobs, "sigma" = data$sigma, "medlist" = data$medlist)
for(i in 1:nrow(data)){
  dzout[i,6:8] <- dzexp(input.base = data$input_base[i], 
                        exp.dist = c(med = data$exp_med[i], iqr1 = data$exp_1q[i], iqr3 = data$exp_3q[i],
                                     gmean = data$exp_gmean[i], gsd = data$exp_gsd[i],
                                     mean = data$exp_mean[i], sd = data$exp_sd[i]),
                        beta.dist = c(data$beta_mean[i], data$beta_lci[i], data$beta_uci[i]),
                        log.base = data$logbase[i])
}
dzout$method <- "Dzierlenga Method"
```


Finally, the outputted datasets for each method along with their
re-expressed values are combined and the table is formatted.


```
dfout <- rbind(rbout, altout, dzout)
dfout$prop_diff_beta <- (dfout$beta/dfout$actual_beta)-1

tab3 <- merge(rbout, altout, by = c("study", "input_base","logbase","nobs","sigma","medlist", "actual_beta", "actual_blci", "actual_buci"))
tab3 <- merge(tab3, dzout, by = c("study", "input_base","logbase","nobs","sigma","medlist", "actual_beta", "actual_blci", "actual_buci"))
tab3$p_d_beta.x <- round((tab3$beta.x/tab3$actual_beta)-1,2)
tab3$p_d_beta.y <- round((tab3$beta.y/tab3$actual_beta)-1,2)
tab3$p_d_beta <- round((tab3$beta/tab3$actual_beta)-1,2)

tab3$alt_beta_lci <- altout$beta_lci
tab3$alt_beta_uci <- altout$beta_uci

tab3 <- tab3 %>% mutate(across(c(actual_beta, beta.x, beta.y, beta), signif, 4))
```


```
Warning: There was 1 warning in `mutate()`.
ℹ In argument: `across(c(actual_beta, beta.x, beta.y, beta), signif, 4)`.
Caused by warning:
! The `...` argument of `across()` is deprecated as of dplyr 1.1.0.
Supply arguments directly to `.fns` through an anonymous function instead.

  # Previously
  across(a:b, mean, na.rm = TRUE)

  # Now
  across(a:b, \(x) mean(x, na.rm = TRUE))
This warning is displayed once every 8 hours.
Call `lifecycle::last_lifecycle_warnings()` to see where this warning was generated.
```


```
tab3 <- tab3 %>% mutate(across(c(p_d_beta.x, p_d_beta.y, p_d_beta), round, 2))
tab3 <- tab3[order(tab3$input_base,decreasing = T),]
#tab3 <- tab3 %>% mutate(across(everything(), as.character))
tab3 <- tab3[c("study","input_base","logbase","nobs","sigma","medlist","actual_beta", "actual_blci", "actual_buci", "beta.x","p_d_beta.x","alt_beta_lci","beta.y","alt_beta_uci","p_d_beta.y","beta","p_d_beta")]
names(tab3) <- c("Study", "Input_Base","logbase","nobs","sigma","medlist","Observed_Beta", "Beta_Lower", "Beta_Upper","RB_Beta","delta_RB_Beta","alt_beta_lci","Alt_Beta","alt_beta_uci","delta_Alt_Beta","Dz_Beta","delta_Dz_Beta")

tab3 <- as.data.table(tab3)
tab3 <- tab3[tab3$Input_Base == "log",]
tab3$logbase[tab3$logbase == 2.718281828] <- "e"
tab3$sigma <- as.numeric(tab3$sigma)
tab3$nobs <- as.numeric(tab3$nobs)
tab3$medlist <- as.numeric(tab3$medlist)
tab4 <- tab3 #Set up next table
tab3 <- dplyr::select(tab3,Study,Observed_Beta,RB_Beta,delta_RB_Beta,Dz_Beta,delta_Dz_Beta,Alt_Beta,delta_Alt_Beta)
tab3 <- tab3 %>% mutate_at(c("Observed_Beta","RB_Beta","delta_RB_Beta","Dz_Beta","delta_Dz_Beta","Alt_Beta","delta_Alt_Beta"),signif, digits = 3)
tab3 <- tab3 %>% mutate_if(is.numeric, as.character)
tab3
```

# Table 4B

This is going to be one long code chunk to generate Table 3B since it
will just be replicating the analysis for Table 3A but with different
input data


```
rbout <- data.frame("study" = datab$study_id, "input_base" = datab$input_base, "actual_beta" = datab$manual_beta, "actual_blci" = datab$manual_blci, "actual_buci" = datab$manual_buci, "beta" = c(1:nrow(datab)), "beta_lci" = c(1:nrow(datab)), "beta_uci" = c(1:nrow(datab)), "logbase" = datab$logbase, "nobs" = datab$nobs, "sigma" = datab$sigma, "medlist" = datab$medlist)
#Here we're defining the log-base exposures as absolute expressions and the linear as relative expressions
x.exp <- ifelse(datab$input_base == "log", "abs", "rel")
for(i in 1:nrow(datab)){
  rbout[i,6:8] <- rbexp(model = datab$rb_model[i],
                        y.exp = "abs",
                        x.exp = x.exp[i],
                        beta = datab$beta_mean[i],
                        se = (datab$beta_uci[i]-datab$beta_lci[i])/3.92,
                        mean.x = datab$exp_mean[i], #datab$exp_med[i], changed 12/13/22
                        mean.y = 0,
                        a = exp(1),
                        b = datab$logbase[i],
                        k = datab$logbase[i],
                        c = 1)
}
rbout$method <- "Rodriguez-Barranco Method" 

altout <- data.frame("study" = datab$study_id, "input_base" = datab$input_base, "actual_beta" = datab$manual_beta, "actual_blci" = datab$manual_blci, "actual_buci" = datab$manual_buci, "beta" = c(1:nrow(datab)), "beta_lci" = c(1:nrow(datab)), "beta_uci" = c(1:nrow(datab)), "logbase" = datab$logbase, "nobs" = datab$nobs, "sigma" = datab$sigma, "medlist" = datab$medlist)
for(i in 1:nrow(datab)){
  altout[i,6:8] <- altexp(input.base = datab$input_base[i],
                          median = datab$exp_med[i],
                          log.base = datab$logbase[i],
                          beta.dist = c(datab$beta_mean[i], datab$beta_lci[i], datab$beta_uci[i]))
}
altout$method <- "Alternative Method"

dzout <- data.frame("study" = datab$study_id, "input_base" = datab$input_base, "actual_beta" = datab$manual_beta, "actual_blci" = datab$manual_blci, "actual_buci" = datab$manual_buci, "beta" = c(1:nrow(datab)), "beta_lci" = c(1:nrow(datab)), "beta_uci" = c(1:nrow(datab)), "logbase" = datab$logbase, "nobs" = datab$nobs, "sigma" = datab$sigma, "medlist" = datab$medlist)
for(i in 1:nrow(datab)){
  dzout[i,6:8] <- dzexp(input.base = datab$input_base[i], 
                        exp.dist = c(med = datab$exp_med[i], iqr1 = datab$exp_1q[i], iqr3 = datab$exp_3q[i],
                                     gmean = datab$exp_gmean[i], gsd = datab$exp_gsd[i],
                                     mean = datab$exp_mean[i], sd = datab$exp_sd[i]),
                        beta.dist = c(datab$beta_mean[i], datab$beta_lci[i], datab$beta_uci[i]),
                        log.base = datab$logbase[i])
}
dzout$method <- "Dzierlenga Method"

dfout <- rbind(rbout, altout, dzout)
dfout$prop_diff_beta <- (dfout$beta/dfout$actual_beta)-1

tab3b <- merge(rbout, altout, by = c("study", "input_base","logbase","nobs","sigma","medlist", "actual_beta", "actual_blci", "actual_buci"))
tab3b <- merge(tab3b, dzout, by = c("study", "input_base","logbase","nobs","sigma","medlist", "actual_beta", "actual_blci", "actual_buci"))
tab3b$p_d_beta.x <- round((tab3b$beta.x/tab3b$actual_beta)-1,2)
tab3b$p_d_beta.y <- round((tab3b$beta.y/tab3b$actual_beta)-1,2)
tab3b$p_d_beta <- round((tab3b$beta/tab3b$actual_beta)-1,2)

tab3b$alt_beta_lci <- altout$beta_lci
tab3b$alt_beta_uci <- altout$beta_uci

tab3b <- tab3b %>% mutate(across(c(actual_beta, beta.x, beta.y, beta), signif, 4))
tab3b <- tab3b %>% mutate(across(c(p_d_beta.x, p_d_beta.y, p_d_beta), round, 2))
tab3b <- tab3b[order(tab3b$input_base,decreasing = T),]
#tab3b <- tab3b %>% mutate(across(everything(), as.character))
tab3b <- tab3b[c("study","input_base","logbase","nobs","sigma","medlist","actual_beta", "actual_blci", "actual_buci", "beta.x","p_d_beta.x","alt_beta_lci","beta.y","alt_beta_uci","p_d_beta.y","beta","p_d_beta")]
names(tab3b) <- c("Study", "Input_Base","logbase","nobs","sigma","medlist","Observed_Beta", "Beta_Lower", "Beta_Upper","RB_Beta","delta_RB_Beta","alt_beta_lci","Alt_Beta","alt_beta_uci","delta_Alt_Beta","Dz_Beta","delta_Dz_Beta")

tab3b <- as.data.table(tab3b)
tab3b <- tab3b[tab3b$Input_Base == "log",]
tab3b$logbase[tab3b$logbase == 2.718281828] <- "e"
tab3b$sigma <- as.numeric(tab3b$sigma)
tab3b$nobs <- as.numeric(tab3b$nobs)
tab3b$medlist <- as.numeric(tab3b$medlist)
tab4 <- tab3b #Set up next table
tab3b <- dplyr::select(tab3b,Study,Observed_Beta,RB_Beta,delta_RB_Beta,Dz_Beta,delta_Dz_Beta,Alt_Beta,delta_Alt_Beta)
tab3b <- tab3b %>% mutate_at(c("Observed_Beta","RB_Beta","delta_RB_Beta","Dz_Beta","delta_Dz_Beta","Alt_Beta","delta_Alt_Beta"),signif, digits = 3)
tab3b <- tab3b %>% mutate_if(is.numeric, as.character)
summary(as.numeric(tab3b$delta_RB_Beta))
```


```
    Min.  1st Qu.   Median     Mean  3rd Qu.     Max. 
-15.9000  -0.4300  -0.0200  -0.3113   0.3300   7.4900
```


```
summary(as.numeric(tab3b$delta_Dz_Beta))
```


```
   Min. 1st Qu.  Median    Mean 3rd Qu.    Max. 
-17.900  -0.225   0.520   0.554   2.575  10.700
```


```
summary(as.numeric(tab3b$delta_Alt_Beta))
```


```
    Min.  1st Qu.   Median     Mean  3rd Qu.     Max. 
-18.1000  -0.2100   0.6600   0.4513   2.7850   8.8100
```


```
tab3b
```

# Table 5

We will then go through the same process, but adjusting for the OLS
regression with sigma performed in the simulation. This will comprise
Table 5.


```
tab4$RB_Beta_adj <- as.numeric(tab4$RB_Beta)/(1+predict(step.2p6,tab4))
tab4$Alt_Beta_adjl <- as.numeric(tab4$alt_beta_lci)/(1+predict(fit2p5l,tab4))
```


```
Warning: contrasts dropped from factor C(as.factor(logbase), base = 3)
```


```
tab4$Alt_Beta_adj <- as.numeric(tab4$Alt_Beta)/(1+predict(step.2p5,tab4))
```


```
Warning: contrasts dropped from factor C(as.factor(logbase), base = 3)
```


```
tab4$Alt_Beta_adju <- as.numeric(tab4$alt_beta_uci)/(1+predict(fit2p5u,tab4))
```


```
Warning: contrasts dropped from factor C(as.factor(logbase), base = 3)
```


```
tab4$Dz_Beta_adjl <- as.numeric(tab4$dz_beta_lci)/(1+predict(fit2p7l,tab4))
colnames(tab4)[colnames(tab4)=="Observed_Beta"] <- "beta"
tab4$Dz_Beta_adj <- as.numeric(tab4$Dz_Beta)/(1+predict(step.2p7,tab4))
```


```
Warning: contrasts dropped from factor C(as.factor(logbase), base = 3)
```


```
colnames(tab4)[colnames(tab4)=="beta"] <- "Observed_Beta"
tab4$Dz_Beta_adju <- as.numeric(tab4$dz_beta_uci)/(1+predict(fit2p7u,tab4))
tab4$Alt_Beta_adjl[tab4$Alt_Beta_adjl > tab4$Alt_Beta_adj] <- tab4$Alt_Beta_adj[tab4$Alt_Beta_adjl > tab4$Alt_Beta_adj]
tab4$Alt_Beta_adju[tab4$Alt_Beta_adju < tab4$Alt_Beta_adj] <- tab4$Alt_Beta_adj[tab4$Alt_Beta_adju < tab4$Alt_Beta_adj]
tab4$delta_RB_Beta_adj <- round((tab4$RB_Beta_adj/as.numeric(tab4$Observed_Beta))-1,2)
tab4$delta_Alt_Beta_adj <- round((tab4$Alt_Beta_adj/as.numeric(tab4$Observed_Beta))-1,2)
tab4$delta_Dz_Beta_adj <- round((tab4$Dz_Beta_adj/as.numeric(tab4$Observed_Beta))-1,2)

tab4$StudybInput <- paste(tab4$Study,tab4$Input_Base, "base")
tab4[,labloc := pmax(Alt_Beta_adju,Beta_Upper)]
tab4 <- dplyr::select(tab4,Study,Observed_Beta,RB_Beta_adj,delta_RB_Beta_adj,Dz_Beta_adj,delta_Dz_Beta_adj,Alt_Beta_adj,delta_Alt_Beta_adj)
tab4
```


```
NA
```

# Supplemental Figures and Tables

## Figure S1

Similar to Figure 2, but includes betaDGM < 0.


```
lrexbetatab$posbeta <- ifelse(lrexbetatab$beta >0,"Positive","Negative")

figs1c <- ggplot(data = lrexbetatab, aes(x = sigma, y = RelBias_alt))+
  geom_jitter(aes(shape = as.factor(logbase), size = medlist
                  , fill = as.character(nobs)
                  )
              , color = "black"
              # , alpha = 0.6
              )+
  geom_line(data = lrexbetatab[rel == "log"], 
            stat = 'smooth',
            method = 'lm',
              formula = y~poly(x,2,raw=T),
              aes(linetype = logbase,
                  shape = logbase,
                  alpha = posbeta,
                  color = as.character(nobs)),
              size = 1.1) +
  scale_x_continuous(name = "Sigma",breaks = c(0.25,0.45,0.65,0.85))+
  scale_y_continuous(name = "Relative Bias Value (Alternative Method)", lim = c(-1.25,1.25))+
  scale_color_manual(name = "Number of Observations",
                     values = c("#D55E00","#56B4E9"),
                     guide = "none"
                     )+
  scale_fill_manual(name = "Number of Observations\n(+ Fitted Curves)",
                    values = alpha(c("#D55E00","#56B4E9"),c(1,1)))+
  scale_shape_manual(name = "Logarithm Base",
                       breaks = c(2,"e",10),
                       labels = c("2","e","10"),
                       # values = c(15,18,20)
                       values = c(21,22,24)
                     )+
  scale_alpha_manual(name = "Fitted Curves for Sign of Beta",
                     values = c(0.5,1))+
  scale_linetype_manual(name = "Fitted Curves for Logarithm Base",
                       breaks = c(10,"e",2),
                       labels = c("10","e","2"),
                       values = c(3,2,1))+
  scale_size(range = c(1,4),name = "Median Value",
             guide = guide_legend(reverse = TRUE)) +
  geom_vline(xintercept = sigma)+
  theme_bw()+
  guides(size = guide_legend(order = 1, reverse = TRUE),
         shape = guide_legend(order = 2,
                              override.aes = list(size = 3)),
         # color = guide_legend(order = 4, reverse = TRUE,
         #                      override.aes = list(color = c("#D55E00","#56B4E9"),
         #                                          size = 3)),
         fill = guide_legend(order = 5,
                             reverse = TRUE,
                             override.aes = list(color = c("#56B4E9","#D55E00"),
                                                 alpha = 1,
                                                 size = 6,
                                                 shape = 15)),
         linetype = guide_legend(order = 3, reverse = TRUE, 
                                 override.aes = list(color = "black")
                                 ),
         alpha = guide_legend(order = 4))+
  theme(axis.title = element_text(size = 14, face = "bold"),
        axis.text = element_text(size = 12, face = "bold"),
        legend.title = element_text(size = 12, face = "bold"),
        legend.text = element_text(size = 12, face = "bold"),
        legend.key.width = unit(3,"line"))
```


```
Warning: Ignoring unknown aesthetics: shape
```


```
figs1a <- ggplot(data = lrexbetatab, aes(x = sigma, y = RelBias_rb))+
  geom_jitter(aes(shape = as.factor(logbase), size = medlist, fill = as.character(nobs)),
              color = "black"
              # , alpha = 0.5
              )+
 geom_line(data = lrexbetatab[rel == "log"], 
            stat = 'smooth',
            method = 'lm',
              formula = y~poly(x,2,raw=T),
              aes(linetype = as.factor(medlist),
                  alpha = posbeta),
              color = "blue",
           size = 1.1) +
  scale_x_continuous(name = "Sigma",breaks = c(0.25,0.45,0.65,0.85))+
  scale_y_continuous(name = "Relative Bias Value (Rodriguez-Barranco Method)", lim = c(-1.25,1.25))+
  scale_shape_manual(name = "Logarithm Base",
                       breaks = c(2,"e",10),
                       labels = c("2","e","10"),
                       # values = c(15,18,20)
                       values = c(21,22,24)
                     )+
  scale_fill_manual(name = "Number of Observations",
                    values = c("#D55E00","#56B4E9"))+
  scale_linetype_discrete(name = "Fitted Curves for Median Values")+
    scale_alpha_manual(name = "Fitted Curves for Sign of Beta",
                     values = c(0.5,1))+
  scale_size(range = c(1,4),
             # breaks = c(0.25,0.5,1,2,4,8,16,32),
             name = "Median Value",
             guide = guide_legend(reverse = TRUE)) +
  geom_vline(xintercept = sigma)+
  theme_bw()+
  guides(size = guide_legend(order = 3, reverse = TRUE),
         shape = guide_legend(order = 4,
                              override.aes = list(size = 3)),
         fill = guide_legend(order = 5, reverse = TRUE,
                              override.aes = list(shape = 15, 
                                                  size = 6, 
                                                  color = c("#56B4E9","#D55E00"),
                                                  alpha = 1)),
         linetype = guide_legend(order = 1, reverse = TRUE),
         alpha = guide_legend(order = 2))+
  theme(axis.title = element_text(size = 14, face = "bold"),
        axis.text = element_text(size = 12, face = "bold"),
        legend.title = element_text(size = 12, face = "bold"),
        legend.text = element_text(size = 12, face = "bold"),
        legend.key.width = unit(3,"line"))

figs1b <- ggplot(data = lrexbetatab, aes(x = sigma, y = RelBias_dz))+ 
  geom_jitter(aes(shape = as.factor(logbase), size = medlist, fill = as.character(nobs)),
              color = "black"
              # , alpha = 0.5
              )+
  geom_line(data = lrexbetatab[rel == "log"], 
            stat = 'smooth',
            method = 'lm',
              formula = y~poly(x,2,raw=T),
              aes(alpha = posbeta,
                  color = as.character(nobs)),
              size = 1.1) +
  scale_x_continuous(name = "Sigma",breaks = c(0.25,0.45,0.65,0.85))+
  scale_y_continuous(name = "Relative Bias Value (Dzierlenga Method)", lim = c(-1.25,1.25))+
  scale_color_manual(name = "Number of Observations",
                     values = c("#D55E00","#56B4E9"),
                     guide = "none"#guide_legend(reverse = TRUE)
                     )+
  scale_alpha_manual(name = "Fitted Curves for Sign of Beta",
                     values = c(0.5,1))+
  scale_fill_manual(name = "Number of Observations\n(+ Fitted Curves)",
                    values = alpha(c("#D55E00","#56B4E9"),c(1,1)))+
  scale_shape_manual(name = "Logarithm Base",
                       breaks = c(2,"e",10),
                       labels = c("2","e","10"),
                       # values = c(15,18,20)
                       values = c(21,22,24))+
  scale_size(range = c(1,4),name = "Median Value",
             guide = guide_legend(reverse = TRUE)) +
  geom_vline(xintercept = sigma)+
  theme_bw()+
  guides(size = guide_legend(order = 1, reverse = TRUE),
         shape = guide_legend(order = 2,
                              override.aes = list(size = 3)),
         fill = guide_legend(order = 3,
                             reverse = TRUE,
                             override.aes = list(color = c("#56B4E9","#D55E00"),
                                                 alpha = 1,
                                                 size = 6,
                                                 shape = 15)))+
  theme(axis.title = element_text(size = 14, face = "bold"), 
        axis.text = element_text(size = 12, face = "bold"),
        legend.title = element_text(size = 12, face = "bold"),
        legend.text = element_text(size = 12, face = "bold"),
        legend.key.width = unit(3,"line"))

ggarrange(figs1a,figs1b,figs1c)
```


```
# Creates figure sy-- Uncomment to generate
# voidplt <- ggplot() + theme_void()
# pdf("figuresy.pdf",width = 15, height = 8)
# ggpubr::ggarrange(figs1a,figs1b, labels = c("A","B"), align = "v")
# ggpubr::ggarrange(figs1c,voidplt, labels = c("C",""), align = "v")
# dev.off()
```

## Table S1


```
tabs1 <- data.table(method = "Rodriguez-Barranco Method", 
                           parameter = names(step.2p6$coefficients[!is.na(step.2p6$coefficients)]), 
                           coefficient = step.2p6$coefficients[!is.na(step.2p6$coefficients)], 
                           se = summary(step.2p6)$coefficients[,2], 
                           p = summary(step.2p6)$coefficients[,4],
                           rmse = sqrt(mean(step.2p6$residuals^2)),
                           r2 = summary(step.2p6)$adj.r.squared)

tabs1 <- rbind(tabs1,
                data.table(method = "Dzierlenga Method", 
                           parameter = names(step.2p7$coefficients[!is.na(step.2p7$coefficients)]), 
                           coefficient = step.2p7$coefficients[!is.na(step.2p7$coefficients)], 
                           se = summary(step.2p7)$coefficients[,2], 
                           p = summary(step.2p7)$coefficients[,4],
                           rmse = sqrt(mean(step.2p7$residuals^2)),
                           r2 = summary(step.2p7)$adj.r.squared),
               data.table(method = "Alternative Method", 
                           parameter = names(step.2p5$coefficients[!is.na(step.2p5$coefficients)]), 
                           coefficient = step.2p5$coefficients[!is.na(step.2p5$coefficients)], 
                           se = summary(step.2p5)$coefficients[,2], 
                           p = summary(step.2p5)$coefficients[,4],
                           rmse = sqrt(mean(step.2p5$residuals^2)),
                           r2 = summary(step.2p5)$adj.r.squared))

# tabs1 <- knitr::kable(tabs1) #For a slightly different format

tabs1
```

## Table S2


```
#Creating Table comparing R^2 for 768 vs. 768*2000
tabs2 <- data.frame("rsq" = c("n = 768",
                                    "n = 768 * 2000"),
                   "rb" = c(summary(step.2p6)$adj.r.squared,
                                   summary(step.2p6f)$adj.r.squared),
                   "dz" = c(summary(step.2p7)$adj.r.squared,
                                   summary(step.2p7f)$adj.r.squared),
                   "alt" =c(summary(step.2p5)$adj.r.squared,
                                   summary(step.2p5f)$adj.r.squared))
names(tabs2) <- c("r^2^ for:","\u03b2~RB~","\u03b2~Dz~","\u03b2~Alt~")

tabs2 <- knitr::kable(tabs2)

tabs2
```


| r2 for: | βRB | βDz | βAlt |
| --- | --- | --- | --- |
| n = 768 | 0.7058464 | 0.9929224 | 0.9957947 |
| n = 768 \* 2000 | 0.7087617 | 0.9930568 | 0.9957407 |

## Table S3

Table S3 was generated by hand and so is not produced here.

## Table S4


```
tabs4df <- data[data$input_base == "log",]
tabs4df <- tabs4df[order(tabs4df$study_id),]
tabs4 <- data.frame("Study, Year" = tabs4df$study_id,
                   "Specific Finding/n(Location, Outcome)" =
                     c("Our analysis, Hib IgG",
                       "Table 3 (Fully Adjusted), Birth Weight (g)",
                       "Table 3 (20-49y), HSV 2",
                       "Table 3, Triglycerides (mg/dL)",
                       "Table 3 (Adjusted), Birth Weight (g)",
                       "Table 6 (Adjusted All Births, Per in unit increase), Birth Weight (g)",
                       "Table 5 (Hamm, PFOS), Birth Weight (g)",
                       "Table 2 (Model 2), Infertility",
                       "Figure 1a (MBzP), Asthma",
                       "Table 4, Rubella (%Δ)",
                       "Table 4, Total Cholesterol",
                       "Table 2, Mumps (%Δ)",
                       "Table 5 (Fully Adjusted), Birth Weight (g)",
                       "Table 2 (Model 1), CVD",
                       "Table 4 (Model 2), Triglycerides (mg/dL)"),
                   "Median" = tabs4df$exp_med,
                   "1st Quartile" = tabs4df$exp_1q,
                   "3rd Quartile" = tabs4df$exp_3q,
                   "Sigma (lognormal distribution)" = tabs4df$sigma,
                   "Mean" = tabs4df$exp_mean)
names(tabs4) <- c("Study, Year","Specific Finding (Location, Outcome)","Median","1st Quartile","3rd Quartile", "Sigma (lognormal distribution)","Mean")

tabs4 <- knitr::kable(tabs4)

tabs4
```


| Study, Year | Specific Finding (Location, Outcome) | Median | 1st Quartile | 3rd Quartile | Sigma (lognormal distribution) | Mean |
| --- | --- | --- | --- | --- | --- | --- |
| Abraham 2020 | Our analysis, Hib IgG | 14.300 | 6.700 | 19.30 | 0.78 | 16.8000000 |
| Apelberg 2007 | Table 3 (Fully Adjusted), Birth Weight (g) | 5.000 | 3.400 | 7.90 | 0.62 | 5.4333333 |
| Bulka 2021 | Table 3 (20-49y), HSV 2 | 2.770 | 1.670 | 4.60 | 0.75 | 3.0133333 |
| Cheang 2021 | Table 3, Triglycerides (mg/dL) | 38.700 | 29.400 | 55.20 | 0.47 | 41.1000000 |
| Chen 2012 | Table 3 (Adjusted), Birth Weight (g) | 5.940 | 3.940 | 8.94 | 0.61 | 6.2733333 |
| Darrow 2013 | Table 6 (Adjusted All Births, Per in unit increase), Birth Weight (g) | 13.900 | 9.500 | 19.70 | 0.54 | 14.3666667 |
| Hamm 2010 | Table 5 (Hamm, PFOS), Birth Weight (g) | 7.800 | 5.700 | 10.70 | 0.47 | 8.0666667 |
| Lee 2020 | Table 2 (Model 2), Infertility | 0.240 | 0.140 | 0.43 | 0.83 | 0.2700000 |
| Odebeatu 2019 | Figure 1a (MBzP), Asthma | 12.300 | 5.000 | 27.30 | 1.26 | 14.8666667 |
| Pilkerton 2018 | Table 4, Rubella (%Δ) | 4.300 | 3.000 | 6.30 | 0.55 | 6.0000000 |
| Steenland 2009 | Table 4, Total Cholesterol | 20.200 | 13.600 | 29.30 | 0.57 | 22.4000000 |
| Stein 2016 | Table 2, Mumps (%Δ) | 22.200 | 15.350 | 30.80 | 0.52 | 22.7833333 |
| Washino 2009 | Table 5 (Fully Adjusted), Birth Weight (g) | 5.200 | 3.400 | 7.00 | 0.53 | 5.2000000 |
| Xu 2020a | Table 2 (Model 1), CVD | 0.521 | 0.346 | 1.03 | 0.81 | 0.6323333 |
| Xu 2020b | Table 4 (Model 2), Triglycerides (mg/dL) | 0.521 | 0.346 | 1.03 | 0.81 | 0.6323333 |

## Table S4b


```
tabs4dfb <- datab[datab$input_base == "log",]
tabs4dfb <- tabs4dfb[order(tabs4dfb$study_id),]
tabs4b <- data.frame("Study, Year" = tabs4dfb$study_id,
                   "Specific Finding/n(Location, Outcome)" =
                     c("Our analysis, Diphtheria IgG, PFOA",
                       "Table 3 (Fully Adjusted), Ponderal Index (g cm3 x 100), PFOA",
                       "Table 3 (20-49 y), Toxocara, PFOS",
                       "Table 3, LDL (mg/dL), HbAA+HbGA",
                       "Table 3 (Adjusted), Birth Weight (g), PFOA",
                       "Table 4 (Adjusted First Prospective Births, Per ln unit increase), 
                       Pregnancy Induced Hypertension, PFOS",
                       "P 592, left column, Birth Weight (g), PFHxS",
                       "Table 2 (Model 2), Infertility, Lead",
                       "Figure 3b, Asthma, ∑DEHP (µmol/L)",
                       "Table 4, Rubella (%Δ), PFOS",
                       "Table 4, HDL, PFOA",
                       "Table 2 (All), Mumps (%Δ), PFHxS",
                       "Table 2 (Model 2), CVD, Propanaldehyde",
                       "Table 4 (Model 2), Monocytes, Isopentanaldehyde",
                       "Table 5 (Fully Adjusted), Birth Weight (g), PFOA (From Verner et al. 2015)"),
                   "Median" = tabs4dfb$exp_med,
                   "1st Quartile" = tabs4dfb$exp_1q,
                   "3rd Quartile" = tabs4dfb$exp_3q,
                   "Sigma (lognormal distribution)" = tabs4dfb$sigma,
                   "Mean" = tabs4dfb$exp_mean)
names(tabs4b) <- c("Study, Year","Specific Finding (Location, Outcome)","Median","1st Quartile","3rd Quartile", "Sigma (lognormal distribution)","Mean")

tabs4b <- knitr::kable(tabs4b)

tabs4b
```


| Study, Year | Specific Finding (Location, Outcome) | Median | 1st Quartile | 3rd Quartile | Sigma (lognormal distribution) | Mean |
| --- | --- | --- | --- | --- | --- | --- |
| Abraham 2020 | Our analysis, Diphtheria IgG, PFOA | 14.300 | 6.700 | 19.300 | 0.78 | 16.800 |
| Apelberg 2007 | Table 3 (Fully Adjusted), Ponderal Index (g cm3 x 100), PFOA | 1.600 | 1.200 | 2.100 | 0.41 | 1.630 |
| Bulka 2021 | Table 3 (20-49 y), Toxocara, PFOS | 5.190 | 3.000 | 8.500 | 0.77 | 6.920 |
| Cheang 2021 | Table 3, LDL (mg/dL), HbAA+HbGA | 83.500 | 64.100 | 127.700 | 0.51 | 119.900 |
| Chen 2012 | Table 3 (Adjusted), Birth Weight (g), PFOA | 1.890 | 0.990 | 3.710 | 0.98 | 2.200 |
| Darrow 2013 | Table 4 (Adjusted First Prospective Births, Per ln unit increase), |  |  |  |  |  |
| Pregnancy Induced Hypertension, PFOS | 13.900 | 9.500 | 19.700 | 0.54 | 15.600 |  |
| Hamm 2010 | P 592, left column, Birth Weight (g), PFHxS | 1.100 | 0.510 | 2.280 | 1.11 | 1.300 |
| Lee 2020 | Table 2 (Model 2), Infertility, Lead | 0.530 | 0.350 | 0.725 | 0.54 | 0.687 |
| Odebeatu 2019 | Figure 3b, Asthma, ∑DEHP (µmol/L) | 0.142 | 0.069 | 0.292 | 1.07 | 0.390 |
| Pilkerton 2018 | Table 4, Rubella (%Δ), PFOS | 21.800 | 14.200 | 30.700 | 0.57 | 25.000 |
| Steenland 2009 | Table 4, HDL, PFOA | 26.600 | 9.200 | 70.900 | 1.51 | 80.300 |
| Stein 2016 | Table 2 (All), Mumps (%Δ), PFHxS | 22.200 | 15.350 | 30.800 | 0.52 | 22.800 |
| Washino 2009 | Table 2 (Model 2), CVD, Propanaldehyde | 1.300 | 0.800 | 1.800 | 0.60 | 1.400 |
| Xu 2020a | Table 4 (Model 2), Monocytes, Isopentanaldehyde | 2.090 | 1.570 | 2.670 | 0.39 | 2.210 |
| Xu 2020b | Table 5 (Fully Adjusted), Birth Weight (g), PFOA (From Verner et al. 2015) | 0.524 | 0.343 | 1.060 | 0.84 | 0.775 |

## Table S5

Table S5 was generated by hand and so is not produced here.

## Table S6

Finally, we investigated the effects of a few very influential points
in 4 studies and whether dropping those points made any of the methods
more accurate in their re-expression of Beta values. The first step in
this part of the analysis is to read in the data files for the studies
of interest and set a couple options for the survey design (weighting,
etc.) and linear regression.


```
df1 <- read.csv(file = "data/Odebeatu_2019.csv")
df2 <- read.csv(file = "data/Pilkerton_2018.csv")
df3 <- read.csv(file = "data/Cheang_2021v2.csv")
df4 <- read.csv(file = "data/Xu_2020.csv")
options(survey.adjust.domain.lonely=TRUE)
options(survey.lonely.psu="adjust")
```


We then assign a survey design to each study using the “survey”
package and the included ids (SDMVPSU), weights, (wgt, wgt3) and strata
(SDMVSTRA).


```
ode <- svydesign(id = ~SDMVPSU, weights = ~wgt, strata = ~SDMVSTRA, nest = TRUE, data = df1)
pil <- svydesign(id = ~SDMVPSU, weights = ~wgt3, strata = ~SDMVSTRA, nest = TRUE, data = df2)
che <- svydesign(id = ~SDMVPSU, weights = ~wgt, strata = ~SDMVSTRA, nest = TRUE, data = df3)
xu <- svydesign(id = ~SDMVPSU, weights = ~wgt, strata = ~SDMVSTRA, nest = TRUE, data = df4)
```


Next, we create an empty list for the generalized linear models
(glm), and run a multiple linear regression for each scenario (4 studies
w/ log to linear re-expression + 4 studies with linear to log
re-expression), using the appropriate explanatory variables as noted in
the original studies.


```
glm <- list()
glm[[1]] <- svyglm(asthma~MBzP, family = quasibinomial, design = ode)
glm[[2]] <- svyglm(asthma~l10MBzP, family = quasibinomial, design = ode)
glm[[3]] <- svyglm(logrubella~pfoa + RIAGENDR + RIDAGEYR + ed1 + ed2 + BMXBMI + eth1 + eth2, design = pil)
glm[[4]] <- svyglm(logrubella~lg2pfoa + RIAGENDR + RIDAGEYR + ed1 + ed2 + BMXBMI + eth1 + eth2, design = pil)
glm[[5]] <- svyglm(LBXTR~HBga + female + RIDAGEYR + INDFMPIR + smk1 + smk2 + diabetes_rev + hyper_rev + energy + eth1 + eth2 + eth3 + eth4 + edu1 + edu2 + edu3 + edu5 + bx1 + bx2 + paqnone + paqmod + alcohol, design = che)
glm[[6]] <- svyglm(LBXTR~l2ga + female + RIDAGEYR + INDFMPIR + smk1 + smk2 + diabetes_rev + hyper_rev + energy + eth1 + eth2 + eth3 + eth4 + edu1 + edu2 + edu3 + edu5 + bx1 + bx2 + paqnone + paqmod + alcohol, design = che)
glm[[7]] <- svyglm(cvd~LBXI5AL + RIDAGEYR + eth1 + eth2 + eth4 + eth5 + edu2 + edu3 + edu4 + edu5 + female, family = quasibinomial, design = xu)
glm[[8]] <- svyglm(cvd~l2i5al + RIDAGEYR + eth1 + eth2 + eth4 + eth5 + edu2 + edu3 + edu4 + edu5 + female, family = quasibinomial, design = xu)
```


Then, using the dfbetas function, we identify the influence of each
data point on the multi-linear regression for each scenario. Points are
considered influential (and are therefore removed from the data subset)
if dfbeta > 2/sqrt(n).


```
dfb <- list(df1, df1, df2, df2, df3, df3, df4, df4)
dfsub <- list()
for(i in 1:length(glm)){
  dfb[[i]]$dfbetas <- 0
  dfb[[i]][complete.cases(dfb[[i]]),]$dfbetas <- dfbetas(glm[[i]])[,2]
  dfsub[[i]] <- dfb[[i]][abs(dfb[[i]]$dfbetas) <= 2/sqrt(nrow(dfb[[i]])),]
}
```


Now we re-create the survey designs…


```
odesub <- svydesign(id = ~SDMVPSU, weights = ~wgt, strata = ~SDMVSTRA, nest = TRUE, data = dfsub[[1]])
odesubl <- svydesign(id = ~SDMVPSU, weights = ~wgt, strata = ~SDMVSTRA, nest = TRUE, data = dfsub[[2]])
pilsub <- svydesign(id = ~SDMVPSU, weights = ~wgt3, strata = ~SDMVSTRA, nest = TRUE, data = dfsub[[3]])
pilsubl <- svydesign(id = ~SDMVPSU, weights = ~wgt3, strata = ~SDMVSTRA, nest = TRUE, data = dfsub[[4]])
chesub <- svydesign(id = ~SDMVPSU, weights = ~wgt, strata = ~SDMVSTRA, nest = TRUE, data = dfsub[[5]])
chesubl <- svydesign(id = ~SDMVPSU, weights = ~wgt, strata = ~SDMVSTRA, nest = TRUE, data = dfsub[[6]])
xusub <- svydesign(id = ~SDMVPSU, weights = ~wgt, strata = ~SDMVSTRA, nest = TRUE, data = dfsub[[7]])
xusubl <- svydesign(id = ~SDMVPSU, weights = ~wgt, strata = ~SDMVSTRA, nest = TRUE, data = dfsub[[8]])
```


and re-run the multi-linear regressions on the subset of each dataset
without the influential points.


```
glmsub <- list()
glmsub[[1]] <- svyglm(asthma~MBzP, family = quasibinomial, design = odesub)
glmsub[[2]] <- svyglm(asthma~l10MBzP, family = quasibinomial, design = odesubl)
glmsub[[3]] <- svyglm(logrubella~pfoa + RIAGENDR + RIDAGEYR + ed1 + ed2 + BMXBMI + eth1 + eth2, design = pilsub)
glmsub[[4]] <- svyglm(logrubella~lg2pfoa + RIAGENDR + RIDAGEYR + ed1 + ed2 + BMXBMI + eth1 + eth2, design = pilsubl)
glmsub[[5]] <- svyglm(LBXTR~HBga + female + RIDAGEYR + INDFMPIR + smk1 + smk2 + diabetes_rev + hyper_rev + energy + eth1 + eth2 + eth3 + eth4 + edu1 + edu2 + edu3 + edu5 + bx1 + bx2 + paqnone + paqmod + alcohol, design = chesub)
glmsub[[6]] <- svyglm(LBXTR~l2ga + female + RIDAGEYR + INDFMPIR + smk1 + smk2 + diabetes_rev + hyper_rev + energy + eth1 + eth2 + eth3 + eth4 + edu1 + edu2 + edu3 + edu5 + bx1 + bx2 + paqnone + paqmod + alcohol, design = chesubl)
glmsub[[7]] <- svyglm(cvd~LBXI5AL + RIDAGEYR + eth1 + eth2 + eth4 + eth5 + edu2 + edu3 + edu4 + edu5 + female, family = quasibinomial, design = xusub)
glmsub[[8]] <- svyglm(cvd~l2i5al + RIDAGEYR + eth1 + eth2 + eth4 + eth5 + edu2 + edu3 + edu4 + edu5 + female, family = quasibinomial, design = xusubl)
```


Now we want to change the beta values and the values of x-variable
central tendency and spread for our adjusted dataset with the
influential points removed. First, the original data frame is cloned
into dataadj and then the new beta values for the studies with
influential points removed are substituted in.


```
dataadj <- read.csv(file = "data/Re_expression_Full_Table_11072022.csv")

dataadj[dataadj$study_id == "Odebeatu 2019" & dataadj$input_base == "lin",11:13] <-
  c(summary(glmsub[[1]])$coef[2,1], confint(glmsub[[1]])[2,])
dataadj[dataadj$study_id == "Odebeatu 2019" & dataadj$input_base == "log",11:13] <-
  c(summary(glmsub[[2]])$coef[2,1], confint(glmsub[[2]])[2,])
dataadj[dataadj$study_id == "Pilkerton 2018" & dataadj$input_base == "lin",11:13] <-
  c(summary(glmsub[[3]])$coef[2,1], confint(glmsub[[3]])[2,])
dataadj[dataadj$study_id == "Pilkerton 2018" & dataadj$input_base == "log",11:13] <-
  c(summary(glmsub[[4]])$coef[2,1], confint(glmsub[[4]])[2,])
dataadj[dataadj$study_id == "Cheang 2021" & dataadj$input_base == "lin",11:13] <-
  c(summary(glmsub[[5]])$coef[2,1], confint(glmsub[[5]])[2,])
dataadj[dataadj$study_id == "Cheang 2021" & dataadj$input_base == "log",11:13] <-
  c(summary(glmsub[[6]])$coef[2,1], confint(glmsub[[6]])[2,])
dataadj[dataadj$study_id == "Xu 2020a" & dataadj$input_base == "lin",11:13] <-
  c(summary(glmsub[[7]])$coef[2,1], confint(glmsub[[7]])[2,])
dataadj[dataadj$study_id == "Xu 2020a" & dataadj$input_base == "log",11:13] <-
  c(summary(glmsub[[8]])$coef[2,1], confint(glmsub[[8]])[2,])

dataadj[dataadj$study_id == "Odebeatu 2019" & dataadj$input_base == "lin",4:6] <- c(median(dfsub[[1]]$MBzP,na.rm = T),quantile(dfsub[[1]]$MBzP,0.25,na.rm = T)[[1]],quantile(dfsub[[1]]$MBzP,0.75,na.rm = T)[[1]])
dataadj[dataadj$study_id == "Odebeatu 2019" & dataadj$input_base == "log",4:6] <- c(median(dfsub[[2]]$MBzP,na.rm = T),quantile(dfsub[[2]]$MBzP,0.25,na.rm = T)[[1]],quantile(dfsub[[2]]$MBzP,0.75,na.rm = T)[[1]])
dataadj[dataadj$study_id == "Pilkerton 2018" & dataadj$input_base == "lin",9:10] <- c(mean(dfsub[[3]]$pfoa,na.rm = T),sd(dfsub[[3]]$pfoa,na.rm = T))
dataadj[dataadj$study_id == "Pilkerton 2018" & dataadj$input_base == "log",9:10] <- c(mean(dfsub[[4]]$pfoa,na.rm = T),sd(dfsub[[4]]$pfoa,na.rm = T))
dataadj[dataadj$study_id == "Cheang 2021" & dataadj$input_base == "lin",4:6] <- c(median(dfsub[[5]]$HBga,na.rm = T),quantile(dfsub[[5]]$HBga,0.25,na.rm = T)[[1]],quantile(dfsub[[5]]$HBga,0.75,na.rm = T)[[1]])
dataadj[dataadj$study_id == "Cheang 2021" & dataadj$input_base == "log",4:6] <- c(median(dfsub[[6]]$HBga,na.rm = T),quantile(dfsub[[6]]$HBga,0.25,na.rm = T)[[1]],quantile(dfsub[[6]]$HBga,0.75,na.rm = T)[[1]])
dataadj[dataadj$study_id == "Xu 2020a" & dataadj$input_base == "lin",4:6] <- c(median(dfsub[[7]]$LBXI5AL,na.rm = T),quantile(dfsub[[7]]$LBXI5AL,0.25,na.rm = T)[[1]],quantile(dfsub[[7]]$LBXI5AL,0.75,na.rm = T)[[1]])
dataadj[dataadj$study_id == "Xu 2020a" & dataadj$input_base == "log",4:6] <- c(median(dfsub[[8]]$LBXI5AL,na.rm = T),quantile(dfsub[[8]]$LBXI5AL,0.25,na.rm = T)[[1]],quantile(dfsub[[8]]$LBXI5AL,0.75,na.rm = T)[[1]])
```


We then re-run the same analysis as done previously.


```
dzoutadj <- data.frame("study" = dataadj$study_id, "input_base" = dataadj$input_base, "actual_beta" = dataadj$manual_beta, "actual_blci" = dataadj$manual_blci, "actual_buci" = dataadj$manual_buci, "beta" = c(1:nrow(dataadj)), "beta_lci" = c(1:nrow(dataadj)), "beta_uci" = c(1:nrow(dataadj)))
for(i in 1:nrow(dataadj)){
  dzoutadj[i,6:8] <- dzexp(input.base = dataadj$input_base[i], 
                        exp.dist = c(med = dataadj$exp_med[i], iqr1 = dataadj$exp_1q[i], iqr3 = dataadj$exp_3q[i],
                                     gmean = dataadj$exp_gmean[i], gsd = dataadj$exp_gsd[i],
                                     mean = dataadj$exp_mean[i], sd = dataadj$exp_sd[i]),
                        beta.dist = c(dataadj$beta_mean[i], dataadj$beta_lci[i], dataadj$beta_uci[i]),
                        log.base = dataadj$logbase[i])
}
dzoutadj$method <- "Dzierlenga Method"

dfoutadj <- dzoutadj
dfoutadj$prop_diff_beta <- (dfoutadj$beta/dfoutadj$actual_beta)-1
```


Finally we integrate the old and new proportional difference in beta
and format Table S6.


```
dfout$prop_diff_beta_adj <- dfoutadj$prop_diff_beta
ts6studylist <- c("Odebeatu 2019","Pilkerton 2018","Cheang 2021","Xu 2020a")
tabs6 <- dfout[dfout$study %in% ts6studylist & dfout$method == "Dzierlenga Method",]
tabs6 <- tabs6[order(match(tabs6$study,ts6studylist)),]
tabs6 <- tabs6[c("study","input_base","method","prop_diff_beta","prop_diff_beta_adj")]
tabs6 <- tabs6 %>% mutate_if(is.numeric, round, 3)
tabs6 <- tabs6[tabs6$input_base == "log",]
tabs6
```

# Table Snew1


```
indivstud <- data[!duplicated(data$study_id),]
indivstud <- indivstud[,c(1,19,14,18,20,21)]
```

# Table Snew1b


```
indivstudb <- datab[!duplicated(datab$study_id),]
indivstudb <- indivstudb[,c(1,19,14,18,20,21)]
```

LS0tDQp0aXRsZTogIlIgU2NyaXB0IGZvciBEYXRhIEFuYWx5c2lzIGFuZCBUYWJsZSBHZW5lcmF0aW9uIg0Kb3V0cHV0Og0KICBodG1sX2RvY3VtZW50Og0KICAgIHRvYzogdHJ1ZQ0KICAgIGRmX3ByaW50OiBwYWdlZA0KICAgIGNvZGVfZm9sZGluZzogc2hvdw0KICBodG1sX25vdGVib29rOiBkZWZhdWx0DQogIHBkZl9kb2N1bWVudDogZGVmYXVsdA0KLS0tDQoNCiMgQURFTVAgT3ZlcnZpZXcNCg0KIyMgQWltcw0KVGhlIGFpbSBvZiB0aGlzIHNpbXVsYXRpb24gaXMgdG8gZGVtb25zdHJhdGUgdGhlIHBlcmZvcm1hbmNlIG9mIHRoZSB0aHJlZSByZS1leHByZXNzaW9uIG1ldGhvZHMgKFJvZHJpZ3Vlei1CYXJyYW5jbywgcmI7IEFsdGVybmF0ZSwgYWx0OyBEemllcmxlbmdhLCBkeikgd2hlbiB1dGlsaXplZCBmb3IgYSByYW5nZSBvZiBkaWZmZXJlbnQsIHJlYWxpc3RpYyBwb3B1bGF0aW9ucyBhbmQgc2NlbmFyaW9zLg0KDQojIyBEYXRhLWdlbmVyYXRpbmcgbWVjaGFuaXNtcw0KRGF0YSB3aWxsIGJlIGdlbmVyYXRlZCBieSBwcm9kdWNpbmcgcGFyYW1ldHJpYyBkcmF3cyBmcm9tIGEga25vd24sIHRydW5jYXRlZCBsb2dub3JtYWwgZGlzdHJpYnV0aW9uIHVzaW5nIHRoZSBFbnZTdGF0czo6cmxub3JtVHJ1bmMoKSBmdW5jdGlvbi4gVGhlIG51bWJlciBvZiBpbmRpdmlkdWFscyBzaW11bGF0ZWQgKG5vYnMpIHdhcyBzZWxlY3RlZCBieSBmaW5kaW5nIHRoZSAxMHRoIGFuZCA5MHRoIHBlcmNlbnRpbGVzIG9mIHRoZSBudW1iZXIgb2YgaW5kaXZpZHVhbHMgaW5jbHVkZWQgaW4gdGhlIDE0IHN0dWRpZXMgZXhhbWluZWQgaW4gdGhpcyBpbnZlc3RpZ2F0aW9uLiBBIGxpc3Qgb2YgdmFsdWVzIHJlcHJlc2VudGluZyBhIHJlYXNvbmFibGUgcmFuZ2Ugb2YgeC12YWx1ZSBtZWFucyB3YXMgbG9nLXRyYW5zZm9ybWVkIGFuZCB1c2VkIGZvciBtZWFubG9nIChtdSkgZm9yIHRoZSBkaXN0cmlidXRpb24sIHdoaWxlIHNpZ21hIHZhbHVlcyB3ZXJlIHNlbGVjdGVkIHRvIGJlIDAuNSBvciAxIGFuZCB1c2VkIGZvciBzZGxvZyAoc2lnbWEpLg0KDQojIyBFc3RpbWFuZHMNCk91ciBlc3RpbWFuZCBvZiBpbnRlcmVzdCBpbiB0aGlzIHN0dWR5IGlzIHRoZSByZS1leHByZXNzZWQgcmVncmVzc2lvbiBtb2RlbCBmaXQgcGFyYW1ldGVyLCBiZXRhcmV4Lg0KDQojIyBNZXRob2RzDQoxKSBUaGlzIGludmVzdGlnYXRpb24gd2lsbCB1c2UgMTAwMCBzaW11bGF0ZWQgZGlzdHJpYnV0aW9ucyAobnNpbSkgZm9yIGVhY2ggc2V0IG9mIHBhcmFtZXRlcnMgdG8gZ2VuZXJhdGUgeC12YWx1ZXMgIA0KMikgWS12YWx1ZXMgd2lsbCB0aGVuIGJlIGNhbGN1bGF0ZWQgdXNpbmcgeSA9ICBiZXRhICogeCArIGVycm9yIHdoZXJlIGJldGEgaXMgc2V0IHRvIDAuNSwgMSwgb3IgMTAgYW5kIGVycm9yIGlzIHJhbmRvbWx5IGFzc2lnbmVkIGZyb20gYSBub3JtYWwgZGlzdHJpYnV0aW9uIHdpdGggYSBtZWFuIG9mIDAgYW5kIHN0YW5kYXJkIGRldmlhdGlvbiBvZiBiZXRhICogbXUgKiAwLjIuIA0KMykgUmVncmVzc2lvbiBtb2RlbHMgKGxvZy1saW5lYXIgb3IgbGluZWFyIGRlcGVuZGluZyBvbiB0aGUgZGlyZWN0aW9uIG9mIHJlLWV4cHJlc3Npb24pIHdpbGwgdGhlbiBiZSBnZW5lcmF0ZWQgZnJvbSB0aGUgeCBhbmQgeSB2YWx1ZXMgDQo0KSBSZS1leHByZXNzZWQgYmV0YXMgYXJlIHRoZW4gZ2VuZXJhdGVkIHVzaW5nIGVhY2ggb2YgdGhlIHJlLWV4cHJlc3Npb24gZnVuY3Rpb25zIGRlZmluZWQgYmVsb3cgKHJiZXhwLCBhbHRleHAsIGR6ZXhwKQ0KNSkgRmluYWxseSwgZGlhZ25vc3RpYyB2YWx1ZXMsIGluY2x1ZGluZyByZWxhdGl2ZSBiaWFzIGFuZCBNb250ZSBDYXJsbyBzdGFuZGFyZCBlcnJvciBvZiB0aGUgcmVsYXRpdmUgYmlhcywgYXJlIGNhbGN1bGF0ZWQgaW4gb3JkZXIgdG8gZGV0ZXJtaW5lIGhvdyB3ZWxsIHRoZSByZS1leHByZXNzZWQgYmV0YXMgcmVmbGVjdCB0cnVlIGJldGEgdmFsdWVzLg0KDQojIyBQZXJmb3JtYW5jZSBNZWFzdXJlcw0KVGhlIGFjY3VyYWN5IG9mIHRoZSByZS1leHByZXNzZWQgYmV0YSB2YWx1ZXMgd2lsbCBiZSBjYWxjdWxhdGVkIGFzIHRoZSByZWxhdGl2ZSBiaWFzIHVzaW5nIHRoZSBmb2xsb3dpbmcgZm9ybXVsYToNCg0KUmVsYXRpdmUgQmlhcyA9IDEvbnNpbSAqIHN1bSgoYmV0YXJleCAtIGJldGEpL2JldGEpDQoNCndoaWxlIHRoZSBza2V3ZWRuZXNzIG9mIHRoZSByZS1leHByZXNzZWQgdmFsdWUgd2FzIGNhbGN1bGF0ZWQgdXNpbmcgdGhlIE1vbnRlIENhcmxvIHN0YW5kYXJkIGVycm9yIG9mIHRoZSByZWxhdGl2ZSBiaWFzOg0KDQpNQ1NFID0gc3FydCgoMS8obnNpbSAtIChuc2ltIC0gMSkpKSAqIHN1bSgoKGJldGFyZXggLSBiZXRhKS9iZXRhKV4yKSkNCg0KIyBTZXR1cA0KDQpMb2FkIGluIHRoZSBuZWVkZWQgcGFja2FnZXMNCg0KYGBge3IgcGFja2FnZXMsIG1lc3NhZ2UgPSBGQUxTRSwgd2FybmluZz1GQUxTRX0NCmxpc3Qub2YucGFja2FnZXMgPC0gYygNCiAgImZvcmVhY2giLA0KICAiZG9QYXJhbGxlbCIsDQogICJnZ3Bsb3QyIiwNCiAgImRwbHlyIiwNCiAgIkVudlN0YXRzIiwNCiAgInN1cnZleSIsDQogICJkYXRhLnRhYmxlIiwNCiAgImRvUk5HIiwNCiAgImRvU05PVyIsDQogICJ0Y2x0ayIsDQogICJrbml0ciIsDQogICJNQVNTIiwNCiAgInRpZHl2ZXJzZSIsDQogICJnZ25ld3NjYWxlIiwNCiAgImdncHViciIsDQogICJnZ3JlcGVsIg0KICApDQoNCm5ldy5wYWNrYWdlcyA8LSBsaXN0Lm9mLnBhY2thZ2VzWyEobGlzdC5vZi5wYWNrYWdlcyAlaW4lIGluc3RhbGxlZC5wYWNrYWdlcygpWywiUGFja2FnZSJdKV0NCg0KaWYobGVuZ3RoKG5ldy5wYWNrYWdlcykgPiAwKXsNCiAgaW5zdGFsbC5wYWNrYWdlcyhuZXcucGFja2FnZXMsIGRlcD1UUlVFKQ0KfQ0KDQojbG9hZGluZyBwYWNrYWdlcw0KZm9yKHBhY2thZ2UuaSBpbiBsaXN0Lm9mLnBhY2thZ2VzKXsNCiAgc3VwcHJlc3NQYWNrYWdlU3RhcnR1cE1lc3NhZ2VzKA0KICAgIGxpYnJhcnkoDQogICAgICBwYWNrYWdlLmksIA0KICAgICAgY2hhcmFjdGVyLm9ubHkgPSBUUlVFDQogICAgICApDQogICAgKQ0KfQ0KYGBgDQoNCkxvYWQgaW4gdGhlIGRhdGFmcmFtZXMNCg0KYGBge3IgZGF0YXJlYWQxfQ0KZGF0YSA8LSByZWFkLmNzdihmaWxlID0gImRhdGEvUmVfZXhwcmVzc2lvbl9GdWxsX1RhYmxlXzExMDcyMDIyLmNzdiIpDQpkYXRhYiA8LSByZWFkLmNzdihmaWxlID0gImRhdGEvUmVfZXhwcmVzc2lvbl9GdWxsX1RhYmxlXzEwMTYyMDIzLmNzdiIpDQoNCiNUaGUgbmV4dCBkYXRhc2V0LCB0aGUgbnNjZW5hcmlvIG91dHB1dCwgY2FuIGJlIHJlZ2VuZXJhdGVkIGJ5IHJ1bm5pbmcgZnVsbHNpbS5SDQojTm90ZSB0aGF0IHRoZSBvdXRwdXQgLmNzdiB3aWxsIGhhdmUgdGhlIGRhdGUgdGhhdCB0aGUgc2ltdWxhdGlvbiB3YXMgY29tcGxldGVkDQojcmVxdWlyaW5nIHRoYXQgdGhlIGJlbG93IGxpbmUgYmUgdXBkYXRlZCB3aXRoIHRoZSBkZXNpcmVkIGZpbGUgZGF0ZQ0KbHJleGJldGF0YWIgPC0gZnJlYWQoImxyZXhiZXRhdGFiXzIwMjMtMDItMjIuY3N2IikNCg0KI0ZpbmFsbHksIGZvciB3b3JraW5nIHdpdGggZWFjaCBpbmRpdmlkdWFsIG5zaW0gZm9yIGFsbCBuc2NlbmFyaW8NCiN0aGUgYmVsb3cgd2lsbCBnZW5lcmF0ZSBhIHNpbmdsZSBkYXRhc2V0LiBJdCB3aWxsIHRha2UgYSBmZXcgc2Vjb25kcyB0byBsb2FkLA0KI2FuZCB0aGUgdmFsdWVzIHdpbGwgY2hhbmdlIGlmIHRoZSBmdWxsIHNpbXVsYXRpb24gaGFzIGJlZW4gcnVuIGFnYWluLg0KZmlsZXMgPC0gbGlzdC5maWxlcyhwYXRoID0gInNpbWRhdGEiLHBhdHRlcm4gPSAiLmNzdiIpDQp0ZW1wIDwtIGxhcHBseShwYXN0ZTAoInNpbWRhdGEvIixmaWxlcyksZnJlYWQsc2VwPSIsIikNCmFsbHNpbWRhdGEgPC0gcmJpbmRsaXN0KHRlbXApDQpybShmaWxlcyx0ZW1wKQ0KDQpgYGANCg0KTG9hZCBpbiB0aGUgcmUtZXhwcmVzc2lvbiBmdW5jdGlvbnMNCg0KYGBge3J9DQpzb3VyY2UoIi4vZnVuY3Rpb25zL3JiZXhwLlIiKQ0Kc291cmNlKCIuL2Z1bmN0aW9ucy9kemV4cC5SIikNCnNvdXJjZSgiLi9mdW5jdGlvbnMvYWx0ZXhwLlIiKQ0KYGBgDQoNCiMgRmlndXJlIDENCg0KRmlyc3Qgd2Ugc2V0IHVwIHRoZSBhZG1pbmlzdHJhdGl2ZSB0aGluZ3MgZm9yIG91ciBzaW1wbGUgcHNldWRvc2ltdWxhdGlvbiB0byBnZW5lcmF0ZSBGaWd1cmUgMSwgaW5jbHVkaW5nIHNldHRpbmcgYSBzZWVkIHRvIGdldCByZXByb2R1Y2libGUgcmVzdWx0cyBhbmQgZGVmaW5pbmcgYSBmZXcgdmFyaWFibGVzIHN1Y2ggYXMgb3VyIGxpc3Qgb2YgeF9tZWRpYW5zIG9mIGludGVyZXN0LiBUaGlzIHNpbXVsYXRlcyBhIHNwZWNpZmljIHNldCBvZiBzY2VuYXJpb3MgKG1lZGlhbiwgc2lnbWEsIGV0Yy4pIGFzIGRlZmluZWQgYmVsb3cgYW5kIHRob3NlIHZhbHVlcyBjYW4gYmUgY2hhbmdlZCB0byBleHBsb3JlIG90aGVyIHNjZW5hcmlvcy4NCg0KYGBge3IgZmlnMWRmZ2VuZXJhdGlvbiwgd2FybmluZyA9IEZBTFNFfQ0Kc2V0LnNlZWQoMTIzNCkNCg0KbWVkbGlzdCA8LSAxDQpucmVwIDwtIDJFMw0KbXUgPC0gbG9nKG1lZGxpc3QpDQpzaWdtYSA8LSAwLjUgDQpsb2diYXNlIDwtIGV4cCgxKSANCmJldGEgPC0gMSANCmVyciA8LSAwDQpyZWwgPC0gImxvZyIgDQoNCiMgR2VuZXJhdGUgdGhlIHgtdmFsdWVzIGJ5IHNhbXBsaW5nIGZyb20gYSBsb2dub3JtYWwgZGlzdHJpYnV0aW9uICJucmVwIiB0aW1lcywgDQojIGFuZCB0aGVuIHdlIGdlbmVyYXRlIHktdmFsdWVzIGJhc2VkIG9uIHRob3NlIHgtdmFsdWVzLg0KeGxvZyA9IHJsbm9ybVRydW5jKG5yZXAsIG1lYW5sb2cgPSBsb2cobWVkbGlzdCksIHNkbG9nID0gc2lnbWEpDQp5bG9nID0gYmV0YSAqIGxvZyh4bG9nLCBiYXNlID0gbG9nYmFzZSkNCg0KI1N0b3JpbmcgdmFsdWVzIGZyb20gdGhlIGxpbmVhciByZWdyZXNzaW9uIG9mIHkgdnMuIHggb3IgbG9nKHgpLg0KbG1saW4gPC0gbG0oeWxvZ354bG9nKQ0KbG1sb2cgPC0gbG0oeWxvZ35sb2coeGxvZywgYmFzZSA9IGxvZ2Jhc2UpKQ0KDQpyc3F1YXJlIDwtIHN1bW1hcnkobG1saW4pJHIuc3F1YXJlZA0KYmV0YSA8LSBsbWxpbiRjb2VmW1syXV0NCmxiZXRhIDwtIGxtbG9nJGNvZWZbWzJdXQ0KbGJfbG93X2NpIDwtIGNvbmZpbnQobG1sb2cpW1syLDFdXQ0KbGJfaGlfY2kgPC0gY29uZmludChsbWxvZylbWzIsMl1dDQogIA0KZmlnMWRmIDwtIGRhdGEuZnJhbWUoImV4X25vIiA9IDEsIA0KICAgICAgICAgICAgICAgICAgICAgICAgICJ4X21lZGlhbiIgPSByb3VuZChtZWRpYW4oeGxvZyksIDIpLCANCiAgICAgICAgICAgICAgICAgICAgICAgICAieF8yNSIgPSByb3VuZChzdW1tYXJ5KHhsb2cpLDIpW1syXV0sIA0KICAgICAgICAgICAgICAgICAgICAgICAgICJ4Xzc1IiA9IHJvdW5kKHN1bW1hcnkoeGxvZyksMilbWzVdXSwNCiAgICAgICAgICAgICAgICAgICAgICAgICAicmIiID0gcmJleHAobW9kZWwgPSAiYiIsIA0KICAgICAgICAgICAgICAgICAgICAgICAgICAgICAgeS5leHAgPSAiYWJzIiwgDQogICAgICAgICAgICAgICAgICAgICAgICAgICAgICB4LmV4cCA9ICJhYnMiLCANCiAgICAgICAgICAgICAgICAgICAgICAgICAgICAgIGJldGEgPSBsYmV0YSwgDQogICAgICAgICAgICAgICAgICAgICAgICAgICAgICBzZSA9IChsYl9oaV9jaS1sYl9sb3dfY2kpLzMuOTIsDQogICAgICAgICAgICAgICAgICAgICAgICAgICAgICBtZWFuLnggPSBtZWRpYW4oeGxvZyksDQogICAgICAgICAgICAgICAgICAgICAgICAgICAgICBtZWFuLnkgPSAwLA0KICAgICAgICAgICAgICAgICAgICAgICAgICAgICAgYSA9IGxvZ2Jhc2UsIA0KICAgICAgICAgICAgICAgICAgICAgICAgICAgICAgYiA9IGxvZ2Jhc2UsIA0KICAgICAgICAgICAgICAgICAgICAgICAgICAgICAgayA9IGxvZ2Jhc2UsIA0KICAgICAgICAgICAgICAgICAgICAgICAgICAgICAgYyA9IDEpW1sxXV0sIA0KICAgICAgICAgICAgICAgICAgICAgImR6IiA9IGR6ZXhwKGlucHV0LmJhc2UgPSAibG9nIiwNCiAgICAgICAgICAgICAgICAgICAgICAgICAgICAgIGV4cC5kaXN0ID0gYyhtZWQgPSBtZWRpYW4oeGxvZyksIA0KICAgICAgICAgICAgICAgICAgICAgICAgICAgICAgICAgICAgICAgICAgIGlxcjEgPSBzdW1tYXJ5KHhsb2cpW1syXV0sIA0KICAgICAgICAgICAgICAgICAgICAgICAgICAgICAgICAgICAgICAgICAgIGlxcjMgPXN1bW1hcnkoeGxvZylbWzVdXSksDQogICAgICAgICAgICAgICAgICAgICAgICAgICAgICBiZXRhLmRpc3QgPSBjKGxiZXRhLA0KICAgICAgICAgICAgICAgICAgICAgICAgICAgICAgICAgICAgICAgICAgICBsYl9sb3dfY2ksIA0KICAgICAgICAgICAgICAgICAgICAgICAgICAgICAgICAgICAgICAgICAgICBsYl9oaV9jaSksDQogICAgICAgICAgICAgICAgICAgICAgICAgICAgICBsb2cuYmFzZSA9IGxvZ2Jhc2UpW1sxXV0sIA0KICAgICAgICAgICAgICAgICAgICAgICAgICJhbHQiID0gYWx0ZXhwKGlucHV0LmJhc2UgPSAibG9nIiwgDQogICAgICAgICAgICAgICAgICAgICAgICAgICAgICAgIG1lZGlhbiA9IG1lZGlhbih4bG9nKSwNCiAgICAgICAgICAgICAgICAgICAgICAgICAgICAgICAgYmV0YS5kaXN0ID0gYyhsYmV0YSwNCiAgICAgICAgICAgICAgICAgICAgICAgICAgICAgICAgICAgICAgICAgICAgICBsYl9sb3dfY2ksIA0KICAgICAgICAgICAgICAgICAgICAgICAgICAgICAgICAgICAgICAgICAgICAgIGxiX2hpX2NpKSwNCiAgICAgICAgICAgICAgICAgICAgICAgICAgICAgICAgbG9nLmJhc2UgPSBsb2diYXNlKVtbMV1dLCANCiAgICAgICAgICAgICAgICAgICAgICAgICAib2JzZXJ2ZWRfYmV0YSIgPSByb3VuZChiZXRhLDIpLA0KICAgICAgICAgICAgICAgICAgICAgICAgICJyc3F1YXJlIiA9IHJzcXVhcmUpDQpgYGANCg0KIyMgUHNldWRvc2ltdWxhdGlvbiBGaWd1cmUNCldlIGNhbiB0aGVuIHRha2UgdGhlIHZhbHVlcyBzaW11bGF0ZWQgdG8gZ2VuZXJhdGUgYSBmaWd1cmUgdGhhdCBjYW4gaGVscCBkZW1vbnN0cmF0ZSB0aGUgcmFuZ2Ugb3ZlciB3aGljaCBhIGdpdmVuIHJlLWV4cHJlc3Npb24gbWV0aG9kIG1heSBiZSB1c2VmdWwuDQoNCmBgYHtyIGZpZzEsIGZpZy5oZWlnaHQ9IDcsIGZpZy53aWR0aCA9IDl9DQoNCnAxIDwtIGdncGxvdCgpKw0KICBnZW9tX2xpbmUoYWVzKHggPSB4bG9nLCB5ID0geWxvZywgY29sb3IgPSAnb2cnKSwgc2l6ZSA9IDEuNCwgbGluZXR5cGUgPSAxKSsNCiAgZ2VvbV9zZWdtZW50KGFlcyh4ID0gMCwgeSA9IDAqZmlnMWRmJG9ic2VydmVkX2JldGEtZmlnMWRmJG9ic2VydmVkX2JldGEsIHhlbmQgPSAobWF4KHlsb2cpK2ZpZzFkZiRvYnNlcnZlZF9iZXRhKS9maWcxZGYkb2JzZXJ2ZWRfYmV0YSwgeWVuZCA9IG1heCh5bG9nKSwgY29sb3IgPSAnb2JzJyksc2l6ZSA9IDEpKw0KICBnZW9tX3NlZ21lbnQoYWVzKHggPSAwLCB5ID0gMCpmaWcxZGYkcmItZmlnMWRmJHJiLCB4ZW5kID0gKG1heCh5bG9nKStmaWcxZGYkcmIpL2ZpZzFkZiRyYiwgeWVuZCA9IG1heCh5bG9nKSwgY29sb3IgPSAncmInKSxzaXplID0gMSwgbGluZXR5cGUgPSA2KSsNCiAgZ2VvbV9zZWdtZW50KGFlcyh4ID0gMCwgeSA9ICgwKSpmaWcxZGYkZHotZmlnMWRmJGR6LCB4ZW5kID0gKG1heCh5bG9nKStmaWcxZGYkZHopL2ZpZzFkZiRkeiwgeWVuZCA9IG1heCh5bG9nKSwgY29sb3IgPSAnZHonKSxzaXplID0gMS41LGxpbmV0eXBlID0gMykrDQogIGdlb21fc2VnbWVudChhZXMoeCA9IDAsIHkgPSAoMCkqZmlnMWRmJGFsdC1maWcxZGYkYWx0LCB4ZW5kID0gKG1heCh5bG9nKStmaWcxZGYkYWx0KS9maWcxZGYkYWx0LCB5ZW5kID0gbWF4KHlsb2cpLCBjb2xvciA9ICdhbHQnKSxzaXplID0gMSwgbGluZXR5cGUgPSAyKSsNCiAgc2NhbGVfY29sb3VyX21hbnVhbChuYW1lID0gJycsDQogICAgICAgICAgICAgICAgICAgICAgdmFsdWVzID1jKCdvZyc9J2JsYWNrJywnb2JzJyA9ICcjRTY5RjAwJywgJ3JiJyA9ICcjQ0M3OUE3JywnZHonPScjNTZCNEU5JywnYWx0Jz0nI0Q1NUUwMCcpLA0KICAgICAgICAgICAgICAgICAgICAgICMgbGFiZWxzID0gYygiU2ltdWxhdGVkIERhdGEiLCJTdGFuZGFyZCIsICJSb2RyaWd1ZXogQmFycmFuY28iLCJBbHRlcm5hdGUiLCJEemllcmxlbmdhIiksDQogICAgICAgICAgICAgICAgICAgICAgbGFiZWxzID0gYygiU2ltdWxhdGVkXG5EYXRhIiwgDQogICAgICAgICAgICAgICAgICAgICAgICAgICAgICAgICBleHByZXNzaW9uKGl0YWxpYygiXHUwM2IyIilbRXN0aW1hbmRdKSwNCiAgICAgICAgICAgICAgICAgICAgICAgICAgICAgICAgZXhwcmVzc2lvbihpdGFsaWMoIlx1MDNiMiIpW1JCXSksDQogICAgICAgICAgICAgICAgICAgICAgICAgICAgICAgIGV4cHJlc3Npb24oaXRhbGljKCJcdTAzYjIiKVtEel0pLA0KICAgICAgICAgICAgICAgICAgICAgICAgICAgICAgICBleHByZXNzaW9uKGl0YWxpYygiXHUwM2IyIilbQWx0XSkpLA0KICAgICAgICAgICAgICAgICAgICAgIGd1aWRlID0gZ3VpZGVfbGVnZW5kKG92ZXJyaWRlLmFlcyA9IGxpc3QobGluZXR5cGUgPSBjKDEsMSw2LDMsMiksbHdkID0gYygxLDEsMSwxLDEpKSkpKw0KICB0aGVtZV9idygpKw0KICBzY2FsZV95X2NvbnRpbnVvdXMobmFtZSA9ICJPdXRjb21lIFZhcmlhYmxlIFZhbHVlc1xuIikrDQogIHNjYWxlX3hfY29udGludW91cyhuYW1lID0gIlxuRXhwb3N1cmUgVmFyaWFibGUgVmFsdWVzIikrDQogIGdlb21fdmxpbmUoYWVzKHhpbnRlcmNlcHQgPSBmaWcxZGYkeF8yNSkpKw0KICBnZW9tX3ZsaW5lKGFlcyh4aW50ZXJjZXB0ID0gZmlnMWRmJHhfNzUpKSsNCiAgYW5ub3RhdGUoZ2VvbSA9ICJ0ZXh0IiwgeCA9IGZpZzFkZiR4XzI1LShmaWcxZGYkeF8yNSowLjQpLCB5ID0gbWF4KHlsb2cpKjAuOCwgbGFiZWwgPSBwYXN0ZTAoIlF1YXJ0aWxlIDFcbj0gIiwgcm91bmQoZmlnMWRmJHhfMjUsMikpLCBmb250ZmFjZSA9IDIpKw0KICBhbm5vdGF0ZShnZW9tID0gInRleHQiLCB4ID0gZmlnMWRmJHhfNzUrKGZpZzFkZiR4Xzc1KjAuMiksIHkgPSBtYXgoeWxvZykqMC44LCBsYWJlbCA9IHBhc3RlMCgiUXVhcnRpbGUgM1xuPSAiLCByb3VuZChmaWcxZGYkeF83NSwyKSksIGZvbnRmYWNlID0gMikrDQogIGFubm90YXRlKGdlb20gPSAidGV4dCIsIHggPSAoKG1heCh5bG9nKStmaWcxZGYkb2JzZXJ2ZWRfYmV0YSkvZmlnMWRmJG9ic2VydmVkX2JldGEpKjEuMDEsIHkgPSBtYXgoeWxvZykqMS4wOCwgY29sb3IgPSAnZ29sZGVucm9kJywgZm9udGZhY2UgPSAyLCBsYWJlbCA9ICJTdGFuZGFyZCBsaW5lYXIgcmVncmVzc2lvbiBsaW5lXG5maXQgdG8gdGhlIHNpbXVsYXRlZCBkYXRhIikrDQogIGdlb21fc2VnbWVudChhZXMoeCA9ICgobWF4KHlsb2cpK2ZpZzFkZiRvYnNlcnZlZF9iZXRhKS9maWcxZGYkb2JzZXJ2ZWRfYmV0YSkqMC45NSwgeSA9IG1heCh5bG9nKSoxLjAyLCB4ZW5kID0gKG1heCh5bG9nKSowLjkzICtmaWcxZGYkb2JzZXJ2ZWRfYmV0YSkvZmlnMWRmJG9ic2VydmVkX2JldGEsIHllbmQgPSBtYXgoeWxvZykqMC45NSksc2l6ZSA9IDAuNzUsY29sb3IgPSAnYmxhY2snKSsNCiAgdGhlbWUoYXhpcy50aXRsZS54ID0gZWxlbWVudF90ZXh0KHNpemUgPSAxNCwgZmFjZSA9ICdib2xkJyksDQogICAgICAgIGF4aXMudGV4dC54ID0gZWxlbWVudF90ZXh0KHNpemUgPSAxMiwgZmFjZSA9ICdib2xkJyksDQogICAgICAgIGF4aXMudGl0bGUueSA9IGVsZW1lbnRfdGV4dChzaXplID0gMTQsIGZhY2UgPSAnYm9sZCcpLA0KICAgICAgICBheGlzLnRleHQueSA9IGVsZW1lbnRfdGV4dChzaXplID0gMTIsIGZhY2UgPSAnYm9sZCcpLA0KICAgICAgICBsZWdlbmQudGl0bGUgPSBlbGVtZW50X3RleHQoc2l6ZSA9IDE0LCBmYWNlID0gJ2JvbGQnKSwNCiAgICAgICAgbGVnZW5kLnRleHQgPSBlbGVtZW50X3RleHQoc2l6ZSA9IDEyLCBmYWNlID0gJ2JvbGQnKSwNCiAgICAgICAgbGVnZW5kLmtleS53aWR0aCA9IHVuaXQoMiwibGluZSIpLA0KICAgICAgICBsZWdlbmQudGV4dC5hbGlnbiA9IDApDQpwMQ0KYGBgDQoNCiMgVGFibGUgMQ0KDQpGaXJzdCB3ZSBuZWVkIHRvIGRvIHNvbWUgbGlnaHQgZm9ybWF0dGluZyBvZiB0aGUgZGF0YXNldHMgdXNlZCBmb3IgdGhpcyBwYXJ0IG9mIHRoZSBhbmFseXNpcy4gRm9yIGV4YW1wbGUsIHdlIGNhbiBnZW5lcmF0ZSBhIHNtYWxsIHRhYmxlIGRldGFpbGluZyBzb21lIG9mIHRoZSBpbmZvcm1hdGlvbiBmcm9tIHRoZSBhY3R1YWwgc3R1ZGllcyBhbmQgdGhlIHNpbXVsYXRpb24gZGF0YXNldCB0aGF0IGRldGFpbHMgdGhlIHNjZW5hcmlvcyB1c2VkIGluIHRoZSBzaW11bGF0aW9uDQoNCmBgYHtyIGZpZzJkZmZvcm1hdH0NCg0Kc3R1ZHlsaXN0IDwtIGMoIkJ1bGthIDIwMjEiLCAiTGVlIDIwMjAiLCJPZGViZWF0dSAyMDE5IiwNCiAgICAgICAgICAgICAgICJYdSAyMDIwIiwiU3RlaW4gMjAxNiIsIlBpbGtlcnRvbiAyMDE4IiwNCiAgICAgICAgICAgICAgICJDaGVhbmcgMjAyMSIsIkFicmFoYW0gMjAyMCIsIkFwZWxiZXJnIDIwMDciLA0KICAgICAgICAgICAgICAgIldhc2hpbm8gMjAwOSIsIkhhbW0gMjAxMCIsICJDaGVuIDIwMTIiLA0KICAgICAgICAgICAgICAgIkRhcnJvdyAyMDEzIiwiU3RlZW5sYW5kIDIwMDkiKSAjU3R1ZHkgSURzDQpzdHVkeW4gPC0gYyg4Nzc4LCAxMjQsNzc2NSwgMTk0NywgMTE5MSwgNjIxLCAyODk5LCANCiAgICAgICAgICAgIDEwMSwgMjkzLCA0MjgsIDI1MiwgNDI5LCAxNjMwLCA0NjI5NCkgI24gaW4gZWFjaCBzdHVkeQ0KYWdnbiA8LSByb3VuZChjKHF1YW50aWxlKHN0dWR5biwgMC4xKSxxdWFudGlsZShzdHVkeW4sIDAuMjUpLA0KICAgICAgICAgICAgICAgIHF1YW50aWxlKHN0dWR5biwgMC41KSxxdWFudGlsZShzdHVkeW4sIDAuNzUpLA0KICAgICAgICAgICAgICAgIHF1YW50aWxlKHN0dWR5biwgMC45KSksIGRpZ2l0cyA9IDApICNuIG9mIHZhcmlvdXMgcXVhbnRpbGVzIGZvciBzdHVkaWVzIG92ZXJhbGwNCg0KcmVsIDwtIGMoImxvZyIpICNGb3IgdGhpcyBpbnZlc3RpZ2F0aW9uLCBvbmx5IHJlLWV4cHJlc3NlZCBmcm9tIGxvZyB0byBsaW4NCmxvZ2Jhc2UgPC0gYyhleHAoMSksMiwxMCkgI3ZhcmlvdXMgbG9nIGJhc2VzIGludmVzdGlnYXRlZA0Kc2lnbWEgPC0gYygwLjI1LDAuNDUsMC42NSwwLjg1KSAjdmFyaW91cyBzaWdtYSB2YWx1ZXMgaW52ZXN0aWdhdGVkDQpiZXRhIDwtIGMoLTE1LDAuNSwxLDEwLDMwKSAjQmV0YURHTSwgTm90ZSB0aGF0IGluY2x1ZGluZyBhIGJldGEgPSAwIGJyZWFrcyB0aGluZ3MgYmVjYXVzZSB3ZSdyZSBub3JtYWxseSBmaWx0ZXJpbmcgb3V0IHZlcnkgc21hbGwgYmV0YXMgYmVjYXVzZSBldmVuIGEgc21hbGwgY2hhbmdlIGluIGEgdmVyeSBzbWFsbCBiZXRhIGNhbiByZXN1bHQgaW4gYSBodWdlIHJlbGF0aXZlIGJpYXMgdmFsdWUgdGhhdCdzIG5vdCByZWFsbHkgcmVsZXZhbnQgb24gbW9zdCBzY2FsZXMgKGUuZy4gZGlmZmVyZW5jZSBiZXR3ZWVuIDEuMEUtOCBhbmQgMS4wRS05KQ0KbWVkbGlzdCA8LSBjKDAuMjUsMC41LDEsMiw0LDgsMTYsMzIpICNNZWRpYW5zDQpub2JzIDwtIGMoYWdnbltbMV1dLGFnZ25bWzVdXSkgI0NvbnNpZGVyZWQgdGhlIDEwdGggYW5kIDkwdGggJWlsZSBvZiBhY3R1YWwgc3R1ZHkgbidzDQpuc2ltIDwtIDIwMDAgIzIwMDAgc2ltdWxhdGlvbnMgY2hvc2VuIGZvciB0aGlzIGludmVzdGlnYXRpb24NCg0Kc2ltZGYgPC0gYXMuZGF0YS50YWJsZShleHBhbmQuZ3JpZChub2JzID0gbm9icyxtZWRsaXN0ID0gbWVkbGlzdCxiZXRhID0gYmV0YSxzaWdtYSA9IHNpZ21hLGxvZ2Jhc2UgPSBsb2diYXNlLHJlbCA9IHJlbCkpDQpzaW1kZiRleF9ubyA8LSBjKDE6bnJvdyhzaW1kZikpDQpzaW1kZiA8LSByZXYoc2ltZGYpDQpzaW1kZiRuc2ltIDwtIG5zaW0NCg0KbHJleGJldGF0YWIkbG9nYmFzZVtscmV4YmV0YXRhYiRsb2diYXNlID4gMiAmIGxyZXhiZXRhdGFiJGxvZ2Jhc2UgPCAzXSA8LSAiZSINCmFsbHNpbWRhdGEkbG9nYmFzZVthbGxzaW1kYXRhJGxvZ2Jhc2UgPT0gZXhwKDEpXSA8LSAiZSINCg0KYWxsc2ltZGF0YSA8LSBhbGxzaW1kYXRhWyxjKCJleF9ubyIsInhsb2ciLCJlcnJvciIsImVycnNkIiwieWxvZyIsInhfbWVkaWFuIiwieF8yNSIsInhfNzUiLCJyYmwiLCJyYiIsInJidSIsImFsdGwiLCJhbHQiLCJhbHR1IiwiZHpsIiwiZHoiLCJkenUiLCJiX2xvd19jaSIsIm9ic2VydmVkX2JldGEiLCJiX2hpX2NpIiwicnNxdWFyZSIsIlJlbEJpYXNfcmJsIiwiUmVsQmlhc19yYiIsIlJlbEJpYXNfcmJ1IiwiUmVsQmlhc19hbHRsIiwiUmVsQmlhc19hbHQiLCJSZWxCaWFzX2FsdHUiLCJSZWxCaWFzX2R6bCIsIlJlbEJpYXNfZHoiLCJSZWxCaWFzX2R6dSIsIk1DX1NFX1JlbEJpYXNfcmIiLCJNQ19TRV9SZWxCaWFzX2FsdCIsIk1DX1NFX1JlbEJpYXNfZHoiKV0NCmFsbHNpbWRhdGEgPC0gbWVyZ2Uoc2ltZGYsYWxsc2ltZGF0YSxieSA9ICJleF9ubyIpDQoNCiNDcmVhdGUgYSBkYXRhZnJhbWUgZm9yIHdoZW4gYmV0YSA+IDANCmxyZXhiZXRhcmVnIDwtIGxyZXhiZXRhdGFiW2JldGE+MF0NCmBgYA0KDQpUaGVuIHdlIGNhbiBnZW5lcmF0ZSBUYWJsZSAxDQoNCmBgYHtyfQ0KdGFiMSA8LSBkYXRhLmZyYW1lKCJQYXJhbWV0ZXIiID0gYygibn5vYnN+IiwNCiAgICAgICAgICAgICAgICAgICAgICAgICAgICAgICAgICAgImUiLA0KICAgICAgICAgICAgICAgICAgICAgICAgICAgICAgICAgICAiXHUwM2IyfkRHTX4iLA0KICAgICAgICAgICAgICAgICAgICAgICAgICAgICAgICAgICAiTG9nYmFzZSIsDQogICAgICAgICAgICAgICAgICAgICAgICAgICAgICAgICAgICJTIiwNCiAgICAgICAgICAgICAgICAgICAgICAgICAgICAgICAgICAgIm1lZGlhbiIpLA0KICAgICAgICAgICAgICAgICAgICJQb3NzaWJsZSBWYWx1ZXMiID0gYyhwYXN0ZShub2JzLGNvbGxhcHNlID0gIiwgIiksDQogICAgICAgICAgICAgICAgICAgICAgICAgICAgICAgICAgICAgICAgICJTZWxlY3RlZCBmcm9tIGEgbm9ybWFsIGRpc3RyaWJ1dGlvbiB3aXRoIG1lYW4gPSAwIGFuZCBTRCA9IFNEZSoiLA0KICAgICAgICAgICAgICAgICAgICAgICAgICAgICAgICAgICAgICAgICAgcGFzdGUoYmV0YSwgY29sbGFwc2UgPSAiLCAiKSwNCiAgICAgICAgICAgICAgICAgICAgICAgICAgICAgICAgICAgICAgICAgIHBhc3RlKGxvZ2Jhc2UsIGNvbGxhcHNlID0gIiwgIiksDQogICAgICAgICAgICAgICAgICAgICAgICAgICAgICAgICAgICAgICAgICBwYXN0ZShzaWdtYSwgY29sbGFwc2UgPSAiLCAiKSwNCiAgICAgICAgICAgICAgICAgICAgICAgICAgICAgICAgICAgICAgICAgIHBhc3RlKG1lZGxpc3QsIGNvbGxhcHNlID0gIiwgIikpLA0KICAgICAgICAgICAgICAgICAgICJSYXRpb25hbGUgZm9yIENob2ljZSIgPSBjKCJRdWFudGlsZXMgKDEwdGgsOTB0aCkgb2YgdGhlIGRpc3RyaWJ1dGlvbiBvZiBzYW1wbGUgc2l6ZXMgZm9yIHRoZSAxNSByZWFsIGRhdGEgZXhhbXBsZXMiLA0KICAgICAgICAgICAgICAgICAgICAgICAgICAgICAgICAgICAgICAgICAgICAgICJTdGFuZGFyZCBkZXZpYXRpb24gc2VsZWN0ZWQgdG8gcmVzdWx0IGluIGFuIFJeMiB+IDAuMiwgdG8gbWFrZSByZWFsaXN0aWMgbW9kZWxzIiwNCiAgICAgICAgICAgICAgICAgICAgICAgICAgICAgICAgICAgICAgICAgICAgICAiQnJvYWQgcmFuZ2Ugb2YgZWZmZWN0IHNpemVzIGVuY29tcGFzc2luZyB0aG9zZSBpbnQgaGUgcmVhbCBkYXRhIGV4YW1wbGVzLiIsDQogICAgICAgICAgICAgICAgICAgICAgICAgICAgICAgICAgICAgICAgICAgICAgIkxvZyBiYXNlcyB1c2VkIGluIHRoZSAxNSByZWFsIGRhdGEgZXhhbXBsZXMiLA0KICAgICAgICAgICAgICAgICAgICAgICAgICAgICAgICAgICAgICAgICAgICAgICJTZWxlY3RlZCB2YWx1ZXMgY292ZXIgdGhlIGFwcHJveGltYXRlIHJhbmdlIG9mIHNpZ21hIHZhbHVlcyBpbiB0aGUgMTUgcmVhbCBkYXRhIGV4YW1wbGVzLiIsDQogICAgICAgICAgICAgICAgICAgICAgICAgICAgICAgICAgICAgICAgICAgICAgIlNlbGVjdGVkIHZhbHVlcyBjb3ZlciB0aGUgcmFuZ2Ugb2YgbWVkaWFuIGV4cG9zdXJlIGluIHRoZSAxNSByZWFsIGRhdGEgZXhhbXBsZXMuIikpDQp0YWIxIDwtIGtuaXRyOjprYWJsZSh0YWIxKQ0KDQp0YWIxDQpgYGANCg0KIyBGaWd1cmUgMg0KDQoNCg0KIyMgT3JkaW5hcnkgTGVhc3QgU3F1YXJlcyBSZWdyZXNzaW9uDQoNCkhlcmUgaXMgd2hlcmUgd2UgcnVuIHRoZSBvcmRpbmFyeSBsZWFzdCBzcXVhcmVzIHJlZ3Jlc3Npb24gYW5kIHBpY2sgdGhlIG1vc3QgYXBwcm9wcmlhdGUgbW9kZWwgYmFzZWQgb24gbWluaW1pemluZyBBSUMgd2l0aCBhIGstcGFyYW1ldGVyIH4zLjg0Lg0KDQpgYGB7ciBPTFN9DQoNCiNGaXQgZm9yIFJlbGF0aXZlIEJpYXMgdnMuIHNpZ21hIGZvciBBbHRlcm5hdGl2ZSBNZXRob2QNCiAgI0Jhc2VsaW5lIGZpdCB3aXRoIGFsbCB2YXJpYWJsZXMgaW5jbHVkZWQNCmZpdDJwNSA8LSBsbShSZWxCaWFzX2FsdH5wb2x5KHNpZ21hLDIscmF3PVQpKw0KICAgICAgICAgICAgICAgQyhhcy5mYWN0b3IobG9nYmFzZSksYmFzZSA9IDMpK2xvZ2Jhc2U6c2lnbWErDQogICAgICAgICAgICAgICBiZXRhK2JldGE6c2lnbWErDQogICAgICAgICAgICAgICBtZWRsaXN0K21lZGxpc3Q6c2lnbWErDQogICAgICAgICAgICAgICBub2JzK25vYnM6c2lnbWEsIGRhdGEgPSBscmV4YmV0YXJlZykgDQogICNNaW5pbWl6ZSBBSUMgYmFzZWQgb24gYSBrID0gcCA8IDAuMDUgaW4gQ2hpLXNxIHRlc3QNCnN0ZXAuMnA1IDwtIHN0ZXBBSUMoZml0MnA1LGRpcmVjdGlvbiA9ICJib3RoIix0cmFjZSA9IEYsIA0KICAgICAgICAgICAgICAgICAgICBrID0gcWNoaXNxKDAuMDUsMSxsb3dlci50YWlsID0gRkFMU0UpKSANCiMgc3VtbWFyeShzdGVwLjJwNSkNCiAgI0Jhc2VkIG9uIHZhcmlhYmxlcyByZXRhaW5lZCBhYm92ZSwgZ2VuZXJhdGUgZml0cyBmb3IgdGhlIGxvd2VyIGFuZCB1cHBlciBjb25maWRlbmNlIGludGVydmFscw0KZml0MnA1bCA8LSBsbShSZWxCaWFzX2FsdGx+cG9seShzaWdtYSwyLHJhdz1UKStDKGFzLmZhY3Rvcihsb2diYXNlKSxiYXNlID0gMykrbG9nYmFzZTpzaWdtYStub2JzK25vYnM6c2lnbWEsIGRhdGEgPSBscmV4YmV0YXJlZykNCiMgc3VtbWFyeShmaXQycDVsKQ0KIGZpdDJwNXUgPC0gbG0oUmVsQmlhc19hbHR1fnBvbHkoc2lnbWEsMixyYXc9VCkrQyhhcy5mYWN0b3IobG9nYmFzZSksYmFzZSA9IDMpK2xvZ2Jhc2U6c2lnbWErbm9icytub2JzOnNpZ21hLCBkYXRhID0gbHJleGJldGFyZWcpDQojIHN1bW1hcnkoZml0MnA1dSkNCg0KI0ZpdCBmb3IgUmVsYXRpdmUgQmlhcyB2cy4gc2lnbWEgZm9yIFJvZHJpZ3Vlei1CYXJyYW5jbyBNZXRob2QNCmZpdDJwNiA8LSBsbShSZWxCaWFzX3JifnBvbHkoc2lnbWEsMixyYXc9VCkrDQogICAgICAgICAgICAgICBsb2diYXNlK2xvZ2Jhc2U6c2lnbWErDQogICAgICAgICAgICAgICBiZXRhK2JldGE6c2lnbWErDQogICAgICAgICAgICAgICBtZWRsaXN0K21lZGxpc3Q6c2lnbWErDQogICAgICAgICAgICAgICBub2JzK25vYnM6c2lnbWEsIGRhdGEgPSBscmV4YmV0YXJlZykNCnN0ZXAuMnA2IDwtIHN0ZXBBSUMoZml0MnA2LGRpcmVjdGlvbiA9ICJib3RoIix0cmFjZSA9IEYsIA0KICAgICAgICAgICAgICAgICAgICBrID0gcWNoaXNxKDAuMDUsMSxsb3dlci50YWlsID0gRkFMU0UpKQ0KIyBzdW1tYXJ5KHN0ZXAuMnA2KQ0KZml0MnA2bCA8LSBsbShSZWxCaWFzX3JibH5wb2x5KHNpZ21hLDIscmF3PVQpK21lZGxpc3Qrc2lnbWE6bWVkbGlzdCwgZGF0YSA9IGxyZXhiZXRhcmVnKQ0KIyBzdW1tYXJ5KGZpdDJwNmwpDQpmaXQycDZ1IDwtIGxtKFJlbEJpYXNfcmJ1fnBvbHkoc2lnbWEsMixyYXc9VCkrbWVkbGlzdCtzaWdtYTptZWRsaXN0LCBkYXRhID0gbHJleGJldGFyZWcpDQojIHN1bW1hcnkoZml0MnA2dSkNCg0KI0ZpdCBmb3IgUmVsYXRpdmUgQmlhcyB2cy4gc2lnbWEgZm9yIER6aWVybGVuZ2EgTWV0aG9kDQpmaXQycDcgPC0gbG0oUmVsQmlhc19ken5wb2x5KHNpZ21hLDIscmF3PVQpKw0KICAgICAgICAgICAgICAgQyhhcy5mYWN0b3IobG9nYmFzZSksYmFzZSA9IDMpK2xvZ2Jhc2U6c2lnbWErDQogICAgICAgICAgICAgICBiZXRhK2JldGE6c2lnbWErDQogICAgICAgICAgICAgICBtZWRsaXN0K21lZGxpc3Q6c2lnbWErDQogICAgICAgICAgICAgICBub2JzK25vYnM6c2lnbWEsIGRhdGEgPSBscmV4YmV0YXJlZykNCnN0ZXAuMnA3IDwtIHN0ZXBBSUMoZml0MnA3LGRpcmVjdGlvbiA9ICJib3RoIix0cmFjZSA9IEYsIA0KICAgICAgICAgICAgICAgICAgICBrID0gcWNoaXNxKDAuMDUsMSxsb3dlci50YWlsID0gRkFMU0UpKQ0KIyBzdW1tYXJ5KHN0ZXAuMnA3KQ0KZml0MnA3bCA8LSBsbShSZWxCaWFzX2R6bH5wb2x5KHNpZ21hLDIscmF3PVQpK25vYnMrbm9iczpzaWdtYSwgZGF0YSA9IGxyZXhiZXRhcmVnKQ0KIyBzdW1tYXJ5KGZpdDJwN2wpDQpmaXQycDd1IDwtIGxtKFJlbEJpYXNfZHp1fnBvbHkoc2lnbWEsMixyYXc9VCkrbm9icytub2JzOnNpZ21hLCBkYXRhID0gbHJleGJldGFyZWcpDQojIHN1bW1hcnkoZml0MnA3dSkNCg0KI1dlIHRoZW4gcmVwZWF0IHRoZSBzYW1lIHRoaW5nIGZvciB0aGUgZnVsbCBuc2ltKm5zY2VuYXJpbyBkYXRhc2V0ICh3aGVyZSBiZXRhID4gMCkNCmZpdDJwNWYgPC0gbG0oUmVsQmlhc19hbHR+cG9seShzaWdtYSwyLHJhdz1UKSsNCiAgICAgICAgICAgICAgICBsb2diYXNlK2xvZ2Jhc2U6c2lnbWErDQogICAgICAgICAgICAgICAgYmV0YStiZXRhOnNpZ21hKw0KICAgICAgICAgICAgICAgIG1lZGxpc3QrbWVkbGlzdDpzaWdtYSsNCiAgICAgICAgICAgICAgICBub2JzK25vYnM6c2lnbWEsIGRhdGEgPSBhbGxzaW1kYXRhW2JldGEgPiAwXSkNCnN0ZXAuMnA1ZiA8LSBzdGVwQUlDKGZpdDJwNWYsZGlyZWN0aW9uID0gImJvdGgiLHRyYWNlID0gRiwgDQogICAgICAgICAgICAgICAgICAgICBrID0gcWNoaXNxKDAuMDUsMSxsb3dlci50YWlsID0gRkFMU0UpKQ0KIyBzdW1tYXJ5KHN0ZXAuMnA1ZikNCg0KZml0MnA2ZiA8LSBsbShSZWxCaWFzX3JifnBvbHkoc2lnbWEsMixyYXc9VCkrDQogICAgICAgICAgICAgICAgbG9nYmFzZStsb2diYXNlOnNpZ21hKw0KICAgICAgICAgICAgICAgIGJldGErYmV0YTpzaWdtYSsNCiAgICAgICAgICAgICAgICBtZWRsaXN0K21lZGxpc3Q6c2lnbWErDQogICAgICAgICAgICAgICAgbm9icytub2JzOnNpZ21hLCBkYXRhID0gYWxsc2ltZGF0YVtiZXRhID4gMF0pDQpzdGVwLjJwNmYgPC0gc3RlcEFJQyhmaXQycDZmLGRpcmVjdGlvbiA9ICJib3RoIix0cmFjZSA9IEYsIA0KICAgICAgICAgICAgICAgICAgICAgayA9IHFjaGlzcSgwLjA1LDEsbG93ZXIudGFpbCA9IEZBTFNFKSkNCiMgc3VtbWFyeShzdGVwLjJwNmYpDQoNCmZpdDJwN2YgPC0gbG0oUmVsQmlhc19ken5wb2x5KHNpZ21hLDIscmF3PVQpKw0KICAgICAgICAgICAgICAgIGxvZ2Jhc2UrbG9nYmFzZTpzaWdtYSsNCiAgICAgICAgICAgICAgICBiZXRhK2JldGE6c2lnbWErDQogICAgICAgICAgICAgICAgbWVkbGlzdCttZWRsaXN0OnNpZ21hKw0KICAgICAgICAgICAgICAgIG5vYnMrbm9iczpzaWdtYSwgZGF0YSA9IGFsbHNpbWRhdGFbYmV0YSA+IDBdKQ0Kc3RlcC4ycDdmIDwtIHN0ZXBBSUMoZml0MnA3ZixkaXJlY3Rpb24gPSAiYm90aCIsdHJhY2UgPSBGLCANCiAgICAgICAgICAgICAgICAgICAgIGsgPSBxY2hpc3EoMC4wNSwxLGxvd2VyLnRhaWwgPSBGQUxTRSkpDQojc3VtbWFyeShzdGVwLjJwN2YpDQpgYGANCiMgUmVmaXRzDQpgYGB7ciBPTFMtcmVydW4gd2l0aCBSMn0NCg0KI0ZpdCBmb3IgUmVsYXRpdmUgQmlhcyB2cy4gc2lnbWEgZm9yIEFsdGVybmF0aXZlIE1ldGhvZA0KICAjQmFzZWxpbmUgZml0IHdpdGggYWxsIHZhcmlhYmxlcyBpbmNsdWRlZA0KZml0MnA1ciA8LSBsbShSZWxCaWFzX2FsdH5wb2x5KHNpZ21hLDIscmF3PVQpKw0KICAgICAgICAgICAgICAgQyhhcy5mYWN0b3IobG9nYmFzZSksYmFzZSA9IDMpK2xvZ2Jhc2U6c2lnbWErDQogICAgICAgICAgICAgICBiZXRhK2JldGE6c2lnbWErDQogICAgICAgICAgICAgICBtZWRsaXN0K21lZGxpc3Q6c2lnbWErDQogICAgICAgICAgICAgICBub2JzK25vYnM6c2lnbWErDQogICAgICAgICAgICAgICByc3F1YXJlK3JzcXVhcmU6c2lnbWEsIGRhdGEgPSBscmV4YmV0YXJlZykgDQogICNNaW5pbWl6ZSBBSUMgYmFzZWQgb24gYSBrID0gcCA8IDAuMDUgaW4gQ2hpLXNxIHRlc3QNCnN0ZXAuMnA1ciA8LSBzdGVwQUlDKGZpdDJwNSxkaXJlY3Rpb24gPSAiYm90aCIsdHJhY2UgPSBGLCANCiAgICAgICAgICAgICAgICAgICAgayA9IHFjaGlzcSgwLjA1LDEsbG93ZXIudGFpbCA9IEZBTFNFKSkgDQojIHN1bW1hcnkoc3RlcC4ycDVyKQ0KICAjQmFzZWQgb24gdmFyaWFibGVzIHJldGFpbmVkIGFib3ZlLCBnZW5lcmF0ZSBmaXRzIGZvciB0aGUgbG93ZXIgYW5kIHVwcGVyIGNvbmZpZGVuY2UgaW50ZXJ2YWxzDQpmaXQycDVsciA8LSBsbShSZWxCaWFzX2FsdGx+cG9seShzaWdtYSwyLHJhdz1UKStDKGFzLmZhY3Rvcihsb2diYXNlKSxiYXNlID0gMykrbG9nYmFzZTpzaWdtYStub2JzK25vYnM6c2lnbWEsIGRhdGEgPSBscmV4YmV0YXJlZykNCiMgc3VtbWFyeShmaXQycDVscikNCiBmaXQycDV1ciA8LSBsbShSZWxCaWFzX2FsdHV+cG9seShzaWdtYSwyLHJhdz1UKStDKGFzLmZhY3Rvcihsb2diYXNlKSxiYXNlID0gMykrbG9nYmFzZTpzaWdtYStub2JzK25vYnM6c2lnbWEsIGRhdGEgPSBscmV4YmV0YXJlZykNCiMgc3VtbWFyeShmaXQycDV1cikNCg0KI0ZpdCBmb3IgUmVsYXRpdmUgQmlhcyB2cy4gc2lnbWEgZm9yIFJvZHJpZ3Vlei1CYXJyYW5jbyBNZXRob2QNCmZpdDJwNnIgPC0gbG0oUmVsQmlhc19yYn5wb2x5KHNpZ21hLDIscmF3PVQpKw0KICAgICAgICAgICAgICAgbG9nYmFzZStsb2diYXNlOnNpZ21hKw0KICAgICAgICAgICAgICAgYmV0YStiZXRhOnNpZ21hKw0KICAgICAgICAgICAgICAgbWVkbGlzdCttZWRsaXN0OnNpZ21hKw0KICAgICAgICAgICAgICAgbm9icytub2JzOnNpZ21hKw0KICAgICAgICAgICAgICAgcnNxdWFyZStyc3F1YXJlOnNpZ21hLCBkYXRhID0gbHJleGJldGFyZWcpDQpzdGVwLjJwNnIgPC0gc3RlcEFJQyhmaXQycDYsZGlyZWN0aW9uID0gImJvdGgiLHRyYWNlID0gVCwgDQogICAgICAgICAgICAgICAgICAgIGsgPSBxY2hpc3EoMC4wNSwxLGxvd2VyLnRhaWwgPSBGQUxTRSkpDQojIHN1bW1hcnkoc3RlcC4ycDZyKQ0KZml0MnA2bHIgPC0gbG0oUmVsQmlhc19yYmx+cG9seShzaWdtYSwyLHJhdz1UKSttZWRsaXN0K3NpZ21hOm1lZGxpc3QsIGRhdGEgPSBscmV4YmV0YXJlZykNCiMgc3VtbWFyeShmaXQycDZscikNCmZpdDJwNnVyIDwtIGxtKFJlbEJpYXNfcmJ1fnBvbHkoc2lnbWEsMixyYXc9VCkrbWVkbGlzdCtzaWdtYTptZWRsaXN0LCBkYXRhID0gbHJleGJldGFyZWcpDQojIHN1bW1hcnkoZml0MnA2dXIpDQoNCiNGaXQgZm9yIFJlbGF0aXZlIEJpYXMgdnMuIHNpZ21hIGZvciBEemllcmxlbmdhIE1ldGhvZA0KZml0MnA3ciA8LSBsbShSZWxCaWFzX2R6fnBvbHkoc2lnbWEsMixyYXc9VCkrDQogICAgICAgICAgICAgICBDKGFzLmZhY3Rvcihsb2diYXNlKSxiYXNlID0gMykrbG9nYmFzZTpzaWdtYSsNCiAgICAgICAgICAgICAgIGJldGErYmV0YTpzaWdtYSsNCiAgICAgICAgICAgICAgIG1lZGxpc3QrbWVkbGlzdDpzaWdtYSsNCiAgICAgICAgICAgICAgIG5vYnMrbm9iczpzaWdtYSsNCiAgICAgICAgICAgICAgIHJzcXVhcmUrcnNxdWFyZTpzaWdtYSwgZGF0YSA9IGxyZXhiZXRhcmVnKQ0Kc3RlcC4ycDdyIDwtIHN0ZXBBSUMoZml0MnA3LGRpcmVjdGlvbiA9ICJib3RoIix0cmFjZSA9IEYsIA0KICAgICAgICAgICAgICAgICAgICBrID0gcWNoaXNxKDAuMDUsMSxsb3dlci50YWlsID0gRkFMU0UpKQ0KIyBzdW1tYXJ5KHN0ZXAuMnA3cikNCmZpdDJwN2xyIDwtIGxtKFJlbEJpYXNfZHpsfnBvbHkoc2lnbWEsMixyYXc9VCkrbm9icytub2JzOnNpZ21hLCBkYXRhID0gbHJleGJldGFyZWcpDQojIHN1bW1hcnkoZml0MnA3bHIpDQpmaXQycDd1ciA8LSBsbShSZWxCaWFzX2R6dX5wb2x5KHNpZ21hLDIscmF3PVQpK25vYnMrbm9iczpzaWdtYSwgZGF0YSA9IGxyZXhiZXRhcmVnKQ0KIyBzdW1tYXJ5KGZpdDJwN3VyKQ0KDQojV2UgdGhlbiByZXBlYXQgdGhlIHNhbWUgdGhpbmcgZm9yIHRoZSBmdWxsIG5zaW0qbnNjZW5hcmlvIGRhdGFzZXQgKHdoZXJlIGJldGEgPiAwKQ0KZml0MnA1ZnIgPC0gbG0oUmVsQmlhc19hbHR+cG9seShzaWdtYSwyLHJhdz1UKSsNCiAgICAgICAgICAgICAgICBsb2diYXNlK2xvZ2Jhc2U6c2lnbWErDQogICAgICAgICAgICAgICAgYmV0YStiZXRhOnNpZ21hKw0KICAgICAgICAgICAgICAgIG1lZGxpc3QrbWVkbGlzdDpzaWdtYSsNCiAgICAgICAgICAgICAgICBub2JzK25vYnM6c2lnbWErDQogICAgICAgICAgICAgICByc3F1YXJlK3JzcXVhcmU6c2lnbWEsIGRhdGEgPSBhbGxzaW1kYXRhW2JldGEgPiAwXSkNCnN0ZXAuMnA1ZnIgPC0gc3RlcEFJQyhmaXQycDVmLGRpcmVjdGlvbiA9ICJib3RoIix0cmFjZSA9IEYsIA0KICAgICAgICAgICAgICAgICAgICAgayA9IHFjaGlzcSgwLjA1LDEsbG93ZXIudGFpbCA9IEZBTFNFKSkNCiMgc3VtbWFyeShzdGVwLjJwNWZyKQ0KDQpmaXQycDZmciA8LSBsbShSZWxCaWFzX3JifnBvbHkoc2lnbWEsMixyYXc9VCkrDQogICAgICAgICAgICAgICAgbG9nYmFzZStsb2diYXNlOnNpZ21hKw0KICAgICAgICAgICAgICAgIGJldGErYmV0YTpzaWdtYSsNCiAgICAgICAgICAgICAgICBtZWRsaXN0K21lZGxpc3Q6c2lnbWErDQogICAgICAgICAgICAgICAgbm9icytub2JzOnNpZ21hKw0KICAgICAgICAgICAgICAgcnNxdWFyZStyc3F1YXJlOnNpZ21hLCBkYXRhID0gYWxsc2ltZGF0YVtiZXRhID4gMF0pDQpzdGVwLjJwNmZyIDwtIHN0ZXBBSUMoZml0MnA2ZixkaXJlY3Rpb24gPSAiYm90aCIsdHJhY2UgPSBGLCANCiAgICAgICAgICAgICAgICAgICAgIGsgPSBxY2hpc3EoMC4wNSwxLGxvd2VyLnRhaWwgPSBGQUxTRSkpDQojIHN1bW1hcnkoc3RlcC4ycDZmcikNCg0KZml0MnA3ZnIgPC0gbG0oUmVsQmlhc19ken5wb2x5KHNpZ21hLDIscmF3PVQpKw0KICAgICAgICAgICAgICAgIGxvZ2Jhc2UrbG9nYmFzZTpzaWdtYSsNCiAgICAgICAgICAgICAgICBiZXRhK2JldGE6c2lnbWErDQogICAgICAgICAgICAgICAgbWVkbGlzdCttZWRsaXN0OnNpZ21hKw0KICAgICAgICAgICAgICAgIG5vYnMrbm9iczpzaWdtYSsNCiAgICAgICAgICAgICAgIHJzcXVhcmUrcnNxdWFyZTpzaWdtYSwgZGF0YSA9IGFsbHNpbWRhdGFbYmV0YSA+IDBdKQ0Kc3RlcC4ycDdmciA8LSBzdGVwQUlDKGZpdDJwN2YsZGlyZWN0aW9uID0gImJvdGgiLHRyYWNlID0gRiwgDQogICAgICAgICAgICAgICAgICAgICBrID0gcWNoaXNxKDAuMDUsMSxsb3dlci50YWlsID0gRkFMU0UpKQ0KI3N1bW1hcnkoc3RlcC4ycDdmcikNCg0KIyBzdW1tYXJ5KGFsbHNpbWRhdGEkcnNxdWFyZSkNCmBgYA0KDQpUaG9zZSBtb2RlbHMgY2FuIHRoZW4gYmUgdXNlZCB0byBndWlkZSBob3cgdGhlIGZpdHMgYXJlIGRpc3BsYXllZCBpbiBGaWd1cmUgMg0KDQpgYGB7ciBmaWcycGxvdCwgbWVzc2FnZSA9IEZBTFNFLCB3YXJuaW5nPUZBTFNFfQ0KDQpmaWcyYyA8LSBnZ3Bsb3QoZGF0YSA9IGxyZXhiZXRhcmVnLCBhZXMoeCA9IHNpZ21hLCB5ID0gUmVsQmlhc19hbHQpKSsNCiAgZ2VvbV9qaXR0ZXIoYWVzKHNoYXBlID0gYXMuZmFjdG9yKGxvZ2Jhc2UpLCBzaXplID0gbWVkbGlzdA0KICAgICAgICAgICAgICAgICAgLCBmaWxsID0gYXMuY2hhcmFjdGVyKG5vYnMpDQogICAgICAgICAgICAgICAgICApDQogICAgICAgICAgICAgICwgY29sb3IgPSAiYmxhY2siDQogICAgICAgICAgICAgICkrDQogIGdlb21fc21vb3RoKGRhdGEgPSBscmV4YmV0YXJlZywgbWV0aG9kID0gJ2xtJywNCiAgICAgICAgICAgICAgZm9ybXVsYSA9IHl+cG9seSh4LDIscmF3PVQpLA0KICAgICAgICAgICAgICBhZXMoc2hhcGUgPSBhcy5mYWN0b3IobG9nYmFzZSksDQogICAgICAgICAgICAgICAgICBjb2xvciA9IGFzLmNoYXJhY3Rlcihub2JzKSwNCiAgICAgICAgICAgICAgICAgIGxpbmV0eXBlID0gYXMuZmFjdG9yKGxvZ2Jhc2UpKSwNCiAgICAgICAgICAgICAgYWxwaGEgPSAwLjAsDQogICAgICAgICAgICAgIHNpemUgPSAxLjEpICsNCiAgc2NhbGVfeF9jb250aW51b3VzKG5hbWUgPSAiU2lnbWEiLGJyZWFrcyA9IGMoMC4yNSwwLjQ1LDAuNjUsMC44NSkpKw0KICBzY2FsZV95X2NvbnRpbnVvdXMobmFtZSA9ICJSZWxhdGl2ZSBCaWFzIFZhbHVlIChBbHRlcm5hdGl2ZSBNZXRob2QpIiwgbGltID0gYygtMC43NSwxLjI1KSkrDQogIHNjYWxlX2NvbG9yX21hbnVhbChuYW1lID0gIk51bWJlciBvZiBPYnNlcnZhdGlvbnMgKCsgRml0dGVkIEN1cnZlcykiLA0KICAgICAgICAgICAgICAgICAgICAgdmFsdWVzID0gYygiI0Q1NUUwMCIsIiM1NkI0RTkiKSwNCiAgICAgICAgICAgICAgICAgICAgIGd1aWRlID0gIm5vbmUiDQogICAgICAgICAgICAgICAgICAgICApKw0KICBzY2FsZV9maWxsX21hbnVhbChuYW1lID0gIk51bWJlciBvZiBPYnNlcnZhdGlvbnNcbigrIEZpdHRlZCBDdXJ2ZXMpIiwNCiAgICAgICAgICAgICAgICAgICAgdmFsdWVzID0gYWxwaGEoYygiI0Q1NUUwMCIsIiM1NkI0RTkiKSxjKDEsMSkpKSsNCiAgc2NhbGVfc2hhcGVfbWFudWFsKG5hbWUgPSAiTG9nYXJpdGhtIEJhc2UiLA0KICAgICAgICAgICAgICAgICAgICAgICBicmVha3MgPSBjKDIsImUiLDEwKSwNCiAgICAgICAgICAgICAgICAgICAgICAgbGFiZWxzID0gYygiMiIsImUiLCIxMCIpLA0KICAgICAgICAgICAgICAgICAgICAgICAjIHZhbHVlcyA9IGMoMTUsMTgsMjApDQogICAgICAgICAgICAgICAgICAgICAgIHZhbHVlcyA9IGMoMjEsMjIsMjQpDQogICAgICAgICAgICAgICAgICAgICApKw0KICBzY2FsZV9saW5ldHlwZV9tYW51YWwobmFtZSA9ICJGaXR0ZWQgQ3VydmVzIGZvciBMb2dhcml0aG0gQmFzZSIsDQogICAgICAgICAgICAgICAgICAgICAgIGJyZWFrcyA9IGMoMTAsImUiLDIpLA0KICAgICAgICAgICAgICAgICAgICAgICBsYWJlbHMgPSBjKCIxMCIsImUiLCIyIiksDQogICAgICAgICAgICAgICAgICAgICAgIHZhbHVlcyA9IGMoMywyLDEpKSsNCiAgc2NhbGVfc2l6ZShyYW5nZSA9IGMoMSw0KSxuYW1lID0gIk1lZGlhbiBWYWx1ZSIsDQogICAgICAgICAgICAgZ3VpZGUgPSBndWlkZV9sZWdlbmQocmV2ZXJzZSA9IFRSVUUpKSArDQogIGdlb21fdmxpbmUoeGludGVyY2VwdCA9IHNpZ21hKSsNCiAgdGhlbWVfYncoKSsNCiAgZ3VpZGVzKHNpemUgPSBndWlkZV9sZWdlbmQob3JkZXIgPSAxLCByZXZlcnNlID0gVFJVRSksDQogICAgICAgICBzaGFwZSA9IGd1aWRlX2xlZ2VuZChvcmRlciA9IDIsDQogICAgICAgICAgICAgICAgICAgICAgICAgICAgICBvdmVycmlkZS5hZXMgPSBsaXN0KHNpemUgPSAzKSksDQogICAgICAgICBmaWxsID0gZ3VpZGVfbGVnZW5kKG9yZGVyID0gNCwNCiAgICAgICAgICAgICAgICAgICAgICAgICAgICAgcmV2ZXJzZSA9IFRSVUUsDQogICAgICAgICAgICAgICAgICAgICAgICAgICAgIG92ZXJyaWRlLmFlcyA9IGxpc3QoY29sb3IgPSBjKCIjNTZCNEU5IiwiI0Q1NUUwMCIpLA0KICAgICAgICAgICAgICAgICAgICAgICAgICAgICAgICAgICAgICAgICAgICAgICAgIGFscGhhID0gMSwNCiAgICAgICAgICAgICAgICAgICAgICAgICAgICAgICAgICAgICAgICAgICAgICAgICBzaXplID0gNiwNCiAgICAgICAgICAgICAgICAgICAgICAgICAgICAgICAgICAgICAgICAgICAgICAgICBzaGFwZSA9IDE1KSksDQogICAgICAgICBsaW5ldHlwZSA9IGd1aWRlX2xlZ2VuZChvcmRlciA9IDMsIHJldmVyc2UgPSBUUlVFLCANCiAgICAgICAgICAgICAgICAgICAgICAgICAgICAgICAgIG92ZXJyaWRlLmFlcyA9IGxpc3QoY29sb3IgPSAiYmxhY2siKQ0KICAgICAgICAgICAgICAgICAgICAgICAgICAgICAgICAgKSkrDQogIHRoZW1lKGF4aXMudGl0bGUgPSBlbGVtZW50X3RleHQoc2l6ZSA9IDE0LCBmYWNlID0gImJvbGQiKSwNCiAgICAgICAgYXhpcy50ZXh0ID0gZWxlbWVudF90ZXh0KHNpemUgPSAxMiwgZmFjZSA9ICJib2xkIiksDQogICAgICAgIGxlZ2VuZC50aXRsZSA9IGVsZW1lbnRfdGV4dChzaXplID0gMTIsIGZhY2UgPSAiYm9sZCIpLA0KICAgICAgICBsZWdlbmQudGV4dCA9IGVsZW1lbnRfdGV4dChzaXplID0gMTIsIGZhY2UgPSAiYm9sZCIpLA0KICAgICAgICBsZWdlbmQua2V5LndpZHRoID0gdW5pdCgzLCJsaW5lIikpDQoNCg0KZmlnMmEgPC0gZ2dwbG90KGRhdGEgPSBscmV4YmV0YXJlZywgYWVzKHggPSBzaWdtYSwgeSA9IFJlbEJpYXNfcmIpKSsNCiAgZ2VvbV9qaXR0ZXIoYWVzKHNoYXBlID0gYXMuZmFjdG9yKGxvZ2Jhc2UpLCBzaXplID0gbWVkbGlzdCwgZmlsbCA9IGFzLmNoYXJhY3Rlcihub2JzKSksDQogICAgICAgICAgICAgIGNvbG9yID0gImJsYWNrIg0KICAgICAgICAgICAgICAjICwgYWxwaGEgPSAwLjUNCiAgICAgICAgICAgICAgKSsNCiAgZ2VvbV9zbW9vdGgoZGF0YSA9IGxyZXhiZXRhcmVnLCBtZXRob2QgPSAnbG0nLA0KICAgICAgICAgICAgICBmb3JtdWxhID0geX5wb2x5KHgsMixyYXc9VCksDQogICAgICAgICAgICAgIGFlcyhsaW5ldHlwZSA9IGFzLmZhY3RvcihtZWRsaXN0KSksDQogICAgICAgICAgICAgIGFscGhhID0gMC4wLA0KICAgICAgICAgICAgICBjb2xvciA9ICJibHVlIikgKw0KICBzY2FsZV94X2NvbnRpbnVvdXMobmFtZSA9ICJTaWdtYSIsYnJlYWtzID0gYygwLjI1LDAuNDUsMC42NSwwLjg1KSkrDQogIHNjYWxlX3lfY29udGludW91cyhuYW1lID0gIlJlbGF0aXZlIEJpYXMgVmFsdWUgKFJvZHJpZ3Vlei1CYXJyYW5jbyBNZXRob2QpIiwgbGltID0gYygtMC43NSwxLjI1KSkrDQogIHNjYWxlX3NoYXBlX21hbnVhbChuYW1lID0gIkxvZ2FyaXRobSBCYXNlIiwNCiAgICAgICAgICAgICAgICAgICAgICAgYnJlYWtzID0gYygyLCJlIiwxMCksDQogICAgICAgICAgICAgICAgICAgICAgIGxhYmVscyA9IGMoIjIiLCJlIiwiMTAiKSwNCiAgICAgICAgICAgICAgICAgICAgICAgdmFsdWVzID0gYygyMSwyMiwyNCkNCiAgICAgICAgICAgICAgICAgICAgICkrDQogIHNjYWxlX2ZpbGxfbWFudWFsKG5hbWUgPSAiTnVtYmVyIG9mIE9ic2VydmF0aW9ucyIsDQogICAgICAgICAgICAgICAgICAgIHZhbHVlcyA9IGMoIiNENTVFMDAiLCIjNTZCNEU5IikpKw0KICBzY2FsZV9saW5ldHlwZV9kaXNjcmV0ZShuYW1lID0gIkZpdHRlZCBDdXJ2ZXMgZm9yIE1lZGlhbiBWYWx1ZXMiKSsNCiAgc2NhbGVfc2l6ZShyYW5nZSA9IGMoMSw0KSwNCiAgICAgICAgICAgICBuYW1lID0gIk1lZGlhbiBWYWx1ZSIsDQogICAgICAgICAgICAgZ3VpZGUgPSBndWlkZV9sZWdlbmQocmV2ZXJzZSA9IFRSVUUpKSArDQogIGdlb21fdmxpbmUoeGludGVyY2VwdCA9IHNpZ21hKSsNCiAgdGhlbWVfYncoKSsNCiAgZ3VpZGVzKHNpemUgPSBndWlkZV9sZWdlbmQob3JkZXIgPSAyLCByZXZlcnNlID0gVFJVRSksDQogICAgICAgICBzaGFwZSA9IGd1aWRlX2xlZ2VuZChvcmRlciA9IDMsDQogICAgICAgICAgICAgICAgICAgICAgICAgICAgICBvdmVycmlkZS5hZXMgPSBsaXN0KHNpemUgPSAzKSksDQogICAgICAgICBmaWxsID0gZ3VpZGVfbGVnZW5kKG9yZGVyID0gNCwgcmV2ZXJzZSA9IFRSVUUsDQogICAgICAgICAgICAgICAgICAgICAgICAgICAgICBvdmVycmlkZS5hZXMgPSBsaXN0KHNoYXBlID0gMTUsIA0KICAgICAgICAgICAgICAgICAgICAgICAgICAgICAgICAgICAgICAgICAgICAgICAgICBzaXplID0gNiwgDQogICAgICAgICAgICAgICAgICAgICAgICAgICAgICAgICAgICAgICAgICAgICAgICAgIGNvbG9yID0gYygiIzU2QjRFOSIsIiNENTVFMDAiKSwNCiAgICAgICAgICAgICAgICAgICAgICAgICAgICAgICAgICAgICAgICAgICAgICAgICAgYWxwaGEgPSAxKSksDQogICAgICAgICBsaW5ldHlwZSA9IGd1aWRlX2xlZ2VuZChvcmRlciA9IDEsIHJldmVyc2UgPSBUUlVFKSkrDQogIHRoZW1lKGF4aXMudGl0bGUgPSBlbGVtZW50X3RleHQoc2l6ZSA9IDE0LCBmYWNlID0gImJvbGQiKSwNCiAgICAgICAgYXhpcy50ZXh0ID0gZWxlbWVudF90ZXh0KHNpemUgPSAxMiwgZmFjZSA9ICJib2xkIiksDQogICAgICAgIGxlZ2VuZC50aXRsZSA9IGVsZW1lbnRfdGV4dChzaXplID0gMTIsIGZhY2UgPSAiYm9sZCIpLA0KICAgICAgICBsZWdlbmQudGV4dCA9IGVsZW1lbnRfdGV4dChzaXplID0gMTIsIGZhY2UgPSAiYm9sZCIpLA0KICAgICAgICBsZWdlbmQua2V5LndpZHRoID0gdW5pdCgzLCJsaW5lIikpDQoNCmZpZzJiIDwtIGdncGxvdChkYXRhID0gbHJleGJldGFyZWcsIGFlcyh4ID0gc2lnbWEsIHkgPSBSZWxCaWFzX2R6KSkrIA0KICBnZW9tX2ppdHRlcihhZXMoc2hhcGUgPSBhcy5mYWN0b3IobG9nYmFzZSksIHNpemUgPSBtZWRsaXN0LCBmaWxsID0gYXMuY2hhcmFjdGVyKG5vYnMpKSwNCiAgICAgICAgICAgICAgY29sb3IgPSAiYmxhY2siDQogICAgICAgICAgICAgICkrDQogIGdlb21fc21vb3RoKGRhdGEgPSBscmV4YmV0YXJlZywgbWV0aG9kID0gJ2xtJywgDQogICAgICAgICAgICAgIGZvcm11bGEgPSB5fnBvbHkoeCwyLHJhdz1UKSwNCiAgICAgICAgICAgICAgYWVzKGNvbG9yID0gYXMuZmFjdG9yKG5vYnMpKSxhbHBoYSA9IDAuMCkgKyANCiAgc2NhbGVfeF9jb250aW51b3VzKG5hbWUgPSAiU2lnbWEiLGJyZWFrcyA9IGMoMC4yNSwwLjQ1LDAuNjUsMC44NSkpKw0KICBzY2FsZV95X2NvbnRpbnVvdXMobmFtZSA9ICJSZWxhdGl2ZSBCaWFzIFZhbHVlIChEemllcmxlbmdhIE1ldGhvZCkiLCBsaW0gPSBjKC0wLjc1LDEuMjUpKSsNCiAgc2NhbGVfY29sb3JfbWFudWFsKG5hbWUgPSAiTnVtYmVyIG9mIE9ic2VydmF0aW9ucyIsDQogICAgICAgICAgICAgICAgICAgICB2YWx1ZXMgPSBjKCIjRDU1RTAwIiwiIzU2QjRFOSIpLA0KICAgICAgICAgICAgICAgICAgICAgZ3VpZGUgPSAibm9uZSIjZ3VpZGVfbGVnZW5kKHJldmVyc2UgPSBUUlVFKQ0KICAgICAgICAgICAgICAgICAgICAgKSsNCiAgc2NhbGVfZmlsbF9tYW51YWwobmFtZSA9ICJOdW1iZXIgb2YgT2JzZXJ2YXRpb25zXG4oKyBGaXR0ZWQgQ3VydmVzKSIsDQogICAgICAgICAgICAgICAgICAgIHZhbHVlcyA9IGFscGhhKGMoIiNENTVFMDAiLCIjNTZCNEU5IiksYygxLDEpKSkrDQogIHNjYWxlX3NoYXBlX21hbnVhbChuYW1lID0gIkxvZ2FyaXRobSBCYXNlIiwNCiAgICAgICAgICAgICAgICAgICAgICAgYnJlYWtzID0gYygyLCJlIiwxMCksDQogICAgICAgICAgICAgICAgICAgICAgIGxhYmVscyA9IGMoIjIiLCJlIiwiMTAiKSwNCiAgICAgICAgICAgICAgICAgICAgICAgdmFsdWVzID0gYygyMSwyMiwyNCkpKw0KICBzY2FsZV9zaXplKHJhbmdlID0gYygxLDQpLG5hbWUgPSAiTWVkaWFuIFZhbHVlIiwNCiAgICAgICAgICAgICBndWlkZSA9IGd1aWRlX2xlZ2VuZChyZXZlcnNlID0gVFJVRSkpICsNCiAgZ2VvbV92bGluZSh4aW50ZXJjZXB0ID0gc2lnbWEpKw0KICB0aGVtZV9idygpKw0KICBndWlkZXMoc2l6ZSA9IGd1aWRlX2xlZ2VuZChvcmRlciA9IDEsIHJldmVyc2UgPSBUUlVFKSwNCiAgICAgICAgIHNoYXBlID0gZ3VpZGVfbGVnZW5kKG9yZGVyID0gMiwNCiAgICAgICAgICAgICAgICAgICAgICAgICAgICAgIG92ZXJyaWRlLmFlcyA9IGxpc3Qoc2l6ZSA9IDMpKSwNCiAgICAgICAgIGZpbGwgPSBndWlkZV9sZWdlbmQob3JkZXIgPSAzLA0KICAgICAgICAgICAgICAgICAgICAgICAgICAgICByZXZlcnNlID0gVFJVRSwNCiAgICAgICAgICAgICAgICAgICAgICAgICAgICAgb3ZlcnJpZGUuYWVzID0gbGlzdChjb2xvciA9IGMoIiM1NkI0RTkiLCIjRDU1RTAwIiksDQogICAgICAgICAgICAgICAgICAgICAgICAgICAgICAgICAgICAgICAgICAgICAgICAgYWxwaGEgPSAxLA0KICAgICAgICAgICAgICAgICAgICAgICAgICAgICAgICAgICAgICAgICAgICAgICAgIHNpemUgPSA2LA0KICAgICAgICAgICAgICAgICAgICAgICAgICAgICAgICAgICAgICAgICAgICAgICAgIHNoYXBlID0gMTUpKSkrDQogIHRoZW1lKGF4aXMudGl0bGUgPSBlbGVtZW50X3RleHQoc2l6ZSA9IDE0LCBmYWNlID0gImJvbGQiKSwgDQogICAgICAgIGF4aXMudGV4dCA9IGVsZW1lbnRfdGV4dChzaXplID0gMTIsIGZhY2UgPSAiYm9sZCIpLA0KICAgICAgICBsZWdlbmQudGl0bGUgPSBlbGVtZW50X3RleHQoc2l6ZSA9IDEyLCBmYWNlID0gImJvbGQiKSwNCiAgICAgICAgbGVnZW5kLnRleHQgPSBlbGVtZW50X3RleHQoc2l6ZSA9IDEyLCBmYWNlID0gImJvbGQiKSwNCiAgICAgICAgbGVnZW5kLmtleS53aWR0aCA9IHVuaXQoMywibGluZSIpKQ0KDQojQ3JlYXRpbmcgYSBkYXRhZnJhbWUgd2l0aCB0aGUgc3BlY2lmaWMgc2NlbmFyaW9zIG9mIGludGVyZXN0IGFuZCBjb252ZXJ0aW5nIGl0DQojZnJvbSB3aWRlIHRvIGxvbmcgZm9yIEZpZ3VyZSAyRA0KbHJleGJldGF0YWJzdWIgPC0gbHJleGJldGF0YWJbYmV0YSA9PSAwLjUgJiBtZWRsaXN0ICVpbiUgYygwLjUsOCkmbG9nYmFzZSAlaW4lIGMoMiwxMCksXQ0KbHJleGJldGFsb25nIDwtIG1lbHQobHJleGJldGF0YWJzdWIsbWVhc3VyZSA9IGMoIlJlbEJpYXNfcmIiLCJSZWxCaWFzX2FsdCIsIlJlbEJpYXNfZHoiKSkNCg0KZmlnMmQgPC0gZ2dwbG90KGRhdGEgPSBscmV4YmV0YWxvbmcsIA0KICAgICAgICAgICAgICAgYWVzKHggPSBzaWdtYSwgeSA9IHZhbHVlLCBmaWxsID0gdmFyaWFibGUpKSsNCiAgZ2VvbV9qaXR0ZXIoc2hhcGUgPSAyMSwNCiAgICAgICAgICAgICAgY29sb3IgPSAiYmxhY2siLA0KICAgICAgICAgICAgICBzaXplID0gMw0KICAgICAgICAgICAgICAjICwgYWxwaGEgPSAwLjUNCiAgICAgICAgICAgICAgKSsNCiAgZ2VvbV9zbW9vdGgoZGF0YSA9IGxyZXhiZXRhbG9uZ1tyZWwgPT0gImxvZyIgJiAodmFyaWFibGUgJWluJSBjKCJSZWxCaWFzX2R6IikpXSwNCiAgICAgICAgICAgICAgbWV0aG9kID0gJ2xtJywgDQogICAgICAgICAgICAgIGZvcm11bGEgPSB5fnBvbHkoeCwyLHJhdz1UKSwgDQogICAgICAgICAgICAgIGFscGhhID0gMC4wLA0KICAgICAgICAgICAgICBjb2xvciA9ICIjMDA5RTczIiwNCiAgICAgICAgICAgICAgc2l6ZSA9IDEuNSkgKw0KICBnZW9tX3Ntb290aChkYXRhID0gbHJleGJldGFsb25nW3JlbCA9PSAibG9nIiAmICh2YXJpYWJsZSAlaW4lIGMoIlJlbEJpYXNfYWx0IikpXSwNCiAgICAgICAgICAgICAgbWV0aG9kID0gJ2xtJywgDQogICAgICAgICAgICAgIGZvcm11bGEgPSB5fnBvbHkoeCwyLHJhdz1UKSwNCiAgICAgICAgICAgICAgYWVzKGxpbmV0eXBlID0gbG9nYmFzZSksIA0KICAgICAgICAgICAgICBhbHBoYSA9IDAuMCwNCiAgICAgICAgICAgICBjb2xvciA9ICIjQ0M3OUE3IiwNCiAgICAgICAgICAgICBzaXplID0gMS41KSsgDQogIHNjYWxlX3hfY29udGludW91cyhuYW1lID0gIlNpZ21hIixicmVha3MgPSBjKDAuMjUsMC40NSwwLjY1LDAuODUpKSsNCiAgc2NhbGVfeV9jb250aW51b3VzKG5hbWUgPSAiUmVsYXRpdmUgQmlhcyBWYWx1ZSIsIGxpbSA9IGMoLTAuNzUsMS4yNSkpKw0KICBzY2FsZV9jb2xvcl9tYW51YWwobmFtZSA9ICJSZS1leHByZXNzaW9uXG5BbGdvcml0aG0iLA0KICAgICAgICAgICAgICAgICAgICAgICBicmVha3MgPSBjKCJSZWxCaWFzX3JiIiwiUmVsQmlhc19keiIsIlJlbEJpYXNfYWx0IiksDQogICAgICAgICAgICAgICAgICAgICAgIGxhYmVscyA9IGMoZXhwcmVzc2lvbigiXHUwM2IyIltSQl0pLA0KICAgICAgICAgICAgICAgICAgICAgICAgICAgICAgICAgIGV4cHJlc3Npb24oIlx1MDNiMiJbRHpdKSwNCiAgICAgICAgICAgICAgICAgICAgICAgICAgICAgICAgICBleHByZXNzaW9uKCJcdTAzYjIiW0FsdF0pKSwNCiAgICAgICAgICAgICAgICAgICAgICAgdmFsdWVzID0gYygiI0U2OUYwMCIsIiMwMDlFNzMiLCIjQ0M3OUE3IiksDQogICAgICAgICAgICAgICAgICAgICAgIGd1aWRlID0gIm5vbmUiKSsNCiAgICBzY2FsZV9maWxsX21hbnVhbChuYW1lID0gIlJlLWV4cHJlc3Npb25cbkFsZ29yaXRobSIsDQogICAgICAgICAgICAgICAgICAgICAgYnJlYWtzID0gYygiUmVsQmlhc19yYiIsIlJlbEJpYXNfZHoiLCJSZWxCaWFzX2FsdCIpLA0KICAgICAgICAgICAgICAgICAgICAgIGxhYmVscyA9IGMoZXhwcmVzc2lvbihpdGFsaWMoIlx1MDNiMiIpW1JCXSksDQogICAgICAgICAgICAgICAgICAgICAgICAgICAgICAgICBleHByZXNzaW9uKGl0YWxpYygiXHUwM2IyIilbRHpdKSwNCiAgICAgICAgICAgICAgICAgICAgICAgICAgICAgICAgIGV4cHJlc3Npb24oaXRhbGljKCJcdTAzYjIiKVtBbHRdKSksDQogICAgICAgICAgICAgICAgICAgICAgdmFsdWVzID0gYygiI0U2OUYwMCIsIiMwMDlFNzMiLCIjQ0M3OUE3IikNCiAgICAgICAgICAgICAgICAgICAgICApKw0KICBzY2FsZV9saW5ldHlwZV9tYW51YWwobmFtZSA9ICJMb2cgQmFzZSAoQWx0ZXJuYXRpdmVcblJlZ3Jlc3Npb24gRXN0aW1hdG9yKSIsDQogICAgICAgICAgICAgICAgICAgICAgICBndWlkZSA9IGd1aWRlX2xlZ2VuZChyZXZlcnNlID0gVFJVRSksDQogICAgICAgICAgICAgICAgICAgICAgICB2YWx1ZXMgPSBjKDEsNCkpKw0KICBuZXdfc2NhbGUoImxpbmV0eXBlIikrDQogICAgZ2VvbV9zbW9vdGgoZGF0YSA9IGxyZXhiZXRhbG9uZ1tyZWwgPT0gImxvZyIgJiAodmFyaWFibGUgPT0gIlJlbEJpYXNfcmIiKV0sDQogICAgICAgICAgICAgIGNvbG9yID0gIiNFNjlGMDAiLCBtZXRob2QgPSAibG0iLGZvcm11bGEgPSB5fnBvbHkoeCwyLHJhdz1UKSwNCiAgICAgICAgICAgICAgYWVzKGxpbmV0eXBlID0gYXMuZmFjdG9yKG1lZGxpc3QpKSwgYWxwaGEgPSAwLjAsIHNpemUgPSAxLjUpKw0KICAgIHNjYWxlX2xpbmV0eXBlX2Rpc2NyZXRlKG5hbWUgPSAiTWVkaWFuIChSb2RyaWd1ZXotQmFycmFuY29cblJlZ3Jlc3Npb24gRXN0aW1hdG9yKSIsDQogICAgICAgICAgICAgZ3VpZGUgPSBndWlkZV9sZWdlbmQocmV2ZXJzZSA9IFRSVUUpKSsNCiAgZ2VvbV92bGluZSh4aW50ZXJjZXB0ID0gc2lnbWEpKw0KICB0aGVtZV9idygpKw0KICBndWlkZXMoZmlsbCA9IGd1aWRlX2xlZ2VuZChvcmRlciA9IDEsDQogICAgICAgICAgICAgICAgICAgICAgICAgICAgIG92ZXJyaWRlLmFlcyA9IGxpc3QoY29sb3IgPSBjKCIjRTY5RjAwIiwiIzAwOUU3MyIsIiNDQzc5QTciKSkNCiAgICAgICAgICAgICAgICAgICAgICAgICAgICAgKSkrDQogIHRoZW1lKGF4aXMudGl0bGUgPSBlbGVtZW50X3RleHQoc2l6ZSA9IDE0LCBmYWNlID0gImJvbGQiKSwgDQogICAgICAgIGF4aXMudGV4dCA9IGVsZW1lbnRfdGV4dChzaXplID0gMTIsIGZhY2UgPSAiYm9sZCIpLA0KICAgICAgICBsZWdlbmQudGl0bGUgPSBlbGVtZW50X3RleHQoc2l6ZSA9IDEyLCBmYWNlID0gImJvbGQiKSwNCiAgICAgICAgbGVnZW5kLnRleHQgPSBlbGVtZW50X3RleHQoc2l6ZSA9IDEyLCBmYWNlID0gImJvbGQiKSwNCiAgICAgICAgbGVnZW5kLmtleS53aWR0aCA9IHVuaXQoMywibGluZSIpKQ0KZ2dhcnJhbmdlKGZpZzJhLGZpZzJiLGZpZzJjLGZpZzJkLG5yb3cgPSAyKQ0KDQojIENyZWF0ZXMgZmlndXJlIDItLSBVbmNvbW1lbnQgdG8gZ2VuZXJhdGUNCiMgcGRmKCJmaWd1cmUyLnBkZiIsd2lkdGggPSAxNSwgaGVpZ2h0ID0gOCkNCiMgZ2dwdWJyOjpnZ2FycmFuZ2UoZmlnMmEsZmlnMmIsIGxhYmVscyA9IGMoIkEiLCJCIiksIGFsaWduID0gInYiKQ0KIyBnZ3B1YnI6OmdnYXJyYW5nZShmaWcyYyxmaWcyZCwgbGFiZWxzID0gYygiQyIsIkQiKSwgYWxpZ24gPSAidiIpDQojIGRldi5vZmYoKQ0KDQpscmV4YmV0YXJlZyRyc3FjYXQgPC0gMA0KbHJleGJldGFyZWckcnNxY2F0IDwtIGlmZWxzZShscmV4YmV0YXJlZyRyc3F1YXJlIDwgMC4wNywiPDAuMDciLA0KICAgICAgICAgICAgICAgICAgICAgICAgICAgICBpZmVsc2UobHJleGJldGFyZWckcnNxdWFyZSA+IDAuMzQsICI+MC4zNCIsIjAuMDctMC4zNCIpKQ0KDQphbHRmaWcyYSA8LSBnZ3Bsb3QoZGF0YSA9IGxyZXhiZXRhcmVnLCBhZXMoeCA9IHNpZ21hLCB5ID0gUmVsQmlhc19yYikpKw0KICBnZW9tX2ppdHRlcihhZXMoc2hhcGUgPSBhcy5mYWN0b3IobG9nYmFzZSksIHNpemUgPSBtZWRsaXN0LCBmaWxsID0gYXMuY2hhcmFjdGVyKG5vYnMpKSwNCiAgICAgICAgICAgICAgY29sb3IgPSAiYmxhY2siDQogICAgICAgICAgICAgICMgLCBhbHBoYSA9IDAuNQ0KICAgICAgICAgICAgICApKw0KIGdlb21fbGluZShkYXRhID0gbHJleGJldGFyZWcsIA0KICAgICAgICAgICAgc3RhdCA9ICdzbW9vdGgnLA0KICAgICAgICAgICAgbWV0aG9kID0gJ2xtJywNCiAgICAgICAgICAgICAgZm9ybXVsYSA9IHl+cG9seSh4LDIscmF3PVQpLA0KICAgICAgICAgICAgICBhZXMobGluZXR5cGUgPSBhcy5mYWN0b3IobWVkbGlzdCksDQogICAgICAgICAgICAgICAgICBhbHBoYSA9IHJzcWNhdCksDQogICAgICAgICAgICAgIGNvbG9yID0gImJsdWUiLA0KICAgICAgICAgICBzaXplID0gMS4xKSArDQogIHNjYWxlX3hfY29udGludW91cyhuYW1lID0gIlNpZ21hIixicmVha3MgPSBjKDAuMjUsMC40NSwwLjY1LDAuODUpKSsNCiAgc2NhbGVfeV9jb250aW51b3VzKG5hbWUgPSAiUmVsYXRpdmUgQmlhcyBWYWx1ZSAoUm9kcmlndWV6LUJhcnJhbmNvIE1ldGhvZCkiLCBsaW0gPSBjKC0xLjI1LDEuMjUpKSsNCiAgc2NhbGVfc2hhcGVfbWFudWFsKG5hbWUgPSAiTG9nYXJpdGhtIEJhc2UiLA0KICAgICAgICAgICAgICAgICAgICAgICBicmVha3MgPSBjKDIsImUiLDEwKSwNCiAgICAgICAgICAgICAgICAgICAgICAgbGFiZWxzID0gYygiMiIsImUiLCIxMCIpLA0KICAgICAgICAgICAgICAgICAgICAgICAjIHZhbHVlcyA9IGMoMTUsMTgsMjApDQogICAgICAgICAgICAgICAgICAgICAgIHZhbHVlcyA9IGMoMjEsMjIsMjQpDQogICAgICAgICAgICAgICAgICAgICApKw0KICBzY2FsZV9maWxsX21hbnVhbChuYW1lID0gIk51bWJlciBvZiBPYnNlcnZhdGlvbnMiLA0KICAgICAgICAgICAgICAgICAgICB2YWx1ZXMgPSBjKCIjRDU1RTAwIiwiIzU2QjRFOSIpKSsNCiAgc2NhbGVfbGluZXR5cGVfZGlzY3JldGUobmFtZSA9ICJGaXR0ZWQgQ3VydmVzIGZvciBNZWRpYW4gVmFsdWVzIikrDQogICAgc2NhbGVfYWxwaGFfbWFudWFsKG5hbWUgPSAiUnNxdWFyZWQgVmFsdWUiLA0KICAgICAgICAgICAgICAgICAgICAgdmFsdWVzID0gYygwLjMzLDAuNjcsMSkpKw0KICBzY2FsZV9zaXplKHJhbmdlID0gYygxLDQpLA0KICAgICAgICAgICAgICMgYnJlYWtzID0gYygwLjI1LDAuNSwxLDIsNCw4LDE2LDMyKSwNCiAgICAgICAgICAgICBuYW1lID0gIk1lZGlhbiBWYWx1ZSIsDQogICAgICAgICAgICAgZ3VpZGUgPSBndWlkZV9sZWdlbmQocmV2ZXJzZSA9IFRSVUUpKSArDQogIGdlb21fdmxpbmUoeGludGVyY2VwdCA9IHNpZ21hKSsNCiAgdGhlbWVfYncoKSsNCiAgZ3VpZGVzKHNpemUgPSBndWlkZV9sZWdlbmQob3JkZXIgPSAzLCByZXZlcnNlID0gVFJVRSksDQogICAgICAgICBzaGFwZSA9IGd1aWRlX2xlZ2VuZChvcmRlciA9IDQsDQogICAgICAgICAgICAgICAgICAgICAgICAgICAgICBvdmVycmlkZS5hZXMgPSBsaXN0KHNpemUgPSAzKSksDQogICAgICAgICBmaWxsID0gZ3VpZGVfbGVnZW5kKG9yZGVyID0gNSwgcmV2ZXJzZSA9IFRSVUUsDQogICAgICAgICAgICAgICAgICAgICAgICAgICAgICBvdmVycmlkZS5hZXMgPSBsaXN0KHNoYXBlID0gMTUsIA0KICAgICAgICAgICAgICAgICAgICAgICAgICAgICAgICAgICAgICAgICAgICAgICAgICBzaXplID0gNiwgDQogICAgICAgICAgICAgICAgICAgICAgICAgICAgICAgICAgICAgICAgICAgICAgICAgIGNvbG9yID0gYygiIzU2QjRFOSIsIiNENTVFMDAiKSwNCiAgICAgICAgICAgICAgICAgICAgICAgICAgICAgICAgICAgICAgICAgICAgICAgICAgYWxwaGEgPSAxKSksDQogICAgICAgICBsaW5ldHlwZSA9IGd1aWRlX2xlZ2VuZChvcmRlciA9IDEsIHJldmVyc2UgPSBUUlVFKSwNCiAgICAgICAgIGFscGhhID0gZ3VpZGVfbGVnZW5kKG9yZGVyID0gMikpKw0KICB0aGVtZShheGlzLnRpdGxlID0gZWxlbWVudF90ZXh0KHNpemUgPSAxNCwgZmFjZSA9ICJib2xkIiksDQogICAgICAgIGF4aXMudGV4dCA9IGVsZW1lbnRfdGV4dChzaXplID0gMTIsIGZhY2UgPSAiYm9sZCIpLA0KICAgICAgICBsZWdlbmQudGl0bGUgPSBlbGVtZW50X3RleHQoc2l6ZSA9IDEyLCBmYWNlID0gImJvbGQiKSwNCiAgICAgICAgbGVnZW5kLnRleHQgPSBlbGVtZW50X3RleHQoc2l6ZSA9IDEyLCBmYWNlID0gImJvbGQiKSwNCiAgICAgICAgbGVnZW5kLmtleS53aWR0aCA9IHVuaXQoMywibGluZSIpKQ0KDQphbHRmaWcyYiA8LSBnZ3Bsb3QoZGF0YSA9IGxyZXhiZXRhcmVnLCBhZXMoeCA9IHNpZ21hLCB5ID0gUmVsQmlhc19keikpKyANCiAgZ2VvbV9qaXR0ZXIoYWVzKHNoYXBlID0gYXMuZmFjdG9yKGxvZ2Jhc2UpLCBzaXplID0gbWVkbGlzdCwgZmlsbCA9IGFzLmNoYXJhY3Rlcihub2JzKSksDQogICAgICAgICAgICAgIGNvbG9yID0gImJsYWNrIg0KICAgICAgICAgICAgICAjICwgYWxwaGEgPSAwLjUNCiAgICAgICAgICAgICAgKSsNCiAgZ2VvbV9saW5lKGRhdGEgPSBscmV4YmV0YXJlZ1tyZWwgPT0gImxvZyJdLCANCiAgICAgICAgICAgIHN0YXQgPSAnc21vb3RoJywNCiAgICAgICAgICAgIG1ldGhvZCA9ICdsbScsDQogICAgICAgICAgICAgIGZvcm11bGEgPSB5fnBvbHkoeCwyLHJhdz1UKSwNCiAgICAgICAgICAgICAgYWVzKGFscGhhID0gcnNxY2F0LA0KICAgICAgICAgICAgICAgICAgY29sb3IgPSBhcy5jaGFyYWN0ZXIobm9icykpLA0KICAgICAgICAgICAgICBzaXplID0gMS4xKSArDQogIHNjYWxlX3hfY29udGludW91cyhuYW1lID0gIlNpZ21hIixicmVha3MgPSBjKDAuMjUsMC40NSwwLjY1LDAuODUpKSsNCiAgc2NhbGVfeV9jb250aW51b3VzKG5hbWUgPSAiUmVsYXRpdmUgQmlhcyBWYWx1ZSAoRHppZXJsZW5nYSBNZXRob2QpIiwgbGltID0gYygtMS4yNSwxLjI1KSkrDQogIHNjYWxlX2NvbG9yX21hbnVhbChuYW1lID0gIk51bWJlciBvZiBPYnNlcnZhdGlvbnMiLA0KICAgICAgICAgICAgICAgICAgICAgdmFsdWVzID0gYygiI0Q1NUUwMCIsIiM1NkI0RTkiKSwNCiAgICAgICAgICAgICAgICAgICAgIGd1aWRlID0gIm5vbmUiI2d1aWRlX2xlZ2VuZChyZXZlcnNlID0gVFJVRSkNCiAgICAgICAgICAgICAgICAgICAgICkrDQogIHNjYWxlX2FscGhhX21hbnVhbChuYW1lID0gIlJzcXVhcmVkIFZhbHVlIiwNCiAgICAgICAgICAgICAgICAgICAgIHZhbHVlcyA9IGMoMC4zMywwLjY3LDEpKSsNCiAgc2NhbGVfZmlsbF9tYW51YWwobmFtZSA9ICJOdW1iZXIgb2YgT2JzZXJ2YXRpb25zXG4oKyBGaXR0ZWQgQ3VydmVzKSIsDQogICAgICAgICAgICAgICAgICAgIHZhbHVlcyA9IGFscGhhKGMoIiNENTVFMDAiLCIjNTZCNEU5IiksYygxLDEpKSkrDQogIHNjYWxlX3NoYXBlX21hbnVhbChuYW1lID0gIkxvZ2FyaXRobSBCYXNlIiwNCiAgICAgICAgICAgICAgICAgICAgICAgYnJlYWtzID0gYygyLCJlIiwxMCksDQogICAgICAgICAgICAgICAgICAgICAgIGxhYmVscyA9IGMoIjIiLCJlIiwiMTAiKSwNCiAgICAgICAgICAgICAgICAgICAgICAgIyB2YWx1ZXMgPSBjKDE1LDE4LDIwKQ0KICAgICAgICAgICAgICAgICAgICAgICB2YWx1ZXMgPSBjKDIxLDIyLDI0KSkrDQogIHNjYWxlX3NpemUocmFuZ2UgPSBjKDEsNCksbmFtZSA9ICJNZWRpYW4gVmFsdWUiLA0KICAgICAgICAgICAgIGd1aWRlID0gZ3VpZGVfbGVnZW5kKHJldmVyc2UgPSBUUlVFKSkgKw0KICBnZW9tX3ZsaW5lKHhpbnRlcmNlcHQgPSBzaWdtYSkrDQogIHRoZW1lX2J3KCkrDQogIGd1aWRlcyhzaXplID0gZ3VpZGVfbGVnZW5kKG9yZGVyID0gMSwgcmV2ZXJzZSA9IFRSVUUpLA0KICAgICAgICAgc2hhcGUgPSBndWlkZV9sZWdlbmQob3JkZXIgPSAyLA0KICAgICAgICAgICAgICAgICAgICAgICAgICAgICAgb3ZlcnJpZGUuYWVzID0gbGlzdChzaXplID0gMykpLA0KICAgICAgICAgZmlsbCA9IGd1aWRlX2xlZ2VuZChvcmRlciA9IDMsDQogICAgICAgICAgICAgICAgICAgICAgICAgICAgIHJldmVyc2UgPSBUUlVFLA0KICAgICAgICAgICAgICAgICAgICAgICAgICAgICBvdmVycmlkZS5hZXMgPSBsaXN0KGNvbG9yID0gYygiIzU2QjRFOSIsIiNENTVFMDAiKSwNCiAgICAgICAgICAgICAgICAgICAgICAgICAgICAgICAgICAgICAgICAgICAgICAgICBhbHBoYSA9IDEsDQogICAgICAgICAgICAgICAgICAgICAgICAgICAgICAgICAgICAgICAgICAgICAgICAgc2l6ZSA9IDYsDQogICAgICAgICAgICAgICAgICAgICAgICAgICAgICAgICAgICAgICAgICAgICAgICAgc2hhcGUgPSAxNSkpKSsNCiAgdGhlbWUoYXhpcy50aXRsZSA9IGVsZW1lbnRfdGV4dChzaXplID0gMTQsIGZhY2UgPSAiYm9sZCIpLCANCiAgICAgICAgYXhpcy50ZXh0ID0gZWxlbWVudF90ZXh0KHNpemUgPSAxMiwgZmFjZSA9ICJib2xkIiksDQogICAgICAgIGxlZ2VuZC50aXRsZSA9IGVsZW1lbnRfdGV4dChzaXplID0gMTIsIGZhY2UgPSAiYm9sZCIpLA0KICAgICAgICBsZWdlbmQudGV4dCA9IGVsZW1lbnRfdGV4dChzaXplID0gMTIsIGZhY2UgPSAiYm9sZCIpLA0KICAgICAgICBsZWdlbmQua2V5LndpZHRoID0gdW5pdCgzLCJsaW5lIikpDQoNCmFsdGZpZzJjIDwtIGdncGxvdChkYXRhID0gbHJleGJldGFyZWcsIGFlcyh4ID0gc2lnbWEsIHkgPSBSZWxCaWFzX2FsdCkpKw0KICBnZW9tX2ppdHRlcihhZXMoc2hhcGUgPSBhcy5mYWN0b3IobG9nYmFzZSksIHNpemUgPSBtZWRsaXN0DQogICAgICAgICAgICAgICAgICAsIGZpbGwgPSBhcy5jaGFyYWN0ZXIobm9icykNCiAgICAgICAgICAgICAgICAgICkNCiAgICAgICAgICAgICAgLCBjb2xvciA9ICJibGFjayINCiAgICAgICAgICAgICAgIyAsIGFscGhhID0gMC42DQogICAgICAgICAgICAgICkrDQogIGdlb21fbGluZShkYXRhID0gbHJleGJldGFyZWcsIA0KICAgICAgICAgICAgc3RhdCA9ICdzbW9vdGgnLA0KICAgICAgICAgICAgbWV0aG9kID0gJ2xtJywNCiAgICAgICAgICAgICAgZm9ybXVsYSA9IHl+cG9seSh4LDIscmF3PVQpLA0KICAgICAgICAgICAgICBhZXMobGluZXR5cGUgPSBsb2diYXNlLA0KICAgICAgICAgICAgICAgICAgc2hhcGUgPSBsb2diYXNlLA0KICAgICAgICAgICAgICAgICAgYWxwaGEgPSByc3FjYXQsDQogICAgICAgICAgICAgICAgICBjb2xvciA9IGFzLmNoYXJhY3Rlcihub2JzKSksDQogICAgICAgICAgICAgIHNpemUgPSAxLjEpICsNCiAgc2NhbGVfeF9jb250aW51b3VzKG5hbWUgPSAiU2lnbWEiLGJyZWFrcyA9IGMoMC4yNSwwLjQ1LDAuNjUsMC44NSkpKw0KICBzY2FsZV95X2NvbnRpbnVvdXMobmFtZSA9ICJSZWxhdGl2ZSBCaWFzIFZhbHVlIChBbHRlcm5hdGl2ZSBNZXRob2QpIiwgbGltID0gYygtMS4yNSwxLjI1KSkrDQogIHNjYWxlX2NvbG9yX21hbnVhbChuYW1lID0gIk51bWJlciBvZiBPYnNlcnZhdGlvbnMiLA0KICAgICAgICAgICAgICAgICAgICAgdmFsdWVzID0gYygiI0Q1NUUwMCIsIiM1NkI0RTkiKSwNCiAgICAgICAgICAgICAgICAgICAgIGd1aWRlID0gIm5vbmUiDQogICAgICAgICAgICAgICAgICAgICApKw0KICBzY2FsZV9maWxsX21hbnVhbChuYW1lID0gIk51bWJlciBvZiBPYnNlcnZhdGlvbnNcbigrIEZpdHRlZCBDdXJ2ZXMpIiwNCiAgICAgICAgICAgICAgICAgICAgdmFsdWVzID0gYWxwaGEoYygiI0Q1NUUwMCIsIiM1NkI0RTkiKSxjKDEsMSkpKSsNCiAgc2NhbGVfc2hhcGVfbWFudWFsKG5hbWUgPSAiTG9nYXJpdGhtIEJhc2UiLA0KICAgICAgICAgICAgICAgICAgICAgICBicmVha3MgPSBjKDIsImUiLDEwKSwNCiAgICAgICAgICAgICAgICAgICAgICAgbGFiZWxzID0gYygiMiIsImUiLCIxMCIpLA0KICAgICAgICAgICAgICAgICAgICAgICAjIHZhbHVlcyA9IGMoMTUsMTgsMjApDQogICAgICAgICAgICAgICAgICAgICAgIHZhbHVlcyA9IGMoMjEsMjIsMjQpDQogICAgICAgICAgICAgICAgICAgICApKw0KICBzY2FsZV9hbHBoYV9tYW51YWwobmFtZSA9ICJSc3F1YXJlZCBWYWx1ZSIsDQogICAgICAgICAgICAgICAgICAgICB2YWx1ZXMgPSBjKDAuMzMsMC42NywxKSkrDQogIHNjYWxlX2xpbmV0eXBlX21hbnVhbChuYW1lID0gIkZpdHRlZCBDdXJ2ZXMgZm9yIExvZ2FyaXRobSBCYXNlIiwNCiAgICAgICAgICAgICAgICAgICAgICAgYnJlYWtzID0gYygxMCwiZSIsMiksDQogICAgICAgICAgICAgICAgICAgICAgIGxhYmVscyA9IGMoIjEwIiwiZSIsIjIiKSwNCiAgICAgICAgICAgICAgICAgICAgICAgdmFsdWVzID0gYygzLDIsMSkpKw0KICBzY2FsZV9zaXplKHJhbmdlID0gYygxLDQpLG5hbWUgPSAiTWVkaWFuIFZhbHVlIiwNCiAgICAgICAgICAgICBndWlkZSA9IGd1aWRlX2xlZ2VuZChyZXZlcnNlID0gVFJVRSkpICsNCiAgZ2VvbV92bGluZSh4aW50ZXJjZXB0ID0gc2lnbWEpKw0KICB0aGVtZV9idygpKw0KICBndWlkZXMoc2l6ZSA9IGd1aWRlX2xlZ2VuZChvcmRlciA9IDEsIHJldmVyc2UgPSBUUlVFKSwNCiAgICAgICAgIHNoYXBlID0gZ3VpZGVfbGVnZW5kKG9yZGVyID0gMiwNCiAgICAgICAgICAgICAgICAgICAgICAgICAgICAgIG92ZXJyaWRlLmFlcyA9IGxpc3Qoc2l6ZSA9IDMpKSwNCiAgICAgICAgICMgY29sb3IgPSBndWlkZV9sZWdlbmQob3JkZXIgPSA0LCByZXZlcnNlID0gVFJVRSwNCiAgICAgICAgICMgICAgICAgICAgICAgICAgICAgICAgb3ZlcnJpZGUuYWVzID0gbGlzdChjb2xvciA9IGMoIiNENTVFMDAiLCIjNTZCNEU5IiksDQogICAgICAgICAjICAgICAgICAgICAgICAgICAgICAgICAgICAgICAgICAgICAgICAgICAgc2l6ZSA9IDMpKSwNCiAgICAgICAgIGZpbGwgPSBndWlkZV9sZWdlbmQob3JkZXIgPSA1LA0KICAgICAgICAgICAgICAgICAgICAgICAgICAgICByZXZlcnNlID0gVFJVRSwNCiAgICAgICAgICAgICAgICAgICAgICAgICAgICAgb3ZlcnJpZGUuYWVzID0gbGlzdChjb2xvciA9IGMoIiM1NkI0RTkiLCIjRDU1RTAwIiksDQogICAgICAgICAgICAgICAgICAgICAgICAgICAgICAgICAgICAgICAgICAgICAgICAgYWxwaGEgPSAxLA0KICAgICAgICAgICAgICAgICAgICAgICAgICAgICAgICAgICAgICAgICAgICAgICAgIHNpemUgPSA2LA0KICAgICAgICAgICAgICAgICAgICAgICAgICAgICAgICAgICAgICAgICAgICAgICAgIHNoYXBlID0gMTUpKSwNCiAgICAgICAgIGxpbmV0eXBlID0gZ3VpZGVfbGVnZW5kKG9yZGVyID0gMywgcmV2ZXJzZSA9IFRSVUUsIA0KICAgICAgICAgICAgICAgICAgICAgICAgICAgICAgICAgb3ZlcnJpZGUuYWVzID0gbGlzdChjb2xvciA9ICJibGFjayIpDQogICAgICAgICAgICAgICAgICAgICAgICAgICAgICAgICApLA0KICAgICAgICAgYWxwaGEgPSBndWlkZV9sZWdlbmQob3JkZXIgPSA0KSkrDQogIHRoZW1lKGF4aXMudGl0bGUgPSBlbGVtZW50X3RleHQoc2l6ZSA9IDE0LCBmYWNlID0gImJvbGQiKSwNCiAgICAgICAgYXhpcy50ZXh0ID0gZWxlbWVudF90ZXh0KHNpemUgPSAxMiwgZmFjZSA9ICJib2xkIiksDQogICAgICAgIGxlZ2VuZC50aXRsZSA9IGVsZW1lbnRfdGV4dChzaXplID0gMTIsIGZhY2UgPSAiYm9sZCIpLA0KICAgICAgICBsZWdlbmQudGV4dCA9IGVsZW1lbnRfdGV4dChzaXplID0gMTIsIGZhY2UgPSAiYm9sZCIpLA0KICAgICAgICBsZWdlbmQua2V5LndpZHRoID0gdW5pdCgzLCJsaW5lIikpDQpgYGANCg0KIyBUYWJsZSAyDQoNCkZvciB0aGUgZnVsbCBuc2ltKm5zY2VuYXJpbyBkYXRhc2V0LCBjYWxjdWxhdGUgdGhlIHJlbGF0aXZlIGJpYXMgYW5kIGNvdmVyYWdlIHByb2JhYmlsaXR5IGZvciBlYWNoIGluZGl2aWR1YWwgc2ltdWxhdGlvbi4NCg0KYGBge3IgaW5kaXZjYWxjfQ0KYWxsc2ltZGF0YSRSZWxCaWFzX3JiMSA8LSAoYWxsc2ltZGF0YSRyYi1hbGxzaW1kYXRhJG9ic2VydmVkX2JldGEpL2FicyhhbGxzaW1kYXRhJG9ic2VydmVkX2JldGEpDQphbGxzaW1kYXRhJFJlbEJpYXNfZHoxIDwtIChhbGxzaW1kYXRhJGR6LWFsbHNpbWRhdGEkb2JzZXJ2ZWRfYmV0YSkvYWJzKGFsbHNpbWRhdGEkb2JzZXJ2ZWRfYmV0YSkNCmFsbHNpbWRhdGEkUmVsQmlhc19hbHQxIDwtIChhbGxzaW1kYXRhJGFsdC1hbGxzaW1kYXRhJG9ic2VydmVkX2JldGEpL2FicyhhbGxzaW1kYXRhJG9ic2VydmVkX2JldGEpDQoNCmFsbHNpbWRhdGEkY292ZXJhZ2VfcHJvYl9yYiA8LSBhbGxzaW1kYXRhJGNvdmVyYWdlX3Byb2JfZHogPC0gYWxsc2ltZGF0YSRjb3ZlcmFnZV9wcm9iX2FsdCA8LSAwDQphbGxzaW1kYXRhJGNvdmVyYWdlX3Byb2JfcmIgPC0gYygwLDEpWyhhbGxzaW1kYXRhJG9ic2VydmVkX2JldGEgPD0gYWxsc2ltZGF0YSRyYnUgJiBhbGxzaW1kYXRhJG9ic2VydmVkX2JldGEgPj0gYWxsc2ltZGF0YSRyYmwpKzFdDQphbGxzaW1kYXRhJGNvdmVyYWdlX3Byb2JfZHogPC0gYygwLDEpWyhhbGxzaW1kYXRhJG9ic2VydmVkX2JldGEgPD0gYWxsc2ltZGF0YSRkenUgJiBhbGxzaW1kYXRhJG9ic2VydmVkX2JldGEgPj0gYWxsc2ltZGF0YSRkemwpKzFdDQphbGxzaW1kYXRhJGNvdmVyYWdlX3Byb2JfYWx0IDwtIGMoMCwxKVsoYWxsc2ltZGF0YSRvYnNlcnZlZF9iZXRhIDw9IGFsbHNpbWRhdGEkYWx0dSAmIGFsbHNpbWRhdGEkb2JzZXJ2ZWRfYmV0YSA+PSBhbGxzaW1kYXRhJGFsdGwpKzFdDQpgYGANCg0KVGhlbiBUYWJsZSAyIGNhbiBiZSBnZW5lcmF0ZWQNCg0KYGBge3J9DQp0YWIyIDwtIGRhdGEuZnJhbWUoIkVzdGltYXRvciIgPSBjKCJcdTAzYjJ+UkJ+IiwNCiAgICAgICAgICAgICAgICAgICAgICAgICAgICAgICAgICAgIlx1MDNiMn5Een4iLA0KICAgICAgICAgICAgICAgICAgICAgICAgICAgICAgICAgICAiXHUwM2IyfkFsdH4iKSwNCiAgICAgICAgICAgICAgICAgICAiQXZlcmFnZSBhYnNvbHV0ZSByZWxhdGl2ZSBiaWFzIiA9IGMobWVhbihhYnMoYWxsc2ltZGF0YSRSZWxCaWFzX3JiMSksbmEucm0gPSBUKSwNCiAgICAgICAgICAgICAgICAgICAgICAgICAgICAgICAgICAgICAgICAgICAgICAgbWVhbihhYnMoYWxsc2ltZGF0YSRSZWxCaWFzX2R6MSksbmEucm0gPSBUKSwNCiAgICAgICAgICAgICAgICAgICAgICAgICAgICAgICAgICAgICAgICAgICAgICAgbWVhbihhYnMoYWxsc2ltZGF0YSRSZWxCaWFzX2FsdDEpLG5hLnJtID0gVCkpLA0KICAgICAgICAgICAgICAgICAgICJGaXJzdCBRdWFydGlsZSBhYnNvbHV0ZSByZWxhdGl2ZSBiaWFzIiA9IGMoc3VtbWFyeShhYnMoYWxsc2ltZGF0YSRSZWxCaWFzX3JiMSkpW1syXV0sDQogICAgICAgICAgICAgICAgICAgICAgICAgICAgICAgICAgICAgICAgICAgICAgIHN1bW1hcnkoYWJzKGFsbHNpbWRhdGEkUmVsQmlhc19kejEpKVtbMl1dLA0KICAgICAgICAgICAgICAgICAgICAgICAgICAgICAgICAgICAgICAgICAgICAgICBzdW1tYXJ5KGFicyhhbGxzaW1kYXRhJFJlbEJpYXNfYWx0MSkpW1syXV0pLA0KICAgICAgICAgICAgICAgICAgICJUaGlyZCBRdWFydGlsZSBhYnNvbHV0ZSByZWxhdGl2ZSBiaWFzIiA9IGMoc3VtbWFyeShhYnMoYWxsc2ltZGF0YSRSZWxCaWFzX3JiMSkpW1s1XV0sDQogICAgICAgICAgICAgICAgICAgICAgICAgICAgICAgICAgICAgICAgICAgICAgIHN1bW1hcnkoYWJzKGFsbHNpbWRhdGEkUmVsQmlhc19kejEpKVtbNV1dLA0KICAgICAgICAgICAgICAgICAgICAgICAgICAgICAgICAgICAgICAgICAgICAgICBzdW1tYXJ5KGFicyhhbGxzaW1kYXRhJFJlbEJpYXNfYWx0MSkpW1s1XV0pLA0KICAgICAgICAgICAgICAgICAgICJBdmVyYWdlIGNvdmVyYWdlIHByb2JhYmlsaXR5IiA9IGMobWVhbihhbGxzaW1kYXRhJGNvdmVyYWdlX3Byb2JfcmIsbmEucm0gPSBUKSwNCiAgICAgICAgICAgICAgICAgICAgICAgICAgICAgICAgICAgICAgICAgICAgICAgICAgICAgIG1lYW4oYWxsc2ltZGF0YSRjb3ZlcmFnZV9wcm9iX2R6LG5hLnJtID0gVCksDQogICAgICAgICAgICAgICAgICAgICAgICAgICAgICAgICAgICAgICAgICAgICAgICAgICAgICBtZWFuKGFsbHNpbWRhdGEkY292ZXJhZ2VfcHJvYl9hbHQsbmEucm0gPSBUKSkpDQpuYW1lcyh0YWIyKSA8LSBjKCJFc3RpbWF0b3IiLCJBdmVyYWdlIGFic29sdXRlIHJlbGF0aXZlIGJpYXMiLCJGaXJzdCBxdWFydGlsZSBhYnNvbHV0ZSByZWxhdGl2ZSBiaWFzIiwiVGhpcmQgUXVhcnRpbGUgYWJzb2x1dGUgcmVsYXRpdmUgYmlhcyIsIkF2ZXJhZ2UgY292ZXJhZ2UgcHJvYmFiaWxpdHkiKQ0KDQp0YWIyIDwtIGtuaXRyOjprYWJsZSh0YWIyKQ0KDQp0YWIyDQpgYGANCg0KIyBUYWJsZSAzDQoNCmBgYHtyfQ0KdGFiMm4gPC0gYWxsc2ltZGF0YVthbGxzaW1kYXRhJG5vYnMgPT0gMTYyICYgYWxsc2ltZGF0YSRiZXRhID09IDEgJiAoYWxsc2ltZGF0YSRzaWdtYSA9PSAwLjQ1IHwgYWxsc2ltZGF0YSRzaWdtYSA9PSAwLjg1KSAmIChhbGxzaW1kYXRhJG1lZGxpc3QgPT0gMiB8IGFsbHNpbWRhdGEkbWVkbGlzdCA9PSAxNikgJiAoYWxsc2ltZGF0YSRsb2diYXNlID09IDIgfCBhbGxzaW1kYXRhJGxvZ2Jhc2UgPT0gMTApXQ0KdGFiMm4gPC0gZHBseXI6OnNlbGVjdCh0YWIybixzaWdtYSxtZWRsaXN0LGxvZ2Jhc2UsUmVsQmlhc19yYixSZWxCaWFzX2R6LFJlbEJpYXNfYWx0LCBjb3ZlcmFnZV9wcm9iX3JiLCBjb3ZlcmFnZV9wcm9iX2R6LCBjb3ZlcmFnZV9wcm9iX2FsdCkNCnRhYjJuIDwtIGFnZ3JlZ2F0ZSgufnNpZ21hK21lZGxpc3QrbG9nYmFzZSwgZGF0YSA9IHRhYjJuLCBtZWFuKQ0KdGFiMm4gPC0gdGFiMm5bb3JkZXIodGFiMm4kc2lnbWEsIHRhYjJuJG1lZGxpc3QsIHRhYjJuJGxvZ2Jhc2UpLF0NCnRhYjJuMSA8LSBwaXZvdF9sb25nZXIodGFiMm4sIA0KICAgICAgICAgICAgICAgICAgICAgIGNvbHMgPSBjKCJjb3ZlcmFnZV9wcm9iX3JiIiwgImNvdmVyYWdlX3Byb2JfZHoiLCAiY292ZXJhZ2VfcHJvYl9hbHQiKSwgDQogICAgICAgICAgICAgICAgICAgICAgbmFtZXNfdG8gPSAidmFyMiIsDQogICAgICAgICAgICAgICAgICAgICAgdmFsdWVzX3RvID0gImNvdmVyYWdlX3Byb2IiKQ0KdGFiMm4gPC0gcGl2b3RfbG9uZ2VyKHRhYjJuLCANCiAgICAgICAgICAgICAgICAgICAgICBjb2xzID0gYygiUmVsQmlhc19yYiIsIlJlbEJpYXNfZHoiLCJSZWxCaWFzX2FsdCIpLCANCiAgICAgICAgICAgICAgICAgICAgICBuYW1lc190byA9ICJyZXhtZXRob2QiLA0KICAgICAgICAgICAgICAgICAgICAgIHZhbHVlc190byA9ICJSZWxCaWFzIikNCnRhYjJuJGNvdmVyYWdlX3Byb2IgPC0gdGFiMm4xJGNvdmVyYWdlX3Byb2INCnRhYjJuJHJleG1ldGhvZFtzdHJfZGV0ZWN0KHRhYjJuJHJleG1ldGhvZCwicmIiKV0gPC0gInJiIg0KdGFiMm4kcmV4bWV0aG9kW3N0cl9kZXRlY3QodGFiMm4kcmV4bWV0aG9kLCJkeiIpXSA8LSAiZHoiDQp0YWIybiRyZXhtZXRob2Rbc3RyX2RldGVjdCh0YWIybiRyZXhtZXRob2QsImFsdCIpXSA8LSAiYWx0Ig0KdGFiMm5bLDQ6Nl0gPC0gTlVMTA0KdGFiMm4gPC0gdGFiMm4gJT4lIG11dGF0ZV9hdChjKCJSZWxCaWFzIiwiY292ZXJhZ2VfcHJvYiIpLHJvdW5kLCBkaWdpdHMgPSAzKQ0KdGFiMm4kY292ZXJhZ2VfcHJvYiA8LSB0YWIybiRjb3ZlcmFnZV9wcm9iICogMTAwDQp0YWIybg0KYGBgDQoNCiMgVGFibGUgNA0KDQpXZSdyZSBmaXJzdCBwbHVnZ2luZyBpbiBlc3RpbWF0ZXMgZm9yIG1lZGlhbnMgYW5kIElRUnMgZm9yIHN0dWRpZXMgdGhhdCBkb24ndCByZXBvcnQgdGhlbS4gV2hpbGUgdGhlIER6aWVybGVuZ2EgbWV0aG9kIGRvZXNuJ3QgZXhwbGljaXRseSBuZWVkIHRoZXNlIHZhbHVlcywgZm9yIHRoZSBzYWtlIG9mIGNvbXBhcmlzb24gYWNyb3NzIG1ldGhvZHMsIGl0IHdpbGwgYWxzbyB1c2UgdGhlc2UgZXN0aW1hdGVkIHZhbHVlcyBmb3IgVGFibGUgMy4gQWRkaXRpb25hbGx5IGluIHRoaXMgc3RlcCwgbWVhbnMgYXJlIGVzdGltYXRlZCBmcm9tIG1lZGlhbnMgd2hlbiB0aGUgZm9ybWVyIGFyZW4ndCBhdmFpbGFibGUgYnV0IHRoZSBsYXR0ZXIgYXJlLg0KDQpgYGB7ciB0YWI0c2V0dXB9DQoNCiNBZGQgbWVkaWFucyBhbmQgSVFScw0KZGF0YVtkYXRhJHN0dWR5X2lkID09ICJTdGVpbiAyMDE2Iiw0OjZdIDwtIGxpc3QoMjIuMiwgMTUuMzUsIDMwLjgpDQpkYXRhW2RhdGEkc3R1ZHlfaWQgPT0gIlBpbGtlcnRvbiAyMDE4Iiw0OjZdIDwtIGxpc3QoNC4zLCAzLCA2LjMpDQpkYXRhW2RhdGEkc3R1ZHlfaWQgPT0gIkhhbW0gMjAxMCIsNDo2XSA8LSBsaXN0KDcuOCwgNS43LCAxMC43KQ0KZGF0YVtkYXRhJHN0dWR5X2lkID09ICJDaGVuIDIwMTIiLDQ6Nl0gPC0gbGlzdCg1Ljk0LCAzLjk0LCA4Ljk0KQ0KZGF0YVtkYXRhJHN0dWR5X2lkID09ICJTdGVlbmxhbmQgMjAwOSIsNDo2XSA8LSBsaXN0KDIwLjIsIDEzLjYsIDI5LjMpDQoNCiNDYWxjdWxhdGUgbWVhbiBhbmQgU0QgZnJvbSBtZWRpYW4NCmRhdGEkZXhwX21lYW5baXMubmEoZGF0YSRleHBfbWVhbikgJiAhaXMubmEoZGF0YSRleHBfbWVkKV0gPC0gDQogIChkYXRhJGV4cF8xcVtpcy5uYShkYXRhJGV4cF9tZWFuKSAmICFpcy5uYShkYXRhJGV4cF9tZWQpXSArIA0KICAgICBkYXRhJGV4cF9tZWRbaXMubmEoZGF0YSRleHBfbWVhbikgJiAhaXMubmEoZGF0YSRleHBfbWVkKV0gKyANCiAgICAgZGF0YSRleHBfM3FbaXMubmEoZGF0YSRleHBfbWVhbikgJiAhaXMubmEoZGF0YSRleHBfbWVkKV0pIC8gMw0KYGBgDQoNCkZpcnN0IHdlIHJ1biB0aGUgUm9kcmlndWV6LUJhcnJhbmNvIG1ldGhvZCB1c2luZyBtb2RlbCBBIChsaW5lYXItbGluZWFyKSBmb3IgbGluZWFyLWJhc2UgZGF0YSAocmVsYXRpdmUgeCkgYW5kIG1vZGVsIEIgKGxvZy1saW5lYXIpIGZvciBsb2ctYmFzZSBkYXRhIChhYnNvbHV0ZSB4KS4gQWJzb2x1dGUgeSBpcyBhbHdheXMgdXNlZC4gVGhlIGFic29sdXRlIGNoYW5nZSAoYykgaXMgMSB3aGVyZWFzIHRoZSByZWxhdGl2ZSBjaGFuZ2UgKGspIGlzIGVxdWl2YWxlbnQgdG8gdGhlIGxvZyBiYXNlIGJlaW5nIHVzZWQuDQoNCmBgYHtyIHRhYjRyYn0NCnJib3V0IDwtIGRhdGEuZnJhbWUoInN0dWR5IiA9IGRhdGEkc3R1ZHlfaWQsICJpbnB1dF9iYXNlIiA9IGRhdGEkaW5wdXRfYmFzZSwgImFjdHVhbF9iZXRhIiA9IGRhdGEkbWFudWFsX2JldGEsICJhY3R1YWxfYmxjaSIgPSBkYXRhJG1hbnVhbF9ibGNpLCAiYWN0dWFsX2J1Y2kiID0gZGF0YSRtYW51YWxfYnVjaSwgImJldGEiID0gYygxOm5yb3coZGF0YSkpLCAiYmV0YV9sY2kiID0gYygxOm5yb3coZGF0YSkpLCAiYmV0YV91Y2kiID0gYygxOm5yb3coZGF0YSkpLCAibG9nYmFzZSIgPSBkYXRhJGxvZ2Jhc2UsICJub2JzIiA9IGRhdGEkbm9icywgInNpZ21hIiA9IGRhdGEkc2lnbWEsICJtZWRsaXN0IiA9IGRhdGEkbWVkbGlzdCkNCiNIZXJlIHdlJ3JlIGRlZmluaW5nIHRoZSBsb2ctYmFzZSBleHBvc3VyZXMgYXMgYWJzb2x1dGUgZXhwcmVzc2lvbnMgYW5kIHRoZSBsaW5lYXIgYXMgcmVsYXRpdmUgZXhwcmVzc2lvbnMNCnguZXhwIDwtIGlmZWxzZShkYXRhJGlucHV0X2Jhc2UgPT0gImxvZyIsICJhYnMiLCAicmVsIikNCmZvcihpIGluIDE6bnJvdyhkYXRhKSl7DQogIHJib3V0W2ksNjo4XSA8LSByYmV4cChtb2RlbCA9IGRhdGEkcmJfbW9kZWxbaV0sDQogICAgICAgICAgICAgICAgICAgICAgICB5LmV4cCA9ICJhYnMiLA0KICAgICAgICAgICAgICAgICAgICAgICAgeC5leHAgPSB4LmV4cFtpXSwNCiAgICAgICAgICAgICAgICAgICAgICAgIGJldGEgPSBkYXRhJGJldGFfbWVhbltpXSwNCiAgICAgICAgICAgICAgICAgICAgICAgIHNlID0gKGRhdGEkYmV0YV91Y2lbaV0tZGF0YSRiZXRhX2xjaVtpXSkvMy45MiwNCiAgICAgICAgICAgICAgICAgICAgICAgIG1lYW4ueCA9IGRhdGEkZXhwX21lYW5baV0sICNkYXRhJGV4cF9tZWRbaV0sIGNoYW5nZWQgMTIvMTMvMjINCiAgICAgICAgICAgICAgICAgICAgICAgIG1lYW4ueSA9IDAsDQogICAgICAgICAgICAgICAgICAgICAgICBhID0gZXhwKDEpLA0KICAgICAgICAgICAgICAgICAgICAgICAgYiA9IGRhdGEkbG9nYmFzZVtpXSwNCiAgICAgICAgICAgICAgICAgICAgICAgIGsgPSBkYXRhJGxvZ2Jhc2VbaV0sDQogICAgICAgICAgICAgICAgICAgICAgICBjID0gMSkNCn0NCnJib3V0JG1ldGhvZCA8LSAiUm9kcmlndWV6LUJhcnJhbmNvIE1ldGhvZCIgDQpgYGANCg0KTmV4dCB3ZSBydW4gdGhlICJBbHRlcm5hdGl2ZSIgbWV0aG9kLiBUaGlzIG9uZSBpcyByZWxhdGl2ZWx5IHN0cmFpZ2h0Zm9yd2FyZCwgYW5kIHRoZSB2YWx1ZXMgY2FuIGp1c3QgYmUgaW5wdXQgZGlyZWN0bHkgZnJvbSB0aGUgZGF0YSBmcmFtZS4NCg0KYGBge3IgdGFiNGFsdH0NCmFsdG91dCA8LSBkYXRhLmZyYW1lKCJzdHVkeSIgPSBkYXRhJHN0dWR5X2lkLCAiaW5wdXRfYmFzZSIgPSBkYXRhJGlucHV0X2Jhc2UsICJhY3R1YWxfYmV0YSIgPSBkYXRhJG1hbnVhbF9iZXRhLCAiYWN0dWFsX2JsY2kiID0gZGF0YSRtYW51YWxfYmxjaSwgImFjdHVhbF9idWNpIiA9IGRhdGEkbWFudWFsX2J1Y2ksICJiZXRhIiA9IGMoMTpucm93KGRhdGEpKSwgImJldGFfbGNpIiA9IGMoMTpucm93KGRhdGEpKSwgImJldGFfdWNpIiA9IGMoMTpucm93KGRhdGEpKSwgImxvZ2Jhc2UiID0gZGF0YSRsb2diYXNlLCAibm9icyIgPSBkYXRhJG5vYnMsICJzaWdtYSIgPSBkYXRhJHNpZ21hLCAibWVkbGlzdCIgPSBkYXRhJG1lZGxpc3QpDQpmb3IoaSBpbiAxOm5yb3coZGF0YSkpew0KICBhbHRvdXRbaSw2OjhdIDwtIGFsdGV4cChpbnB1dC5iYXNlID0gZGF0YSRpbnB1dF9iYXNlW2ldLA0KICAgICAgICAgICAgICAgICAgICAgICAgICBtZWRpYW4gPSBkYXRhJGV4cF9tZWRbaV0sDQogICAgICAgICAgICAgICAgICAgICAgICAgIGxvZy5iYXNlID0gZGF0YSRsb2diYXNlW2ldLA0KICAgICAgICAgICAgICAgICAgICAgICAgICBiZXRhLmRpc3QgPSBjKGRhdGEkYmV0YV9tZWFuW2ldLCBkYXRhJGJldGFfbGNpW2ldLCBkYXRhJGJldGFfdWNpW2ldKSkNCn0NCmFsdG91dCRtZXRob2QgPC0gIkFsdGVybmF0aXZlIE1ldGhvZCINCmBgYA0KDQpGaW5hbGx5LCB3ZSdsbCByZXBsaWNhdGUgdGhlIGNvZGUgdXNlZCBhYm92ZSBmb3IgVGFibGUgMyB3aXRoIHRoZSBEemllcmxlbmdhIG1ldGhvZCwgYnV0IHRoaXMgdGltZSB1c2luZyB0aGUgZXN0aW1hdGVkIG1lZGlhbnMgZm9yIGNvbnNpc3RlbmN5IGFjcm9zcyBtZXRob2RzLg0KDQpgYGB7ciB0YWI0ZHp9DQpkem91dCA8LSBkYXRhLmZyYW1lKCJzdHVkeSIgPSBkYXRhJHN0dWR5X2lkLCAiaW5wdXRfYmFzZSIgPSBkYXRhJGlucHV0X2Jhc2UsICJhY3R1YWxfYmV0YSIgPSBkYXRhJG1hbnVhbF9iZXRhLCAiYWN0dWFsX2JsY2kiID0gZGF0YSRtYW51YWxfYmxjaSwgImFjdHVhbF9idWNpIiA9IGRhdGEkbWFudWFsX2J1Y2ksICJiZXRhIiA9IGMoMTpucm93KGRhdGEpKSwgImJldGFfbGNpIiA9IGMoMTpucm93KGRhdGEpKSwgImJldGFfdWNpIiA9IGMoMTpucm93KGRhdGEpKSwgImxvZ2Jhc2UiID0gZGF0YSRsb2diYXNlLCAibm9icyIgPSBkYXRhJG5vYnMsICJzaWdtYSIgPSBkYXRhJHNpZ21hLCAibWVkbGlzdCIgPSBkYXRhJG1lZGxpc3QpDQpmb3IoaSBpbiAxOm5yb3coZGF0YSkpew0KICBkem91dFtpLDY6OF0gPC0gZHpleHAoaW5wdXQuYmFzZSA9IGRhdGEkaW5wdXRfYmFzZVtpXSwgDQogICAgICAgICAgICAgICAgICAgICAgICBleHAuZGlzdCA9IGMobWVkID0gZGF0YSRleHBfbWVkW2ldLCBpcXIxID0gZGF0YSRleHBfMXFbaV0sIGlxcjMgPSBkYXRhJGV4cF8zcVtpXSwNCiAgICAgICAgICAgICAgICAgICAgICAgICAgICAgICAgICAgICBnbWVhbiA9IGRhdGEkZXhwX2dtZWFuW2ldLCBnc2QgPSBkYXRhJGV4cF9nc2RbaV0sDQogICAgICAgICAgICAgICAgICAgICAgICAgICAgICAgICAgICAgbWVhbiA9IGRhdGEkZXhwX21lYW5baV0sIHNkID0gZGF0YSRleHBfc2RbaV0pLA0KICAgICAgICAgICAgICAgICAgICAgICAgYmV0YS5kaXN0ID0gYyhkYXRhJGJldGFfbWVhbltpXSwgZGF0YSRiZXRhX2xjaVtpXSwgZGF0YSRiZXRhX3VjaVtpXSksDQogICAgICAgICAgICAgICAgICAgICAgICBsb2cuYmFzZSA9IGRhdGEkbG9nYmFzZVtpXSkNCn0NCmR6b3V0JG1ldGhvZCA8LSAiRHppZXJsZW5nYSBNZXRob2QiDQpgYGANCg0KRmluYWxseSwgdGhlIG91dHB1dHRlZCBkYXRhc2V0cyBmb3IgZWFjaCBtZXRob2QgYWxvbmcgd2l0aCB0aGVpciByZS1leHByZXNzZWQgdmFsdWVzIGFyZSBjb21iaW5lZCBhbmQgdGhlIHRhYmxlIGlzIGZvcm1hdHRlZC4NCg0KYGBge3IgdGFiNGdlbn0NCmRmb3V0IDwtIHJiaW5kKHJib3V0LCBhbHRvdXQsIGR6b3V0KQ0KZGZvdXQkcHJvcF9kaWZmX2JldGEgPC0gKGRmb3V0JGJldGEvZGZvdXQkYWN0dWFsX2JldGEpLTENCg0KdGFiNCA8LSBtZXJnZShyYm91dCwgYWx0b3V0LCBieSA9IGMoInN0dWR5IiwgImlucHV0X2Jhc2UiLCJsb2diYXNlIiwibm9icyIsInNpZ21hIiwibWVkbGlzdCIsICJhY3R1YWxfYmV0YSIsICJhY3R1YWxfYmxjaSIsICJhY3R1YWxfYnVjaSIpKQ0KdGFiNCA8LSBtZXJnZSh0YWI0LCBkem91dCwgYnkgPSBjKCJzdHVkeSIsICJpbnB1dF9iYXNlIiwibG9nYmFzZSIsIm5vYnMiLCJzaWdtYSIsIm1lZGxpc3QiLCAiYWN0dWFsX2JldGEiLCAiYWN0dWFsX2JsY2kiLCAiYWN0dWFsX2J1Y2kiKSkNCnRhYjQkcF9kX2JldGEueCA8LSByb3VuZCgodGFiNCRiZXRhLngvdGFiNCRhY3R1YWxfYmV0YSktMSwyKQ0KdGFiNCRwX2RfYmV0YS55IDwtIHJvdW5kKCh0YWI0JGJldGEueS90YWI0JGFjdHVhbF9iZXRhKS0xLDIpDQp0YWI0JHBfZF9iZXRhIDwtIHJvdW5kKCh0YWI0JGJldGEvdGFiNCRhY3R1YWxfYmV0YSktMSwyKQ0KDQp0YWI0JGFsdF9iZXRhX2xjaSA8LSBhbHRvdXQkYmV0YV9sY2kNCnRhYjQkYWx0X2JldGFfdWNpIDwtIGFsdG91dCRiZXRhX3VjaQ0KDQp0YWI0IDwtIHRhYjQgJT4lIG11dGF0ZShhY3Jvc3MoYyhhY3R1YWxfYmV0YSwgYmV0YS54LCBiZXRhLnksIGJldGEpLCBzaWduaWYsIDQpKQ0KdGFiNCA8LSB0YWI0ICU+JSBtdXRhdGUoYWNyb3NzKGMocF9kX2JldGEueCwgcF9kX2JldGEueSwgcF9kX2JldGEpLCByb3VuZCwgMikpDQp0YWI0IDwtIHRhYjRbb3JkZXIodGFiNCRpbnB1dF9iYXNlLGRlY3JlYXNpbmcgPSBUKSxdDQojdGFiNCA8LSB0YWI0ICU+JSBtdXRhdGUoYWNyb3NzKGV2ZXJ5dGhpbmcoKSwgYXMuY2hhcmFjdGVyKSkNCnRhYjQgPC0gdGFiNFtjKCJzdHVkeSIsImlucHV0X2Jhc2UiLCJsb2diYXNlIiwibm9icyIsInNpZ21hIiwibWVkbGlzdCIsImFjdHVhbF9iZXRhIiwgImFjdHVhbF9ibGNpIiwgImFjdHVhbF9idWNpIiwgImJldGEueCIsInBfZF9iZXRhLngiLCJhbHRfYmV0YV9sY2kiLCJiZXRhLnkiLCJhbHRfYmV0YV91Y2kiLCJwX2RfYmV0YS55IiwiYmV0YSIsInBfZF9iZXRhIildDQpuYW1lcyh0YWI0KSA8LSBjKCJTdHVkeSIsICJJbnB1dF9CYXNlIiwibG9nYmFzZSIsIm5vYnMiLCJzaWdtYSIsIm1lZGxpc3QiLCJPYnNlcnZlZF9CZXRhIiwgIkJldGFfTG93ZXIiLCAiQmV0YV9VcHBlciIsIlJCX0JldGEiLCJkZWx0YV9SQl9CZXRhIiwiYWx0X2JldGFfbGNpIiwiQWx0X0JldGEiLCJhbHRfYmV0YV91Y2kiLCJkZWx0YV9BbHRfQmV0YSIsIkR6X0JldGEiLCJkZWx0YV9Eel9CZXRhIikNCg0KdGFiNCA8LSBhcy5kYXRhLnRhYmxlKHRhYjQpDQp0YWI0IDwtIHRhYjRbdGFiNCRJbnB1dF9CYXNlID09ICJsb2ciLF0NCnRhYjQkbG9nYmFzZVt0YWI0JGxvZ2Jhc2UgPT0gMi43MTgyODE4MjhdIDwtICJlIg0KdGFiNCRzaWdtYSA8LSBhcy5udW1lcmljKHRhYjQkc2lnbWEpDQp0YWI0JG5vYnMgPC0gYXMubnVtZXJpYyh0YWI0JG5vYnMpDQp0YWI0JG1lZGxpc3QgPC0gYXMubnVtZXJpYyh0YWI0JG1lZGxpc3QpDQp0YWI1IDwtIHRhYjQgI1NldCB1cCBuZXh0IHRhYmxlDQp0YWI0IDwtIGRwbHlyOjpzZWxlY3QodGFiNCxTdHVkeSxPYnNlcnZlZF9CZXRhLFJCX0JldGEsZGVsdGFfUkJfQmV0YSxEel9CZXRhLGRlbHRhX0R6X0JldGEsQWx0X0JldGEsZGVsdGFfQWx0X0JldGEpDQp0YWI0IDwtIHRhYjQgJT4lIG11dGF0ZV9hdChjKCJPYnNlcnZlZF9CZXRhIiwiUkJfQmV0YSIsImRlbHRhX1JCX0JldGEiLCJEel9CZXRhIiwiZGVsdGFfRHpfQmV0YSIsIkFsdF9CZXRhIiwiZGVsdGFfQWx0X0JldGEiKSxzaWduaWYsIGRpZ2l0cyA9IDMpDQp0YWI0IDwtIHRhYjQgJT4lIG11dGF0ZV9pZihpcy5udW1lcmljLCBhcy5jaGFyYWN0ZXIpDQp0YWI0DQpgYGANCg0KIyBUYWJsZSA0Qg0KDQpUaGlzIGlzIGdvaW5nIHRvIGJlIG9uZSBsb25nIGNvZGUgY2h1bmsgdG8gZ2VuZXJhdGUgVGFibGUgM0Igc2luY2UgaXQgd2lsbCBqdXN0IGJlIHJlcGxpY2F0aW5nIHRoZSBhbmFseXNpcyBmb3IgVGFibGUgM0EgYnV0IHdpdGggZGlmZmVyZW50IGlucHV0IGRhdGENCg0KYGBge3IgdGFiM2JnZW59DQpyYm91dCA8LSBkYXRhLmZyYW1lKCJzdHVkeSIgPSBkYXRhYiRzdHVkeV9pZCwgImlucHV0X2Jhc2UiID0gZGF0YWIkaW5wdXRfYmFzZSwgImFjdHVhbF9iZXRhIiA9IGRhdGFiJG1hbnVhbF9iZXRhLCAiYWN0dWFsX2JsY2kiID0gZGF0YWIkbWFudWFsX2JsY2ksICJhY3R1YWxfYnVjaSIgPSBkYXRhYiRtYW51YWxfYnVjaSwgImJldGEiID0gYygxOm5yb3coZGF0YWIpKSwgImJldGFfbGNpIiA9IGMoMTpucm93KGRhdGFiKSksICJiZXRhX3VjaSIgPSBjKDE6bnJvdyhkYXRhYikpLCAibG9nYmFzZSIgPSBkYXRhYiRsb2diYXNlLCAibm9icyIgPSBkYXRhYiRub2JzLCAic2lnbWEiID0gZGF0YWIkc2lnbWEsICJtZWRsaXN0IiA9IGRhdGFiJG1lZGxpc3QpDQojSGVyZSB3ZSdyZSBkZWZpbmluZyB0aGUgbG9nLWJhc2UgZXhwb3N1cmVzIGFzIGFic29sdXRlIGV4cHJlc3Npb25zIGFuZCB0aGUgbGluZWFyIGFzIHJlbGF0aXZlIGV4cHJlc3Npb25zDQp4LmV4cCA8LSBpZmVsc2UoZGF0YWIkaW5wdXRfYmFzZSA9PSAibG9nIiwgImFicyIsICJyZWwiKQ0KZm9yKGkgaW4gMTpucm93KGRhdGFiKSl7DQogIHJib3V0W2ksNjo4XSA8LSByYmV4cChtb2RlbCA9IGRhdGFiJHJiX21vZGVsW2ldLA0KICAgICAgICAgICAgICAgICAgICAgICAgeS5leHAgPSAiYWJzIiwNCiAgICAgICAgICAgICAgICAgICAgICAgIHguZXhwID0geC5leHBbaV0sDQogICAgICAgICAgICAgICAgICAgICAgICBiZXRhID0gZGF0YWIkYmV0YV9tZWFuW2ldLA0KICAgICAgICAgICAgICAgICAgICAgICAgc2UgPSAoZGF0YWIkYmV0YV91Y2lbaV0tZGF0YWIkYmV0YV9sY2lbaV0pLzMuOTIsDQogICAgICAgICAgICAgICAgICAgICAgICBtZWFuLnggPSBkYXRhYiRleHBfbWVhbltpXSwgI2RhdGFiJGV4cF9tZWRbaV0sIGNoYW5nZWQgMTIvMTMvMjINCiAgICAgICAgICAgICAgICAgICAgICAgIG1lYW4ueSA9IDAsDQogICAgICAgICAgICAgICAgICAgICAgICBhID0gZXhwKDEpLA0KICAgICAgICAgICAgICAgICAgICAgICAgYiA9IGRhdGFiJGxvZ2Jhc2VbaV0sDQogICAgICAgICAgICAgICAgICAgICAgICBrID0gZGF0YWIkbG9nYmFzZVtpXSwNCiAgICAgICAgICAgICAgICAgICAgICAgIGMgPSAxKQ0KfQ0KcmJvdXQkbWV0aG9kIDwtICJSb2RyaWd1ZXotQmFycmFuY28gTWV0aG9kIiANCg0KYWx0b3V0IDwtIGRhdGEuZnJhbWUoInN0dWR5IiA9IGRhdGFiJHN0dWR5X2lkLCAiaW5wdXRfYmFzZSIgPSBkYXRhYiRpbnB1dF9iYXNlLCAiYWN0dWFsX2JldGEiID0gZGF0YWIkbWFudWFsX2JldGEsICJhY3R1YWxfYmxjaSIgPSBkYXRhYiRtYW51YWxfYmxjaSwgImFjdHVhbF9idWNpIiA9IGRhdGFiJG1hbnVhbF9idWNpLCAiYmV0YSIgPSBjKDE6bnJvdyhkYXRhYikpLCAiYmV0YV9sY2kiID0gYygxOm5yb3coZGF0YWIpKSwgImJldGFfdWNpIiA9IGMoMTpucm93KGRhdGFiKSksICJsb2diYXNlIiA9IGRhdGFiJGxvZ2Jhc2UsICJub2JzIiA9IGRhdGFiJG5vYnMsICJzaWdtYSIgPSBkYXRhYiRzaWdtYSwgIm1lZGxpc3QiID0gZGF0YWIkbWVkbGlzdCkNCmZvcihpIGluIDE6bnJvdyhkYXRhYikpew0KICBhbHRvdXRbaSw2OjhdIDwtIGFsdGV4cChpbnB1dC5iYXNlID0gZGF0YWIkaW5wdXRfYmFzZVtpXSwNCiAgICAgICAgICAgICAgICAgICAgICAgICAgbWVkaWFuID0gZGF0YWIkZXhwX21lZFtpXSwNCiAgICAgICAgICAgICAgICAgICAgICAgICAgbG9nLmJhc2UgPSBkYXRhYiRsb2diYXNlW2ldLA0KICAgICAgICAgICAgICAgICAgICAgICAgICBiZXRhLmRpc3QgPSBjKGRhdGFiJGJldGFfbWVhbltpXSwgZGF0YWIkYmV0YV9sY2lbaV0sIGRhdGFiJGJldGFfdWNpW2ldKSkNCn0NCmFsdG91dCRtZXRob2QgPC0gIkFsdGVybmF0aXZlIE1ldGhvZCINCg0KZHpvdXQgPC0gZGF0YS5mcmFtZSgic3R1ZHkiID0gZGF0YWIkc3R1ZHlfaWQsICJpbnB1dF9iYXNlIiA9IGRhdGFiJGlucHV0X2Jhc2UsICJhY3R1YWxfYmV0YSIgPSBkYXRhYiRtYW51YWxfYmV0YSwgImFjdHVhbF9ibGNpIiA9IGRhdGFiJG1hbnVhbF9ibGNpLCAiYWN0dWFsX2J1Y2kiID0gZGF0YWIkbWFudWFsX2J1Y2ksICJiZXRhIiA9IGMoMTpucm93KGRhdGFiKSksICJiZXRhX2xjaSIgPSBjKDE6bnJvdyhkYXRhYikpLCAiYmV0YV91Y2kiID0gYygxOm5yb3coZGF0YWIpKSwgImxvZ2Jhc2UiID0gZGF0YWIkbG9nYmFzZSwgIm5vYnMiID0gZGF0YWIkbm9icywgInNpZ21hIiA9IGRhdGFiJHNpZ21hLCAibWVkbGlzdCIgPSBkYXRhYiRtZWRsaXN0KQ0KZm9yKGkgaW4gMTpucm93KGRhdGFiKSl7DQogIGR6b3V0W2ksNjo4XSA8LSBkemV4cChpbnB1dC5iYXNlID0gZGF0YWIkaW5wdXRfYmFzZVtpXSwgDQogICAgICAgICAgICAgICAgICAgICAgICBleHAuZGlzdCA9IGMobWVkID0gZGF0YWIkZXhwX21lZFtpXSwgaXFyMSA9IGRhdGFiJGV4cF8xcVtpXSwgaXFyMyA9IGRhdGFiJGV4cF8zcVtpXSwNCiAgICAgICAgICAgICAgICAgICAgICAgICAgICAgICAgICAgICBnbWVhbiA9IGRhdGFiJGV4cF9nbWVhbltpXSwgZ3NkID0gZGF0YWIkZXhwX2dzZFtpXSwNCiAgICAgICAgICAgICAgICAgICAgICAgICAgICAgICAgICAgICBtZWFuID0gZGF0YWIkZXhwX21lYW5baV0sIHNkID0gZGF0YWIkZXhwX3NkW2ldKSwNCiAgICAgICAgICAgICAgICAgICAgICAgIGJldGEuZGlzdCA9IGMoZGF0YWIkYmV0YV9tZWFuW2ldLCBkYXRhYiRiZXRhX2xjaVtpXSwgZGF0YWIkYmV0YV91Y2lbaV0pLA0KICAgICAgICAgICAgICAgICAgICAgICAgbG9nLmJhc2UgPSBkYXRhYiRsb2diYXNlW2ldKQ0KfQ0KZHpvdXQkbWV0aG9kIDwtICJEemllcmxlbmdhIE1ldGhvZCINCg0KZGZvdXQgPC0gcmJpbmQocmJvdXQsIGFsdG91dCwgZHpvdXQpDQpkZm91dCRwcm9wX2RpZmZfYmV0YSA8LSAoZGZvdXQkYmV0YS9kZm91dCRhY3R1YWxfYmV0YSktMQ0KDQp0YWIzYiA8LSBtZXJnZShyYm91dCwgYWx0b3V0LCBieSA9IGMoInN0dWR5IiwgImlucHV0X2Jhc2UiLCJsb2diYXNlIiwibm9icyIsInNpZ21hIiwibWVkbGlzdCIsICJhY3R1YWxfYmV0YSIsICJhY3R1YWxfYmxjaSIsICJhY3R1YWxfYnVjaSIpKQ0KdGFiM2IgPC0gbWVyZ2UodGFiM2IsIGR6b3V0LCBieSA9IGMoInN0dWR5IiwgImlucHV0X2Jhc2UiLCJsb2diYXNlIiwibm9icyIsInNpZ21hIiwibWVkbGlzdCIsICJhY3R1YWxfYmV0YSIsICJhY3R1YWxfYmxjaSIsICJhY3R1YWxfYnVjaSIpKQ0KdGFiM2IkcF9kX2JldGEueCA8LSByb3VuZCgodGFiM2IkYmV0YS54L3RhYjNiJGFjdHVhbF9iZXRhKS0xLDIpDQp0YWIzYiRwX2RfYmV0YS55IDwtIHJvdW5kKCh0YWIzYiRiZXRhLnkvdGFiM2IkYWN0dWFsX2JldGEpLTEsMikNCnRhYjNiJHBfZF9iZXRhIDwtIHJvdW5kKCh0YWIzYiRiZXRhL3RhYjNiJGFjdHVhbF9iZXRhKS0xLDIpDQoNCnRhYjNiJGFsdF9iZXRhX2xjaSA8LSBhbHRvdXQkYmV0YV9sY2kNCnRhYjNiJGFsdF9iZXRhX3VjaSA8LSBhbHRvdXQkYmV0YV91Y2kNCg0KdGFiM2IgPC0gdGFiM2IgJT4lIG11dGF0ZShhY3Jvc3MoYyhhY3R1YWxfYmV0YSwgYmV0YS54LCBiZXRhLnksIGJldGEpLCBzaWduaWYsIDQpKQ0KdGFiM2IgPC0gdGFiM2IgJT4lIG11dGF0ZShhY3Jvc3MoYyhwX2RfYmV0YS54LCBwX2RfYmV0YS55LCBwX2RfYmV0YSksIHJvdW5kLCAyKSkNCnRhYjNiIDwtIHRhYjNiW29yZGVyKHRhYjNiJGlucHV0X2Jhc2UsZGVjcmVhc2luZyA9IFQpLF0NCiN0YWIzYiA8LSB0YWIzYiAlPiUgbXV0YXRlKGFjcm9zcyhldmVyeXRoaW5nKCksIGFzLmNoYXJhY3RlcikpDQp0YWIzYiA8LSB0YWIzYltjKCJzdHVkeSIsImlucHV0X2Jhc2UiLCJsb2diYXNlIiwibm9icyIsInNpZ21hIiwibWVkbGlzdCIsImFjdHVhbF9iZXRhIiwgImFjdHVhbF9ibGNpIiwgImFjdHVhbF9idWNpIiwgImJldGEueCIsInBfZF9iZXRhLngiLCJhbHRfYmV0YV9sY2kiLCJiZXRhLnkiLCJhbHRfYmV0YV91Y2kiLCJwX2RfYmV0YS55IiwiYmV0YSIsInBfZF9iZXRhIildDQpuYW1lcyh0YWIzYikgPC0gYygiU3R1ZHkiLCAiSW5wdXRfQmFzZSIsImxvZ2Jhc2UiLCJub2JzIiwic2lnbWEiLCJtZWRsaXN0IiwiT2JzZXJ2ZWRfQmV0YSIsICJCZXRhX0xvd2VyIiwgIkJldGFfVXBwZXIiLCJSQl9CZXRhIiwiZGVsdGFfUkJfQmV0YSIsImFsdF9iZXRhX2xjaSIsIkFsdF9CZXRhIiwiYWx0X2JldGFfdWNpIiwiZGVsdGFfQWx0X0JldGEiLCJEel9CZXRhIiwiZGVsdGFfRHpfQmV0YSIpDQoNCnRhYjNiIDwtIGFzLmRhdGEudGFibGUodGFiM2IpDQp0YWIzYiA8LSB0YWIzYlt0YWIzYiRJbnB1dF9CYXNlID09ICJsb2ciLF0NCnRhYjNiJGxvZ2Jhc2VbdGFiM2IkbG9nYmFzZSA9PSAyLjcxODI4MTgyOF0gPC0gImUiDQp0YWIzYiRzaWdtYSA8LSBhcy5udW1lcmljKHRhYjNiJHNpZ21hKQ0KdGFiM2Ikbm9icyA8LSBhcy5udW1lcmljKHRhYjNiJG5vYnMpDQp0YWIzYiRtZWRsaXN0IDwtIGFzLm51bWVyaWModGFiM2IkbWVkbGlzdCkNCnRhYjQgPC0gdGFiM2IgI1NldCB1cCBuZXh0IHRhYmxlDQp0YWIzYiA8LSBkcGx5cjo6c2VsZWN0KHRhYjNiLFN0dWR5LE9ic2VydmVkX0JldGEsUkJfQmV0YSxkZWx0YV9SQl9CZXRhLER6X0JldGEsZGVsdGFfRHpfQmV0YSxBbHRfQmV0YSxkZWx0YV9BbHRfQmV0YSkNCnRhYjNiIDwtIHRhYjNiICU+JSBtdXRhdGVfYXQoYygiT2JzZXJ2ZWRfQmV0YSIsIlJCX0JldGEiLCJkZWx0YV9SQl9CZXRhIiwiRHpfQmV0YSIsImRlbHRhX0R6X0JldGEiLCJBbHRfQmV0YSIsImRlbHRhX0FsdF9CZXRhIiksc2lnbmlmLCBkaWdpdHMgPSAzKQ0KdGFiM2IgPC0gdGFiM2IgJT4lIG11dGF0ZV9pZihpcy5udW1lcmljLCBhcy5jaGFyYWN0ZXIpDQpzdW1tYXJ5KGFzLm51bWVyaWModGFiM2IkZGVsdGFfUkJfQmV0YSkpDQpzdW1tYXJ5KGFzLm51bWVyaWModGFiM2IkZGVsdGFfRHpfQmV0YSkpDQpzdW1tYXJ5KGFzLm51bWVyaWModGFiM2IkZGVsdGFfQWx0X0JldGEpKQ0KdGFiM2INCmBgYA0KDQoNCiMgVGFibGUgNQ0KDQpXZSB3aWxsIHRoZW4gZ28gdGhyb3VnaCB0aGUgc2FtZSBwcm9jZXNzLCBidXQgYWRqdXN0aW5nIGZvciB0aGUgT0xTIHJlZ3Jlc3Npb24gd2l0aCBzaWdtYSBwZXJmb3JtZWQgaW4gdGhlIHNpbXVsYXRpb24uIFRoaXMgd2lsbCBjb21wcmlzZSBUYWJsZSA1Lg0KYGBge3IgdGFiNWdlbn0NCg0KdGFiNSRSQl9CZXRhX2FkaiA8LSBhcy5udW1lcmljKHRhYjUkUkJfQmV0YSkvKDErcHJlZGljdChzdGVwLjJwNix0YWI1KSkNCnRhYjUkQWx0X0JldGFfYWRqbCA8LSBhcy5udW1lcmljKHRhYjUkYWx0X2JldGFfbGNpKS8oMStwcmVkaWN0KGZpdDJwNWwsdGFiNSkpDQp0YWI1JEFsdF9CZXRhX2FkaiA8LSBhcy5udW1lcmljKHRhYjUkQWx0X0JldGEpLygxK3ByZWRpY3Qoc3RlcC4ycDUsdGFiNSkpDQp0YWI1JEFsdF9CZXRhX2FkanUgPC0gYXMubnVtZXJpYyh0YWI1JGFsdF9iZXRhX3VjaSkvKDErcHJlZGljdChmaXQycDV1LHRhYjUpKQ0KdGFiNSREel9CZXRhX2FkamwgPC0gYXMubnVtZXJpYyh0YWI1JGR6X2JldGFfbGNpKS8oMStwcmVkaWN0KGZpdDJwN2wsdGFiNSkpDQpjb2xuYW1lcyh0YWI1KVtjb2xuYW1lcyh0YWI1KT09Ik9ic2VydmVkX0JldGEiXSA8LSAiYmV0YSINCnRhYjUkRHpfQmV0YV9hZGogPC0gYXMubnVtZXJpYyh0YWI1JER6X0JldGEpLygxK3ByZWRpY3Qoc3RlcC4ycDcsdGFiNSkpDQpjb2xuYW1lcyh0YWI1KVtjb2xuYW1lcyh0YWI1KT09ImJldGEiXSA8LSAiT2JzZXJ2ZWRfQmV0YSINCnRhYjUkRHpfQmV0YV9hZGp1IDwtIGFzLm51bWVyaWModGFiNSRkel9iZXRhX3VjaSkvKDErcHJlZGljdChmaXQycDd1LHRhYjUpKQ0KdGFiNSRBbHRfQmV0YV9hZGpsW3RhYjUkQWx0X0JldGFfYWRqbCA+IHRhYjUkQWx0X0JldGFfYWRqXSA8LSB0YWI1JEFsdF9CZXRhX2Fkalt0YWI1JEFsdF9CZXRhX2FkamwgPiB0YWI1JEFsdF9CZXRhX2Fkal0NCnRhYjUkQWx0X0JldGFfYWRqdVt0YWI1JEFsdF9CZXRhX2FkanUgPCB0YWI1JEFsdF9CZXRhX2Fkal0gPC0gdGFiNSRBbHRfQmV0YV9hZGpbdGFiNSRBbHRfQmV0YV9hZGp1IDwgdGFiNSRBbHRfQmV0YV9hZGpdDQp0YWI1JGRlbHRhX1JCX0JldGFfYWRqIDwtIHJvdW5kKCh0YWI1JFJCX0JldGFfYWRqL2FzLm51bWVyaWModGFiNSRPYnNlcnZlZF9CZXRhKSktMSwyKQ0KdGFiNSRkZWx0YV9BbHRfQmV0YV9hZGogPC0gcm91bmQoKHRhYjUkQWx0X0JldGFfYWRqL2FzLm51bWVyaWModGFiNSRPYnNlcnZlZF9CZXRhKSktMSwyKQ0KdGFiNSRkZWx0YV9Eel9CZXRhX2FkaiA8LSByb3VuZCgodGFiNSREel9CZXRhX2Fkai9hcy5udW1lcmljKHRhYjUkT2JzZXJ2ZWRfQmV0YSkpLTEsMikNCg0KdGFiNSRTdHVkeWJJbnB1dCA8LSBwYXN0ZSh0YWI1JFN0dWR5LHRhYjUkSW5wdXRfQmFzZSwgImJhc2UiKQ0KdGFiNVssbGFibG9jIDo9IHBtYXgoQWx0X0JldGFfYWRqdSxCZXRhX1VwcGVyKV0NCnRhYjUgPC0gZHBseXI6OnNlbGVjdCh0YWI1LFN0dWR5LE9ic2VydmVkX0JldGEsUkJfQmV0YV9hZGosZGVsdGFfUkJfQmV0YV9hZGosRHpfQmV0YV9hZGosZGVsdGFfRHpfQmV0YV9hZGosQWx0X0JldGFfYWRqLGRlbHRhX0FsdF9CZXRhX2FkaikNCnRhYjUNCg0KYGBgDQoNCiMgU3VwcGxlbWVudGFsIEZpZ3VyZXMgYW5kIFRhYmxlcw0KDQojIyBGaWd1cmUgUzENCg0KU2ltaWxhciB0byBGaWd1cmUgMiwgYnV0IGluY2x1ZGVzIGJldGFER00gPCAwLg0KDQpgYGB7ciBmaWdzMWdlbn0NCg0KbHJleGJldGF0YWIkcG9zYmV0YSA8LSBpZmVsc2UobHJleGJldGF0YWIkYmV0YSA+MCwiUG9zaXRpdmUiLCJOZWdhdGl2ZSIpDQoNCmZpZ3MxYyA8LSBnZ3Bsb3QoZGF0YSA9IGxyZXhiZXRhdGFiLCBhZXMoeCA9IHNpZ21hLCB5ID0gUmVsQmlhc19hbHQpKSsNCiAgZ2VvbV9qaXR0ZXIoYWVzKHNoYXBlID0gYXMuZmFjdG9yKGxvZ2Jhc2UpLCBzaXplID0gbWVkbGlzdA0KICAgICAgICAgICAgICAgICAgLCBmaWxsID0gYXMuY2hhcmFjdGVyKG5vYnMpDQogICAgICAgICAgICAgICAgICApDQogICAgICAgICAgICAgICwgY29sb3IgPSAiYmxhY2siDQogICAgICAgICAgICAgICMgLCBhbHBoYSA9IDAuNg0KICAgICAgICAgICAgICApKw0KICBnZW9tX2xpbmUoZGF0YSA9IGxyZXhiZXRhdGFiW3JlbCA9PSAibG9nIl0sIA0KICAgICAgICAgICAgc3RhdCA9ICdzbW9vdGgnLA0KICAgICAgICAgICAgbWV0aG9kID0gJ2xtJywNCiAgICAgICAgICAgICAgZm9ybXVsYSA9IHl+cG9seSh4LDIscmF3PVQpLA0KICAgICAgICAgICAgICBhZXMobGluZXR5cGUgPSBsb2diYXNlLA0KICAgICAgICAgICAgICAgICAgc2hhcGUgPSBsb2diYXNlLA0KICAgICAgICAgICAgICAgICAgYWxwaGEgPSBwb3NiZXRhLA0KICAgICAgICAgICAgICAgICAgY29sb3IgPSBhcy5jaGFyYWN0ZXIobm9icykpLA0KICAgICAgICAgICAgICBzaXplID0gMS4xKSArDQogIHNjYWxlX3hfY29udGludW91cyhuYW1lID0gIlNpZ21hIixicmVha3MgPSBjKDAuMjUsMC40NSwwLjY1LDAuODUpKSsNCiAgc2NhbGVfeV9jb250aW51b3VzKG5hbWUgPSAiUmVsYXRpdmUgQmlhcyBWYWx1ZSAoQWx0ZXJuYXRpdmUgTWV0aG9kKSIsIGxpbSA9IGMoLTEuMjUsMS4yNSkpKw0KICBzY2FsZV9jb2xvcl9tYW51YWwobmFtZSA9ICJOdW1iZXIgb2YgT2JzZXJ2YXRpb25zIiwNCiAgICAgICAgICAgICAgICAgICAgIHZhbHVlcyA9IGMoIiNENTVFMDAiLCIjNTZCNEU5IiksDQogICAgICAgICAgICAgICAgICAgICBndWlkZSA9ICJub25lIg0KICAgICAgICAgICAgICAgICAgICAgKSsNCiAgc2NhbGVfZmlsbF9tYW51YWwobmFtZSA9ICJOdW1iZXIgb2YgT2JzZXJ2YXRpb25zXG4oKyBGaXR0ZWQgQ3VydmVzKSIsDQogICAgICAgICAgICAgICAgICAgIHZhbHVlcyA9IGFscGhhKGMoIiNENTVFMDAiLCIjNTZCNEU5IiksYygxLDEpKSkrDQogIHNjYWxlX3NoYXBlX21hbnVhbChuYW1lID0gIkxvZ2FyaXRobSBCYXNlIiwNCiAgICAgICAgICAgICAgICAgICAgICAgYnJlYWtzID0gYygyLCJlIiwxMCksDQogICAgICAgICAgICAgICAgICAgICAgIGxhYmVscyA9IGMoIjIiLCJlIiwiMTAiKSwNCiAgICAgICAgICAgICAgICAgICAgICAgIyB2YWx1ZXMgPSBjKDE1LDE4LDIwKQ0KICAgICAgICAgICAgICAgICAgICAgICB2YWx1ZXMgPSBjKDIxLDIyLDI0KQ0KICAgICAgICAgICAgICAgICAgICAgKSsNCiAgc2NhbGVfYWxwaGFfbWFudWFsKG5hbWUgPSAiRml0dGVkIEN1cnZlcyBmb3IgU2lnbiBvZiBCZXRhIiwNCiAgICAgICAgICAgICAgICAgICAgIHZhbHVlcyA9IGMoMC41LDEpKSsNCiAgc2NhbGVfbGluZXR5cGVfbWFudWFsKG5hbWUgPSAiRml0dGVkIEN1cnZlcyBmb3IgTG9nYXJpdGhtIEJhc2UiLA0KICAgICAgICAgICAgICAgICAgICAgICBicmVha3MgPSBjKDEwLCJlIiwyKSwNCiAgICAgICAgICAgICAgICAgICAgICAgbGFiZWxzID0gYygiMTAiLCJlIiwiMiIpLA0KICAgICAgICAgICAgICAgICAgICAgICB2YWx1ZXMgPSBjKDMsMiwxKSkrDQogIHNjYWxlX3NpemUocmFuZ2UgPSBjKDEsNCksbmFtZSA9ICJNZWRpYW4gVmFsdWUiLA0KICAgICAgICAgICAgIGd1aWRlID0gZ3VpZGVfbGVnZW5kKHJldmVyc2UgPSBUUlVFKSkgKw0KICBnZW9tX3ZsaW5lKHhpbnRlcmNlcHQgPSBzaWdtYSkrDQogIHRoZW1lX2J3KCkrDQogIGd1aWRlcyhzaXplID0gZ3VpZGVfbGVnZW5kKG9yZGVyID0gMSwgcmV2ZXJzZSA9IFRSVUUpLA0KICAgICAgICAgc2hhcGUgPSBndWlkZV9sZWdlbmQob3JkZXIgPSAyLA0KICAgICAgICAgICAgICAgICAgICAgICAgICAgICAgb3ZlcnJpZGUuYWVzID0gbGlzdChzaXplID0gMykpLA0KICAgICAgICAgIyBjb2xvciA9IGd1aWRlX2xlZ2VuZChvcmRlciA9IDQsIHJldmVyc2UgPSBUUlVFLA0KICAgICAgICAgIyAgICAgICAgICAgICAgICAgICAgICBvdmVycmlkZS5hZXMgPSBsaXN0KGNvbG9yID0gYygiI0Q1NUUwMCIsIiM1NkI0RTkiKSwNCiAgICAgICAgICMgICAgICAgICAgICAgICAgICAgICAgICAgICAgICAgICAgICAgICAgICBzaXplID0gMykpLA0KICAgICAgICAgZmlsbCA9IGd1aWRlX2xlZ2VuZChvcmRlciA9IDUsDQogICAgICAgICAgICAgICAgICAgICAgICAgICAgIHJldmVyc2UgPSBUUlVFLA0KICAgICAgICAgICAgICAgICAgICAgICAgICAgICBvdmVycmlkZS5hZXMgPSBsaXN0KGNvbG9yID0gYygiIzU2QjRFOSIsIiNENTVFMDAiKSwNCiAgICAgICAgICAgICAgICAgICAgICAgICAgICAgICAgICAgICAgICAgICAgICAgICBhbHBoYSA9IDEsDQogICAgICAgICAgICAgICAgICAgICAgICAgICAgICAgICAgICAgICAgICAgICAgICAgc2l6ZSA9IDYsDQogICAgICAgICAgICAgICAgICAgICAgICAgICAgICAgICAgICAgICAgICAgICAgICAgc2hhcGUgPSAxNSkpLA0KICAgICAgICAgbGluZXR5cGUgPSBndWlkZV9sZWdlbmQob3JkZXIgPSAzLCByZXZlcnNlID0gVFJVRSwgDQogICAgICAgICAgICAgICAgICAgICAgICAgICAgICAgICBvdmVycmlkZS5hZXMgPSBsaXN0KGNvbG9yID0gImJsYWNrIikNCiAgICAgICAgICAgICAgICAgICAgICAgICAgICAgICAgICksDQogICAgICAgICBhbHBoYSA9IGd1aWRlX2xlZ2VuZChvcmRlciA9IDQpKSsNCiAgdGhlbWUoYXhpcy50aXRsZSA9IGVsZW1lbnRfdGV4dChzaXplID0gMTQsIGZhY2UgPSAiYm9sZCIpLA0KICAgICAgICBheGlzLnRleHQgPSBlbGVtZW50X3RleHQoc2l6ZSA9IDEyLCBmYWNlID0gImJvbGQiKSwNCiAgICAgICAgbGVnZW5kLnRpdGxlID0gZWxlbWVudF90ZXh0KHNpemUgPSAxMiwgZmFjZSA9ICJib2xkIiksDQogICAgICAgIGxlZ2VuZC50ZXh0ID0gZWxlbWVudF90ZXh0KHNpemUgPSAxMiwgZmFjZSA9ICJib2xkIiksDQogICAgICAgIGxlZ2VuZC5rZXkud2lkdGggPSB1bml0KDMsImxpbmUiKSkNCg0KDQpmaWdzMWEgPC0gZ2dwbG90KGRhdGEgPSBscmV4YmV0YXRhYiwgYWVzKHggPSBzaWdtYSwgeSA9IFJlbEJpYXNfcmIpKSsNCiAgZ2VvbV9qaXR0ZXIoYWVzKHNoYXBlID0gYXMuZmFjdG9yKGxvZ2Jhc2UpLCBzaXplID0gbWVkbGlzdCwgZmlsbCA9IGFzLmNoYXJhY3Rlcihub2JzKSksDQogICAgICAgICAgICAgIGNvbG9yID0gImJsYWNrIg0KICAgICAgICAgICAgICAjICwgYWxwaGEgPSAwLjUNCiAgICAgICAgICAgICAgKSsNCiBnZW9tX2xpbmUoZGF0YSA9IGxyZXhiZXRhdGFiW3JlbCA9PSAibG9nIl0sIA0KICAgICAgICAgICAgc3RhdCA9ICdzbW9vdGgnLA0KICAgICAgICAgICAgbWV0aG9kID0gJ2xtJywNCiAgICAgICAgICAgICAgZm9ybXVsYSA9IHl+cG9seSh4LDIscmF3PVQpLA0KICAgICAgICAgICAgICBhZXMobGluZXR5cGUgPSBhcy5mYWN0b3IobWVkbGlzdCksDQogICAgICAgICAgICAgICAgICBhbHBoYSA9IHBvc2JldGEpLA0KICAgICAgICAgICAgICBjb2xvciA9ICJibHVlIiwNCiAgICAgICAgICAgc2l6ZSA9IDEuMSkgKw0KICBzY2FsZV94X2NvbnRpbnVvdXMobmFtZSA9ICJTaWdtYSIsYnJlYWtzID0gYygwLjI1LDAuNDUsMC42NSwwLjg1KSkrDQogIHNjYWxlX3lfY29udGludW91cyhuYW1lID0gIlJlbGF0aXZlIEJpYXMgVmFsdWUgKFJvZHJpZ3Vlei1CYXJyYW5jbyBNZXRob2QpIiwgbGltID0gYygtMS4yNSwxLjI1KSkrDQogIHNjYWxlX3NoYXBlX21hbnVhbChuYW1lID0gIkxvZ2FyaXRobSBCYXNlIiwNCiAgICAgICAgICAgICAgICAgICAgICAgYnJlYWtzID0gYygyLCJlIiwxMCksDQogICAgICAgICAgICAgICAgICAgICAgIGxhYmVscyA9IGMoIjIiLCJlIiwiMTAiKSwNCiAgICAgICAgICAgICAgICAgICAgICAgIyB2YWx1ZXMgPSBjKDE1LDE4LDIwKQ0KICAgICAgICAgICAgICAgICAgICAgICB2YWx1ZXMgPSBjKDIxLDIyLDI0KQ0KICAgICAgICAgICAgICAgICAgICAgKSsNCiAgc2NhbGVfZmlsbF9tYW51YWwobmFtZSA9ICJOdW1iZXIgb2YgT2JzZXJ2YXRpb25zIiwNCiAgICAgICAgICAgICAgICAgICAgdmFsdWVzID0gYygiI0Q1NUUwMCIsIiM1NkI0RTkiKSkrDQogIHNjYWxlX2xpbmV0eXBlX2Rpc2NyZXRlKG5hbWUgPSAiRml0dGVkIEN1cnZlcyBmb3IgTWVkaWFuIFZhbHVlcyIpKw0KICAgIHNjYWxlX2FscGhhX21hbnVhbChuYW1lID0gIkZpdHRlZCBDdXJ2ZXMgZm9yIFNpZ24gb2YgQmV0YSIsDQogICAgICAgICAgICAgICAgICAgICB2YWx1ZXMgPSBjKDAuNSwxKSkrDQogIHNjYWxlX3NpemUocmFuZ2UgPSBjKDEsNCksDQogICAgICAgICAgICAgIyBicmVha3MgPSBjKDAuMjUsMC41LDEsMiw0LDgsMTYsMzIpLA0KICAgICAgICAgICAgIG5hbWUgPSAiTWVkaWFuIFZhbHVlIiwNCiAgICAgICAgICAgICBndWlkZSA9IGd1aWRlX2xlZ2VuZChyZXZlcnNlID0gVFJVRSkpICsNCiAgZ2VvbV92bGluZSh4aW50ZXJjZXB0ID0gc2lnbWEpKw0KICB0aGVtZV9idygpKw0KICBndWlkZXMoc2l6ZSA9IGd1aWRlX2xlZ2VuZChvcmRlciA9IDMsIHJldmVyc2UgPSBUUlVFKSwNCiAgICAgICAgIHNoYXBlID0gZ3VpZGVfbGVnZW5kKG9yZGVyID0gNCwNCiAgICAgICAgICAgICAgICAgICAgICAgICAgICAgIG92ZXJyaWRlLmFlcyA9IGxpc3Qoc2l6ZSA9IDMpKSwNCiAgICAgICAgIGZpbGwgPSBndWlkZV9sZWdlbmQob3JkZXIgPSA1LCByZXZlcnNlID0gVFJVRSwNCiAgICAgICAgICAgICAgICAgICAgICAgICAgICAgIG92ZXJyaWRlLmFlcyA9IGxpc3Qoc2hhcGUgPSAxNSwgDQogICAgICAgICAgICAgICAgICAgICAgICAgICAgICAgICAgICAgICAgICAgICAgICAgIHNpemUgPSA2LCANCiAgICAgICAgICAgICAgICAgICAgICAgICAgICAgICAgICAgICAgICAgICAgICAgICAgY29sb3IgPSBjKCIjNTZCNEU5IiwiI0Q1NUUwMCIpLA0KICAgICAgICAgICAgICAgICAgICAgICAgICAgICAgICAgICAgICAgICAgICAgICAgICBhbHBoYSA9IDEpKSwNCiAgICAgICAgIGxpbmV0eXBlID0gZ3VpZGVfbGVnZW5kKG9yZGVyID0gMSwgcmV2ZXJzZSA9IFRSVUUpLA0KICAgICAgICAgYWxwaGEgPSBndWlkZV9sZWdlbmQob3JkZXIgPSAyKSkrDQogIHRoZW1lKGF4aXMudGl0bGUgPSBlbGVtZW50X3RleHQoc2l6ZSA9IDE0LCBmYWNlID0gImJvbGQiKSwNCiAgICAgICAgYXhpcy50ZXh0ID0gZWxlbWVudF90ZXh0KHNpemUgPSAxMiwgZmFjZSA9ICJib2xkIiksDQogICAgICAgIGxlZ2VuZC50aXRsZSA9IGVsZW1lbnRfdGV4dChzaXplID0gMTIsIGZhY2UgPSAiYm9sZCIpLA0KICAgICAgICBsZWdlbmQudGV4dCA9IGVsZW1lbnRfdGV4dChzaXplID0gMTIsIGZhY2UgPSAiYm9sZCIpLA0KICAgICAgICBsZWdlbmQua2V5LndpZHRoID0gdW5pdCgzLCJsaW5lIikpDQoNCmZpZ3MxYiA8LSBnZ3Bsb3QoZGF0YSA9IGxyZXhiZXRhdGFiLCBhZXMoeCA9IHNpZ21hLCB5ID0gUmVsQmlhc19keikpKyANCiAgZ2VvbV9qaXR0ZXIoYWVzKHNoYXBlID0gYXMuZmFjdG9yKGxvZ2Jhc2UpLCBzaXplID0gbWVkbGlzdCwgZmlsbCA9IGFzLmNoYXJhY3Rlcihub2JzKSksDQogICAgICAgICAgICAgIGNvbG9yID0gImJsYWNrIg0KICAgICAgICAgICAgICAjICwgYWxwaGEgPSAwLjUNCiAgICAgICAgICAgICAgKSsNCiAgZ2VvbV9saW5lKGRhdGEgPSBscmV4YmV0YXRhYltyZWwgPT0gImxvZyJdLCANCiAgICAgICAgICAgIHN0YXQgPSAnc21vb3RoJywNCiAgICAgICAgICAgIG1ldGhvZCA9ICdsbScsDQogICAgICAgICAgICAgIGZvcm11bGEgPSB5fnBvbHkoeCwyLHJhdz1UKSwNCiAgICAgICAgICAgICAgYWVzKGFscGhhID0gcG9zYmV0YSwNCiAgICAgICAgICAgICAgICAgIGNvbG9yID0gYXMuY2hhcmFjdGVyKG5vYnMpKSwNCiAgICAgICAgICAgICAgc2l6ZSA9IDEuMSkgKw0KICBzY2FsZV94X2NvbnRpbnVvdXMobmFtZSA9ICJTaWdtYSIsYnJlYWtzID0gYygwLjI1LDAuNDUsMC42NSwwLjg1KSkrDQogIHNjYWxlX3lfY29udGludW91cyhuYW1lID0gIlJlbGF0aXZlIEJpYXMgVmFsdWUgKER6aWVybGVuZ2EgTWV0aG9kKSIsIGxpbSA9IGMoLTEuMjUsMS4yNSkpKw0KICBzY2FsZV9jb2xvcl9tYW51YWwobmFtZSA9ICJOdW1iZXIgb2YgT2JzZXJ2YXRpb25zIiwNCiAgICAgICAgICAgICAgICAgICAgIHZhbHVlcyA9IGMoIiNENTVFMDAiLCIjNTZCNEU5IiksDQogICAgICAgICAgICAgICAgICAgICBndWlkZSA9ICJub25lIiNndWlkZV9sZWdlbmQocmV2ZXJzZSA9IFRSVUUpDQogICAgICAgICAgICAgICAgICAgICApKw0KICBzY2FsZV9hbHBoYV9tYW51YWwobmFtZSA9ICJGaXR0ZWQgQ3VydmVzIGZvciBTaWduIG9mIEJldGEiLA0KICAgICAgICAgICAgICAgICAgICAgdmFsdWVzID0gYygwLjUsMSkpKw0KICBzY2FsZV9maWxsX21hbnVhbChuYW1lID0gIk51bWJlciBvZiBPYnNlcnZhdGlvbnNcbigrIEZpdHRlZCBDdXJ2ZXMpIiwNCiAgICAgICAgICAgICAgICAgICAgdmFsdWVzID0gYWxwaGEoYygiI0Q1NUUwMCIsIiM1NkI0RTkiKSxjKDEsMSkpKSsNCiAgc2NhbGVfc2hhcGVfbWFudWFsKG5hbWUgPSAiTG9nYXJpdGhtIEJhc2UiLA0KICAgICAgICAgICAgICAgICAgICAgICBicmVha3MgPSBjKDIsImUiLDEwKSwNCiAgICAgICAgICAgICAgICAgICAgICAgbGFiZWxzID0gYygiMiIsImUiLCIxMCIpLA0KICAgICAgICAgICAgICAgICAgICAgICAjIHZhbHVlcyA9IGMoMTUsMTgsMjApDQogICAgICAgICAgICAgICAgICAgICAgIHZhbHVlcyA9IGMoMjEsMjIsMjQpKSsNCiAgc2NhbGVfc2l6ZShyYW5nZSA9IGMoMSw0KSxuYW1lID0gIk1lZGlhbiBWYWx1ZSIsDQogICAgICAgICAgICAgZ3VpZGUgPSBndWlkZV9sZWdlbmQocmV2ZXJzZSA9IFRSVUUpKSArDQogIGdlb21fdmxpbmUoeGludGVyY2VwdCA9IHNpZ21hKSsNCiAgdGhlbWVfYncoKSsNCiAgZ3VpZGVzKHNpemUgPSBndWlkZV9sZWdlbmQob3JkZXIgPSAxLCByZXZlcnNlID0gVFJVRSksDQogICAgICAgICBzaGFwZSA9IGd1aWRlX2xlZ2VuZChvcmRlciA9IDIsDQogICAgICAgICAgICAgICAgICAgICAgICAgICAgICBvdmVycmlkZS5hZXMgPSBsaXN0KHNpemUgPSAzKSksDQogICAgICAgICBmaWxsID0gZ3VpZGVfbGVnZW5kKG9yZGVyID0gMywNCiAgICAgICAgICAgICAgICAgICAgICAgICAgICAgcmV2ZXJzZSA9IFRSVUUsDQogICAgICAgICAgICAgICAgICAgICAgICAgICAgIG92ZXJyaWRlLmFlcyA9IGxpc3QoY29sb3IgPSBjKCIjNTZCNEU5IiwiI0Q1NUUwMCIpLA0KICAgICAgICAgICAgICAgICAgICAgICAgICAgICAgICAgICAgICAgICAgICAgICAgIGFscGhhID0gMSwNCiAgICAgICAgICAgICAgICAgICAgICAgICAgICAgICAgICAgICAgICAgICAgICAgICBzaXplID0gNiwNCiAgICAgICAgICAgICAgICAgICAgICAgICAgICAgICAgICAgICAgICAgICAgICAgICBzaGFwZSA9IDE1KSkpKw0KICB0aGVtZShheGlzLnRpdGxlID0gZWxlbWVudF90ZXh0KHNpemUgPSAxNCwgZmFjZSA9ICJib2xkIiksIA0KICAgICAgICBheGlzLnRleHQgPSBlbGVtZW50X3RleHQoc2l6ZSA9IDEyLCBmYWNlID0gImJvbGQiKSwNCiAgICAgICAgbGVnZW5kLnRpdGxlID0gZWxlbWVudF90ZXh0KHNpemUgPSAxMiwgZmFjZSA9ICJib2xkIiksDQogICAgICAgIGxlZ2VuZC50ZXh0ID0gZWxlbWVudF90ZXh0KHNpemUgPSAxMiwgZmFjZSA9ICJib2xkIiksDQogICAgICAgIGxlZ2VuZC5rZXkud2lkdGggPSB1bml0KDMsImxpbmUiKSkNCg0KZ2dhcnJhbmdlKGZpZ3MxYSxmaWdzMWIsZmlnczFjKQ0KDQojIENyZWF0ZXMgZmlndXJlIHN5LS0gVW5jb21tZW50IHRvIGdlbmVyYXRlDQojIHZvaWRwbHQgPC0gZ2dwbG90KCkgKyB0aGVtZV92b2lkKCkNCiMgcGRmKCJmaWd1cmVzeS5wZGYiLHdpZHRoID0gMTUsIGhlaWdodCA9IDgpDQojIGdncHVicjo6Z2dhcnJhbmdlKGZpZ3MxYSxmaWdzMWIsIGxhYmVscyA9IGMoIkEiLCJCIiksIGFsaWduID0gInYiKQ0KIyBnZ3B1YnI6OmdnYXJyYW5nZShmaWdzMWMsdm9pZHBsdCwgbGFiZWxzID0gYygiQyIsIiIpLCBhbGlnbiA9ICJ2IikNCiMgZGV2Lm9mZigpDQpgYGANCg0KIyMgVGFibGUgUzENCg0KYGBge3IgdGFiczFnZW59DQp0YWJzMSA8LSBkYXRhLnRhYmxlKG1ldGhvZCA9ICJSb2RyaWd1ZXotQmFycmFuY28gTWV0aG9kIiwgDQogICAgICAgICAgICAgICAgICAgICAgICAgICBwYXJhbWV0ZXIgPSBuYW1lcyhzdGVwLjJwNiRjb2VmZmljaWVudHNbIWlzLm5hKHN0ZXAuMnA2JGNvZWZmaWNpZW50cyldKSwgDQogICAgICAgICAgICAgICAgICAgICAgICAgICBjb2VmZmljaWVudCA9IHN0ZXAuMnA2JGNvZWZmaWNpZW50c1shaXMubmEoc3RlcC4ycDYkY29lZmZpY2llbnRzKV0sIA0KICAgICAgICAgICAgICAgICAgICAgICAgICAgc2UgPSBzdW1tYXJ5KHN0ZXAuMnA2KSRjb2VmZmljaWVudHNbLDJdLCANCiAgICAgICAgICAgICAgICAgICAgICAgICAgIHAgPSBzdW1tYXJ5KHN0ZXAuMnA2KSRjb2VmZmljaWVudHNbLDRdLA0KICAgICAgICAgICAgICAgICAgICAgICAgICAgcm1zZSA9IHNxcnQobWVhbihzdGVwLjJwNiRyZXNpZHVhbHNeMikpLA0KICAgICAgICAgICAgICAgICAgICAgICAgICAgcjIgPSBzdW1tYXJ5KHN0ZXAuMnA2KSRhZGouci5zcXVhcmVkKQ0KDQp0YWJzMSA8LSByYmluZCh0YWJzMSwNCiAgICAgICAgICAgICAgICBkYXRhLnRhYmxlKG1ldGhvZCA9ICJEemllcmxlbmdhIE1ldGhvZCIsIA0KICAgICAgICAgICAgICAgICAgICAgICAgICAgcGFyYW1ldGVyID0gbmFtZXMoc3RlcC4ycDckY29lZmZpY2llbnRzWyFpcy5uYShzdGVwLjJwNyRjb2VmZmljaWVudHMpXSksIA0KICAgICAgICAgICAgICAgICAgICAgICAgICAgY29lZmZpY2llbnQgPSBzdGVwLjJwNyRjb2VmZmljaWVudHNbIWlzLm5hKHN0ZXAuMnA3JGNvZWZmaWNpZW50cyldLCANCiAgICAgICAgICAgICAgICAgICAgICAgICAgIHNlID0gc3VtbWFyeShzdGVwLjJwNykkY29lZmZpY2llbnRzWywyXSwgDQogICAgICAgICAgICAgICAgICAgICAgICAgICBwID0gc3VtbWFyeShzdGVwLjJwNykkY29lZmZpY2llbnRzWyw0XSwNCiAgICAgICAgICAgICAgICAgICAgICAgICAgIHJtc2UgPSBzcXJ0KG1lYW4oc3RlcC4ycDckcmVzaWR1YWxzXjIpKSwNCiAgICAgICAgICAgICAgICAgICAgICAgICAgIHIyID0gc3VtbWFyeShzdGVwLjJwNykkYWRqLnIuc3F1YXJlZCksDQogICAgICAgICAgICAgICBkYXRhLnRhYmxlKG1ldGhvZCA9ICJBbHRlcm5hdGl2ZSBNZXRob2QiLCANCiAgICAgICAgICAgICAgICAgICAgICAgICAgIHBhcmFtZXRlciA9IG5hbWVzKHN0ZXAuMnA1JGNvZWZmaWNpZW50c1shaXMubmEoc3RlcC4ycDUkY29lZmZpY2llbnRzKV0pLCANCiAgICAgICAgICAgICAgICAgICAgICAgICAgIGNvZWZmaWNpZW50ID0gc3RlcC4ycDUkY29lZmZpY2llbnRzWyFpcy5uYShzdGVwLjJwNSRjb2VmZmljaWVudHMpXSwgDQogICAgICAgICAgICAgICAgICAgICAgICAgICBzZSA9IHN1bW1hcnkoc3RlcC4ycDUpJGNvZWZmaWNpZW50c1ssMl0sIA0KICAgICAgICAgICAgICAgICAgICAgICAgICAgcCA9IHN1bW1hcnkoc3RlcC4ycDUpJGNvZWZmaWNpZW50c1ssNF0sDQogICAgICAgICAgICAgICAgICAgICAgICAgICBybXNlID0gc3FydChtZWFuKHN0ZXAuMnA1JHJlc2lkdWFsc14yKSksDQogICAgICAgICAgICAgICAgICAgICAgICAgICByMiA9IHN1bW1hcnkoc3RlcC4ycDUpJGFkai5yLnNxdWFyZWQpKQ0KDQojIHRhYnMxIDwtIGtuaXRyOjprYWJsZSh0YWJzMSkgI0ZvciBhIHNsaWdodGx5IGRpZmZlcmVudCBmb3JtYXQNCg0KdGFiczENCmBgYA0KDQojIyBUYWJsZSBTMg0KDQpgYGB7cn0NCiNDcmVhdGluZyBUYWJsZSBjb21wYXJpbmcgUl4yIGZvciA3NjggdnMuIDc2OCoyMDAwDQp0YWJzMiA8LSBkYXRhLmZyYW1lKCJyc3EiID0gYygibiA9IDc2OCIsDQogICAgICAgICAgICAgICAgICAgICAgICAgICAgICAgICAgICAibiA9IDc2OCAqIDIwMDAiKSwNCiAgICAgICAgICAgICAgICAgICAicmIiID0gYyhzdW1tYXJ5KHN0ZXAuMnA2KSRhZGouci5zcXVhcmVkLA0KICAgICAgICAgICAgICAgICAgICAgICAgICAgICAgICAgICBzdW1tYXJ5KHN0ZXAuMnA2ZikkYWRqLnIuc3F1YXJlZCksDQogICAgICAgICAgICAgICAgICAgImR6IiA9IGMoc3VtbWFyeShzdGVwLjJwNykkYWRqLnIuc3F1YXJlZCwNCiAgICAgICAgICAgICAgICAgICAgICAgICAgICAgICAgICAgc3VtbWFyeShzdGVwLjJwN2YpJGFkai5yLnNxdWFyZWQpLA0KICAgICAgICAgICAgICAgICAgICJhbHQiID1jKHN1bW1hcnkoc3RlcC4ycDUpJGFkai5yLnNxdWFyZWQsDQogICAgICAgICAgICAgICAgICAgICAgICAgICAgICAgICAgIHN1bW1hcnkoc3RlcC4ycDVmKSRhZGouci5zcXVhcmVkKSkNCm5hbWVzKHRhYnMyKSA8LSBjKCJyXjJeIGZvcjoiLCJcdTAzYjJ+UkJ+IiwiXHUwM2IyfkR6fiIsIlx1MDNiMn5BbHR+IikNCg0KdGFiczIgPC0ga25pdHI6OmthYmxlKHRhYnMyKQ0KDQp0YWJzMg0KYGBgDQoNCiMjIFRhYmxlIFMzIA0KDQpUYWJsZSBTMyB3YXMgZ2VuZXJhdGVkIGJ5IGhhbmQgYW5kIHNvIGlzIG5vdCBwcm9kdWNlZCBoZXJlLg0KDQojIyBUYWJsZSBTNA0KDQpgYGB7ciB0YWJzNGdlbn0NCnRhYnM0ZGYgPC0gZGF0YVtkYXRhJGlucHV0X2Jhc2UgPT0gImxvZyIsXQ0KdGFiczRkZiA8LSB0YWJzNGRmW29yZGVyKHRhYnM0ZGYkc3R1ZHlfaWQpLF0NCnRhYnM0IDwtIGRhdGEuZnJhbWUoIlN0dWR5LCBZZWFyIiA9IHRhYnM0ZGYkc3R1ZHlfaWQsDQogICAgICAgICAgICAgICAgICAgIlNwZWNpZmljIEZpbmRpbmcvbihMb2NhdGlvbiwgT3V0Y29tZSkiID0NCiAgICAgICAgICAgICAgICAgICAgIGMoIk91ciBhbmFseXNpcywgSGliIElnRyIsDQogICAgICAgICAgICAgICAgICAgICAgICJUYWJsZSAzIChGdWxseSBBZGp1c3RlZCksIEJpcnRoIFdlaWdodCAoZykiLA0KICAgICAgICAgICAgICAgICAgICAgICAiVGFibGUgMyAoMjAtNDl5KSwgSFNWIDIiLA0KICAgICAgICAgICAgICAgICAgICAgICAiVGFibGUgMywgVHJpZ2x5Y2VyaWRlcyAobWcvZEwpIiwNCiAgICAgICAgICAgICAgICAgICAgICAgIlRhYmxlIDMgKEFkanVzdGVkKSwgQmlydGggV2VpZ2h0IChnKSIsDQogICAgICAgICAgICAgICAgICAgICAgICJUYWJsZSA2IChBZGp1c3RlZCBBbGwgQmlydGhzLCBQZXIgaW4gdW5pdCBpbmNyZWFzZSksIEJpcnRoIFdlaWdodCAoZykiLA0KICAgICAgICAgICAgICAgICAgICAgICAiVGFibGUgNSAoSGFtbSwgUEZPUyksIEJpcnRoIFdlaWdodCAoZykiLA0KICAgICAgICAgICAgICAgICAgICAgICAiVGFibGUgMiAoTW9kZWwgMiksIEluZmVydGlsaXR5IiwNCiAgICAgICAgICAgICAgICAgICAgICAgIkZpZ3VyZSAxYSAoTUJ6UCksIEFzdGhtYSIsDQogICAgICAgICAgICAgICAgICAgICAgICJUYWJsZSA0LCBSdWJlbGxhICglzpQpIiwNCiAgICAgICAgICAgICAgICAgICAgICAgIlRhYmxlIDQsIFRvdGFsIENob2xlc3Rlcm9sIiwNCiAgICAgICAgICAgICAgICAgICAgICAgIlRhYmxlIDIsIE11bXBzICglzpQpIiwNCiAgICAgICAgICAgICAgICAgICAgICAgIlRhYmxlIDUgKEZ1bGx5IEFkanVzdGVkKSwgQmlydGggV2VpZ2h0IChnKSIsDQogICAgICAgICAgICAgICAgICAgICAgICJUYWJsZSAyIChNb2RlbCAxKSwgQ1ZEIiwNCiAgICAgICAgICAgICAgICAgICAgICAgIlRhYmxlIDQgKE1vZGVsIDIpLCBUcmlnbHljZXJpZGVzIChtZy9kTCkiKSwNCiAgICAgICAgICAgICAgICAgICAiTWVkaWFuIiA9IHRhYnM0ZGYkZXhwX21lZCwNCiAgICAgICAgICAgICAgICAgICAiMXN0IFF1YXJ0aWxlIiA9IHRhYnM0ZGYkZXhwXzFxLA0KICAgICAgICAgICAgICAgICAgICIzcmQgUXVhcnRpbGUiID0gdGFiczRkZiRleHBfM3EsDQogICAgICAgICAgICAgICAgICAgIlNpZ21hIChsb2dub3JtYWwgZGlzdHJpYnV0aW9uKSIgPSB0YWJzNGRmJHNpZ21hLA0KICAgICAgICAgICAgICAgICAgICJNZWFuIiA9IHRhYnM0ZGYkZXhwX21lYW4pDQpuYW1lcyh0YWJzNCkgPC0gYygiU3R1ZHksIFllYXIiLCJTcGVjaWZpYyBGaW5kaW5nIChMb2NhdGlvbiwgT3V0Y29tZSkiLCJNZWRpYW4iLCIxc3QgUXVhcnRpbGUiLCIzcmQgUXVhcnRpbGUiLCAiU2lnbWEgKGxvZ25vcm1hbCBkaXN0cmlidXRpb24pIiwiTWVhbiIpDQoNCnRhYnM0IDwtIGtuaXRyOjprYWJsZSh0YWJzNCkNCg0KdGFiczQNCmBgYA0KIyMgVGFibGUgUzRiDQoNCmBgYHtyIHRhYnM0Ymdlbn0NCnRhYnM0ZGZiIDwtIGRhdGFiW2RhdGFiJGlucHV0X2Jhc2UgPT0gImxvZyIsXQ0KdGFiczRkZmIgPC0gdGFiczRkZmJbb3JkZXIodGFiczRkZmIkc3R1ZHlfaWQpLF0NCnRhYnM0YiA8LSBkYXRhLmZyYW1lKCJTdHVkeSwgWWVhciIgPSB0YWJzNGRmYiRzdHVkeV9pZCwNCiAgICAgICAgICAgICAgICAgICAiU3BlY2lmaWMgRmluZGluZy9uKExvY2F0aW9uLCBPdXRjb21lKSIgPQ0KICAgICAgICAgICAgICAgICAgICAgYygiT3VyIGFuYWx5c2lzLCBEaXBodGhlcmlhIElnRywgUEZPQSIsDQogICAgICAgICAgICAgICAgICAgICAgICJUYWJsZSAzIChGdWxseSBBZGp1c3RlZCksIFBvbmRlcmFsIEluZGV4IChnIGNtMyB4IDEwMCksIFBGT0EiLA0KICAgICAgICAgICAgICAgICAgICAgICAiVGFibGUgMyAoMjAtNDkgeSksIFRveG9jYXJhLCBQRk9TIiwNCiAgICAgICAgICAgICAgICAgICAgICAgIlRhYmxlIDMsIExETCAobWcvZEwpLCBIYkFBK0hiR0EiLA0KICAgICAgICAgICAgICAgICAgICAgICAiVGFibGUgMyAoQWRqdXN0ZWQpLCBCaXJ0aCBXZWlnaHQgKGcpLCBQRk9BIiwNCiAgICAgICAgICAgICAgICAgICAgICAgIlRhYmxlIDQgKEFkanVzdGVkIEZpcnN0IFByb3NwZWN0aXZlIEJpcnRocywgUGVyIGxuIHVuaXQgaW5jcmVhc2UpLCANCiAgICAgICAgICAgICAgICAgICAgICAgUHJlZ25hbmN5IEluZHVjZWQgSHlwZXJ0ZW5zaW9uLCBQRk9TIiwNCiAgICAgICAgICAgICAgICAgICAgICAgIlAgNTkyLCBsZWZ0IGNvbHVtbiwgQmlydGggV2VpZ2h0IChnKSwgUEZIeFMiLA0KICAgICAgICAgICAgICAgICAgICAgICAiVGFibGUgMiAoTW9kZWwgMiksIEluZmVydGlsaXR5LCBMZWFkIiwNCiAgICAgICAgICAgICAgICAgICAgICAgIkZpZ3VyZSAzYiwgQXN0aG1hLCDiiJFERUhQICjCtW1vbC9MKSIsDQogICAgICAgICAgICAgICAgICAgICAgICJUYWJsZSA0LCBSdWJlbGxhICglzpQpLCBQRk9TIiwNCiAgICAgICAgICAgICAgICAgICAgICAgIlRhYmxlIDQsIEhETCwgUEZPQSIsDQogICAgICAgICAgICAgICAgICAgICAgICJUYWJsZSAyIChBbGwpLCBNdW1wcyAoJc6UKSwgUEZIeFMiLA0KICAgICAgICAgICAgICAgICAgICAgICAiVGFibGUgMiAoTW9kZWwgMiksIENWRCwgUHJvcGFuYWxkZWh5ZGUiLA0KICAgICAgICAgICAgICAgICAgICAgICAiVGFibGUgNCAoTW9kZWwgMiksIE1vbm9jeXRlcywgSXNvcGVudGFuYWxkZWh5ZGUiLA0KICAgICAgICAgICAgICAgICAgICAgICAiVGFibGUgNSAoRnVsbHkgQWRqdXN0ZWQpLCBCaXJ0aCBXZWlnaHQgKGcpLCBQRk9BIChGcm9tIFZlcm5lciBldCBhbC4gMjAxNSkiKSwNCiAgICAgICAgICAgICAgICAgICAiTWVkaWFuIiA9IHRhYnM0ZGZiJGV4cF9tZWQsDQogICAgICAgICAgICAgICAgICAgIjFzdCBRdWFydGlsZSIgPSB0YWJzNGRmYiRleHBfMXEsDQogICAgICAgICAgICAgICAgICAgIjNyZCBRdWFydGlsZSIgPSB0YWJzNGRmYiRleHBfM3EsDQogICAgICAgICAgICAgICAgICAgIlNpZ21hIChsb2dub3JtYWwgZGlzdHJpYnV0aW9uKSIgPSB0YWJzNGRmYiRzaWdtYSwNCiAgICAgICAgICAgICAgICAgICAiTWVhbiIgPSB0YWJzNGRmYiRleHBfbWVhbikNCm5hbWVzKHRhYnM0YikgPC0gYygiU3R1ZHksIFllYXIiLCJTcGVjaWZpYyBGaW5kaW5nIChMb2NhdGlvbiwgT3V0Y29tZSkiLCJNZWRpYW4iLCIxc3QgUXVhcnRpbGUiLCIzcmQgUXVhcnRpbGUiLCAiU2lnbWEgKGxvZ25vcm1hbCBkaXN0cmlidXRpb24pIiwiTWVhbiIpDQoNCnRhYnM0YiA8LSBrbml0cjo6a2FibGUodGFiczRiKQ0KDQp0YWJzNGINCmBgYA0KDQojIyBUYWJsZSBTNSANCg0KVGFibGUgUzUgd2FzIGdlbmVyYXRlZCBieSBoYW5kIGFuZCBzbyBpcyBub3QgcHJvZHVjZWQgaGVyZS4NCg0KIyMgVGFibGUgUzYNCg0KRmluYWxseSwgd2UgaW52ZXN0aWdhdGVkIHRoZSBlZmZlY3RzIG9mIGEgZmV3IHZlcnkgaW5mbHVlbnRpYWwgcG9pbnRzIGluIDQgc3R1ZGllcyBhbmQgd2hldGhlciBkcm9wcGluZyB0aG9zZSBwb2ludHMgbWFkZSBhbnkgb2YgdGhlIG1ldGhvZHMgbW9yZSBhY2N1cmF0ZSBpbiB0aGVpciByZS1leHByZXNzaW9uIG9mIEJldGEgdmFsdWVzLiBUaGUgZmlyc3Qgc3RlcCBpbiB0aGlzIHBhcnQgb2YgdGhlIGFuYWx5c2lzIGlzIHRvIHJlYWQgaW4gdGhlIGRhdGEgZmlsZXMgZm9yIHRoZSBzdHVkaWVzIG9mIGludGVyZXN0IGFuZCBzZXQgYSBjb3VwbGUgb3B0aW9ucyBmb3IgdGhlIHN1cnZleSBkZXNpZ24gKHdlaWdodGluZywgZXRjLikgYW5kIGxpbmVhciByZWdyZXNzaW9uLg0KDQpgYGB7ciwgbWVzc2FnZSA9IEZBTFNFfQ0KZGYxIDwtIHJlYWQuY3N2KGZpbGUgPSAiZGF0YS9PZGViZWF0dV8yMDE5LmNzdiIpDQpkZjIgPC0gcmVhZC5jc3YoZmlsZSA9ICJkYXRhL1BpbGtlcnRvbl8yMDE4LmNzdiIpDQpkZjMgPC0gcmVhZC5jc3YoZmlsZSA9ICJkYXRhL0NoZWFuZ18yMDIxdjIuY3N2IikNCmRmNCA8LSByZWFkLmNzdihmaWxlID0gImRhdGEvWHVfMjAyMC5jc3YiKQ0Kb3B0aW9ucyhzdXJ2ZXkuYWRqdXN0LmRvbWFpbi5sb25lbHk9VFJVRSkNCm9wdGlvbnMoc3VydmV5LmxvbmVseS5wc3U9ImFkanVzdCIpDQpgYGANCg0KV2UgdGhlbiBhc3NpZ24gYSBzdXJ2ZXkgZGVzaWduIHRvIGVhY2ggc3R1ZHkgdXNpbmcgdGhlICJzdXJ2ZXkiIHBhY2thZ2UgYW5kIHRoZSBpbmNsdWRlZCBpZHMgKFNETVZQU1UpLCB3ZWlnaHRzLCAod2d0LCB3Z3QzKSBhbmQgc3RyYXRhIChTRE1WU1RSQSkuIA0KDQpgYGB7cn0NCm9kZSA8LSBzdnlkZXNpZ24oaWQgPSB+U0RNVlBTVSwgd2VpZ2h0cyA9IH53Z3QsIHN0cmF0YSA9IH5TRE1WU1RSQSwgbmVzdCA9IFRSVUUsIGRhdGEgPSBkZjEpDQpwaWwgPC0gc3Z5ZGVzaWduKGlkID0gflNETVZQU1UsIHdlaWdodHMgPSB+d2d0Mywgc3RyYXRhID0gflNETVZTVFJBLCBuZXN0ID0gVFJVRSwgZGF0YSA9IGRmMikNCmNoZSA8LSBzdnlkZXNpZ24oaWQgPSB+U0RNVlBTVSwgd2VpZ2h0cyA9IH53Z3QsIHN0cmF0YSA9IH5TRE1WU1RSQSwgbmVzdCA9IFRSVUUsIGRhdGEgPSBkZjMpDQp4dSA8LSBzdnlkZXNpZ24oaWQgPSB+U0RNVlBTVSwgd2VpZ2h0cyA9IH53Z3QsIHN0cmF0YSA9IH5TRE1WU1RSQSwgbmVzdCA9IFRSVUUsIGRhdGEgPSBkZjQpDQpgYGANCg0KTmV4dCwgd2UgY3JlYXRlIGFuIGVtcHR5IGxpc3QgZm9yIHRoZSBnZW5lcmFsaXplZCBsaW5lYXIgbW9kZWxzIChnbG0pLCBhbmQgcnVuIGEgbXVsdGlwbGUgbGluZWFyIHJlZ3Jlc3Npb24gZm9yIGVhY2ggc2NlbmFyaW8gKDQgc3R1ZGllcyB3LyBsb2cgdG8gbGluZWFyIHJlLWV4cHJlc3Npb24gKyA0IHN0dWRpZXMgd2l0aCBsaW5lYXIgdG8gbG9nIHJlLWV4cHJlc3Npb24pLCB1c2luZyB0aGUgYXBwcm9wcmlhdGUgZXhwbGFuYXRvcnkgdmFyaWFibGVzIGFzIG5vdGVkIGluIHRoZSBvcmlnaW5hbCBzdHVkaWVzLg0KDQpgYGB7cn0NCmdsbSA8LSBsaXN0KCkNCmdsbVtbMV1dIDwtIHN2eWdsbShhc3RobWF+TUJ6UCwgZmFtaWx5ID0gcXVhc2liaW5vbWlhbCwgZGVzaWduID0gb2RlKQ0KZ2xtW1syXV0gPC0gc3Z5Z2xtKGFzdGhtYX5sMTBNQnpQLCBmYW1pbHkgPSBxdWFzaWJpbm9taWFsLCBkZXNpZ24gPSBvZGUpDQpnbG1bWzNdXSA8LSBzdnlnbG0obG9ncnViZWxsYX5wZm9hICsgUklBR0VORFIgKyBSSURBR0VZUiArIGVkMSArIGVkMiArIEJNWEJNSSArIGV0aDEgKyBldGgyLCBkZXNpZ24gPSBwaWwpDQpnbG1bWzRdXSA8LSBzdnlnbG0obG9ncnViZWxsYX5sZzJwZm9hICsgUklBR0VORFIgKyBSSURBR0VZUiArIGVkMSArIGVkMiArIEJNWEJNSSArIGV0aDEgKyBldGgyLCBkZXNpZ24gPSBwaWwpDQpnbG1bWzVdXSA8LSBzdnlnbG0oTEJYVFJ+SEJnYSArIGZlbWFsZSArIFJJREFHRVlSICsgSU5ERk1QSVIgKyBzbWsxICsgc21rMiArIGRpYWJldGVzX3JldiArIGh5cGVyX3JldiArIGVuZXJneSArIGV0aDEgKyBldGgyICsgZXRoMyArIGV0aDQgKyBlZHUxICsgZWR1MiArIGVkdTMgKyBlZHU1ICsgYngxICsgYngyICsgcGFxbm9uZSArIHBhcW1vZCArIGFsY29ob2wsIGRlc2lnbiA9IGNoZSkNCmdsbVtbNl1dIDwtIHN2eWdsbShMQlhUUn5sMmdhICsgZmVtYWxlICsgUklEQUdFWVIgKyBJTkRGTVBJUiArIHNtazEgKyBzbWsyICsgZGlhYmV0ZXNfcmV2ICsgaHlwZXJfcmV2ICsgZW5lcmd5ICsgZXRoMSArIGV0aDIgKyBldGgzICsgZXRoNCArIGVkdTEgKyBlZHUyICsgZWR1MyArIGVkdTUgKyBieDEgKyBieDIgKyBwYXFub25lICsgcGFxbW9kICsgYWxjb2hvbCwgZGVzaWduID0gY2hlKQ0KZ2xtW1s3XV0gPC0gc3Z5Z2xtKGN2ZH5MQlhJNUFMICsgUklEQUdFWVIgKyBldGgxICsgZXRoMiArIGV0aDQgKyBldGg1ICsgZWR1MiArIGVkdTMgKyBlZHU0ICsgZWR1NSArIGZlbWFsZSwgZmFtaWx5ID0gcXVhc2liaW5vbWlhbCwgZGVzaWduID0geHUpDQpnbG1bWzhdXSA8LSBzdnlnbG0oY3ZkfmwyaTVhbCArIFJJREFHRVlSICsgZXRoMSArIGV0aDIgKyBldGg0ICsgZXRoNSArIGVkdTIgKyBlZHUzICsgZWR1NCArIGVkdTUgKyBmZW1hbGUsIGZhbWlseSA9IHF1YXNpYmlub21pYWwsIGRlc2lnbiA9IHh1KQ0KYGBgDQoNClRoZW4sIHVzaW5nIHRoZSBkZmJldGFzIGZ1bmN0aW9uLCB3ZSBpZGVudGlmeSB0aGUgaW5mbHVlbmNlIG9mIGVhY2ggZGF0YSBwb2ludCBvbiB0aGUgbXVsdGktbGluZWFyIHJlZ3Jlc3Npb24gZm9yIGVhY2ggc2NlbmFyaW8uIFBvaW50cyBhcmUgY29uc2lkZXJlZCBpbmZsdWVudGlhbCAoYW5kIGFyZSB0aGVyZWZvcmUgcmVtb3ZlZCBmcm9tIHRoZSBkYXRhIHN1YnNldCkgaWYgZGZiZXRhID4gMi9zcXJ0KG4pLg0KDQpgYGB7cn0NCmRmYiA8LSBsaXN0KGRmMSwgZGYxLCBkZjIsIGRmMiwgZGYzLCBkZjMsIGRmNCwgZGY0KQ0KZGZzdWIgPC0gbGlzdCgpDQpmb3IoaSBpbiAxOmxlbmd0aChnbG0pKXsNCiAgZGZiW1tpXV0kZGZiZXRhcyA8LSAwDQogIGRmYltbaV1dW2NvbXBsZXRlLmNhc2VzKGRmYltbaV1dKSxdJGRmYmV0YXMgPC0gZGZiZXRhcyhnbG1bW2ldXSlbLDJdDQogIGRmc3ViW1tpXV0gPC0gZGZiW1tpXV1bYWJzKGRmYltbaV1dJGRmYmV0YXMpIDw9IDIvc3FydChucm93KGRmYltbaV1dKSksXQ0KfQ0KDQpgYGANCg0KTm93IHdlIHJlLWNyZWF0ZSB0aGUgc3VydmV5IGRlc2lnbnMuLi4NCg0KYGBge3J9DQpvZGVzdWIgPC0gc3Z5ZGVzaWduKGlkID0gflNETVZQU1UsIHdlaWdodHMgPSB+d2d0LCBzdHJhdGEgPSB+U0RNVlNUUkEsIG5lc3QgPSBUUlVFLCBkYXRhID0gZGZzdWJbWzFdXSkNCm9kZXN1YmwgPC0gc3Z5ZGVzaWduKGlkID0gflNETVZQU1UsIHdlaWdodHMgPSB+d2d0LCBzdHJhdGEgPSB+U0RNVlNUUkEsIG5lc3QgPSBUUlVFLCBkYXRhID0gZGZzdWJbWzJdXSkNCnBpbHN1YiA8LSBzdnlkZXNpZ24oaWQgPSB+U0RNVlBTVSwgd2VpZ2h0cyA9IH53Z3QzLCBzdHJhdGEgPSB+U0RNVlNUUkEsIG5lc3QgPSBUUlVFLCBkYXRhID0gZGZzdWJbWzNdXSkNCnBpbHN1YmwgPC0gc3Z5ZGVzaWduKGlkID0gflNETVZQU1UsIHdlaWdodHMgPSB+d2d0Mywgc3RyYXRhID0gflNETVZTVFJBLCBuZXN0ID0gVFJVRSwgZGF0YSA9IGRmc3ViW1s0XV0pDQpjaGVzdWIgPC0gc3Z5ZGVzaWduKGlkID0gflNETVZQU1UsIHdlaWdodHMgPSB+d2d0LCBzdHJhdGEgPSB+U0RNVlNUUkEsIG5lc3QgPSBUUlVFLCBkYXRhID0gZGZzdWJbWzVdXSkNCmNoZXN1YmwgPC0gc3Z5ZGVzaWduKGlkID0gflNETVZQU1UsIHdlaWdodHMgPSB+d2d0LCBzdHJhdGEgPSB+U0RNVlNUUkEsIG5lc3QgPSBUUlVFLCBkYXRhID0gZGZzdWJbWzZdXSkNCnh1c3ViIDwtIHN2eWRlc2lnbihpZCA9IH5TRE1WUFNVLCB3ZWlnaHRzID0gfndndCwgc3RyYXRhID0gflNETVZTVFJBLCBuZXN0ID0gVFJVRSwgZGF0YSA9IGRmc3ViW1s3XV0pDQp4dXN1YmwgPC0gc3Z5ZGVzaWduKGlkID0gflNETVZQU1UsIHdlaWdodHMgPSB+d2d0LCBzdHJhdGEgPSB+U0RNVlNUUkEsIG5lc3QgPSBUUlVFLCBkYXRhID0gZGZzdWJbWzhdXSkNCmBgYA0KDQphbmQgcmUtcnVuIHRoZSBtdWx0aS1saW5lYXIgcmVncmVzc2lvbnMgb24gdGhlIHN1YnNldCBvZiBlYWNoIGRhdGFzZXQgd2l0aG91dCB0aGUgaW5mbHVlbnRpYWwgcG9pbnRzLg0KDQpgYGB7cn0NCmdsbXN1YiA8LSBsaXN0KCkNCmdsbXN1YltbMV1dIDwtIHN2eWdsbShhc3RobWF+TUJ6UCwgZmFtaWx5ID0gcXVhc2liaW5vbWlhbCwgZGVzaWduID0gb2Rlc3ViKQ0KZ2xtc3ViW1syXV0gPC0gc3Z5Z2xtKGFzdGhtYX5sMTBNQnpQLCBmYW1pbHkgPSBxdWFzaWJpbm9taWFsLCBkZXNpZ24gPSBvZGVzdWJsKQ0KZ2xtc3ViW1szXV0gPC0gc3Z5Z2xtKGxvZ3J1YmVsbGF+cGZvYSArIFJJQUdFTkRSICsgUklEQUdFWVIgKyBlZDEgKyBlZDIgKyBCTVhCTUkgKyBldGgxICsgZXRoMiwgZGVzaWduID0gcGlsc3ViKQ0KZ2xtc3ViW1s0XV0gPC0gc3Z5Z2xtKGxvZ3J1YmVsbGF+bGcycGZvYSArIFJJQUdFTkRSICsgUklEQUdFWVIgKyBlZDEgKyBlZDIgKyBCTVhCTUkgKyBldGgxICsgZXRoMiwgZGVzaWduID0gcGlsc3VibCkNCmdsbXN1YltbNV1dIDwtIHN2eWdsbShMQlhUUn5IQmdhICsgZmVtYWxlICsgUklEQUdFWVIgKyBJTkRGTVBJUiArIHNtazEgKyBzbWsyICsgZGlhYmV0ZXNfcmV2ICsgaHlwZXJfcmV2ICsgZW5lcmd5ICsgZXRoMSArIGV0aDIgKyBldGgzICsgZXRoNCArIGVkdTEgKyBlZHUyICsgZWR1MyArIGVkdTUgKyBieDEgKyBieDIgKyBwYXFub25lICsgcGFxbW9kICsgYWxjb2hvbCwgZGVzaWduID0gY2hlc3ViKQ0KZ2xtc3ViW1s2XV0gPC0gc3Z5Z2xtKExCWFRSfmwyZ2EgKyBmZW1hbGUgKyBSSURBR0VZUiArIElOREZNUElSICsgc21rMSArIHNtazIgKyBkaWFiZXRlc19yZXYgKyBoeXBlcl9yZXYgKyBlbmVyZ3kgKyBldGgxICsgZXRoMiArIGV0aDMgKyBldGg0ICsgZWR1MSArIGVkdTIgKyBlZHUzICsgZWR1NSArIGJ4MSArIGJ4MiArIHBhcW5vbmUgKyBwYXFtb2QgKyBhbGNvaG9sLCBkZXNpZ24gPSBjaGVzdWJsKQ0KZ2xtc3ViW1s3XV0gPC0gc3Z5Z2xtKGN2ZH5MQlhJNUFMICsgUklEQUdFWVIgKyBldGgxICsgZXRoMiArIGV0aDQgKyBldGg1ICsgZWR1MiArIGVkdTMgKyBlZHU0ICsgZWR1NSArIGZlbWFsZSwgZmFtaWx5ID0gcXVhc2liaW5vbWlhbCwgZGVzaWduID0geHVzdWIpDQpnbG1zdWJbWzhdXSA8LSBzdnlnbG0oY3ZkfmwyaTVhbCArIFJJREFHRVlSICsgZXRoMSArIGV0aDIgKyBldGg0ICsgZXRoNSArIGVkdTIgKyBlZHUzICsgZWR1NCArIGVkdTUgKyBmZW1hbGUsIGZhbWlseSA9IHF1YXNpYmlub21pYWwsIGRlc2lnbiA9IHh1c3VibCkNCmBgYA0KDQpOb3cgd2Ugd2FudCB0byBjaGFuZ2UgdGhlIGJldGEgdmFsdWVzIGFuZCB0aGUgdmFsdWVzIG9mIHgtdmFyaWFibGUgY2VudHJhbCB0ZW5kZW5jeSBhbmQgc3ByZWFkIGZvciBvdXIgYWRqdXN0ZWQgZGF0YXNldCB3aXRoIHRoZSBpbmZsdWVudGlhbCBwb2ludHMgcmVtb3ZlZC4gRmlyc3QsIHRoZSBvcmlnaW5hbCBkYXRhIGZyYW1lIGlzIGNsb25lZCBpbnRvIGRhdGFhZGogYW5kIHRoZW4gdGhlIG5ldyBiZXRhIHZhbHVlcyBmb3IgdGhlIHN0dWRpZXMgd2l0aCBpbmZsdWVudGlhbCBwb2ludHMgcmVtb3ZlZCBhcmUgc3Vic3RpdHV0ZWQgaW4uDQoNCmBgYHtyfQ0KZGF0YWFkaiA8LSByZWFkLmNzdihmaWxlID0gImRhdGEvUmVfZXhwcmVzc2lvbl9GdWxsX1RhYmxlXzExMDcyMDIyLmNzdiIpDQoNCmRhdGFhZGpbZGF0YWFkaiRzdHVkeV9pZCA9PSAiT2RlYmVhdHUgMjAxOSIgJiBkYXRhYWRqJGlucHV0X2Jhc2UgPT0gImxpbiIsMTE6MTNdIDwtDQogIGMoc3VtbWFyeShnbG1zdWJbWzFdXSkkY29lZlsyLDFdLCBjb25maW50KGdsbXN1YltbMV1dKVsyLF0pDQpkYXRhYWRqW2RhdGFhZGokc3R1ZHlfaWQgPT0gIk9kZWJlYXR1IDIwMTkiICYgZGF0YWFkaiRpbnB1dF9iYXNlID09ICJsb2ciLDExOjEzXSA8LQ0KICBjKHN1bW1hcnkoZ2xtc3ViW1syXV0pJGNvZWZbMiwxXSwgY29uZmludChnbG1zdWJbWzJdXSlbMixdKQ0KZGF0YWFkaltkYXRhYWRqJHN0dWR5X2lkID09ICJQaWxrZXJ0b24gMjAxOCIgJiBkYXRhYWRqJGlucHV0X2Jhc2UgPT0gImxpbiIsMTE6MTNdIDwtDQogIGMoc3VtbWFyeShnbG1zdWJbWzNdXSkkY29lZlsyLDFdLCBjb25maW50KGdsbXN1YltbM11dKVsyLF0pDQpkYXRhYWRqW2RhdGFhZGokc3R1ZHlfaWQgPT0gIlBpbGtlcnRvbiAyMDE4IiAmIGRhdGFhZGokaW5wdXRfYmFzZSA9PSAibG9nIiwxMToxM10gPC0NCiAgYyhzdW1tYXJ5KGdsbXN1YltbNF1dKSRjb2VmWzIsMV0sIGNvbmZpbnQoZ2xtc3ViW1s0XV0pWzIsXSkNCmRhdGFhZGpbZGF0YWFkaiRzdHVkeV9pZCA9PSAiQ2hlYW5nIDIwMjEiICYgZGF0YWFkaiRpbnB1dF9iYXNlID09ICJsaW4iLDExOjEzXSA8LQ0KICBjKHN1bW1hcnkoZ2xtc3ViW1s1XV0pJGNvZWZbMiwxXSwgY29uZmludChnbG1zdWJbWzVdXSlbMixdKQ0KZGF0YWFkaltkYXRhYWRqJHN0dWR5X2lkID09ICJDaGVhbmcgMjAyMSIgJiBkYXRhYWRqJGlucHV0X2Jhc2UgPT0gImxvZyIsMTE6MTNdIDwtDQogIGMoc3VtbWFyeShnbG1zdWJbWzZdXSkkY29lZlsyLDFdLCBjb25maW50KGdsbXN1YltbNl1dKVsyLF0pDQpkYXRhYWRqW2RhdGFhZGokc3R1ZHlfaWQgPT0gIlh1IDIwMjBhIiAmIGRhdGFhZGokaW5wdXRfYmFzZSA9PSAibGluIiwxMToxM10gPC0NCiAgYyhzdW1tYXJ5KGdsbXN1YltbN11dKSRjb2VmWzIsMV0sIGNvbmZpbnQoZ2xtc3ViW1s3XV0pWzIsXSkNCmRhdGFhZGpbZGF0YWFkaiRzdHVkeV9pZCA9PSAiWHUgMjAyMGEiICYgZGF0YWFkaiRpbnB1dF9iYXNlID09ICJsb2ciLDExOjEzXSA8LQ0KICBjKHN1bW1hcnkoZ2xtc3ViW1s4XV0pJGNvZWZbMiwxXSwgY29uZmludChnbG1zdWJbWzhdXSlbMixdKQ0KDQpkYXRhYWRqW2RhdGFhZGokc3R1ZHlfaWQgPT0gIk9kZWJlYXR1IDIwMTkiICYgZGF0YWFkaiRpbnB1dF9iYXNlID09ICJsaW4iLDQ6Nl0gPC0gYyhtZWRpYW4oZGZzdWJbWzFdXSRNQnpQLG5hLnJtID0gVCkscXVhbnRpbGUoZGZzdWJbWzFdXSRNQnpQLDAuMjUsbmEucm0gPSBUKVtbMV1dLHF1YW50aWxlKGRmc3ViW1sxXV0kTUJ6UCwwLjc1LG5hLnJtID0gVClbWzFdXSkNCmRhdGFhZGpbZGF0YWFkaiRzdHVkeV9pZCA9PSAiT2RlYmVhdHUgMjAxOSIgJiBkYXRhYWRqJGlucHV0X2Jhc2UgPT0gImxvZyIsNDo2XSA8LSBjKG1lZGlhbihkZnN1YltbMl1dJE1CelAsbmEucm0gPSBUKSxxdWFudGlsZShkZnN1YltbMl1dJE1CelAsMC4yNSxuYS5ybSA9IFQpW1sxXV0scXVhbnRpbGUoZGZzdWJbWzJdXSRNQnpQLDAuNzUsbmEucm0gPSBUKVtbMV1dKQ0KZGF0YWFkaltkYXRhYWRqJHN0dWR5X2lkID09ICJQaWxrZXJ0b24gMjAxOCIgJiBkYXRhYWRqJGlucHV0X2Jhc2UgPT0gImxpbiIsOToxMF0gPC0gYyhtZWFuKGRmc3ViW1szXV0kcGZvYSxuYS5ybSA9IFQpLHNkKGRmc3ViW1szXV0kcGZvYSxuYS5ybSA9IFQpKQ0KZGF0YWFkaltkYXRhYWRqJHN0dWR5X2lkID09ICJQaWxrZXJ0b24gMjAxOCIgJiBkYXRhYWRqJGlucHV0X2Jhc2UgPT0gImxvZyIsOToxMF0gPC0gYyhtZWFuKGRmc3ViW1s0XV0kcGZvYSxuYS5ybSA9IFQpLHNkKGRmc3ViW1s0XV0kcGZvYSxuYS5ybSA9IFQpKQ0KZGF0YWFkaltkYXRhYWRqJHN0dWR5X2lkID09ICJDaGVhbmcgMjAyMSIgJiBkYXRhYWRqJGlucHV0X2Jhc2UgPT0gImxpbiIsNDo2XSA8LSBjKG1lZGlhbihkZnN1YltbNV1dJEhCZ2EsbmEucm0gPSBUKSxxdWFudGlsZShkZnN1YltbNV1dJEhCZ2EsMC4yNSxuYS5ybSA9IFQpW1sxXV0scXVhbnRpbGUoZGZzdWJbWzVdXSRIQmdhLDAuNzUsbmEucm0gPSBUKVtbMV1dKQ0KZGF0YWFkaltkYXRhYWRqJHN0dWR5X2lkID09ICJDaGVhbmcgMjAyMSIgJiBkYXRhYWRqJGlucHV0X2Jhc2UgPT0gImxvZyIsNDo2XSA8LSBjKG1lZGlhbihkZnN1YltbNl1dJEhCZ2EsbmEucm0gPSBUKSxxdWFudGlsZShkZnN1YltbNl1dJEhCZ2EsMC4yNSxuYS5ybSA9IFQpW1sxXV0scXVhbnRpbGUoZGZzdWJbWzZdXSRIQmdhLDAuNzUsbmEucm0gPSBUKVtbMV1dKQ0KZGF0YWFkaltkYXRhYWRqJHN0dWR5X2lkID09ICJYdSAyMDIwYSIgJiBkYXRhYWRqJGlucHV0X2Jhc2UgPT0gImxpbiIsNDo2XSA8LSBjKG1lZGlhbihkZnN1YltbN11dJExCWEk1QUwsbmEucm0gPSBUKSxxdWFudGlsZShkZnN1YltbN11dJExCWEk1QUwsMC4yNSxuYS5ybSA9IFQpW1sxXV0scXVhbnRpbGUoZGZzdWJbWzddXSRMQlhJNUFMLDAuNzUsbmEucm0gPSBUKVtbMV1dKQ0KZGF0YWFkaltkYXRhYWRqJHN0dWR5X2lkID09ICJYdSAyMDIwYSIgJiBkYXRhYWRqJGlucHV0X2Jhc2UgPT0gImxvZyIsNDo2XSA8LSBjKG1lZGlhbihkZnN1YltbOF1dJExCWEk1QUwsbmEucm0gPSBUKSxxdWFudGlsZShkZnN1YltbOF1dJExCWEk1QUwsMC4yNSxuYS5ybSA9IFQpW1sxXV0scXVhbnRpbGUoZGZzdWJbWzhdXSRMQlhJNUFMLDAuNzUsbmEucm0gPSBUKVtbMV1dKQ0KYGBgDQoNCldlIHRoZW4gcmUtcnVuIHRoZSBzYW1lIGFuYWx5c2lzIGFzIGRvbmUgcHJldmlvdXNseS4NCg0KYGBge3J9DQpkem91dGFkaiA8LSBkYXRhLmZyYW1lKCJzdHVkeSIgPSBkYXRhYWRqJHN0dWR5X2lkLCAiaW5wdXRfYmFzZSIgPSBkYXRhYWRqJGlucHV0X2Jhc2UsICJhY3R1YWxfYmV0YSIgPSBkYXRhYWRqJG1hbnVhbF9iZXRhLCAiYWN0dWFsX2JsY2kiID0gZGF0YWFkaiRtYW51YWxfYmxjaSwgImFjdHVhbF9idWNpIiA9IGRhdGFhZGokbWFudWFsX2J1Y2ksICJiZXRhIiA9IGMoMTpucm93KGRhdGFhZGopKSwgImJldGFfbGNpIiA9IGMoMTpucm93KGRhdGFhZGopKSwgImJldGFfdWNpIiA9IGMoMTpucm93KGRhdGFhZGopKSkNCmZvcihpIGluIDE6bnJvdyhkYXRhYWRqKSl7DQogIGR6b3V0YWRqW2ksNjo4XSA8LSBkemV4cChpbnB1dC5iYXNlID0gZGF0YWFkaiRpbnB1dF9iYXNlW2ldLCANCiAgICAgICAgICAgICAgICAgICAgICAgIGV4cC5kaXN0ID0gYyhtZWQgPSBkYXRhYWRqJGV4cF9tZWRbaV0sIGlxcjEgPSBkYXRhYWRqJGV4cF8xcVtpXSwgaXFyMyA9IGRhdGFhZGokZXhwXzNxW2ldLA0KICAgICAgICAgICAgICAgICAgICAgICAgICAgICAgICAgICAgIGdtZWFuID0gZGF0YWFkaiRleHBfZ21lYW5baV0sIGdzZCA9IGRhdGFhZGokZXhwX2dzZFtpXSwNCiAgICAgICAgICAgICAgICAgICAgICAgICAgICAgICAgICAgICBtZWFuID0gZGF0YWFkaiRleHBfbWVhbltpXSwgc2QgPSBkYXRhYWRqJGV4cF9zZFtpXSksDQogICAgICAgICAgICAgICAgICAgICAgICBiZXRhLmRpc3QgPSBjKGRhdGFhZGokYmV0YV9tZWFuW2ldLCBkYXRhYWRqJGJldGFfbGNpW2ldLCBkYXRhYWRqJGJldGFfdWNpW2ldKSwNCiAgICAgICAgICAgICAgICAgICAgICAgIGxvZy5iYXNlID0gZGF0YWFkaiRsb2diYXNlW2ldKQ0KfQ0KZHpvdXRhZGokbWV0aG9kIDwtICJEemllcmxlbmdhIE1ldGhvZCINCg0KZGZvdXRhZGogPC0gZHpvdXRhZGoNCmRmb3V0YWRqJHByb3BfZGlmZl9iZXRhIDwtIChkZm91dGFkaiRiZXRhL2Rmb3V0YWRqJGFjdHVhbF9iZXRhKS0xDQpgYGANCg0KRmluYWxseSB3ZSBpbnRlZ3JhdGUgdGhlIG9sZCBhbmQgbmV3IHByb3BvcnRpb25hbCBkaWZmZXJlbmNlIGluIGJldGEgYW5kIGZvcm1hdCBUYWJsZSBTNi4NCg0KYGBge3J9DQpkZm91dCRwcm9wX2RpZmZfYmV0YV9hZGogPC0gZGZvdXRhZGokcHJvcF9kaWZmX2JldGENCnRzNnN0dWR5bGlzdCA8LSBjKCJPZGViZWF0dSAyMDE5IiwiUGlsa2VydG9uIDIwMTgiLCJDaGVhbmcgMjAyMSIsIlh1IDIwMjBhIikNCnRhYnM2IDwtIGRmb3V0W2Rmb3V0JHN0dWR5ICVpbiUgdHM2c3R1ZHlsaXN0ICYgZGZvdXQkbWV0aG9kID09ICJEemllcmxlbmdhIE1ldGhvZCIsXQ0KdGFiczYgPC0gdGFiczZbb3JkZXIobWF0Y2godGFiczYkc3R1ZHksdHM2c3R1ZHlsaXN0KSksXQ0KdGFiczYgPC0gdGFiczZbYygic3R1ZHkiLCJpbnB1dF9iYXNlIiwibWV0aG9kIiwicHJvcF9kaWZmX2JldGEiLCJwcm9wX2RpZmZfYmV0YV9hZGoiKV0NCnRhYnM2IDwtIHRhYnM2ICU+JSBtdXRhdGVfaWYoaXMubnVtZXJpYywgcm91bmQsIDMpDQp0YWJzNiA8LSB0YWJzNlt0YWJzNiRpbnB1dF9iYXNlID09ICJsb2ciLF0NCnRhYnM2DQpgYGANCg0KIyBUYWJsZSBTbmV3MQ0KYGBge3J9DQppbmRpdnN0dWQgPC0gZGF0YVshZHVwbGljYXRlZChkYXRhJHN0dWR5X2lkKSxdDQppbmRpdnN0dWQgPC0gaW5kaXZzdHVkWyxjKDEsMTksMTQsMTgsMjAsMjEpXQ0KYGBgDQoNCiMgVGFibGUgU25ldzFiDQpgYGB7cn0NCmluZGl2c3R1ZGIgPC0gZGF0YWJbIWR1cGxpY2F0ZWQoZGF0YWIkc3R1ZHlfaWQpLF0NCmluZGl2c3R1ZGIgPC0gaW5kaXZzdHVkYlssYygxLDE5LDE0LDE4LDIwLDIxKV0NCmBgYA0K
