# Supplementary material for: Re-expressing coefficients from regression models for inclusion in a meta-analysis
Source: BMC Med Res Methodol. 2024 Jan 8;24:6. doi: 10.1186/s12874-023-02132-y (PMC10773134; doi:10.1186/s12874-023-02132-y)
Supplement: Supplementary file 2 — Additional file 2. [file 12874_2023_2132_MOESM2_ESM.zip › Supp_Files_R1/Rex_Supplemental_Figures_and_Tables.pdf]

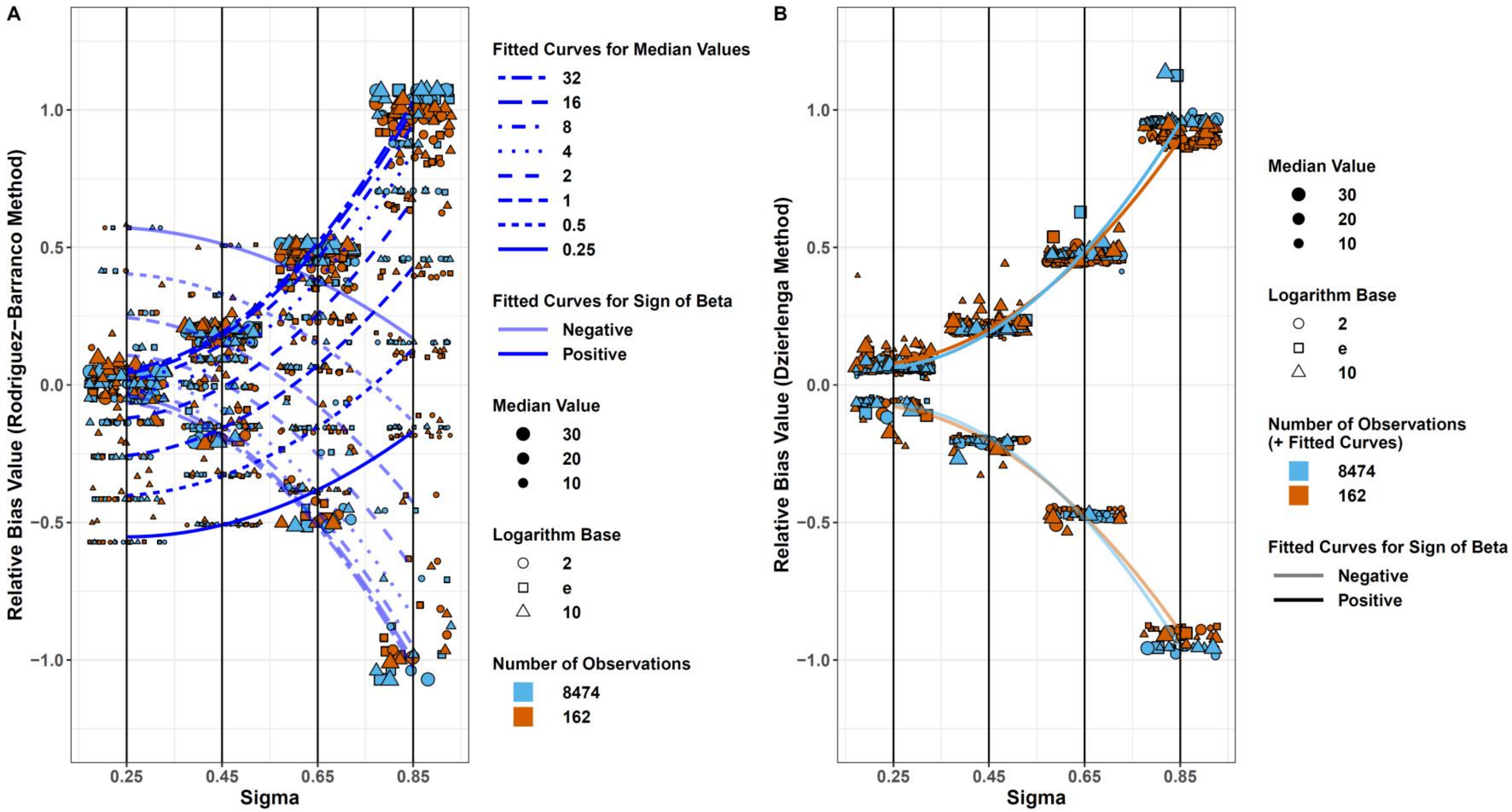
A
B
Relative Bias Value (Rodriguez–Barranco Method)
Relative Bias Value (Dzierlenga Method)
Sigma
0.25
0.45
0.65
0.85
1.0
0.5
0.0
−0.5
−1.0
Fitted Curves for Median Values
32
16
8
4
2
1
0.5
0.25
Fitted Curves for Sign of Beta
Negative
Positive
Median Value
30
20
10
Logarithm Base
2
e
10
Number of Observations
8474
162
Number of Observations (+ Fitted Curves)

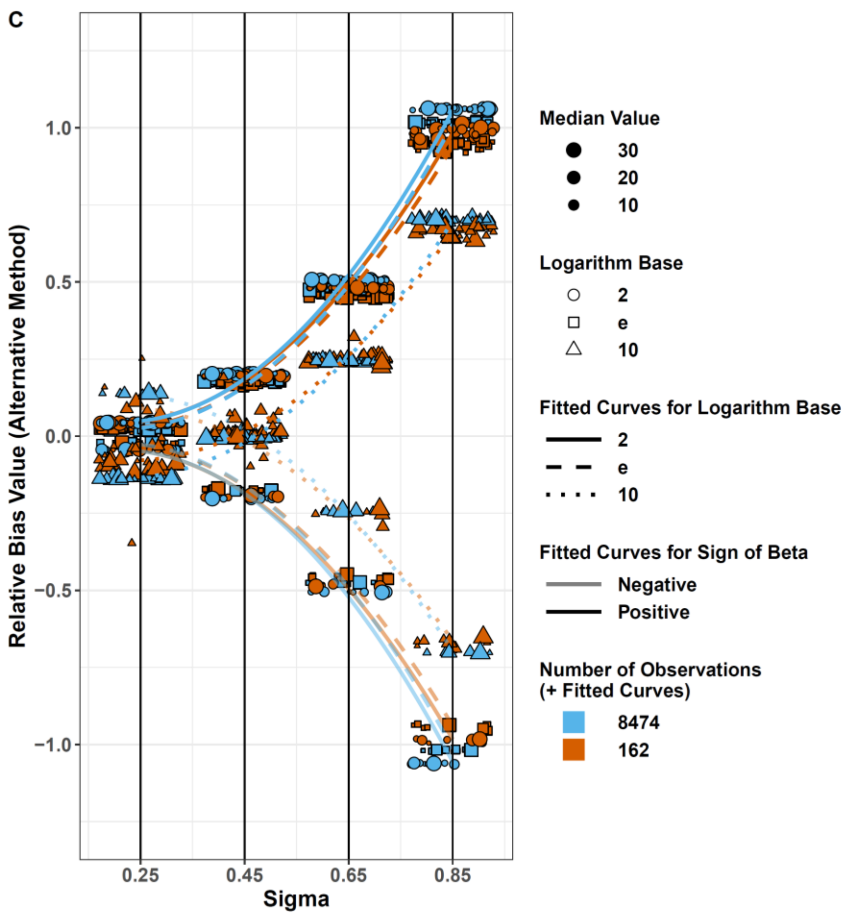

Figure S1: Plots of relative bias as a function of skewness (σ) in the exposure x, by type of estimator, including the scenario where $\beta_{DGM} < 0$. Individual points represent the average result (n_{sim} = 2000) for each simulation scenario. A total of 890 of the possible 1,920,000 observations (960 scenarios x 2000 simulations) were not used in the calculation of the average results because $\beta_{estimand}$ was < 0.0001 (essentially zero). Lines represent quadratic fits to the data for a specified prediction equation and set of values of independent variables (see text). Note that data have been artificially spread along the x-axis for visualization purposes, all actual x-values are the closest black vertical line (0.25, 0.45, 0.65, or 0.85). Figures A-C show points for 960 simulations. A) Rodriguez-Barranco estimator, B) Dzierlenga estimator, and C) Alternative estimator.

Table S1: Coefficients from ordinary least squares models of relative bias, by re-expression method. Each method was described by a quadratic fit (ax^2+bx+c) with additional predictor variables as described in the table.

Method	Parameter	Coefficient	Standard Error	*p*-value	RMSE	Adjusted R^2
**Rodriguez-Barranco Method (β_{RB})**	Intercept (c)	-0.13	0.0596	0.026	0.230	0.706
	Linear sigma term (b)	-0.95	0.2342	<0.001		
	Quadratic sigma term (a)	1.87	0.2085	<0.001		
	Median	0.01	0.0021	0.001		
	Interaction term between median and sigma (μ:σ)	0.02	0.0036	<0.001		
**Dzierlenga Method (β_{Dz})**	Intercept (c)	0.18	0.0076	<0.001	0.028	0.993
	Linear σ term (b)	-0.84	0.0283	<0.001		
	Quadratic σ term (a)	2.00	0.0251	<0.001		
	Number of observations in simulated study (nobs)	-7.02E-06	6.40E-07	<0.001		
	logbase (logbase = 10)	0.0167	0.0025	<0.001		
	logbase (logbase = 2)	-0.0026	0.0025	0.29		
	β_{DGM}	0.0002	8.39E-05	0.004		
	Median	0.0005	9.68E-05	<0.001		
	Interaction term between nobs and sigma (nobs:σ)	1.51E-05	1.08E-06	<0.001		
**Alternative Method (β_{Alt})**	Intercept (c)	0.13	0.0071	<0.001	0.024	0.996
	Linear σ term (b)	-0.90	0.0247	<0.001		

	Quadratic σ term (a)	2.20	0.0214	<0.001	
	logbase (logbase = 10)	-0.05	0.0056	<0.001	
	logbase (logbase = 2)	9.41E-03	0.0056	0.091	
	number of observations in simulated study (nobs)	-7.21E-06	5.46E-07	<0.001	
	Interaction term between σ and logbase = 10 (logbase10: σ)	-0.27	0.0094	<0.001	
	Interaction term between σ and logbase = 2 (logbase2: σ)	0.04	0.0094	<0.001	
	Interaction term between σ and nobs (σ:nobs)	1.56E-05	9.20E-07	<0.001	

Table S2: Comparison of model fits (R^2) when using each simulation scenario with $\beta_{DGM} > 0$ (n = 768) or using each observation with $\beta_{DGM} > 0$ (n = 768 * 2000)

r^2 for:	β_{RB}	β_{Dz}	β_{Alt}
**n = 768**	0.706	0.992	0.996
**n = 768 * 2000**	0.709	0.992	0.996

Table S3. Published analyses of an outcome in relation to a biomarker-based measure of environmental exposure, with raw data available

1st author, year	Outcome	Type of outcome[a]	Exposure[b]	Original unit of exposure	Result presented by original authors[c]	Our result[c] (re-analysis of raw data)
Bulka, 2021	Herpes Simplex Virus 2	D	PFOA	Log_2	1.11 (1.05, 1.17)	1.11 (1.05, 1.17)
Lee, 2020	Infertility	D	Cadmium	Log_2	1.8 (1.1, 3.1)	1.8 (1.1, 3.1)
Odebeatu, 2019	Asthma	D	Mono-benzyl phthalate (urine)	Log_{10}	1.50 (1.09, 2.08)	1.50 (1.08, 2.08)
Xu, 2020	CVD[d]	D	Isopentanaldehyde	ng/ml	P<0.001	1.67 (1.17, 2.25)
Xu, 2020	Triglycerides (mg/dl)	C	Isopentanaldehyde	ng/ml	25.0 (4.8, 45.1)	29.3 (14.1, 44.6)
Stein, 2016	Mumps IgG[e] (%Δ)	C	PFOS	Log_2	-7.4 (-12.8, -1.7)	-10.3 (-19.3, -0.024)
Pilkerton, 2018	Rubella IgG[e] (%Δ)	C	PFOA	Log_2[f]	-8.9 (-16.9, -0.2)[f]	N.A.
Cheang, 2021	Triglycerides (mg/dl)	C	Glycidamide[g]	Log_2	11.4 (5.1, 17.7)	9.65 (1.62, 17.7)
Abraham, 2020	Ln(Hib[h] IgG[e])	C	PFOA	Log_2	-0.3887[i] (-0.6957, -0.0817)	N.A.

[a] C = continuous, D = dichotomous

[b] Measured in serum unless noted otherwise

[c] Results shown are from regression analyses. For dichotomous outcomes, these are odds ratios (and 95% confidence intervals). For continuous outcome, the results are either regression coefficients, or regression results re-expressed as percent difference (%Δ) in outcome per unit exposure

[d] CVD, cardiovascular disease

[e] IgG, immunoglobulin G

[f] The original units were quartiles; we re-analyzed the data to get the original result shown, in percent change in Rubella antibody per log_2 increase in PFOA. See Crawford et al. (reference), Supplementary Material, Section X for an account of the re-analysis.

[g] As reflected by concentration of hemoglobin adduct of glycidamide (HbGA)

[h] Hib, Hemophilus Influenza

[i] Our analysis of the Abraham data. See Crawford et al. (submitted, 2021), Supplementary Material, Section S7 for an account of the re-analysis.

Table S4. Additional details about the 15 example studies

Study, Year	Specific Finding (Location, Outcome)	Exposure Distribution				
		Median	1st Quartile	3rd Quartile	σ (lognormal distribution)	Mean
Abraham 2020	Our analysis, Hib IgG	14.3[a]	6.70	19.3	0.78	16.8
Apelberg, 2007	Table 3 (Fully Adjusted), Birth Weight (g)	5.00[b]	3.40	7.90	0.62	5.43[f]
Bulka, 2021	Table 3 (20-49 y), HSV 2	2.77[a]	1.67	4.6	0.75	3.0[f]
Cheang, 2021	Table 3, Triglycerides (mg/dL)	38.7[a]	29.4	55.2	0.47	41.1[f]
Chen, 2012	Table 3 (Adjusted), Birth Weight (g)	5.94[e]	3.94	8.94	0.61	6.27[f]
Darrow, 2013	Table 6 (Adjusted All Births, Per in unit increase), Birth Weight (g)	13.9[b]	9.5	19.7	0.54	14.4[f]
Hamm, 2010	Table 5 (Hamm, PFOS), Birth Weight (g)	7.80[d]	5.70	10.7	0.47	8.07[f]
Lee, 2020	Table 2 (Model 2), Infertility	0.240[a]	0.14	0.43	0.83	0.270[f]
Odebeatu, 2019	Figure 1a (MBzP), Asthma	12.3[b]	5.00	27.3	1.26	14.9[f]
Pilkerton, 2018	Table 4, Rubella (%Δ)	4.30[a]	3.00	6.3	0.55	6.00
Steenland 2009	Table 4, Total Cholesterol	20.2[c]	13.6	29.3	0.57	22.4
Stein, 2016c	Table 2, Mumps (%Δ)	22.2[a]	15.35	30.8	0.52	22.8[f]
Xu, 2020	Table 2 (Model 1), CVD	0.521[a]	0.346	1.03	0.81	0.632[f]
Xu, 2020	Table 4 (Model 2), Triglycerides (mg/dL)	0.521[a]	0.346	1.03	0.81	0.632[f]
Washino, 2009	Table 5 (Fully Adjusted), Birth Weight (g)	5.20[b]	3.40	7.00	0.53	5.20[f]

[a]Median and IQR calculated from raw data

[b]Median and IQR pulled from publication

[c]Median from publication and IQR adjusted from Steenland 2010 by subtracting 0.6 (difference between medians in studies)

[d]Median from publication and IQR adjusted from Lind 2017, by subtracting 0.3 (difference between medians in studies)

[e]Median from publication and IQR adjusted from Chen 2017 by adding 0.24 (difference between medians in studies)

[f]Mean estimated from median and IQR using the formula from Wan 2014: (Q1+Median+Q3)/3

Table S5. Published analyses of an outcome in relation to a biomarker-based measure of environmental exposure, where the original authors presented results using exposure with and without a log-transformation

1st author, year	Outcome	Type of outcome	Exposure	Logarithmic (β in units/log(ng/ml)) Result reported by original authors[a]	Untransformed (β in units/(ng/ml)) Result reported by original authors[b]
Apelberg, 2007	Birth weight (g)	C	PFOS	-69 (-149, 10)	-12.9 (-27.8, 2)
Chen, 2012	Birth weight (g)	C	PFOS	-110.2 (-176.0, -44.5)	-11.30 (-17.40, -5.20)
Darrow, 2013	Birth weight (g)	C	PFOS	-29 (-66, 7)	-2.3 (-4.8, 0.3)
Hamm, 2010	Birth weight (g)	C	PFOS	31.3 (-43.3, 105.9)	1.5 (-7.6, 10.6)
Steenland, 2009	Ln(Serum cholesterol (mg/dl))	C	PFOS	0.0266 (0.0239, 0.0293)	0.00105 (0.0009, 0.0012)
Washino, 2009	Birth weight (g)	C	PFOS	-148.8 (-297.0, -0.5)	-10.94 (-22.9, 1.10)

[a] Values shown are for $\log_e$ transformations, except for Washino et al, for which a $\log_{10}$ transformation was used. Values in parentheses are 95% confidence intervals.
[b] Values for Washino et al. (2009) and Chen et al. (2012) in this column were presented in Verner et al. (2015).

Table S6 Comparison of re-expression outcome according to whether influential observations were included in the data

1st author, year	Proportional difference with influential observations	Proportional difference with no influential observations
Odebeatu 2019	16.82	19.58
Pilkerton 2018	5.17	2.18
Cheang 2021	0.84	1.11
Xu 2020a	0.88	1.38
